# Rapid Assembly of Pyrrole-Ligated 1,3,4-Oxadiazoles and Excellent Antibacterial Activity of Iodophenol Substituents

**DOI:** 10.3390/molecules28083638

**Published:** 2023-04-21

**Authors:** Hyein Kim, Lina Gu, Huisu Yeo, Umji Choi, Chang-Ro Lee, Haiyang Yu, Sangho Koo

**Affiliations:** 1 Department of Chemistry, Myongji University, Myongji-Ro 116, Cheoin-Gu, Yongin 17058, Gyeonggi-Do, Republic of Korea; 2 School of Pharmacy, East China University of Science and Technology, Meilong Road 130, Shanghai 200237, China; 3 Department of Biological Sciences and Bioinformatics, Myongji University, Myongji-Ro 116, Cheoin-Gu, Yongin 17058, Gyeonggi-Do, Republic of Korea; 4 Tianjin State Key Laboratory of Modern Chinese Medicine, Tianjin University of Traditional Chinese Medicine, Tianjin 300193, China

**Keywords:** pyrrole, 1,3,4-oxadiazole, Maillard reaction, D-ribose, L-amino acid, pyrrole-2-carbaldehyde, iodine effect, antibacterial activity

## Abstract

Pyrrole-ligated 1,3,4-oxadiazole is a very important pharmacophore which exhibits broad therapeutic effects such as anti-tuberculosis, anti-epileptic, anti-HIV, anti-cancer, anti-inflammatory, antioxidant, and antibacterial activities. A one-pot Maillard reaction between D-Ribose and an L-amino methyl ester in DMSO with oxalic acid at 2.5 atm and 80 °C expeditiously produced pyrrole-2-carbaldehyde platform chemicals in reasonable yields, which were utilized for the synthesis of pyrrole-ligated 1,3,4-oxadiazoles. Benzohydrazide reacted with the formyl group of the pyrrole platforms to provide the corresponding imine intermediates, which underwent I_2_-mediated oxidative cyclization to the pyrrole-ligated 1,3,4-oxadiazole skeleton. The structure and activity relationship (SAR) of the target compounds with varying alkyl or aryl substituents of the amino acids and electron-withdrawing or electron-donating substituents on the phenyl ring of benzohydrazide were evaluated for antibacterial activity against *Escherichia coli*, *Staphylococcus aureus*, and *Acinetobacter baumannii* as representative Gram(–) and Gram(+) bacteria. Branched alkyl groups from the amino acid showed better antibacterial activities. Absolutely superior activities were observed for **5f-1** with an iodophenol substituent against *A. baumannii* (MIC < 2 μg/mL), a bacterial pathogen that displays a high resistance to commonly used antibiotics.

## 1. Introduction

Oxadiazole is a five-membered heterocyclic aromatic compound composed of four structural isomers depending on the positions of two nitrogen atoms relative to an oxygen atom [1]. Among them, 1,3,4-oxadiazole has received intensive attention in the field of medicinal chemistry due to its broad metabolic profile [2,3,4] and in the field of material science for its excellent optoelectronic properties [5,6,7,8]. As an isostere of an amide and an ester, 1,3,4-oxadiazole serves as a promising pharmacophore for the discovery of new drugs exhibiting antimicrobial, anticonvulsant, anti-inflammatory, analgesic, antitumor, antiviral, antihypertensive, and enzyme inhibitory activities [9]. There have been extensive literature reviews on the specific synthetic methods and diverse biological activities of 1,3,4-oxadiazole derivatives [10,11,12].

**Figure 1:**
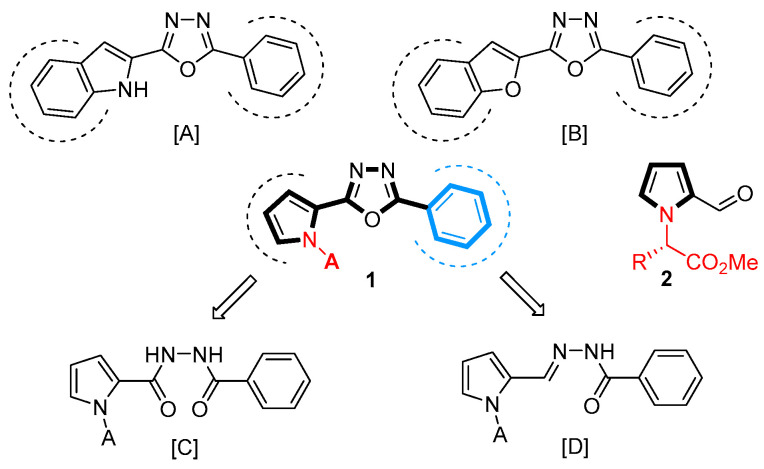
Heterocycle-ligated 1,3,4-oxadiazoles [A] and [B], and retrosynthesis of **1** from pyrrole **2** through the cyclization using key precursors [C] and [D].

Motivated by Raltegravir [13,14], an antiretroviral drug used to treat HIV/AIDS, and Zibotentan [15,16], an anti-cancer drug candidate, a number of poly heterocyclic compounds containing a 1,3,4-oxadiazole pharmacophore were constructed, and their biological activities were investigated. Indole-ligated 1,3,4-oxadiazoles [A] were synthesized and evaluated to show antimicrobial, anti-inflammatory, and antiproliferative activities (Figure 1) [17,18]. Their biological importance was also identified by their antioxidants and acetylcholinesterase inhibition properties [19]. Likewise, various 2-benzofuranyl-1,3,4-oxadiazoles [B] were synthesized [20,21], demonstrating their biological activities in α-Glucosidase inhibition as well as the inhibition of glycogen synthase kinase 3*β* for treating diabetes and Alzheimer’s disease, respectively [22,23,24].

Pyrrole is a very important structural motif in drug discovery projects because of the wide presence of natural, biologically active pyrrole alkaloid products [25,26]; thus, pyrrole-ligated 1,3,4-oxadiazole would be a perfect base structure for the development of potential lead compounds [27]. Considering the efficacy of the procedures of constructing a 1,3,4-oxadiazole ring, 2-pyrrolyl-5-phenyl-1,3,4-oxadizole **1** would be an ideal core structure, achieved either through dehydration from aroylhydrazide [C] or by cyclization from aroylhydrazone [D]. In the benzene ring, the substituent effects of the core structure **1** on antibacterial activity have been reported for the cases of 4,5-dibromopyrrole [28,29] and 4-nitropyrrole [30], respectively.

Pyrrole-2-carbaldehydes **2**, derived from the conversion of D-ribose with L-amino acids [31], were demonstrated to be useful, sustainable platform chemicals for the construction of highly functionalized poly heterocyclic compounds [32]. Since natural amino acids themselves demonstrate specific biological activities [33,34,35], it was envisioned that 1,3,4-oxadiazoles **1** from pyrrole-2-carbaldehydes **2** with the *N*-amino acid moiety would be very interesting core structures for the investigation of their biological activities. The effect of the amino acid moiety of **1** on antimicrobial activities was screened first, and the substituent effects on the benzene ring were then investigated for **5** and **6** with some selected amino acid moieties. We found a marginal size effect of the alkyl groups from amino acids (Val and Ile, etc.) and superior antibacterial activity of the iodophenol substituents of pyrrole-ligated 1,3,4-oxadiazoles **5** and **6** against *S. aureus* and *A. baumannii*. All the syntheses of pyrrole-ligated 1,3,4-oxadiazoles **1**, **5**, and **6** and their antibacterial activities are closely described herein.

## 2. Results and Discussion

**Scheme 1:**
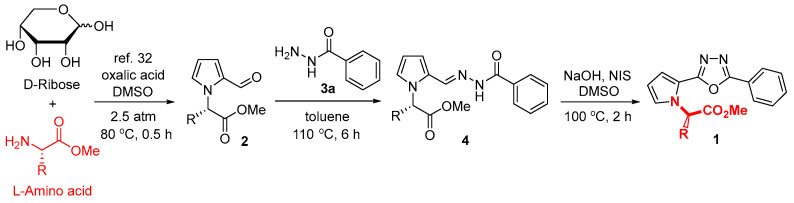
Preparations of pyrrole-2-carbaldehydes **2**, *N*-benzoylhydrazones **4**, and 1,3,4-oxadiazoles **1** from D-ribose conversion with L-amino acids.

Structure and activity relationships (SARs) for pyrrole-ligated 1,3,4-oxadiazoles **1** were generally studied by changing substituent groups in the aromatic rings [28,29,30]. We were interested in the SAR of **1** by *N*-alkyl substituents because pyrrole-2-carbaldehydes **2**, the starting materials for 1,3,4-oxadiazoles **1**, are easily prepared from L-amino acids, and each amino acid has its own biological activity. Ten L-amino acids with hydrogen, alkyl, aralkyl, ester, and sulfide substituents R were selected to assess the size effect (linear or branched) or potential electronic effect. Pyrrole-2-carbaldehydes **2** were efficiently prepared by a one-pot ribose conversion with an L-amino methyl ester in the presence of oxalic acid in DMSO, following an improved procedure under 2.5 atm argon at 80 °C [32]. The corresponding pyrrole platform chemicals **2a**–**2j** with the *N*-amino acid moiety were prepared in yields of 32~63% (Scheme 1 and Table 1).

Two representative procedures are generally utilized for the construction of the 1,3,4-oxadizole core, as depicted in Figure 1 [12]. The cyclodehydration route from diacylhydrazine [C] is suitable for pyrrole-2-carboxylic acids [36], whereas the oxidative cyclization route from *N*-acylhydrazone [D] is widely used for pyrrole-2-carbaldehydes as starting materials [37]. There were various cyclization conditions for 1,3,4-oxadiazoles reported for each conversion [12]. We adopted the oxidative cyclization route of *N*-acylhydrazones **4**, which can be obtained from pyrrole-2-carbaldehydes **2** by condensation with benzohydrazide **3a**. The corresponding *N*-benzoylhydrazones **4a**–**4j** were obtained in decent yields (70~96%) at the reflux temperature of toluene. Oxidative cyclization conditions were then screened using NBS, NIS, and I_2_ under K_2_CO_3_, DBU, Et_3_N, and NaOH as a base. The condition using NIS/NaOH in DMSO at 100 °C was optimal for providing pyrrole-ligated 1,3,4-oxadiazoles **1** in yields of 80~98%. It is noteworthy that the NIS-mediated further cyclization of the methylsulfide chain on the pyrrole ring occurred partly for **1j** derived from methionine to produce **1k** (at a yield of 49%), which explains the lower yield of **1j** (50%). All eleven pyrrole-ligated oxadiazoles **1a**–**1j** were rapidly assembled from pyrrole platform chemicals **2** with different amino acid residues and ready for antibacterial assays against *Escherichia coli* and *Staphylococcus aureus* as two representative Gram(–) and Gram(+) bacteria (Table 2).

There was a definite size effect of the alkyl substituent R on antibacterial activity in 1,3,4-oxadiazoles **1**. The highest MIC value was required for **1a** from glycine (R = H), and it decreased as the size (branch) of the alkyl group increased from alanine (R = Me) to isoleucine (R = *s*-Bu) (entries 1–5, Table 2). A benzene ring seemed be unimportant, judging from the cases of the benzyl and homobenzyl substituents (entries 6–7). There was no functional group effect for the ester and sulfide, reflecting a lack of electronic interactions between the substituent R and the bacterial enzymes. A comparison of the MIC values for **1j** and its cyclized derivative **1k** confirmed the importance of the size (or rigidity) effect of R on antibacterial activity (entries 10 and 11). An additional point to mention is that the MIC values for **1** were not much different between Gram(–) and Gram(+) bacteria, indicating that there would be no transport barriers through membranes for these small molecules.

**Scheme 2:**
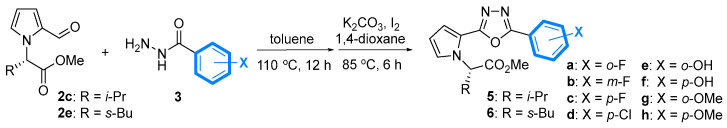
Two-step preparation of various 1,3,4-oxadiazoles **5** and **6** with various electronic substituents X, derived from valine and isoleucine.

The electronic effects of the substituents on the phenyl ring against antibacterial activities were then investigated for 2-pyrrolyl-5-phenyl-1,3,4-oxadiazoles **5** and **6** with the maximum size effect in the series, derived from valine (R = isopropyl) and isoleucine (R = *sec*-butyl), respectively. Commercial benzohydrazides **3** with a substituent X of a different electronic nature (e.g., F, Cl, OH, and OMe) were utilized in the synthesis of **5** and **6** (Scheme 2 and Table 3). The condensation reaction of pyrrole-2-carbaldehyde **2c** (R = *i*-Pr) and **2e** (R = *s*-Bu) with various benzohydrazides **3** produced the corresponding *N*-benzoylhydrazone intermediates in refluxing toluene, which underwent an oxidative cyclization reaction (without purification) to afford 2-pyrrolyl-5-phenyl-1,3,4-oxadiazoles **5** (R = *i*-Pr) and **6** (R = *s*-Bu) with various electronic substituents X on the phenyl ring.

**Figure 2:**
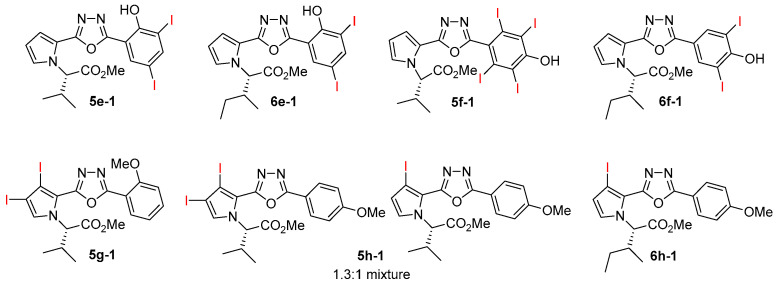
Extra iodination products from 2-pyrrolyl-5-phenyl-1,3,4-oxadiazoles **5** and **6**.

A milder oxidative cyclization condition was required in these cases, for which I_2_/K_2_CO_3_ in 1,4-dioxane at 85 °C was optimal for the production of **5** in yields of 40~85% and **6** in yields of 62~89% in two steps [38]. Serendipitously, we found extra iodination reactions under the oxidative cyclization conditions for 1,3,4-oxadiazoles **5e**, **5f**, **6e**, and **6f** with phenol substituents, obtaining the corresponding di-iodination or tetra-iodination products **5e-1**, **5f-1**, **6e-1**, and **6f-1**, respectively in yields of 32~45% (Figure 2). It was very fortunate to achieve further iodination on the phenol rings so that we were able to find the superior Iodophenol antibacterial effects for these oxadiazole derivatives (vide infra). The corresponding deiodination (originally intended) products were prepared by reduction using Zn dust in AcOH to produce **5f** (X = *p*-OH) in a yield of 97%, **6e** (X = *o*-OH) in a yield of 50%, and **6f** (X = *p*-OH) in a yield of 42%.

To assess the “iodine effect” on antibacterial activity, separate iodination reactions (I_2_ in DMSO at 85 °C) were intentionally carried out for the 1,3,4-oxadiazoles **5g**, **5h**, and **6h** with the electron-rich anisole substituent in which 3-mono-iodination or 3,4-di-iodination reactions proceeded on the pyrrole ring (not on the anisole ring) to provide the corresponding **5g-1** (X = *o*-OMe) in a yield of 61%, **5h-1** (X = *p*-OMe) in a yield of 69% as a 1.3:1 mixture of di- and 3’-mono-iodination products, and **6h-1** (X = *p*-OMe) in a yield of 83%, respectively. All twenty-two pyrrole-ligated oxadiazoles **5** and **6** were rapidly assembled from pyrrole-2-carbaldehydes **2c** (R = *i*-Pr) and **2e** (R = *s*-Bu) with diversely X-substituted benzohydrazide **3** and ready for antibacterial assays against *Escherichia coli, Staphylococcus aureus*, and *Acinetobacter baumannii* together with Vancomycin and Erythromycin as positive controls (Table 4).

SARs of the phenyl substituent X of 2-pyrrolyl-5-phenyl-1,3,4-oxadiazoles **5** and **6** can be deduced from the MIC (μg/mL) in Table 4. *ortho*-F substitutions provided generally better antibacterial activities than the *meta*- and *para*-F counterparts (entries 1–3 and 12–14), and chloride was better than fluoride (entries 4 and 15 versus 3 and 14). Iodophenol substituents exhibited superior antibacterial activities against *A. baumannii* and *S. aureus* regardless of the position of the hydroxyl substituent (entries 5, 6, 16, and 18). The MIC values of <2 μg/mL for **5f-1** and 8 μg/mL for **6f-1** against *A. baumannii* were much lower than those of the positive controls (>1024 μg/mL for vancomycin and 128 μg/mL for erythromycin). The “iodophenol effect” on antibacterial activity is obvious when compared with the cases of deiodination products **5f** and **6f**, the MIC values for which were significantly increased to 128 μg/mL and 512 μg/mL, respectively (entries 7 and 19). The mechanism of the “iodophenol effect” is not clear at present, but it is reasonable to explain that iodide or molecular I_2_ may be liberated by the neighboring OH group [39]. No effects or only slight improvements in antibacterial activities were observed for the pyrrole iodination products **5g-1** and **5h-1** from **5g** and **5h** (entries 8–11), whereas the reverse effect was clear for the pyrrole iodination product **6h-1** from **6h** (entries 21 and 22).

## 3. Materials and Methods

### 3.1. Experimental

#### 3.1.1. General Chemical Syntheses

^1^H- and ^13^C-NMR spectra were recorded on 400 MHz and 100 MHz NMR spectrometers, respectively, in a deuterated solvent (notified in parenthesis) with tetramethylsilane (TMS) as an internal reference. The column chromatography was performed using the method of Still with silica gel 60 and a 70–230 mesh ASTM, using a gradient mixture of EtOAc/hexanes. Reactions were performed in a well-dried flask under an argon atmosphere unless mentioned otherwise.

#### 3.1.2. General Procedure for the Preparation of 1

*Formation of Hydrazone 4 from pyrrole-2-carbaldehyde (Step-1):* The solution of pyrraline **2** (~1.00 g, 1 equiv.) and benzohydrazide **3a** (1 equiv.) in toluene (10 mL) was heated at 110 °C for 6 h. The reaction mixture was cooled to room temperature, diluted with EtOAc, washed with brine and H_2_O, dried over anhydrous Na_2_SO_4_, filtered, and concentrated under reduced pressure. The crude product was purified by SiO_2_ flash column chromatography to obtain the corresponding benzohydrazone **4**.

*2-pyrrolyl-5-phenyl-1,3,4-oxadiazole 1 from hydrazone 4 (Step-2):* The mixture of benzohydrazone **4** (1 equiv.), NaOH (2 equiv.), and *N*-iodosuccinimide (1 equiv.) in DMSO (10 mL) was heated at 110 °C for 2 h. The reaction mixture was cooled to room temperature, diluted with EtOAc, washed with brine and H_2_O, dried over anhydrous Na_2_SO_4_, filtered, and concentrated under reduced pressure. The crude product was purified by SiO_2_ flash column chromatography to obtain the corresponding 2-pyrrolyl-5-phenyl-1,3,4-oxadiazole **1**.

*Methyl 2-(2-(5-phenyl-1,3,4-oxadiazol-2-yl)-1H-pyrrol-1-yl)acetate* (**1a**). Data for **4a**: white solid in a yield of 96% (1.64 g, 5.76 mmol); ^1^H-NMR (DMSO-d_6_) δ = 3.69 (s, 3H), 5.21 (s, 2H), 6.16 (dd, *J* = 3.6, 2.8 Hz, 1H), 6.53 (dd, *J* = 3.6, 1.6 Hz, 1H), 7.02 (dd, *J* = 2.8, 1.6 Hz, 1H), 7.48–7.54 (m, 2H), 7.54–7.60 (m, 1H), 7.86–7.90 (m, 2H), 8.28 (s, 1H), 11.49 (s, 1H) ppm; ^13^C-NMR (DMSO-d_6_) δ = 51.1, 53.1, 109.9, 117.2, 128.4, 128.7, 129.7, 129.9, 132.7, 134.9, 142.0, 163.7, 170.7 ppm; IR (CH_2_Cl_2_) ν = 3237, 3063, 3006, 2954, 2848, 1750, 1649, 1616, 1552, 1494, 1468, 1433, 1345, 1322, 1279, 1216, 1188, 1140, 1086, 1031, 1001, 914, 801, 754, 690 cm^−1^.

Data for **1a**: light-yellow solid in a yield of 80% (0.60 g, 2.13 mmol); ^1^H-NMR (acetone-d_6_) δ = 3.73 (s, 3H), 5.39 (s, 2H), 6.32 (dd, *J* = 4.0, 2.4 Hz, 1H), 7.06 (dd, *J* = 4.0, 1.6 Hz, 1H), 7.18 (dd, *J* = 2.4, 1.6 Hz, 1H), 7.57–7.65 (m, 3H), 8.08–8.14 (m, 2H) ppm; ^13^C-NMR (acetone-d_6_) δ = 50.1, 51.6, 109.2, 114.3, 117.5, 124.0, 126.5, 129.1, 129.2, 131.6, 159.1, 162.4, 168.9 ppm; IR ν = 3119, 3069, 2957, 2927, 2857, 1752, 1713, 1614, 1556, 1501, 1490, 1455, 1424, 1401, 1376, 1328, 1304, 1297, 1264, 1213, 1106, 1081, 995, 956, 787, 773, 760, 725, 693, 583 cm^−1^; HRMS (ESI) calcd for C_15_H_13_N_3_O_3_+Na, 306.0849: found 306.0851.

*Methyl (S)-2-(2-(5-phenyl-1,3,4-oxadiazol-2-yl)-1H-pyrrol-1-yl)propanoate* (**1b**). Data for **4b**: white solid in a yield of 88% (1.45 g, 4.85 mmol); ^1^H-NMR (DMSO-d_6_) δ = 1.70 (d, *J* = 7.2 Hz, 3H), 3.67 (s, 3H), 6.02 (q, *J* = 7.2 Hz, 1H), 6.20 (dd, *J* = 3.6, 2.8 Hz, 1H), 6.54 (dd, *J* = 3.6, 1.6 Hz, 1H), 7.17 (dd, *J* = 2.8, 1.6 Hz, 1H), 7.48–7.61 (m, 3H), 7.86–7.92 (m, 2H), 8.32 (s, 1H), 11.51 (s, 1H) ppm; ^13^C-NMR (DMSO-d_6_) δ = 19.1, 53.4, 56.1, 110.2, 117.5, 126.6, 128.2, 128.7, 129.6, 132.7, 134.9, 142.4, 163.7, 172.8 ppm; IR ν = 3232, 3065, 2954, 1748, 1716, 1644, 1612, 1556, 1494, 1461, 1426, 1359, 1286, 1225, 1146, 1091, 1063, 1031, 962, 910, 887, 857, 802, 714, 695 cm^−1^; HRMS (ESI) calcd for C_16_H_17_N_3_O_3_+Na, 322.1162: found 322.1164.

Data for **1b**: light-yellow solid in a yield of 91% (0.70 g, 2.35 mmol); ^1^H-NMR (CD_3_OD) δ = 1.82 (d, *J* = 7.2 Hz, 3H), 3.71 (s, 3H), 6.00 (q, *J* = 7.2 Hz, 1H), 6.33 (dd, *J* = 4.0, 2.8 Hz, 1H), 7.02 (dd, *J* = 4.0, 1.6 Hz, 1H), 7.23 (dd, *J* = 2.8, 1.6 Hz, 1H), 7.50–7.59 (m, 3H), 8.00–8.04 (m, 2H) ppm; ^13^C-NMR (CD_3_OD) δ = 19.5, 54.5, 58.5, 112.2, 117.6, 119.7, 126.0, 128.1, 129.1, 131.7, 134.4, 162.1, 165.5, 174.7 ppm; IR ν = 3129, 3003, 2955, 2848, 1751, 1663, 1608, 1553, 1505, 1450, 1384, 1336, 1289, 1224, 1203, 1110, 1091, 1062, 1015, 981, 962, 853, 813, 776, 729, 691, 610 cm^−1^; HRMS (ESI) calcd for C_16_H_15_N_3_O_3_+Na, 320.1006: found 320.1007.

*Methyl (S)-3-methyl-2-(2-(5-phenyl-1,3,4-oxadiazol-2-yl)-1H-pyrrol-1-yl)butanoate* (**1c**). Data for **4c**: white solid in a yield of 83% (1.30 g, 3.98 mmol); ^1^H-NMR (DMSO-d_6_) δ = 1.03 (d, *J* = 6.8 Hz, 3H), 1.35 (d, *J* = 6.8 Hz, 3H), 2.76 (m, 1H), 4.04 (s, 3H), 6.35 (d, *J* = 8.8 Hz, 1H), 6.57 (dd, *J* = 3.6, 2.8 Hz, 1H), 6.87 (dd, *J* = 3.6, 1.6 Hz, 1H), 7.51 (dd, *J* = 2.8, 1.6 Hz, 1H), 7.82–7.97 (m, 3H), 8.21–8.30 (m, 2H), 8.73 (s, 1H), 11.90 (s, 1H) ppm; ^13^C-NMR (DMSO-d_6_) δ = 17.6, 18.4, 31.3, 51.4, 62.8, 108.8, 114.6, 124.7, 126.6, 126.7, 127.6, 130.7, 132.8, 140.3, 161.7, 169.9 ppm; IR ν = 3241, 3071, 2975, 2878, 1750, 1644, 1615, 1557, 1495, 1459, 1433, 1392, 1356, 1286, 1211, 1160, 1133, 1083, 1057, 1023, 1005, 952, 915, 890, 835, 800, 755, 715, 695, 617 cm^−1^.

Data for **1c**: light-yellow solid in a yield of 98% (0.15 g, 0.46 mmol); ^1^H-NMR (CD_3_OD) δ = 0.81 (d, *J* = 7.2 Hz, 3H), 1.08 (d, *J* = 7.2 Hz, 3H), 2.53 (m, 1H), 3.76 (s, 3H), 5.99 (d, *J* = 10.0 Hz, 1H), 6.38 (dd, *J* = 4.0, 2.8 Hz, 1H), 7.03 (dd, *J* = 4.0, 1.6 Hz, 1H), 7.36 (dd, *J* = 2.8, 1.6 Hz, 1H), 7.54–7.64 (m, 3H), 8.06–8.12 (m, 2H) ppm; ^13^C-NMR (CD_3_OD) δ = 20.4, 21.1, 35.2, 54.1, 67.1, 112.3, 119.0, 128.5, 130.0, 130.5, 131.1, 134.4, 136.0, 145.0, 167.8, 174.2 ppm; IR ν = 2958, 2927, 2851, 1744, 1669, 1609, 1560, 1500, 1454, 1376, 1260, 1221, 1105, 1076, 1013, 775, 727, 691 cm^−1^; HRMS (ESI) calcd for C_18_H_19_N_3_O_3_+Na, 348.1319: found 348.1319.

*Methyl (S)-4-methyl-2-(2-(5-phenyl-1,3,4-oxadiazol-2-yl)-1H-pyrrol-1-yl)pentanoate* (**1d**). Data for **4d**: white solid in a yield of 77% (1.18 g, 3.46 mmol); ^1^H-NMR (DMSO-d_6_) δ = 0.86 (d, *J* = 6.4 Hz, 3H), 0.92 (d, *J* = 6.4 Hz, 3H), 1.92 (dd of A of ABq, *J_AB_* = 14.0, *J_d_* = 9.2, 4.8 Hz, 1H), 2.12 (dd of B of ABq, *J_AB_* = 14.0, *J_d_* = 11.6, 4.4 Hz, 1H), 3.67 (s, 3H), 6.21 (dd, *J* = 3.6, 2.8 Hz, 1H), 6.30 (dd, *J* = 9.2, 4.4 Hz, 1H), 6.52 (dd, *J* = 3.6, 1.6 Hz, 1H), 7.19 (dd, *J* = 2.8, 1.6 Hz, 1H), 7.48–7.62 (m, 3H), 7.86–7.92 (m, 2H), 8.34 (s, 1H), 11.52 (s, 1H) ppm; ^13^C-NMR (DMSO-d_6_) δ = 22.6, 24.1, 25.7, 42.1, 53.5, 58.5, 110.6, 117.6, 127.2, 128.4, 128.7, 129.6, 132.7, 134.8, 142.5, 163.6, 172.8 ppm; IR ν = 3234, 3065, 2956, 2874, 1743, 1646, 1614, 1556, 1495, 1459, 1427, 1348, 1278, 1240, 1203, 1174, 1086, 1034, 998, 953, 928, 903, 795, 754 cm^−1^.

Data for **1d**: light-yellow solid in 87% yield (1.35g, 4.01 mmol); ^1^H-NMR (acetone-d_6_) δ = 0.94 (d, *J* = 6.4 Hz, 3H), 0.95 (d, *J* = 6.4 Hz, 3H), 1.49 (m, 1H), 2.02–2.16 (m, 1H), 2.16–2.30 (m, 1H), 3.71 (s, 3H), 6.38 (dd, *J* = 3.6, 2.8 Hz, 1H), 7.06 (dd, *J* = 3.6, 1.6 Hz, 1H), 7.34 (dd, *J* = 2.8, 1.6 Hz, 1H), 7.56–7.65 (m, 3H), 8.08–8.16 (m, 2H) ppm; ^13^C-NMR (acetone-d_6_) δ = 20.9, 22.3, 24.7, 41.0, 51.9, 58.0, 109.8, 114.4, 117.6, 123.9, 126.1, 126.6, 129.2, 131.6, 159.3, 162.3, 171.2 ppm; IR ν = 3119, 3069, 2957, 2867, 1750, 1704, 1664, 1609, 1557, 1504, 1454, 1412, 1369, 1333, 1273, 1240, 1197, 1176, 1133, 1106, 1080, 1021, 1000, 964, 925, 880, 836, 811, 730, 690, 613 cm^−1^; HRMS (ESI) calcd for C_19_H_21_N_3_O_3_+Na, 362.1475: found 362.1474.

*Methyl (2S,3S)-3-methyl-2-(2-(5-phenyl-1,3,4-oxadiazol-2-yl)-1H-pyrrol-1-yl)pentanoate* (**1e**). Data for **4e**: white solid in a yield of 86% (0.91 g, 2.67 mmol); ^1^H-NMR (DMSO-d_6_) δ = 0.79 (t, J = 7.2 Hz, 3H), 0.96 (d, *J* = 6.4 Hz, 3H), 1.01–1.20 (m 2H), 2.15–2.26 (m 2H), 3.69 (s, 3H), 6.03 (d, J = 6.8 Hz, 1H), 6.22 (dd, *J* = 3.6, 2.8 Hz, 1H), 6.52 (dd, *J* = 3.6, 1.6 Hz, 1H), 7.18 (dd, *J* = 2.8, 1.6 Hz, 1H), 7.49–7.62 (m, 3H), 7.88–7.95 (m, 2H), 8.39 (s, 1H), 11.55 (s, 1H) ppm; ^13^C-NMR (DMSO-d_6_) δ = 10.7, 15.5, 24.3, 38.1, 52.2, 62.8, 109.7, 115.5, 125.4, 127.5, 127.6, 128.4, 131.6, 133.6, 141.1, 162.5, 170.9 ppm.

Data for **1e**: light-yellow solid in 90% yield (0.42 g, 1.23 mmol, a 2:1 mixture of stereoisomers); ^1^H-NMR (major isomer, CDCl_3_) δ = 0.84 (t, *J* = 7.2 Hz, 3H), 1.05 (d, *J* = 6.4 Hz, 3H), 1.06–1.28 (m, 2H), 2.27–2.40 (m, 1H), 3.75 (s, 3H), 6.18 (d, *J* = 10.4 Hz, 1H), 6.39 (dd, *J* = 4.0, 2.8 Hz, 1H), 7.04 (dd, *J* = 4.0, 2.0 Hz, 1H), 7.40 (dd, *J* = 2.8, 2.0 Hz, 1H), 7.58–7.66 (m, 3H), 8.10–8.16 (m, 2H) ppm; ^13^C-NMR (major isomer, CDCl_3_) δ = 10.1, 15.0, 24.7, 38.8, 51.7, 63.7, 110.1, 114.0, 114.1, 123.9, 126.0, 126.6, 129.2, 131.6, 159.3, 162.4, 170.7 ppm; IR ν = 3144, 3124, 3066, 2970, 2932, 2879, 1747, 1704, 1606, 1552, 1501, 1492, 1449, 1414, 1387, 1334, 1284, 1236, 1196, 1178, 1100, 1077, 1020, 964, 926, 883, 727, 694, 618 cm^−1^; HRMS (ESI) calcd for C_19_H_21_N_3_O_3_+Na, 362.1475: found 362.1475.

*Methyl (S)-3-phenyl-2-(2-(5-phenyl-1,3,4-oxadiazol-2-yl)-1H-pyrrol-1-yl)propanoate* (**1f**). Data for **4f**: white solid in a yield of 83% (0.94 g, 2.51 mmol); ^1^H-NMR (DMSO-d_6_) δ = 3.42 (d of A of ABq, *J_AB_* = 14.4, *J_d_* = 10.0 Hz, 1H), 3.49 (d of B of ABq, *J_AB_* = 14.4, *J_d_* = 6.4 Hz, 1H), 3.67 (s, 3H), 6.11 (dd, *J* = 3.6, 2.8 Hz, 1H), 6.43 (dd, *J* = 3.6, 1.6 Hz, 1H), 6.49 (dd, *J* = 10.0, 6.4 Hz, 1H), 7.10–7.25 (m, 6H), 7.50–7.62 (m, 3H), 7.88–7.93 (m, 2H), 8.23 (s, 1H), 11.50 (s, 1H) ppm; ^13^C-NMR (DMSO-d_6_) δ = 39.1, 53.6, 61.1, 110.4, 117.6, 127.5, 127.7, 128.2, 128.7, 129.3, 129.7, 130.4, 132.8, 134.9, 137.9, 142.2, 163.8, 171.7 ppm; IR ν = 3236, 3064, 3033, 2957, 2843, 1743, 1645, 1613, 1555, 1497, 1455, 1436, 1348, 1280, 1220, 1185, 1164, 1076, 1032, 1008, 904, 843, 802, 753, 699 cm^−1^.

Data for **1f**: light-yellow solid in a yield of 86% (2.31 g, 6.19 mmol); ^1^H-NMR (CD_3_OD) δ = 3.38 (d of A of ABq, *J_AB_* = 14.0, *J_d_* = 10.0 Hz, 1H), 3.59 (d of B of ABq, *J_AB_* = 14.0, *J_d_* = 5.2 Hz, 1H), 3.75 (s, 3H), 6.29 (dd, *J* = 4.0, 2.8 Hz, 1H), 6.39 (dd, *J* = 10.0, 5.2 Hz, 1H), 6.90 (dd, *J* = 4.0, 1.6 Hz, 1H), 7.02–7.14 (m, 5H), 7.24 (dd, *J* = 2.8, 1.6 Hz, 1H), 7.54–7.62 (m, 3H), 8.01–8.05 (m, 2H) ppm; ^13^C-NMR (CD_3_OD) δ = 41.4, 54.6, 63.9, 112.5, 117.3, 119.8, 126.0, 129.2, 129.2, 129.4, 130.7, 131.5, 131.8, 134.5, 139.0, 162.1, 165.5, 173.4 ppm; IR ν = 3068, 3033, 3004, 2956, 2848, 1747, 1703, 1665, 1606, 1559, 1505, 1449, 1412, 1372, 1337, 1274, 1230, 1201, 1174, 1107, 1083, 1012, 982, 926, 884, 778, 728, 699, 615 cm^−1^; HRMS (ESI) calcd for C_22_H_19_N_3_O_3_+Na 396.1319, found 396.1321.

*Methyl (S)-4-phenyl-2-(2-(5-phenyl-1,3,4-oxadiazol-2-yl)-1H-pyrrol-1-yl)butanoate* (**1g**). Data for **4g**: white solid in a yield of 79% (1.31 g, 3.48 mmol); ^1^H-NMR (DMSO-d_6_) δ = 2.36–2.60 (m, 4H), 3.68 (s, 3H), 6.00–6.07 (m, 1H), 6.26 (dd, *J* = 3.6, 2.8 Hz, 1H), 6.58 (dd, *J* = 3.6, 1.2 Hz, 1H), 7.11–7.28 (m, 6H), 7.50–7.61 (m, 3H), 7.87–7.92 (m, 2H), 8.34 (s, 1H), 11.52 (s, 1H) ppm; ^13^C-NMR (DMSO-d_6_) δ = 32.8, 35.1, 53.5, 60.1, 110.6, 117.2, 127.2, 128.5, 128.7, 129.5, 129.6, 129.6, 129.6, 132.7, 134.8, 141.8, 142.3, 163.7, 172.1 ppm; IR ν = 3227, 3063, 3030, 2951, 2866, 1744, 1646, 1610, 1558, 1494, 1457, 1431, 1325, 1287, 1217, 1164, 1079, 1055, 1037, 1029, 1004, 952, 913, 755, 698 cm^−1^.

Data for **1g**: light-yellow solid in 86% yield (0.42 g, 1.13 mmol); ^1^H-NMR (CD_3_OD) δ = 2.40–2.67 (m, 4H), 3.71 (s, 3H), 5.92 (dd, *J* = 10.4, 4.0 Hz, 1H), 6.43 (dd, *J* = 4.0, 2.8 Hz, 1H), 6.99–7.05 (m, 3H), 7.06 (dd, *J* = 4.0, 1.6 Hz, 1H), 7.08–7.16 (m, 2H), 7.31 (dd, *J* = 2.8, 1.6 Hz, 1H), 7.53–7.62 (m, 3H), 8.00–8.06 (m, 2H) ppm; ^13^C-NMR (CD_3_OD) δ = 31.4, 33.3, 51.7, 58.9, 110.0, 114.5, 117.3, 123.2, 125.8, 126.0, 126.4, 128.0, 128.0, 129.0, 131.7, 139.8, 159.2, 162.7, 171.3 ppm; IR ν = 3064, 3026, 2956, 2931, 2854, 1745, 1662, 1606, 1557, 1504, 1450, 1415, 1254, 1233, 1198, 1177, 1082, 1012, 979, 814, 772, 725, 698, 605 cm^−1^; HRMS (ESI) calcd for C_23_H_21_N_3_O_3_+Na, 410.1475: found 410.1477.

*Dimethyl (S)-2-(2-(5-phenyl-1,3,4-oxadiazol-2-yl)-1H-pyrrol-1-yl)succinate* (**1h**). Data for **4h**: white solid in a yield of 81% (1.01g, 2.84 mmol); ^1^H-NMR (CD_3_OD) δ = 3.27 (d of A of ABq, *J_AB_* = 16.8, *J_d_* = 8.0 Hz, 1H), 3.37 (d of B of ABq, *J_AB_* = 16.8, *J_d_* = 6.0 Hz, 1H), 3.63 (s, 3H), 3.73 (s, 3H), 6.20 (dd, *J* = 3.6, 2.8 Hz, 1H), 6.32 (dd, *J* = 8.0, 6.0 Hz, 1H), 6.58 (dd, *J* = 3.6, 1.6 Hz, 1H), 7.02 (dd, *J* = 2.8, 1.6 Hz, 1H), 7.46–7.60 (m, 3H), 7.86–7.91 (m, 2H), 8.24 (s, 1H) ppm; ^13^C-NMR (CD_3_OD) δ = 39.4, 53.8, 54.6, 59.5, 112.0, 120.3, 129.4, 129.9, 130.0, 131.1, 134.4, 136.0, 144.6, 167.8, 173.2, 173.8 ppm; IR ν = 3411, 2958, 1737, 1644, 1613, 1581, 1554, 1495, 1444, 1414, 1348, 1285, 1238, 1177, 1123, 1084, 1008, 979, 908, 803, 786, 712 cm^−1^.

Data for **1h**: light-yellow solid in 88% yield (0.79 g, 2.22 mmol); ^1^H-NMR (CD_3_OD) δ = 3.25 (d of A of ABq, *J_AB_* = 16.8, *J_d_* = 8.0 Hz, 1H), 3.41 (d of B of ABq, *J_AB_* = 16.8, *J_d_* = 6.0 Hz, 1H), 3.63 (s, 3H), 3.72 (s, 3H), 6.32 (dd, *J* = 3.6, 2.8 Hz, 1H), 6.39 (dd, *J* = 8.0, 6.0 Hz, 1H), 7.03 (dd, *J* = 3.6, 1.6 Hz, 1H), 7.17 (dd, *J* = 2.8, 1.6 Hz, 1H), 7.50–7.60 (m, 3H), 8.00–8.07 (m, 2H) ppm; ^13^C-NMR (CD_3_OD) δ = 36.4, 51.2, 52.0, 56.8, 109.8, 115.0, 117.0, 123.2, 126.4, 126.7, 129.0, 131.7, 159.1, 162.8, 169.9, 170.5 ppm; IR ν = 3123, 3006, 2958, 2854, 1741, 1662, 1605, 1553, 1505, 1485, 1439, 1417, 1371, 1271, 1224, 1173, 1083, 1010, 985, 860, 819, 781, 726, 692, 672, 611 cm^−1^; HRMS (ESI) calcd for C_18_H_17_N_3_O_5_+Na, 378.1060: found 378.1062.

*Dimethyl (S)-2-(2-(5-phenyl-1,3,4-oxadiazol-2-yl)-1H-pyrrol-1-yl)pentanedioate* (**1i**). Data for **4i**: white solid in a yield of 70% (1.52 g, 4.08 mmol), ^1^H-NMR (DMSO-d_6_) δ = 2.10–2.21 (m, 1H), 2.24–2.42 (m, 2H), 2.44–2.53 (m, 1H), 3.55 (s, 3H), 3.70 (s, 3H), 6.01–6.09 (m, 1H), 6.22 (dd, *J* = 3.6, 2.8 Hz, 1H), 6.55 (dd, *J* = 3.6, 1.6 Hz, 1H), 7.12 (dd, *J* = 2.8, 1.6 Hz, 1H), 7.49–7.61 (m, 3H), 7.87–7.92 (m, 2H), 8.32 (s, 1H) ppm; ^13^C-NMR (DMSO-d_6_) δ = 28.7, 31.3, 52.7, 53.6, 59.7, 110.7, 117.5, 127.6, 128.5, 128.7, 129.7, 132.8, 134.8, 142.2, 163.7, 171.7, 173.5 ppm; IR ν = 3234, 3020, 2954, 1738, 1650, 1615, 1558, 1458, 1437, 1346, 1280, 1218, 1177, 1075, 1029, 913, 888, 801, 753 cm^−1^.

Data for **1i**: light-yellow solid in a yield of 95% (1.04 g, 2.82 mmol); ^1^H-NMR (acetone-d6) δ = 2.24–2.39 (m, 2H), 2.44–2.54 (m, 1H), 2.63–2.73 (m, 1H), 3.57 (s, 3H), 3.73 (s, 3H), 6.24 (dd, *J* = 10.8, 5.2 Hz, 1H), 6.39 (dd, *J* = 3.6, 2.8 Hz, 1H), 7.06 (dd, *J* = 3.6, 1.6 Hz, 1H), 7.30 (dd, *J* = 2.8, 1.6 Hz, 1H), 7.58–7.65 (m, 3H), 8.10–8.16 (m, 2H) ppm; ^13^C-NMR (acetone-d6) δ = 29.4, 31.4, 52.6, 53.7, 60.7, 111.8, 116.2, 119.4, 125.6, 128.0, 128.3, 130.9, 133.3, 160.9, 164.1, 172.0, 173.7 ppm; IR ν = 3120, 3005, 2956, 2923, 2853, 1741, 1664, 1607, 1552, 1504, 1490, 1450, 1371, 1240, 1202, 1174, 1106, 1078, 1012, 989, 964, 930, 882, 847, 821, 727, 694, 612 cm^−1^; HRMS (ESI) calcd for C_19_H_19_N_3_O_5_+Na, 392.1217: found 392.1220.

Methyl (S)-4-(methylthio)-2-(2-(5-phenyl-1,3,4-oxadiazol-2-yl)-1H-pyrrol-1-yl)butanoate (**1j**) and methyl (S)-6-(5-phenyl-1,3,4-oxadiazol-2-yl)-3,4-dihydro-2H-pyrrolo[2,1-b][1,3]thiazine-4-carboxylate (**1k**). Data for **4j**: white solid in a yield of 82% (0.77 g, 2.14 mmol); ^1^H-NMR (DMSO-d_6_) δ = 2.02 (s, 3H), 2.24–2.46 (m, 4H), 3.69 (s, 3H), 5.97–6.05 (m, 1H), 6.22 (dd, *J* = 3.6, 2.8 Hz, 1H), 6.55 (dd, *J* = 3.6, 1.2 Hz, 1H), 7.15 (dd, *J* = 2.8, 1.2 Hz, 1H), 7.48–7.61 (m, 3H), 7.86–8.02 (m, 2H), 8.33 (s, 1H) ppm; ^13^C-NMR (DMSO-d_6_) δ = 15.8, 30.8, 32.9, 53.6, 59.7, 110.6, 117.3, 127.4, 128.5, 128.7, 129.7, 132.8, 134.8, 142.1, 163.7, 171.8 ppm; IR ν = 3230, 3006, 2957, 12918, 2849, 1743, 1649, 1614, 1556, 1491, 1459, 1431, 1350, 1288, 1230, 1209, 1144, 1091, 1033, 1001, 955, 909, 888, 798, 756, 613 cm^−1^.

Data for **1j**: light-yellow solid in a yield of 50% (0.31 g, 0.86 mmol); ^1^H-NMR (CD_3_OD) δ = 2.03 (s, 3H), 2.28–2.36 (m, 1H), 2.43–2.63 (m, 3H), 3.74 (s, 3H), 6.18 (dd, *J* = 10.0, 4.8 Hz, 1H), 6.39 (dd, *J* = 4.0, 2.8 Hz, 1H), 7.07 (dd, *J* = 4.0, 2.0 Hz, 1H), 7.25 (dd, *J* = 2.8, 2.0 Hz, 1H), 7.55–7.63 (m, 3H), 8.06–8.10 (m, 2H) ppm; ^13^C-NMR (CD_3_OD) δ = 13.7, 29.6, 31.1, 51.8, 58.7, 109.8, 114.8, 117.2, 123.2, 126.4, 129.0, 131.7, 159.2, 162.8, 171.0, 179.6 ppm; IR ν = 2956, 2925, 2857, 1750, 1667, 1603, 1554, 1505, 1452, 1381, 1274, 1243, 1090, 1016, 961, 883, 817, 774, 732, 691 cm^−1^; HRMS (ESI) calcd for C_18_H_19_N_3_O_3_S+Na, 380.1039: found 380.1040.

Data for **1k**: light-yellow solid in 49% yield (0.31g, 0.72 mmol); ^1^H-NMR (CD_3_OD) δ = 2.39–2.49 (m, 1H), 2.86–3.02 (m, 3H), 3.73 (s, 3H), 5.94 (dd, *J* = 5.2, 3.2 Hz, 1H), 6.06 (d, *J* = 4.0 Hz, 1H), 7.01 (d, *J* = 4.0 Hz, 1H), 7.49–7.57 (m, 3H), 7.97–8.01 (m, 2H) ppm; ^13^C-NMR (CD_3_OD) δ = 22.0, 28.0, 53.3, 58.2, 108.5, 116.1, 119.0, 124.7, 127.7, 128.7, 130.3, 132.9, 160.2, 163.6, 172.5 ppm; IR ν = 3008, 2948, 2852, 1751, 1648, 1604, 1552, 1497, 1439, 1401, 1355, 1292, 1257, 1214, 1176, 1145, 1125, 1085, 1070, 1023, 974, 929, 898, 854, 758, 727, 696 cm^−1^; HRMS (ESI) calcd for C_17_H_15_N_3_O_3_S+Na, 364.0726: found 364.0729.

#### 3.1.3. General Procedure for the Preparation of **5** (R = i-Pr) and **6** (R = s-Bu)

Step-1: At 25 °C, under an argon atmosphere, benzohydrazide **3** (1.1 equiv.) was added to a stirred solution of pyrrole-2-carbaldehyde **2** (~1.0 g, 1 equiv.) in toluene/DMSO (*v*:*v* = 15:1). The mixture was heated at 110 °C for 12 h and cooled to room temperature. The solvent was removed under reduced pressure in a rotary evaporator, and the crude product was filtered through a short pad of SiO_2_ (EtOAc eluent) and concentrated under reduced pressure.

Step-2: I_2_ (1.2 equiv.) and K_2_CO_3_ (3 equiv.) were added to a stirred solution of the above imine in 1,4-dioxane (20 mL). The mixture was heated at 85 °C for 6h under an argon atmosphere and cooled to room temperature. The mixture was diluted with CH_2_Cl_2_, washed with a 10% Na_2_S_2_O_3_ solution, dried over anhydrous Na_2_SO_4_, filtered, and concentrated under reduced pressure. The product was purified by SiO_2_ flash column chromatography to obtain pyrrole-fused 1,3,4-oxadiazole **5** or **6**.

*Methyl (S)-2-(2-(5-(2-fluorophenyl)-1,3,4-oxadiazol-2-yl)-1H-pyrrol-1-yl)-3-methylbutanoate* (**5a**). Orange oil, 40% yield (663 mg, 1.93 mmol); R_f_ = 0.45 (4:1 hexane/EtOAc); Data for **5a**: ^1^H-NMR (CDCl_3_) δ = 0.83 (d, *J* = 6.4 Hz, 3H), 1.08 (d, *J* = 6.4 Hz, 3H), 2.48 (m, 1H), 3.75 (s, 3H), 6.15 (d, *J* = 10.0 Hz, 1H), 6.36 (dd, *J* = 4.0, 3.2 Hz, 1H), 6.98 (dd, *J* = 4.0, 2.0 Hz, 1H), 7.23–7.28 (m, 1H), 7.28–7.34 (m, 1H), 7.31 (dd, *J* = 3.2, 2.0 Hz, 1H), 7.50–7.57 (m, 1H), 8.08–8.13 (m, 1H) ppm; ^13^C-NMR (CDCl_3_) δ = 18.5, 19.3, 33.2, 52.3, 64.6, 110.4, 114.5, 117.0 (d, *J* = 20.5 Hz), 117.8, 124.6 (d, *J* = 3.1 Hz), 126.0, 129.6 (d, *J* = 1.5 Hz), 133.3 (d, *J* = 8.4 Hz), 158.8, 159.4, (d, *J* = 5.4 Hz), 159.7 (d, *J* = 1.5 Hz), 161.3, 171.3 ppm; IR ν = 2970, 2880, 1748, 1605, 1500, 1475, 1450, 1273, 1239, 1219, 1200, 1185, 1170, 1102, 1078, 1009, 827, 742, 669, 617 cm^−1^; HRMS (ESI) calcd for C_18_H_18_FN_3_O_3_+Na 366.1224: found 366.1225.

*Methyl (S)-2-(2-(5-(3-fluorophenyl)-1,3,4-oxadiazol-2-yl)-1H-pyrrol-1-yl)-3-methylbutanoate* (**5b**). Orange oil, 70% yield (759 mg, 2.21 mmol); R_f_ = 0.59 (4:1 hexane/EtOAc); Data for **5b**: ^1^H-NMR (CDCl_3_) δ = 0.83 (d, *J* = 6.4 Hz, 3H), 1.08 (d, *J* = 6.4 Hz, 3H), 2.49 (m, 1H), 3.75 (s, 3H), 6.14 (d, *J* = 10.0 Hz, 1H), 6.37 (dd, *J* = 4.0, 2.8 Hz, 1H), 6.97 (dd, *J* = 4.0, 2.0 Hz, 1H), 7.20–7.27 (m, 1H), 7.32 (dd, *J* = 2.8, 2.0 Hz, 1H), 7.47–7.54 (m, 1H), 7.76–7.81 (m, 1H), 7.87–7.91 (m, 1H) ppm; ^13^C-NMR (CDCl_3_) δ = 18.5, 19.3, 33.1, 52.3, 64.6, 110.4, 113.8 (d, *J* = 24.2 Hz), 114.4 117.7, 118.6 (d, *J* = 21.2 Hz), 122.5 (d, *J* = 3.0 Hz), 125.7 (d, *J* = 9.1 Hz), 126.1, 130.9 (d, *J* = 8.3 Hz), 159.7, 161.6, (d, *J* = 2.3 Hz), 164.0, 171.2 ppm; IR ν = 2970, 2880, 1750, 1600, 1565, 1500, 1495, 1450, 1410, 1375, 1307, 1275, 1240, 1205, 1181, 1105, 1080, 1010, 995, 870, 800, 735, 680, 615 cm^−1^; HRMS (ESI) calcd for C_18_H_18_FN_3_O_3_+Na 366.1224: found 366.1226.

*Methyl (S)-2-(2-(5-(4-fluorophenyl)-1,3,4-oxadiazol-2-yl)-1H-pyrrol-1-yl)-3-methylbutanoate* (**5c**). Yellow oil, 54% yield (721 mg, 2.10 mmol); R_f_ = 0.65 (4:1 hexane/EtOAc); Data for **5c**: ^1^H-NMR (CDCl_3_) δ = 0.83 (d, *J* = 6.4 Hz, 3H), 1.08 (d, *J* = 6.4 Hz, 3H), 2.48 (m, 1H), 3.75 (s, 3H), 6.14 (d, *J* = 10.0 Hz, 1H), 6.36 (dd, *J* = 4.0, 2.8 Hz, 1H), 6.94 (dd, *J* = 4.0, 2.0 Hz, 1H), 7.18–7.25 (m, 2H), 7.31 (dd, *J* = 2.8, 2.0 Hz, 1H), 8.07–8.13 (m, 2H) ppm; ^13^C-NMR (CDCl_3_) δ = 18.5, 19.3, 33.1, 52.3, 64.6, 110.3, 114.1, 116.4 (d, *J* = 22.0 Hz), 120.2 (d, *J* = 3.0 Hz), 125.9, 129.0 (d, *J* = 9.1 Hz), 159.4, 161.8, 163.4, 165.9, 171.2 ppm; IR ν = 2970, 2880, 1750, 1605, 1500, 1470, 1455, 1435, 1415, 1270, 1235, 1215, 1200, 1160, 1100, 1075, 1015, 965, 845, 740, 625 cm^−1^; HRMS (ESI) calcd for C_18_H_18_FN_3_O_3_+Na 366.1224, found 366.1228.

*Methyl (S)-2-(2-(5-(4-chlorophenyl)-1,3,4-oxadiazol-2-yl)-1H-pyrrol-1-yl)-3-methylbutanoate* (**5d**). Brown solid, 62% yield (533 mg, 1.48 mmol); R_f_ = 0.61 (4:1 hexane/EtOAc); Data for **5d**: ^1^H-NMR (CDCl_3_) δ = 0.82 (d, *J* = 6.4 Hz, 3H), 1.08 (d, *J* = 6.4 Hz, 3H), 2.48 (m, 1H), 3.75 (s, 3H), 6.14 (d, *J* = 10.0 Hz, 1H), 6.36 (dd, *J* = 4.0, 2.8 Hz, 1H), 6.95 (dd, *J* = 4.0, 2.0 Hz, 1H), 7.31 (dd, *J* = 2.8, 2.0 Hz, 1H), 7.48–7.53 (m, 2H), 8.01–8.06 (m, 2H) ppm; ^13^C-NMR (CDCl_3_) δ = 18.5, 19.3, 33.2, 52.3, 64.6, 110.3, 114.3, 117.8, 122.3, 126.0, 128.1, 129.5, 137.8, 159.6, 161.8, 171.2 ppm; IR ν = 2970, 2880, 1750, 1605, 1500, 1485, 1455, 1410, 1270, 1237, 1200, 1180, 1100, 1080, 1015, 970, 840, 740 cm^−1^; HRMS (ESI) calcd for C_18_H_18_ClN_3_O_3_+Na 382.0929, found 382.0933.

*Methyl (S)-2-(2-(5-(2-hydroxy-3,5-diiodophenyl)-1,3,4-oxadiazol-2-yl)-1H-pyrrol-1-yl)-3-methylbutanoate* (**5e-1**). White solid, 33% yield (748 mg, 1.26 mmol); R_f_ = 0.54 (4:1 hexane/EtOAc); Data for **5e-1**: ^1^H-NMR (CDCl_3_) δ = 0.82 (d, *J* = 6.4 Hz, 3H), 1.09 (d, *J* = 6.4 Hz, 3H), 2.49 (m, 1H), 3.77 (s, 3H), 5.99 (d, *J* = 9.6 Hz, 1H), 6.40 (dd, *J* = 4.0, 2.8 Hz, 1H), 7.05 (dd, *J* = 4.0, 2.0 Hz, 1H), 7.36 (dd, *J* = 2.8, 2.0 Hz, 1H), 8.06 (d, *J* = 2.0 Hz, 1H), 8.18 (d, *J* = 2.0 Hz, 1H) 11.02 (s, 1H) ppm; ^13^C-NMR (CDCl_3_) δ = 18.6, 19.3, 33.2, 52.4, 64.9, 81.3, 86.7, 109.8, 110.8, 115.7, 116.9, 127.0, 134.7, 149.8, 156.2, 158.7, 160.1, 171.0 ppm; IR ν = 2970, 2890, 1750, 1600, 1565, 1535, 1500, 1445, 1415, 1380, 1255, 1240, 1220, 1185, 1105, 1080, 1015, 1000, 915, 870, 740, 660, 615, 600 cm^−1^; HRMS (ESI) calcd for C_18_H_17_I_2_N_3_O_4_+Na 615.9201: found 615.9203.

*Methyl (S)-2-(2-(5-(4-hydroxy-2,3,5,6-tetraiodophenyl)-1,3,4-oxadiazol-2-yl)-1H-pyrrol-1-yl)-3-methylbutanoate* (**5f-1**): Yellow solid, 32% yield (1.33 g, 1.57 mmol); R_f_ = 0.55 (4:1 hexane/EtOAc); Data for **5f-1**. ^1^H-NMR (CDCl_3_) δ = 0.81 (d, *J* = 6.4 Hz, 3H), 1.07 (d, *J* = 6.4 Hz, 3H), 2.41–2.53 (m, 1H), 3.75 (s, 3H), 6.09 (d, *J* = 10.0 Hz, 1H), 6.36 (dd, *J* = 4.0, 2.8 Hz, 1H), 6.96 (dd, *J* = 4.0, 1.6 Hz, 1H), 7.31 (dd, *J* = 2.8, 1.6 Hz, 1H), 8.40 (s, 1H) ppm; ^13^C-NMR (CDCl_3_) δ = 18.5, 19.3, 33.1, 52.3, 64.6, 82.4, 110.4, 114.5, 117.7, 119.9, 126.1, 137.6, 156.2, 159.5, 159.6, 171.2 ppm; IR ν = 3450, 3145, 3125, 2970, 2875, 1745, 1610, 1595, 1500, 1450, 1395, 1300, 1270, 1235, 1220, 1200, 1180, 1160, 1135, 1105, 1075, 1010, 995, 965, 910, 890, 810, 735, 710, 685, 650, 615 cm^−1^; HRMS (ESI) calcd for [C_18_H_15_I_4_N_3_O_4_ –I_2_+H_2_]+Na 615.9201: found 615.9213.

*Methyl (S)-2-(2-(5-(2-methoxyphenyl)-1,3,4-oxadiazol-2-yl)-1H-pyrrol-1-yl)-3-methylbutanoate* (**5g**). Red oil, 85% yield (1.03 g, 2.89 mmol); R_f_ = 0.66 (3:2 hexane/EtOAc); Data for **5g**: ^1^H-NMR (CDCl_3_) δ = 0.83 (d, *J* = 6.4 Hz, 3H), 1.07 (d, *J* = 6.4 Hz, 3H), 2.48 (m, 1H), 3.74 (s, 3H), 4.00 (s, 3H), 6.19 (d, *J* = 10.0 Hz, 1H), 6.35 (dd, *J* = 4.0, 2.8 Hz, 1H), 6.94 (dd, *J* = 4.0, 2.0 Hz, 1H), 7.06–7.12 (m, 2H), 7.29 (dd, *J* = 2.8, 2.0 Hz, 1H), 7.48–7.53 (m, 1H), 7.99 (dd, *J* = 7.6, 2.0 Hz, 1H) ppm; ^13^C-NMR (CDCl_3_) δ = 18.5, 19.3, 33.2, 52.2, 56.0, 64.5, 110.1, 112.0, 112.9, 113.9, 118.2, 120.7, 125.5, 130.2, 132.8, 158.0, 159.0, 161.3, 171.4 ppm; IR ν = 2970, 2875, 2840, 1750, 1600, 1545, 1500, 1470, 1455, 1440, 1270, 1260, 1235, 1215, 1185, 1170, 1130, 1100, 1075, 1025, 750, 735, 675, 616 cm^−1^; HRMS (ESI) calcd for C_19_H_21_N_3_O_4_+Na 378.1424, found 378.1428.

*Methyl (S)-2-(2-(5-(4-methoxyphenyl)-1,3,4-oxadiazol-2-yl)-1H-pyrrol-1-yl)-3-methylbutanoate* (**5h**). Ivory solid, 70% yield (1.12 g, 3.15 mmol); R_f_ = 0.70 (3:2 hexane/EtOAc); Data for **5h**: ^1^H-NMR (CDCl_3_) δ = 0.82 (d, *J* = 6.4 Hz, 3H), 1.07 (d, *J* = 6.4 Hz, 3H), 2.48 (m, 1H), 3.74 (s, 3H), 3.89 (s, 3H), 6.15 (d, *J* = 10.0 Hz, 1H), 6.35 (dd, *J* = 4.0, 2.8 Hz, 1H), 6.92 (dd, *J* = 4.0, 2.0 Hz, 1H), 7.00–7.05 (m, 2H), 7.29 (dd, *J* = 2.8, 2.0 Hz, 1H), 8.01–8.05 (m, 2H), 7.99 (dd, *J* = 7.6, 2.0 Hz, 1H) ppm; ^13^C-NMR (CDCl_3_) δ = 18.5, 19.3, 33.1, 52.3, 55.5, 64.5, 110.1, 113.8, 114.5, 116.4, 118.2, 125.6, 128.6, 159.0, 162.2, 162.6, 171.3 ppm; IR ν = 2970, 2875, 1745, 1610, 1600, 1505, 1450, 1395, 1300, 1270, 1240, 1220, 1200, 1180, 1160, 1135, 1100, 1080, 1015, 995, 910, 890, 735, 715, 685, 650, 615 cm^−1^; HRMS (ESI) calcd for C_19_H_21_N_3_O_4_+Na 378.1424: found 378.1426.

*Methyl (2S,3S)-2-(2-(5-(2-fluorophenyl)-1,3,4-oxadiazol-2-yl)-1H-pyrrol-1-yl)-3-methylpentanoate* (**6a**). Orange-red oil, 89% yield (1.03 g, 2.89 mmol); R_f_ = 0.47 (4:1 hexane/EtOAc); Data for **6a**: ^1^H-NMR (CDCl_3_) δ = 0.84 (t, *J* = 7.2 Hz, 3H), 1.05 (d, *J* = 6.4 Hz, 3H), 1.06–1.27 (m, 2H), 2.21–2.32 (m, 1H), 3.74 (s, 3H), 6.20 (d, *J* = 9.6 Hz, 1H), 6.36 (dd, *J* = 4.0, 2.8 Hz, 1H), 6.98 (dd, *J* = 4.0, 2.0 Hz, 1H), 7.23–7.29 (m, 1H), 7.30–7.34 (m, 1H), 7.31 (dd, *J* = 2.8, 2.0 Hz, 1H), 7.50–7.57 (m, 1H), 8.08–8.13 (m, 1H) ppm; ^13^C-NMR (CDCl_3_) δ = 11.0, 15.6, 24.8, 39.2, 52.3, 63.8, 110.3, 114.6, 117.0 (d, *J* = 21.3 Hz), 117.9, 124.6 (d, *J* = 3.8 Hz), 126.0, 129.6 (d, *J* = 0.5 Hz), 133.3 (d, *J* = 8.4 Hz), 158.8, 159.4, (d, *J* = 5.4 Hz), 159.6 (d, *J* = 1.5 Hz), 161.3, 171.4 ppm; IR ν = 2970, 2937, 2880, 1750, 1600, 1500, 1475, 1450, 1400, 1260, 1235, 1198, 1180, 1100, 1080, 1026, 1000, 910, 825, 767, 735, 698, 670, 615 cm^−1^; HRMS (ESI) calcd for C_19_H_20_FN_3_O_3_+Na 380.1381:found 380.1386.

*Methyl (2S,3S)-2-(2-(5-(3-fluorophenyl)-1,3,4-oxadiazol-2-yl)-1H-pyrrol-1-yl)-3-methylpentanoate* (**6b**). Orange-red oil, 88% yield (0.99 g, 2.77 mmol); R_f_ = 0.47 (4:1 hexane/EtOAc); Data for **6b**: ^1^H-NMR (CDCl_3_) δ = 0.84 (t, *J* = 7.2 Hz, 3H), 1.05 (d, *J* = 6.4 Hz, 3H), 1.06–1.28 (m, 2H), 2.20–2.32 (m, 1H), 3.75 (s, 3H), 6.19 (d, *J* = 10.0 Hz, 1H), 6.36 (dd, *J* = 3.6, 2.8 Hz, 1H), 6.97 (dd, *J* = 3.6, 2.0 Hz, 1H), 7.20–7.27 (m, 1H), 7.33 (dd, *J* = 2.8, 2.0 Hz, 1H), 7.47–7.53 (m, 1H), 7.76–7.81 (m, 1H), 7.87–7.91 (m, 1H) ppm; ^13^C-NMR (CDCl_3_) δ = 10.9, 15.5, 24.8, 39.1, 52.3, 63.8, 110.4, 113.8 (d, *J* = 24.2 Hz), 114.5, 117.7, 118.6 (d, *J* = 21.3 Hz), 122.5 (d, *J* = 3.0 Hz), 125.7 (d, *J* = 8.3 Hz), 126.1, 130.9 (d, *J* = 8.4 Hz), 159.6, 161.6 (d, *J* = 3.1 Hz), 164.0, 171.3 ppm; IR ν = 2970, 2940, 2880, 1750, 1595, 1560, 1505, 1490, 1454, 1415, 1245, 1205, 1178, 1105, 1080, 995, 915, 870, 795, 735, 680, 615 cm^−1^; HRMS (ESI) calcd for C_19_H_20_FN_3_O_3_+Na 380.1381, found 380.1384.

*Methyl (2S,3S)-2-(2-(5-(4-fluorophenyl)-1,3,4-oxadiazol-2-yl)-1H-pyrrol-1-yl)-3-methylpentanoate* (**6c**). Yellow oil, 62% yield (768 mg, 2.15 mmol); R_f_ = 0.58 (4:1 hexane/EtOAc); Data for **6c**: ^1^H-NMR (CDCl_3_) δ = 0.83 (t, *J* = 7.2 Hz, 3H), 1.04 (d, *J* = 6.4 Hz, 3H), 1.05–1.27 (m, 2H), 2.22–2.32 (m, 1H), 3.74 (s, 3H), 6.19 (d, *J* = 10.4 Hz, 1H), 6.36 (dd, *J* = 3.6, 2.8 Hz, 1H), 6.94 (dd, *J* = 3.6, 2.0 Hz, 1H), 7.18–7.25 (m, 2H), 7.31 (dd, *J* = 2.8, 2.0 Hz, 1H), 8.07–8.13 (m, 2H) ppm; ^13^C-NMR (CDCl_3_) δ = 10.9, 15.5, 24.8, 39.1, 52.3, 63.7, 110.3, 114.2, 116.4 (d, *J* = 22.8 Hz), 117.9, 126.0, 129.1 (d, *J* = 8.3 Hz), 159.4, 161.8, 163.4, 165.9, 171.4 ppm; IR ν = 2970, 2940, 2880, 1750, 1670, 1605, 1500, 1455, 1415, 1240, 1200, 1180, 1160, 1080, 1015, 1000, 850, 740, 620 cm^−1^; HRMS (ESI) calcd for C_19_H_20_FN_3_O_3_+Na 380.1381: found 380.1387.

*Methyl (2S,3S)-2-(2-(5-(4-chlorophenyl)-1,3,4-oxadiazol-2-yl)-1H-pyrrol-1-yl)-3-methylpentanoate* (**6d**). Orange oil, 79% yield (254 mg, 0.68 mmol); R_f_ = 0.65 (4:1 hexane/EtOAc); Data for **6d**: ^1^H-NMR (CDCl_3_) δ = 0.83 (t, *J* = 7.2 Hz, 3H), 1.04 (d, *J* = 6.4 Hz, 3H), 1.06–1.27 (m, 2H), 2.20–2.32 (m, 1H), 3.74 (s, 3H), 6.19 (d, *J* = 10.4 Hz, 1H), 6.36 (dd, *J* = 4.0, 2.8 Hz, 1H), 6.95 (dd, *J* = 4.0, 1.6 Hz, 1H), 7.32 (dd, *J* = 2.8, 1.6 Hz, 1H), 7.48–7.52 (m, 2H), 8.01–8.05 (m, 2H) ppm; ^13^C-NMR (CDCl_3_) δ = 10.9, 15.5, 24.8, 39.1, 52.3, 63.8, 110.3, 114.4, 117.8, 122.3, 126.1, 128.1, 129.4, 137.8, 159.5, 161.8, 171.3 ppm; IR ν = 2970, 2935, 2880, 1750, 1605, 1505, 1485, 1455, 1410, 1255, 1235, 1200, 1178, 1095, 1080, 1015, 840, 735 cm^−1^; HRMS (ESI) calcd for C_19_H_20_ClN_3_O_3_+Na 396.1085: found 396.1089.

*Methyl (2S,3S)-2-(2-(5-(2-hydroxy-3,5-diiodophenyl)-1,3,4-oxadiazol-2-yl)-1H-pyrrol-1-yl)-3-methylpentanoate* (**6e-1**). Green solid, 45% yield (893 mg, 1.47 mmol); R_f_ = 0.50 (4:1 hexane/EtOAc); Data for **6e-1**: ^1^H-NMR (CDCl_3_) δ = 0.83 (t, *J* = 7.2 Hz, 3H), 1.05 (d, *J* = 6.4 Hz, 3H), 1.02–1.22 (m, 2H), 2.20–2.33 (m, 1H), 3.77 (s, 3H), 6.04 (d, *J* = 9.6 Hz, 1H), 6.39 (dd, *J* = 4.0, 2.8 Hz, 1H), 7.04 (dd, *J* = 4.0, 1.6 Hz, 1H), 7.37 (dd, *J* = 2.8, 1.6 Hz, 1H), 8.06 (d, *J* = 2.0 Hz, 1H), 8.17 (d, *J* = 2.0 Hz, 1H), 11.02 (s, 1H) ppm; ^13^C-NMR (CDCl_3_) δ = 10.9, 15.5, 24.8, 38.1, 52.4, 64.0, 81.3, 86.7, 109.8, 110.8, 115.7, 116.9, 127.0, 134.7, 149.8, 156.2, 158.7, 160.1, 171.1 ppm; IR ν = 3415, 2970, 2880, 1750, 1605, 1565, 1530, 1500, 1455, 1415, 1380, 1255, 1235, 1190, 1180, 1100, 1080, 990, 915, 875, 758, 740, 665, 598 cm^−1^; HRMS (ESI) calcd for C_19_H_19_I_2_N_3_O_4_+Na 629.9357: found 629.9358.

*Methyl (2S,3S)-2-(2-(5-(4-hydroxy-3,5-diiodophenyl)-1,3,4-oxadiazol-2-yl)-1H-pyrrol-1-yl)-3-methylpentanoate* (**6f-1**). Yellow oil, 43% yield (935 mg, 1.54 mmol); R_f_ = 0.45 (4:1 hexane/EtOAc); Data for **6f-1**: ^1^H-NMR (CDCl_3_) δ = 0.83 (t, *J* = 7.2 Hz, 3H), 1.04 (d, *J* = 6.4 Hz, 3H), 1.05–1.24 (m, 2H), 2.20–2.32 (m, 1H), 3.75 (s, 3H), 6.14 (d, *J* = 10.0 Hz, 1H), 6.21 (br s, 1H), 6.36 (dd, *J* = 4.0, 2.8 Hz, 1H), 6.96 (dd, *J* = 4.0, 2.0 Hz, 1H), 7.32 (dd, *J* = 2.8, 2.0 Hz, 1H), 8.40 (s, 2H), 8.17 (d, *J* = 2.0 Hz, 1H), 11.02 (s, 1H) ppm; ^13^C-NMR (CDCl_3_) δ = 10.9, 15.5, 24.8, 39.1, 52.3, 63.8, 82.5, 110.4, 114.5, 117.7, 119.9, 126.1, 137.6, 156.2, 159.4, 159.6, 171.3 ppm; IR ν = 3450, 3145, 3125, 3070, 2970, 2880, 1745, 1608, 1595, 1500, 1450, 1395, 1300, 1235, 1195, 1178, 1155, 1105, 1075, 990, 890, 775, 736, 710, 685, 615 cm^−1^; HRMS (ESI) calcd for C_19_H_19_I_2_N_3_O_4_+Na 629.9357: found 629.9360.

*Methyl (2S,3S)-2-(2-(5-(2-methoxyphenyl)-1,3,4-oxadiazol-2-yl)-1H-pyrrol-1-yl)-3-methylpentanoate* (**6g**). Orange-red oil, 80% yield (768 mg, 2.08 mmol); R_f_ = 0.19 (4:1 hexane/EtOAc); Data for **6g**: ^1^H-NMR (CDCl_3_) δ = 0.83 (t, *J* = 7.2 Hz, 3H), 1.04 (d, *J* = 6.4 Hz, 3H), 1.05–1.27 (m, 2H), 2.20–2.32 (m, 1H), 3.73 (s, 3H), 3.99 (s, 3H), 6.24 (d, *J* = 9.6 Hz, 1H), 6.34 (dd, *J* = 3.6, 2.8 Hz, 1H), 6.94 (dd, *J* = 3.6, 1.6 Hz, 1H), 7.05–7.11 (m, 2H), 7.30 (dd, *J* = 2.8, 1.6 Hz, 1H), 7.47–7.53 (m, 1H), 7.98 (dd, *J* = 7.6, 2.0 Hz, 1H) ppm; ^13^C-NMR (CDCl_3_) δ = 11.0, 15.5, 24.8, 39.2, 52.2, 56.0, 63.7, 110.1, 112.0, 112.9, 114.0, 118.3, 120.7, 125.6, 130.2, 132.8, 157.9, 159.0, 161.3, 171.5 ppm; IR ν = 2970, 2942, 2882, 2845, 1750, 1605, 1550, 1500, 1485, 1455, 1440, 1415, 1255, 1240, 1200, 1180, 1100, 1080, 1050, 1025, 915, 730, 678, 650, 620 cm^−1^; HRMS (ESI) calcd for C_20_H_23_N_3_O_4_+Na 392.1581: found 392.1585.

*Methyl (2S,3S)-2-(2-(5-(4-methoxyphenyl)-1,3,4-oxadiazol-2-yl)-1H-pyrrol-1-yl)-3-methylpentanoate* (**6h**). Orange oil; 79% yield (739 mg, 2.00 mmol); R_f_ = 0.24 (4:1 hexane/EtOAc); Data for **6h**: ^1^H-NMR (CDCl_3_) δ = 0.83 (t, *J* = 7.2 Hz, 3H), 1.03 (d, *J* = 6.4 Hz, 3H), 1.05–1.23 (m, 2H), 2.20–2.30 (m, 1H), 3.74 (s, 3H), 3.88 (s, 3H), 6.20 (d, *J* = 9.6 Hz, 1H), 6.34 (dd, *J* = 3.6, 2.8 Hz, 1H), 6.92 (dd, *J* = 3.6, 1.6 Hz, 1H), 6.99–7.03 (m, 2H), 7.29 (dd, *J* = 2.8, 1.6 Hz, 1H), 8.00–8.04 (m, 2H) ppm; ^13^C-NMR (CDCl_3_) δ = 11.0, 15.5, 24.8, 39.1, 52.2, 55.4, 63.7, 110.1, 113.8, 114.5, 116.4, 118.2, 125.6, 128.6, 158.9, 162.2, 162.6, 171.4 ppm; IR ν = 2970, 2940, 2880, 2840, 1750, 1610, 1500, 1460, 1445, 1310, 1255, 1180, 1100, 1080, 1030, 1000, 915, 840, 730, 700, 625, 610 cm^−1^; HRMS (ESI) calcd for C_20_H_23_N_3_O_4_+Na 392.1581: found 392.1584.

### 3.2. General Procedure for Deiodination Reaction on the Phenol Ring

Zn dust (2~5 equiv.) and acetic acid (5 equiv.) were added to a stirred solution of 2-pyrrolyl-5-(iodophenolic)-1,3,4-oxadiazole **5f-1** or **6e-1** (~0.5–1.0 g, 1 equiv.) in THF (30mL). The mixture was stirred at 25 °C for 1~6 h under an argon atmosphere. The mixture was quenched with saturated NaHCO_3_ solution, extracted with Et_2_O, dried over anhydrous Na_2_SO_4_, filtered, and concentrated under reduced pressure. The product was purified by SiO_2_ flash column chromatography to produce the deiodination product.

*Methyl (S)-2-(2-(5-(4-hydroxyphenyl)-1,3,4-oxadiazol-2-yl)-1H-pyrrol-1-yl)-3-methylbutanoate* (**5f**). White solid, 97% yield (270 mg, 0.79 mmol); R_f_ = 0.58 (3:2 hexane/EtOAc); Data for **5f**: ^1^H-NMR (CDCl_3_) δ = 0.83 (d, *J* = 6.4 Hz, 3H), 1.08 (d, *J* = 6.4 Hz, 3H), 2.43–2.54 (m, 1H), 3.75 (s, 3H), 6.13 (d, *J* = 9.6 Hz, 1H), 6.36 (dd, *J* = 4.0, 2.8 Hz, 1H), 6.93 (dd, *J* = 4.0, 1.6 Hz, 1H), 7.01 (d, *J* = 8.4 Hz, 2H), 7.30 (dd, *J* = 2.8, 1.6 Hz, 1H), 7.99 (d, J = 8.4 Hz, 2H) ppm; ^13^C-NMR (CDCl_3_) δ = 18.5, 19.3, 33.1, 52.3, 64.6, 110.2, 114.0, 116.0, 116.2, 118.0, 125.7, 128.8, 159.0, 159.1, 162.7, 171.3 ppm; IR ν = 3120, 2970, 2940, 2880, 1750, 1610, 1595, 1500, 1440, 1394, 1375, 1335, 1285, 1270, 1240, 1200, 1105, 1090, 1075, 1010, 995, 915, 840, 775, 735, 719, 635 cm^−1^; HRMS (ESI) calcd for C_18_H_19_N_3_O_4_+Na 364.1268: found 365.1271.

*Methyl (2S,3S)-2-(2-(5-(2-hydroxyphenyl)-1,3,4-oxadiazol-2-yl)-1H-pyrrol-1-yl)-3-methylpentanoate* (**6e**). White solid, 50% yield (210 mg, 0.59 mmol); R_f_ = 0.54 (3:2 hexane/EtOAc); Data for **6e**: ^1^H-NMR (DMSO-d_6_) δ = 0.79 (t, *J* = 7.2 Hz, 3H), 0.96 (d, *J* = 6.4 Hz, 3H), 0.98–1.19 (m, 2H), 2.16–2.26 (m, 1H), 3.69 (s, 3H), 6.02 (d, *J* = 9.6 Hz, 1H), 6.24 (dd, *J* = 3.6, 2.8 Hz, 1H), 6.56 (dd, *J* = 3.6, 1.6 Hz, 1H), 6.92–6.99 (m, 2H), 7.20 (dd, *J* = 2.8, 1.6 Hz, 1H), 7.41–7.46 (m, 1H), 7.86–7.90 (m, 1H) 8.39 (s, 1H) ppm; ^13^C-NMR (DMSO-d_6_) δ = 11.1, 16.0, 24.8, 38.5, 52.7, 63.3, 110.3, 115.9, 116.5, 117.8, 119.3, 126.2, 127.8, 128.6, 134.2, 142.5, 159.8, 164.9, 171.3 ppm; IR ν = 3240, 3080, 2970, 2935, 2880, 1750, 1640, 1600, 1570, 1540, 1495, 1465, 1425, 1360, 1335, 1315, 1258, 1230, 1200, 1175, 1155, 1105, 1075, 1060, 1040, 995, 950, 895, 825, 757, 740, 670, 618 cm^−1^; HRMS (ESI) calcd for C_19_H_23_N_3_O_4_+Na 380.1581: found 380.1586.

*Methyl (2S,3S)-2-(2-(5-(4-hydroxyphenyl)-1,3,4-oxadiazol-2-yl)-1H-pyrrol-1-yl)-3-methylpentanoate* (**6f**). White solid, 42% yield (98 mg, 0.28 mmol); R_f_ = 0.67 (3:2 hexane/EtOAc); Data for **6f**: ^1^H-NMR (CDCl_3_) δ = 0.77 (t, *J* = 7.2 Hz, 3H), 0.98 (d, *J* = 6.8 Hz, 3H), 0.98–1.18 (m, 2H), 2.26–2.35 (m, 1H), 3.70 (s, 3H), 5.96 (d, *J* = 10.0 Hz, 1H), 6.38 (dd, *J* = 3.6, 2.8 Hz, 1H), 6.97 (d, *J* = 8.4 Hz, 2H), 6.98 (dd, *J* = 3.6, 1.6 Hz, 1H), 7.40 (dd, *J* = 2.8, 1.6 Hz, 1H), 7.90 (d, *J* = 8.4 Hz, 2H), 10.34 (s, 1H) ppm; ^13^C-NMR (CDCl_3_) δ = 10.9, 15.5, 24.8, 39.1, 52.3, 63.7, 110.3, 114.2, 115.5, 117.9, 125.9, 129.0, 136.9, 157.7, 159.2, 161.1, 171.4 ppm; IR ν = 3137, 2965, 2875, 2838, 1742, 1610, 1579, 1558, 1497, 1464, 1466, 1438, 1338, 1307, 1287, 1254, 1224, 1202, 1173, 1092, 1060, 1027, 961, 938, 911, 837, 810, 798, 731, 696 cm^−1^; HRMS (ESI) calcd for C_19_H_21_N_3_O_4_+Na 378.1424: found 378.1427.

### 3.3. General Procedure for Iodination Reaction on the Pyrrole Ring

I_2_ (4 equiv.). was added to a stirred solution of 2-pyrrolyl-5-(anisyl)-1,3,4-oxadiazoles **5g**, **5h**, or **6h** (~1.0 g, 1 equiv.) in DMSO (10 mL). The mixture was heated at 90 ℃ for 3~5 h under an argon atmosphere. After cooling to room temperature, the mixture was diluted with Et_2_O, washed with 10% Na_2_S_2_O_3_ solution, dried over anhydrous Na_2_SO_4_, filtered, and concentrated under reduced pressure. The product was purified by SiO_2_ flash column chromatography to produce iodination products **5g-1**, **5h-1**, or **6h-1** on the pyrrole ring.

*Methyl (S)-2-(3,4-diiodo-2-(5-(2-methoxyphenyl)-1,3,4-oxadiazol-2-yl)-1H-pyrrol-1-yl)-3-methylbutanoate* (**5g-1**). Orange oil, 61% yield (923 mg, 1.52 mmol); R_f_ = 0.60 (3:2 hexane/EtOAc); Data for **5g-1**: ^1^H-NMR (CDCl_3_) δ = 0.86 (d, *J* = 6.4 Hz, 3H), 1.04 (d, *J* = 6.4 Hz, 3H), 2.42 (m, 1H), 3.73 (s, 3H), 4.03 (s, 3H), 6.12 (d, *J* = 10.0 Hz, 1H), 7.08–7.14 (m, 2H), 7.45 (s, 1H), 7.51–7.56 (m, 1H), 8.12 (dd, *J* = 7.6, 1.6 Hz, 1H) ppm; ^13^C-NMR (CDCl_3_) δ = 18.6, 19.2, 33.1, 52.5, 56.0, 65.6, 78.0, 80.4, 112.0, 112.3, 120.8, 121.4, 130.4, 130.5, 133.2, 156.7, 158.1, 162.1, 170.6 ppm; IR ν = 2970, 2937, 2880, 2840, 1750, 1665, 1605, 1590, 1545, 1500, 1475, 1470, 1435, 1390, 1285, 1265, 1220, 1205, 1185, 1165, 1130, 1060, 1050, 1025, 943, 915, 768, 755, 735, 677, 650 cm^−1^; HRMS (ESI) calcd for C_19_H_19_I_2_N_3_O_4_+Na 629.9357: found 629.9363.

*Methyl (S)-2-(3,4-diiodo-2-(5-(4-methoxyphenyl)-1,3,4-oxadiazol-2-yl)-1H-pyrrol-1-yl)-3-methylbutanoate* (**5h-1**). Yellow oil (a 1.3:1 mixture of di- and mono-iodide products); di-iodide, 39% yield (199 mg, 0.33 mmol, calcd), mono-iodide, 30% yield (121 mg, 0.25 mmol, calcd); R_f_ = 0.60 (3:2 hexane/EtOAc); Data for di-iodide at pyrrole: ^1^H-NMR (CDCl_3_) δ = 0.86 (d, *J* = 6.4 Hz, 3H), 1.05 (d, *J* = 6.4 Hz, 3H), 2.43 (m, 1H), 3.74 (s, 3H), 3.89 (s, 3H), 6.09 (d, *J* = 10.0 Hz, 1H), 7.05 (d, *J* = 8.8 Hz, 2H), 7.44 (s, 1H), 8.11 (d, *J* = 8.8 Hz, 2H) ppm; ^13^C-NMR (CDCl_3_) δ = 18.6, 19.1, 33.0, 52.5, 55.5, 65.7, 78.0, 80.4, 114.6, 116.0, 120.3, 128.9, 130.6, 156.7, 162.5, 163.4, 170.5 ppm; IR ν = 2970, 1750, 1615, 1590, 1560, 1500, 1485, 1460, 1440, 1390, 1335, 1255, 1205, 1175, 1160, 1065, 1030, 945, 840, 755, 745, 625 cm^−1^; HRMS (ESI) calcd for C_19_H_19_I_2_N_3_O_4_+Na 629.9357, found 629.9358. Data for mono-iodide at pyrrole (C-3): ^1^H-NMR (CDCl_3_) δ = 0.61 (d, *J* = 6.4 Hz, 3H), 1.06 (d, *J* = 6.4 Hz, 3H), 2.43 (m, 1H), 3.76 (s, 3H), 3.89 (s, 3H), 6.13 (d, *J* = 9.6 Hz, 1H), 7.01 (d, *J* = 2.0 Hz, 1H), 7.02 (d, *J* = 8.4 Hz, 2H), 7.35 (d, *J* = 2.0 Hz, 1H), 8.02 (d, *J* = 8.4 Hz, 2H) ppm; ^13^C-NMR (CDCl_3_) δ = 18.5, 19.2, 33.3, 52.4, 61.3, 64.9, 77.2, 114.5, 116.0, 120.1, 121.5, 128.6, 130.1, 157.7, 162.4, 162.8, 170.8 ppm; HRMS (ESI) calcd for C_19_H_20_IN_3_O_4_+Na 504.0396: found 504.0387.

*Methyl (2S,3S)-2-(3-iodo-2-(5-(4-methoxyphenyl)-1,3,4-oxadiazol-2-yl)-1H-pyrrol-1-yl)-3-methylpentanoate* (**6h-1**). Yellow oil, 83% yield (308 mg, 0.67 mmol); R_f_ = 0.40 (4:1 hexane/EtOAc); Data for **6h-1**: ^1^H-NMR (CDCl_3_) δ = 0.84 (t, *J* = 7.2 Hz, 3H), 1.02 (d, *J* = 6.4 Hz, 3H), 1.06–1.26 (m, 2H), 2.16–2.28 (m, 1H), 3.75 (s, 3H), 3.89 (s, 3H), 6.18 (d, *J* = 10.0 Hz, 1H), 6.99–7.04 (m, 2H), 7.01 (d, *J* = 1.6 Hz, 1H), 7.35 (d, *J* = 1.6 Hz, 1H), 7.99–8.03 (m, 2H) ppm; ^13^C-NMR (CDCl_3_) δ = 10.9, 15.4, 24.8, 39.2, 52.4, 55.5, 64.1, 77.2, 114.6, 116.0, 120.1, 120.3, 128.7, 130.1, 157.6, 162.4, 162.9, 171.0 ppm; IR ν = 2970, 2935, 2880, 2840, 1750, 1607, 1498, 1464, 1440, 1310, 1260, 1176, 1100, 1065, 1030, 1000, 915, 840, 815, 745, 640, 625, 607 cm^−1^; HRMS (ESI) calcd for C_20_H_22_IN_3_O_4_+Na 518.0547: found 518.0548.

### 3.4. Biological Evaluation

The minimum inhibitory concentrations (MICs) were determined using the broth microdilution method in a 96-well plate [40,41]. The 96-well plates containing chemicals in two-fold serial dilutions (4 μg/mL to 2048 μg/mL for series **1**; 2 μg/mL to 1024 μg/mL for series **5** and **6**) were prepared in Luria–Bertani (LB) medium. *E. coli*, *S. aureus*, and *A. baumannii* cells were grown in LB broth to the exponential phase. A 10 μL volume of cells diluted with LB broth to a concentration of 10^8^ cells/mL was inoculated on the plates. The MIC was determined after incubation at 37 °C for 16 h under aerobic conditions. The optical density was measured in triple at 600 nm (OD_600_) using a microplate reader (Bio-Rad, USA) at 20 h after treatment of the chemicals in concentrations of 2, 4, 8, 16, 32, 64, 128, 256, 512, and 1024 μg/mL. The average and standard deviation values of OD_600_ are reported in Table S1 and Table S2 of the Supporting Information. Vancomycin and Erythromycin were used as positive controls (see Table S2 for the average and standard deviation values of OD_600_). As the chemicals were dissolved in 100% DMSO, 100% DMSO and triple-distilled water were used as negative controls. The minimum inhibitory concentration (MIC) in Table 2 and Table 4 is defined as the lowest concentration of chemicals which provides an average OD_600_ value of less than 0.100.

## 4. Conclusions

We extended the synthetic utility of pyrrole platform chemicals **2**, which can be readily prepared from the sustainable ribose conversion with amino acids, to the pyrrole-ligated 1,3,4-oxadiazole core structure **1** through the reaction with benzohydrazide **3a**. The size effect of the R group from the amino acids clearly offered better antibacterial activities for 1,3,4-oxadiazoles **1c** and **1e**, which were derived from valine and isoleucine, respectively. Benzohydrazides **3** with various electronic X-substituents were utilized for the construction of 2-pyrrolyl-5-phenyl-1,3,4-oxadiazoles **5** and **6** with *N*-valine and *N*-isoleucine residues, respectively. Relationships of structure and antibacterial activity were deduced from MIC values for 1,3,4-oxadiazoles **5** and **6** against *E. coli, S. aureus* and *A. baumannii*. A positive *ortho* effect was marginally observed for fluoride substituents. Most importantly, a superior iodophenol effect was evident in the antibacterial activities of 1,3,4-oxadiazoles **5f-1** and **6f-1**, which provided much lower MIC values against *A. baumannii* than those of the vancomycin and erythromycin as positive controls. These findings provide a guiding principle for the design of superior future antimicrobial agents.

## Figures and Tables

**Table 1:** Yields of pyrrole-2-carbaldehydes **2**, *N*-benzoylhydrazones **4**, and 1,3,4-oxadiazoles **1** from D-ribose conversion with L-amino acids in Scheme 1.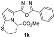.

Entry	Amino Acid (R)	Yield 2 (%) ^1^	Yield 4 (%) ^2^	Yield 1 (%) ^2^
**a**	Gly (H)	32	96	80
**b**	Ala (Me)	38	88	91
**c**	Val (*i*-Pr)	42	83	98
**d**	Leu (*i*-Bu)	63	77	87
**e**	Ile (*s*-Bu)	40	86	90
**f**	Phe (PhCH_2_)	54	83	86
**g**	Bn (PhCH_2_CH_2_)	53	79	86
**h**	AsP (MeO_2_CCH_2_)	47	70	88
**i**	Glu (MeO_2_CCH_2_CH_2_)	37	81	95
**j**	Met (MeSCH_2_CH_2_)	46	82	50 ^3^

^1^ Yields from one-pot reaction [32]. ^2^ Isolated yields after SiO_2_ flash column chromatography. ^3^ Cyclized product **1k** was also obtained in 49% yield.

**Table 2:** Minimum inhibition activity (MIC) of 2-pyrrolyl-5-phenyl-1,3,4-oxadiazoles **1** against *E. coli* and *S. aureus*.

Entry	Compound	Amino Acid (R)	MIC/*E. coli* (μg/mL)	MIC/*S. aureus* (μg/mL)
1	**1a**	Gly (H)	>2048	2048
2	**1b**	Ala (Me)	512	512
3	**1c**	Val (*i*-Pr)	256	128
4	**1d**	Leu (*i*-Bu)	512	128
5	**1e**	Ile (*s*-Bu)	256	64
6	**1f**	Phe (PhCH_2_)	1024	512
7	**1g**	Bn (PhCH_2_CH_2_)	1024	512
8	**1h**	Asp (MeO_2_CCH_2_)	2048	1024
9	**1i**	Glu (MeO_2_CCH_2_CH_2_)	2048	2048
10	**1j**	Met (MeSCH_2_CH_2_)	>2048	2048
11	**2k**	*c*-Met (SCH_2_CH_2_)	256	256

**Table 3:** Yields of various 1,3,4-oxadiazoles **5** and **6** with various electronic substituents X, derived from valine and isoleucine in Scheme 2.

Entry	X	Yield 5 (%)	Yield 6 (%)
**a**	*o*-F	40	89
**b**	*m*-F	70	88
**c**	*p*-F	54	62
**d**	*p*-Cl	62	79
**e-1**	*o*-OH (I)	33 ^1^	45 ^2^
**e**	*o*-OH	-	50 ^3^
**f-1**	*p*-OH (I)	32 ^4^	43 ^5^
**f**	*p*-OH	97 ^6^	42 ^7^
**g**	*o*-OMe	85	80
**g-1**	*o*-OMe	61 ^8^	-
**h**	*p*-OMe	70	79
**h-1**	*p*-OMe	69 ^9^	83 ^10^

The structures of ^1^ **5e-1**, ^2^ **6e-1**, ^4^ **5f-1**, ^5^ **6f-1**. ^8^ **5g-1**, ^9^ **5h-1**, and ^10^ **6h-1** are depicted in Figure 2, which were produced by extra iodination reactions. The compounds ^3^ **6e**, ^6^ **5f**, and ^7^ **6f** were prepared by deiodination using Zn in AcOH at 25 °C from **6e-1**, **5f-1**, and **6f-1**, respectively. The compounds ^8^ **5g-1**, ^9^ **5h-1**, and ^10^ **6h-1** were prepared by separate iodination reaction (I_2_ in DMSO) at 85 °C for 6 h from **5g**, **5h**, and **6h**, respectively.

**Table 4:** Minimum inhibition concentration (MIC) of 1,3,4-oxadiazoles **5** and **6** against *E. coli*, *S. aureus*, and *A. baumannii*.

Entry	Comp’d	X	*E. coli* (μg/mL)	*S. aureus* (μg/mL)	*A. baumannii* (μg/mL)
1	**5a**	*o*-F	512	256	128
2	**5b**	*m*-F	>1024	>1024	512
3	**5c**	*p*-F	>1024	>1024	256
4	**5d**	*p*-Cl	512	256	512
5	**5e-1**	*o*-OH (I) ^1^	1024	4	64
6	**5f-1**	*p*-OH (I) ^1^	1024	4	<2
7	**5f**	*p*-OH	>1024	128	128
8	**5g**	*o*-OMe	>1024	512	256
9	**5g-1**	*o*-OMe (I) ^2^	512	512	256
10	**5h**	*p*-OMe	>1024	512	256
11	**5h-1**	*p*-OMe (I) ^2^	512	256	128
12	**6a**	*o*-F	512	256	256
13	**6b**	*m*-F	1024	256	256
14	**6c**	*p*-F	>1024	512	512
15	**6d**	*p*-Cl	512	512	256
16	**6e-1**	*o*-OH (I) ^1^	1024	<2	512
17	**6e**	*o*-OH	256	64	64
18	**6f-1**	*p*-OH (I) ^1^	1024	<2	8
19	**6f**	*p*-OH	1024	8	512
20	**6g**	*o*-OMe	>1024	512	256
21	**6h**	*p*-OMe	>1024	256	256
22	**6h-1**	*p*-OMe (I) ^2^	1024	512	512
23	Vancomycin	-	>1024	4	>1024
24	Erythromycin	-	1024	4	128

^1^ Extra iodination product on the phenol ring. ^2^ Extra iodination product on the pyrrole ring. The structures of extra iodination products are depicted in Figure 2.

## Data Availability

Data are contained within the article.

## Supplementary Materials

The following supporting information can be downloaded at: https://www.mdpi.com/article/10.3390/molecules28083638/s1, (1) ^1^H/^13^C-NMR spectra; (2) MIC data against *E. coli*, *S. aureus*, and *A. baumannii*; (3) High-Resolution Mass Spectra for the entire 1,3,4-oxadiazoles synthesized in this paper. Table S1. Determination of MIC for **1** for *E. coli* and *S. aureus* by OD600 (20 h). Each value was obtained as an average of at least triple measurements. Table S2. Determination of MIC for **5** and **6** for *E. coli*, *S. aureus,* and *A. baumannii* by OD600 (20 h). Each value was obtained as an average of at least triple measurements (vancomycin and erythromycin as positive controls).
