# Supplementary material for: Rapid Assembly of Pyrrole-Ligated 1,3,4-Oxadiazoles and Excellent Antibacterial Activity of Iodophenol Substituents
Source: Molecules. 2023 Apr 21;28(8):3638. doi: 10.3390/molecules28083638 (PMC10142073; doi:10.3390/molecules28083638)

Supporting Information for

Rapid assembly of pyrrole-ligated 1,3,4-oxadiazoles and excellent antibacterial activity by iodophenol substituents

Hyein Kim,[†1] Lina Gu, [†1,2] Huisu Yeo,[1] Umji Choi,[3] Chang-Ro Lee,*[3] Haiyang Yu,[4] and Sangho Koo*[1,2]

[1] Department of Chemistry, Myongji University, Myongji-Ro 116, Cheoin-Gu, Yongin, Gyeonggi-Do 17058, Korea; [2] School of Pharmacy, East China University of Science and Technology, Meilong Road 130, Shanghai, 200237, China; [3] Department of Biological Sciences and Bioinformatics, Myongji University, Myongji-Ro 116, Cheoin-Gu, Yongin, Gyeonggi-Do 17058, Korea; [4] Tianjin State Key Laboratory of Modern Chinese Medicine, Tianjin University of Traditional Chinese Medicine, Tianjin 300193, China

†These authors contributed equally; Corresponding authors: *crlee@mju.ac.kr; *sangkoo@mju.ac.kr

(1) $^{1}H/^{13}C$-NMR Spectra -- S-2

(2) MIC Data for *E. coli*, *S. aureus*, and *A. baumannii* --- S-87

(3) High-Resolution Mass Spectra -- S-97

Sample Name:
LN-361-2
Data Collected on:
Agilent-NMR.com-vnmrs400
Archive directory:
/home/vnmr1/vnmrsys/data/2011-koo-4
Sample directory:
LN-361-2_01
FidFile: LN-361-2_PROTON_01

Pulse Sequence: PROTON (s2pul)
Solvent: dmso
Data collected on: Nov 10 2020

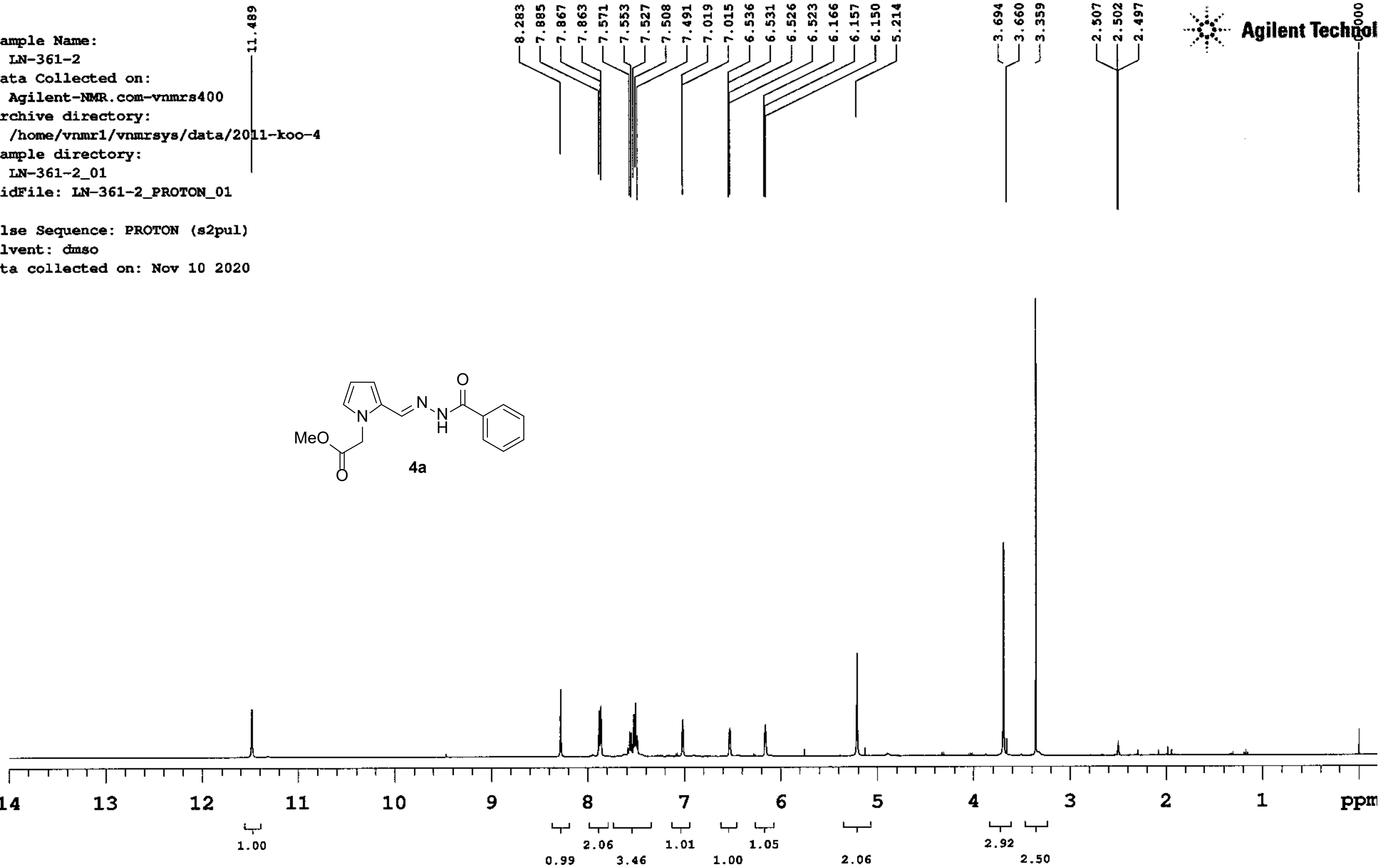

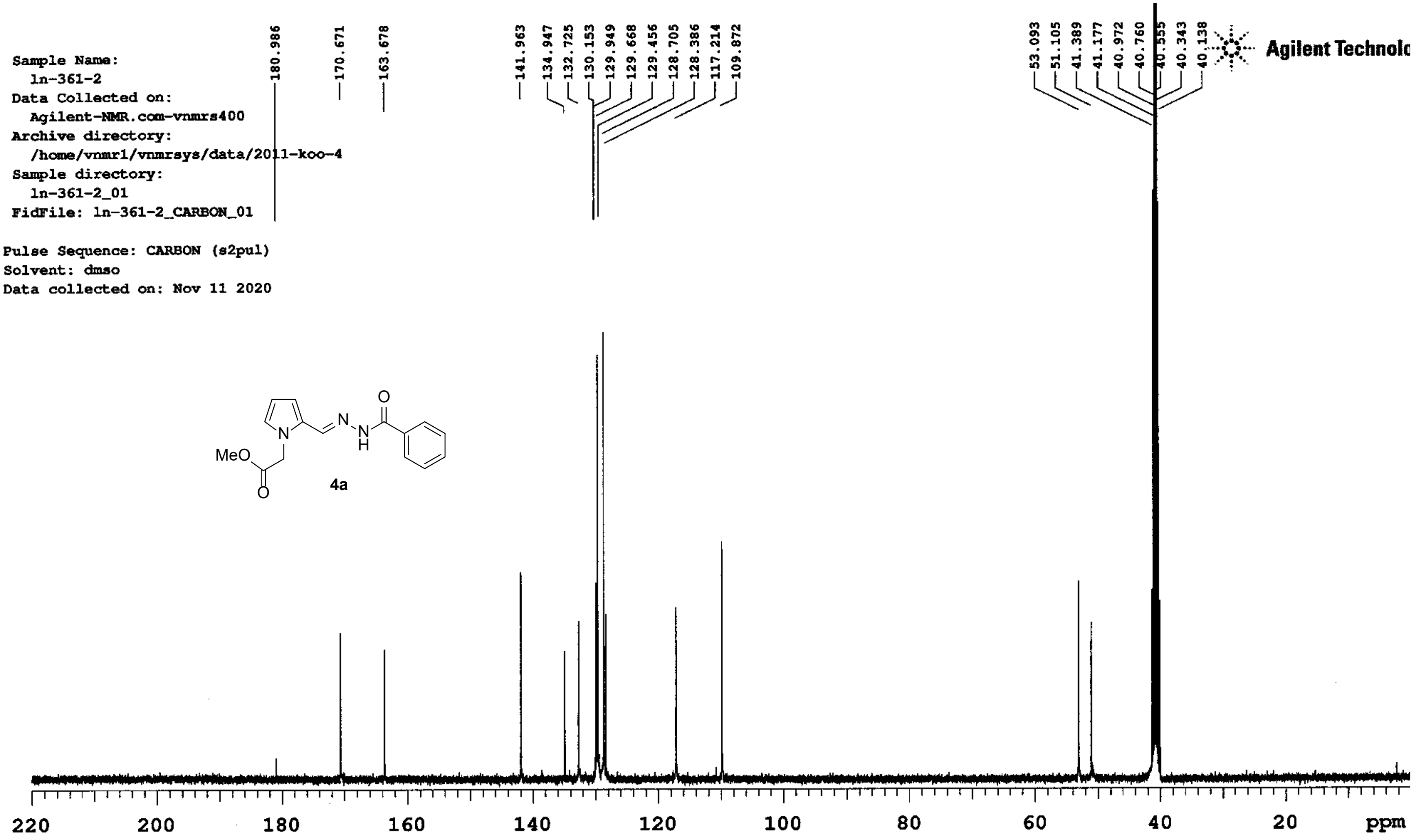
Sample Name:
ln-361-2
Data Collected on:
Agilent-NMR.com-vnmrs400
Archive directory:
/home/vnmr1/vnmrsys/data/2011-koo-4
Sample directory:
ln-361-2_01
FidFile: ln-361-2_CARBON_01
Pulse Sequence: CARBON (s2pul)
Solvent: dmso
Data collected on: Nov 11 2020
180.986
170.671
163.678
141.963
134.947
132.725
130.153
129.949
129.668
129.456
128.705
128.386
117.214
109.872
53.093
51.105
41.389
41.177
40.972
40.760
40.555
40.343
40.138
Agilent Technolo
MeO
O
N
N
N
H
O
4a
220
200
180
160
140
120
100
80
60
40
20
ppm

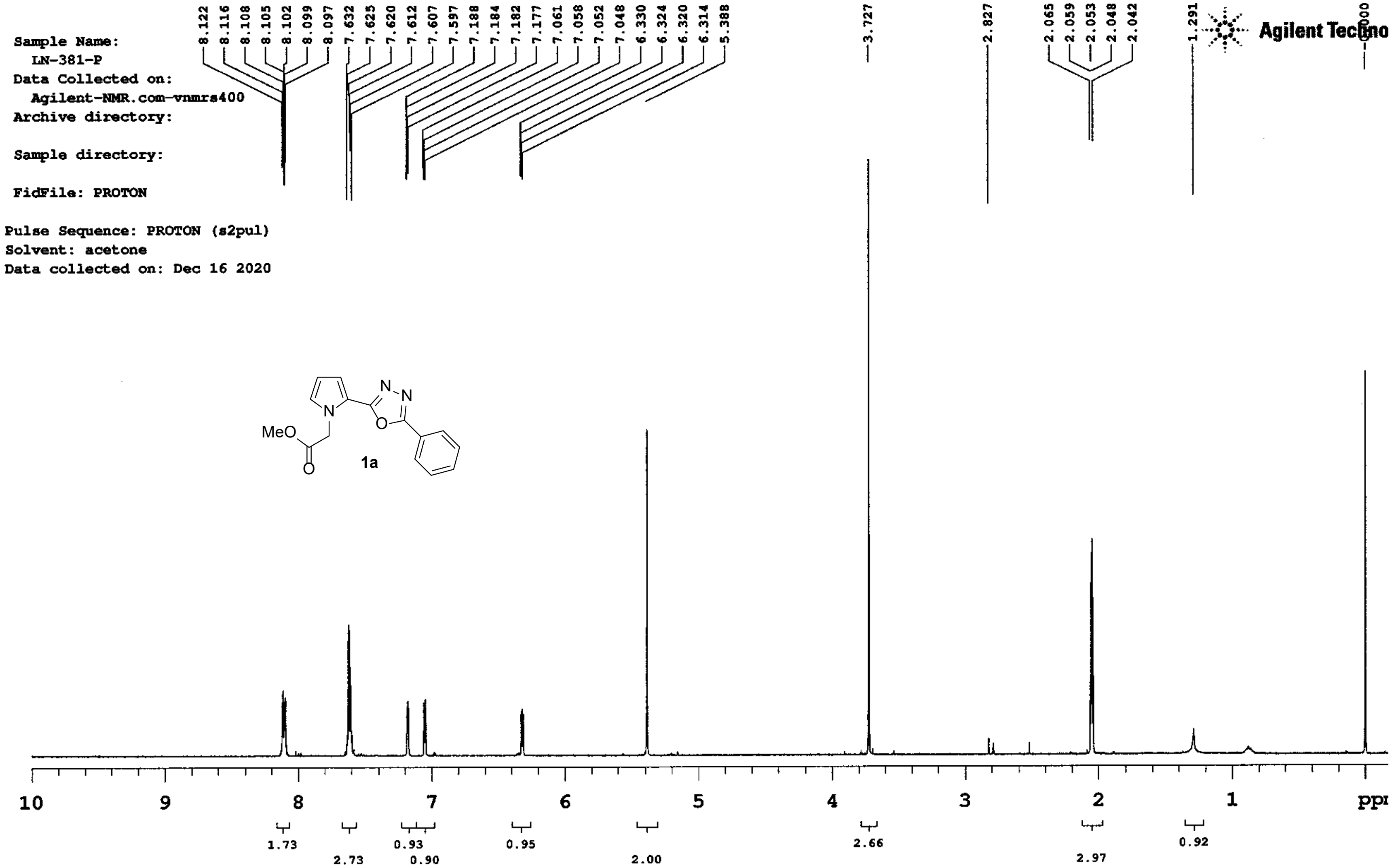
Sample Name:
LN-381-P
Data Collected on:
Agilent-NMR.com-vnmrs400
Archive directory:
Sample directory:
FidFile: PROTON
Pulse Sequence: PROTON (s2pul)
Solvent: acetone
Data collected on: Dec 16 2020
8.122 8.116 8.108 8.105 8.102 8.099 8.097 7.632 7.625 7.620 7.612 7.607 7.597 7.188 7.184 7.182 7.177 7.061 7.058 7.052 7.048 6.330 6.324 6.320 6.314 5.388
3.727
2.827
2.065 2.059 2.053 2.048 2.042
1.291
MeO
N
N
N
O
O
1a
10 9 8 7 6 5 4 3 2 1 ppm
1.73 2.73 0.93 0.90 0.95 2.00 2.66 2.97 0.92

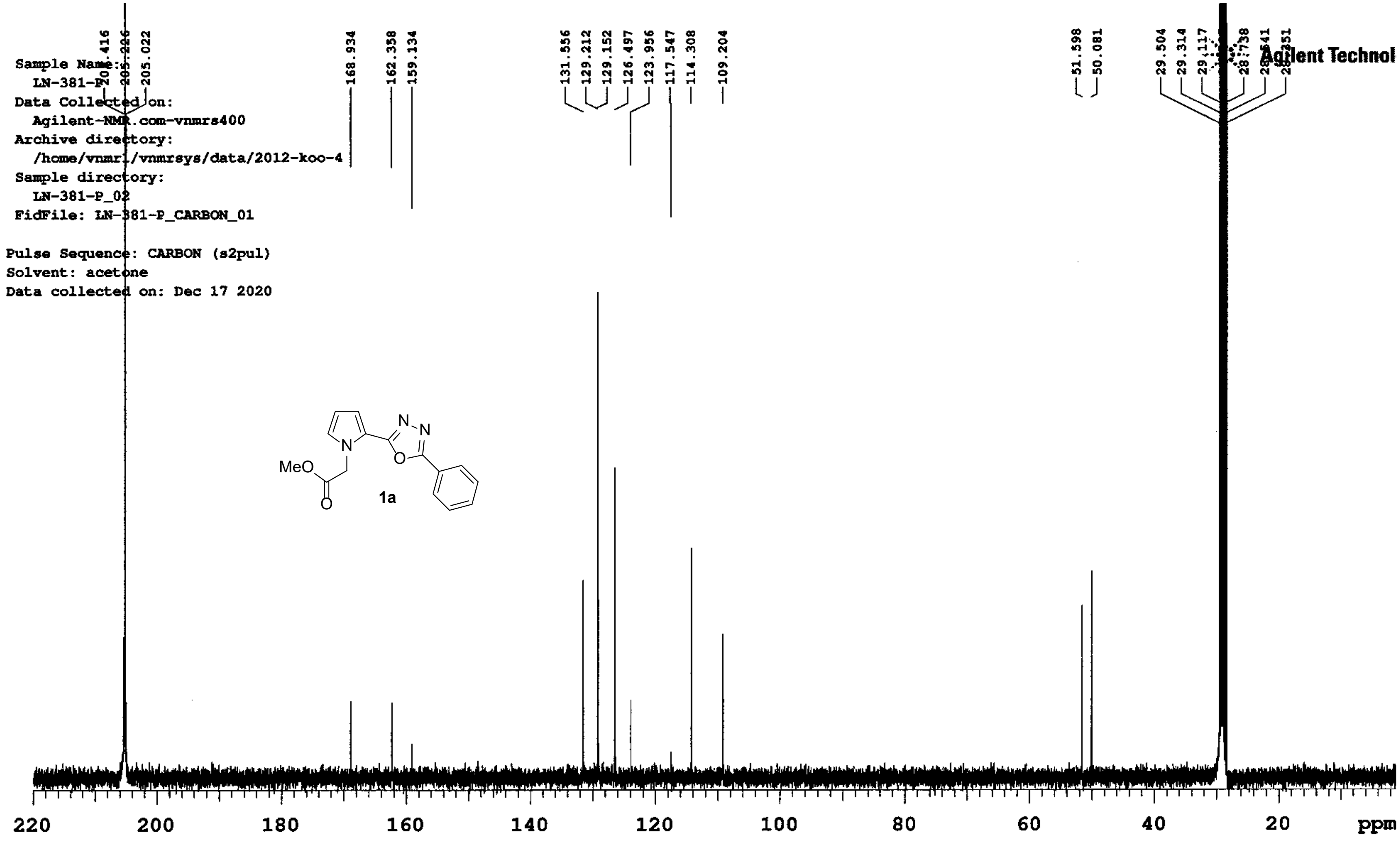

Sample Name:
LN-381-P
Data Collected on:
Agilent-NMR.com-vnmrs400
Archive directory:
/home/vnmr1/vnmrsys/data/2012-koo-4
Sample directory:
LN-381-P_02
FidFile: LN-381-P_CARBON_01
Pulse Sequence: CARBON (s2pul)
Solvent: acetone
Data collected on: Dec 17 2020
168.934
162.358
159.134
131.556
129.212
129.152
126.497
123.956
117.547
114.308
109.204
51.598
50.081
29.504
29.314
29.117
205.022
Agilent Technol
MeO
N
N
N
O
O
1a
220
200
180
160
140
120
100
80
60
40
20
ppm

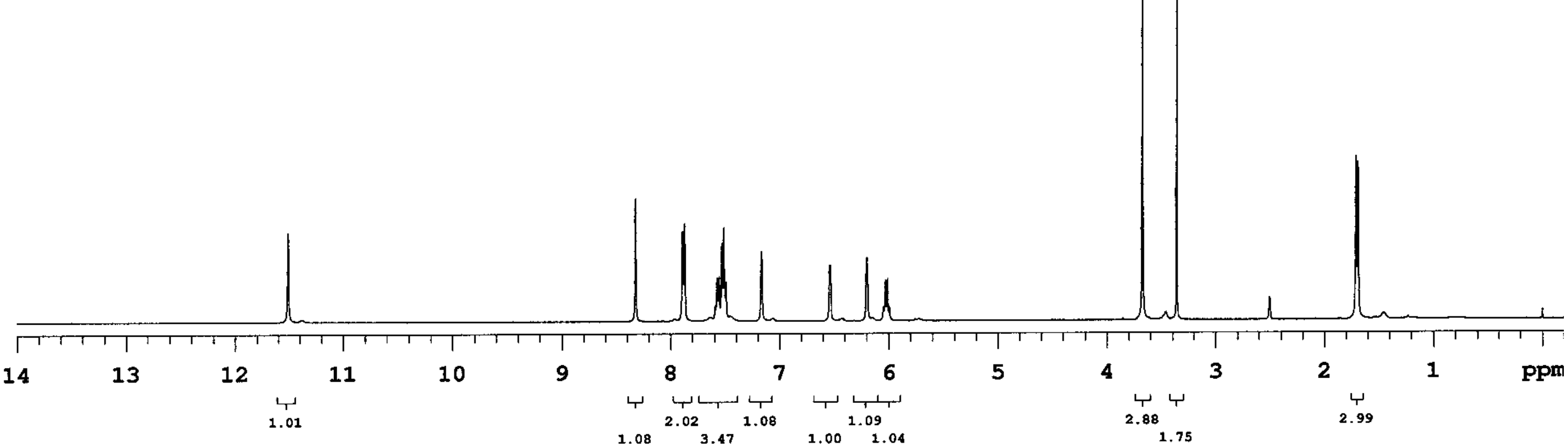
Sample Name:
LN-265-1
Data Collected on:
Agilent-NMR.com-vnmrs400
Archive directory:
/home/vnmr1/vnmrsys/data/2012-koo-4
Sample directory:
LN-265-1_01
FidFile: LN-265-1_PROTON_01
Pulse Sequence: PROTON (s2pul)
Solvent: dmso
Data collected on: Dec 4 2020
Agilent Technol
11.513
8.322
7.892
7.872
7.594
7.576
7.558
7.533
7.514
7.496
7.168
6.541
6.537
6.532
6.204
6.197
6.189
6.047
6.030
6.011
5.993
3.673
3.356
2.508
2.503
2.499
1.710
14 13 12 11 10 9 8 7 6 5 4 3 2 1 ppm
1.01
1.08
2.02
3.47
1.08
1.00
1.09
1.04
2.88
1.75
2.99

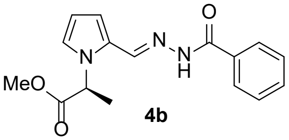
MeO
N
N
N
H
O
O

4b

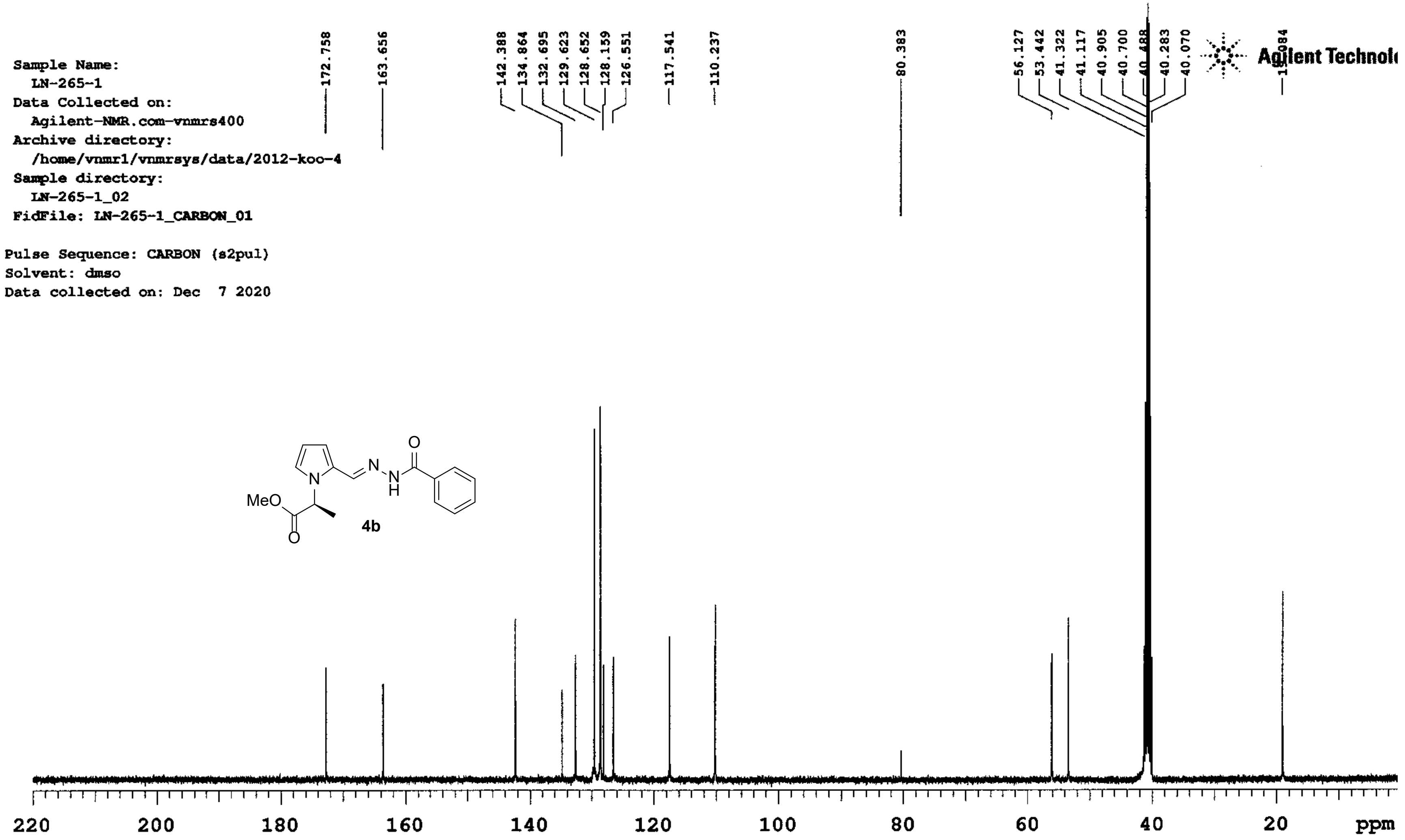
Sample Name:
LN-265-1
Data Collected on:
Agilent-NMR.com-vnmrs400
Archive directory:
/home/vnmr1/vnmrsys/data/2012-koo-4
Sample directory:
LN-265-1_02
FidFile: LN-265-1_CARBON_01
Pulse Sequence: CARBON (s2pul)
Solvent: dmso
Data collected on: Dec 7 2020
172.758
163.656
142.388
134.864
132.695
129.623
128.652
128.159
126.551
117.541
110.237
80.383
56.127
53.442
41.322
41.117
40.905
40.700
40.488
40.283
40.070
Agilent Technol
MeO
O
N
N
N
H
O
4b
220
200
180
160
140
120
100
80
60
40
20
ppm

Sample Name:
LN-279-P
Data Collected on:
Agilent-NMR.com-vnmrs400
Archive directory:
/home/vnmr1/vnmrsys/data/2008-koo-4
Sample directory:
LN-279-P_01
FidFile: LN-279-P_PROTON_01

Pulse Sequence: PROTON (s2pul)
Solvent: cd3od
Data collected on: Aug 6 2020

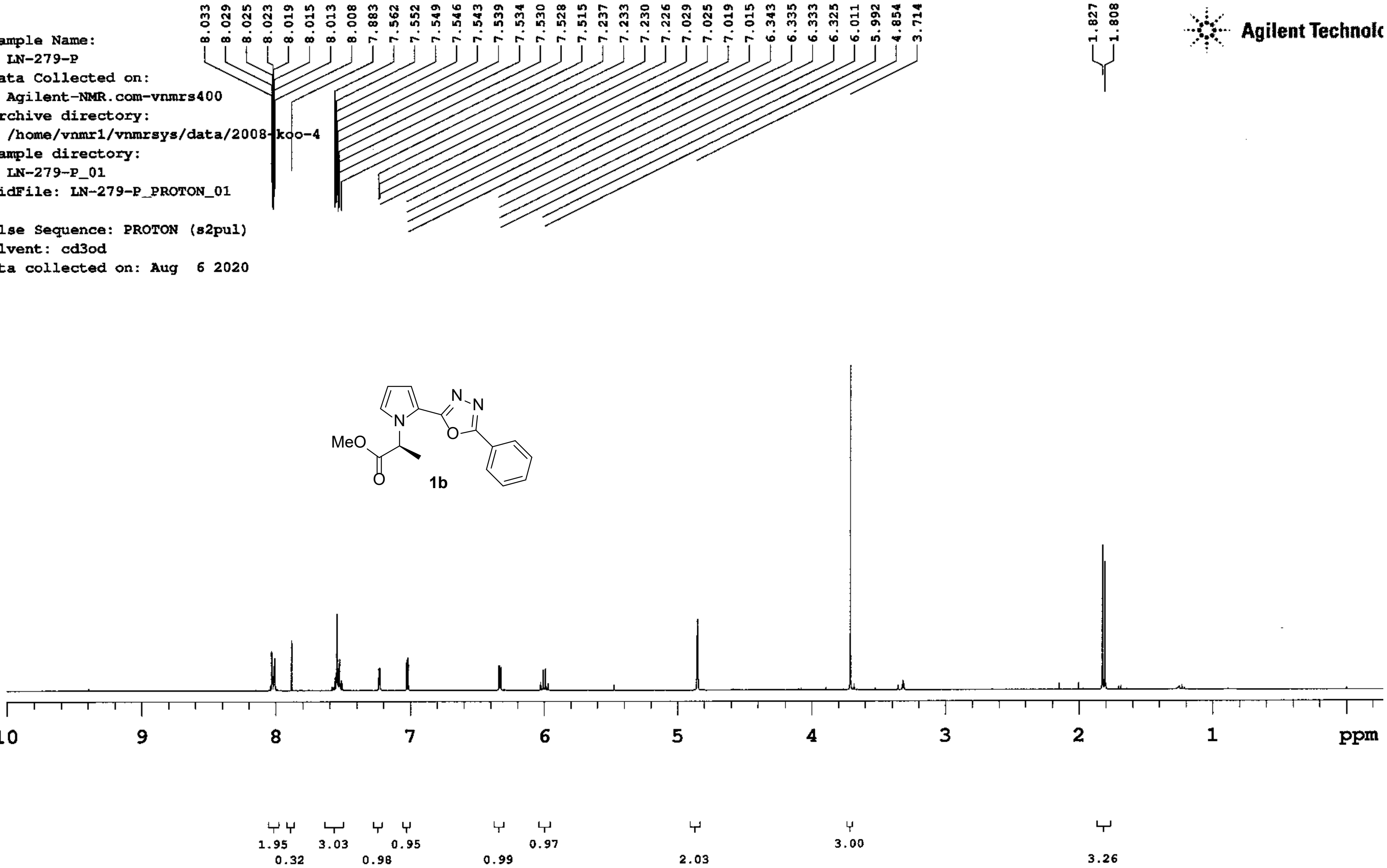

1b

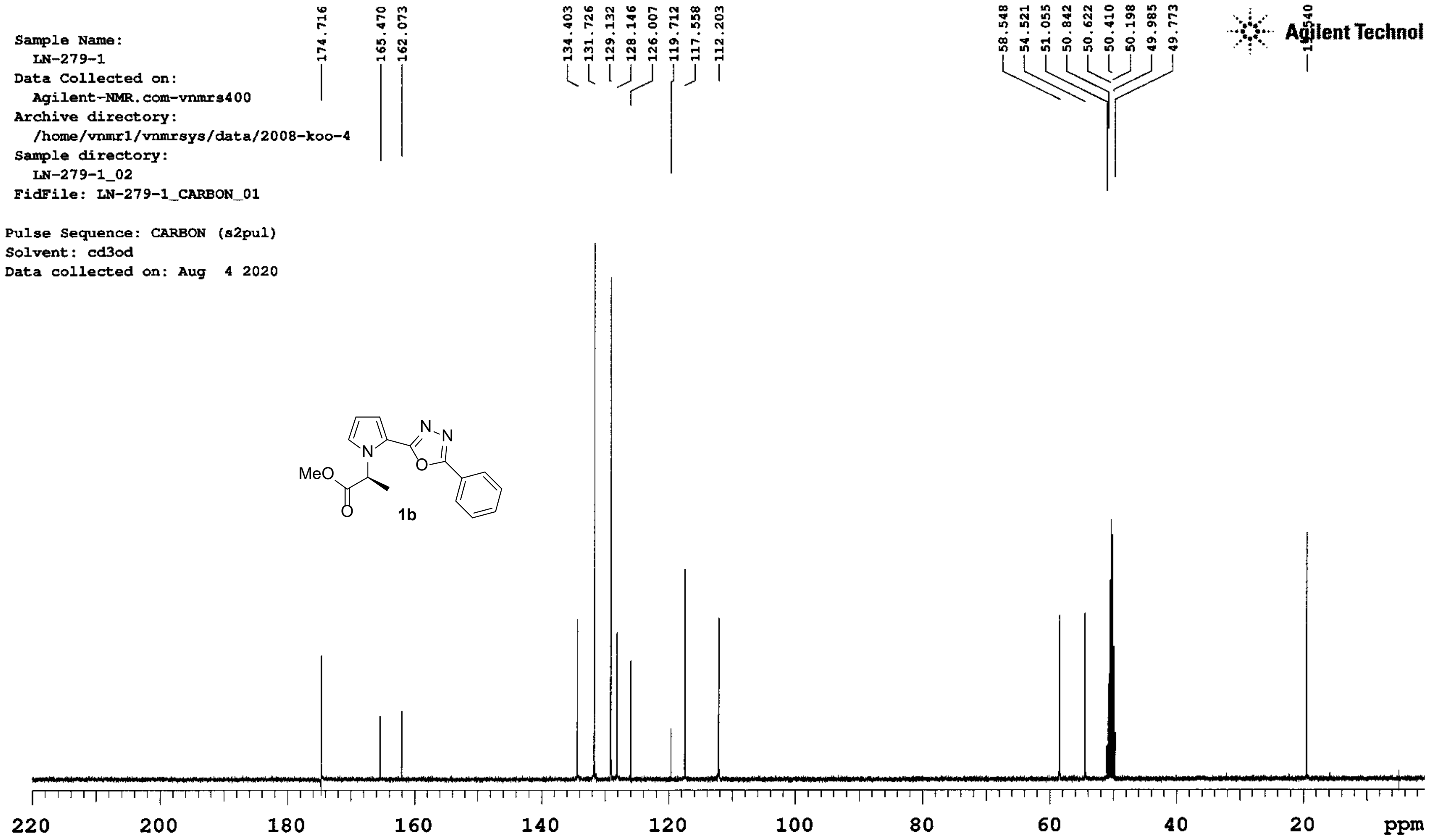
Sample Name:
LN-279-1
Data Collected on:
Agilent-NMR.com-vnmrs400
Archive directory:
/home/vnmr1/vnmrsys/data/2008-koo-4
Sample directory:
LN-279-1_02
FidFile: LN-279-1_CARBON_01
Pulse Sequence: CARBON (s2pul)
Solvent: cd3od
Data collected on: Aug 4 2020
174.716
165.470
162.073
134.403
131.726
129.132
128.146
126.007
119.712
117.558
112.203
58.548
54.521
51.055
50.842
50.622
50.410
50.198
49.985
49.773
Agilent Technol
MeO
O
N
N
N
O
1b
220
200
180
160
140
120
100
80
60
40
20
ppm

Sample Name:
LN-213-2
Data Collected on:
Agilent-NMR.com-vnmrs400
Archive directory:
/home/vnmr1/vnmrsys/data/2005-koo-4
Sample directory:
LN-213-2_01
FidFile: LN-213-2_PROTON_01

Pulse Sequence: PROTON (s2pul)
Solvent: dmso
Data collected on: May 19 2020

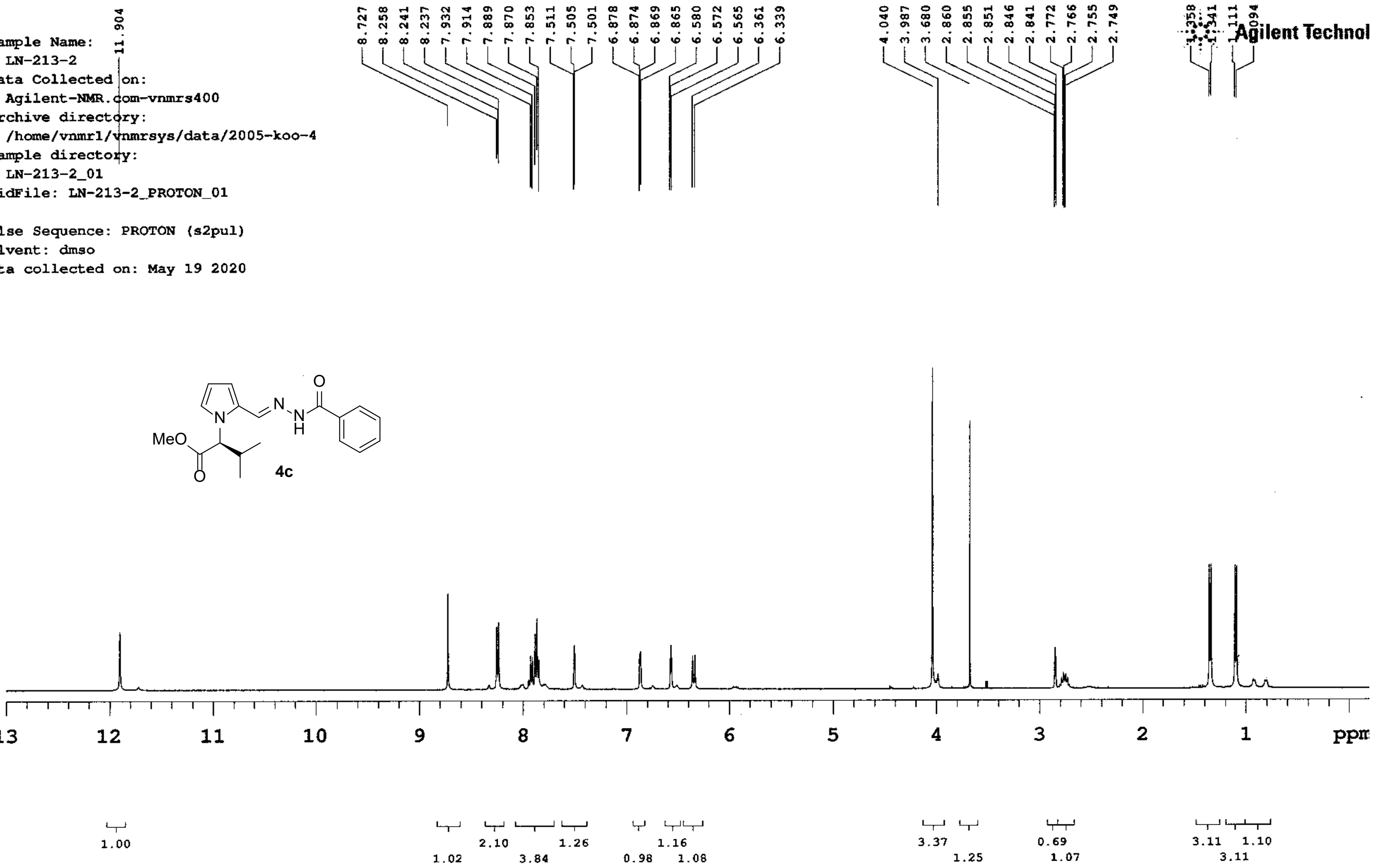

4c

Sample Name:
LN-213-2
Data Collected on:
Agilent-NMR.com-vnmrs400
Archive directory:
/home/vnmr1/vnmrsys/data/2005-koo-4
Sample directory:
LN-213-2_03
FidFile: LN-213-2_CARBON_01

Pulse Sequence: CARBON (s2pul)
Solvent: dmso
Data collected on: May 28 2020

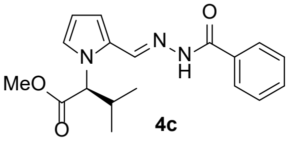

4c

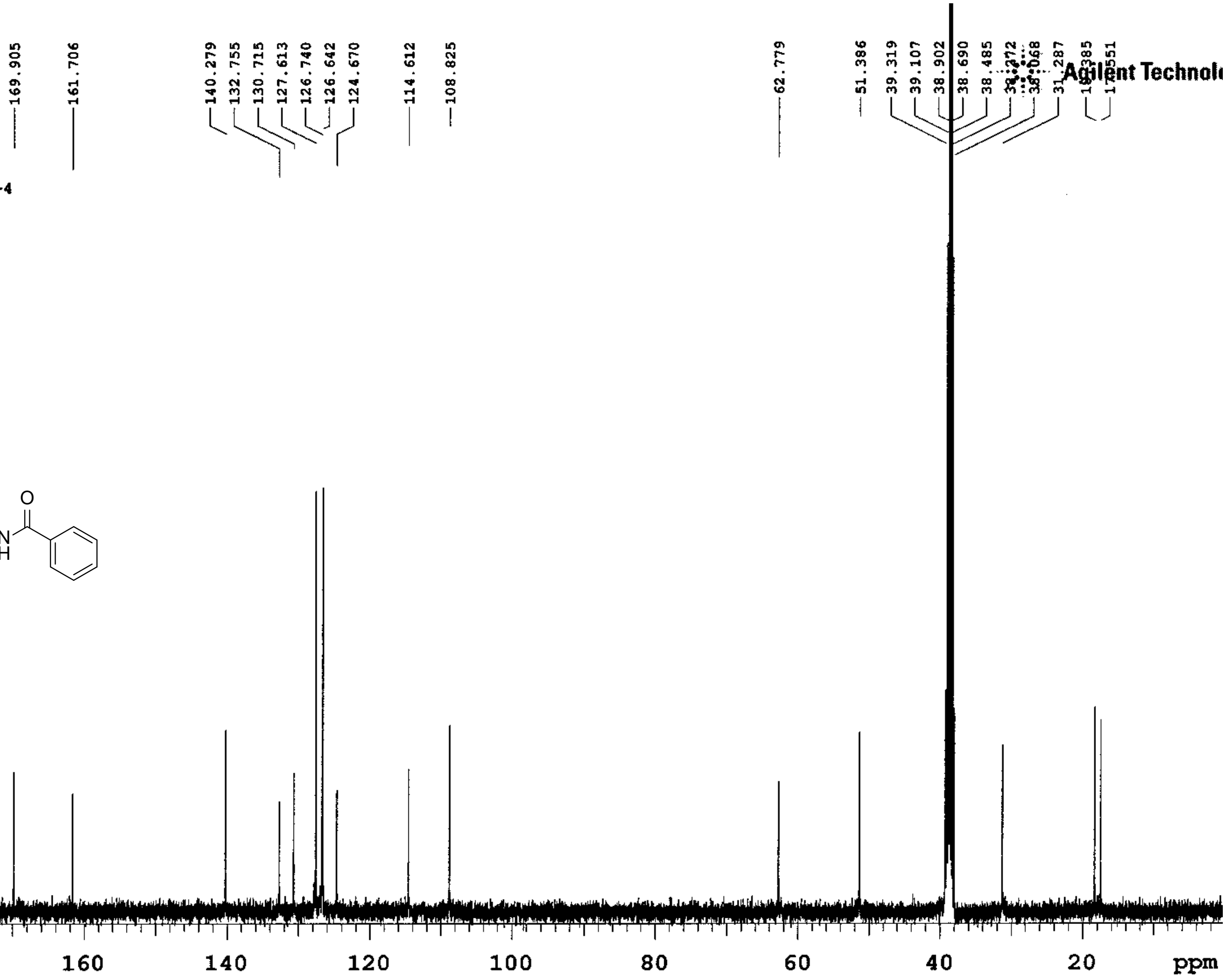

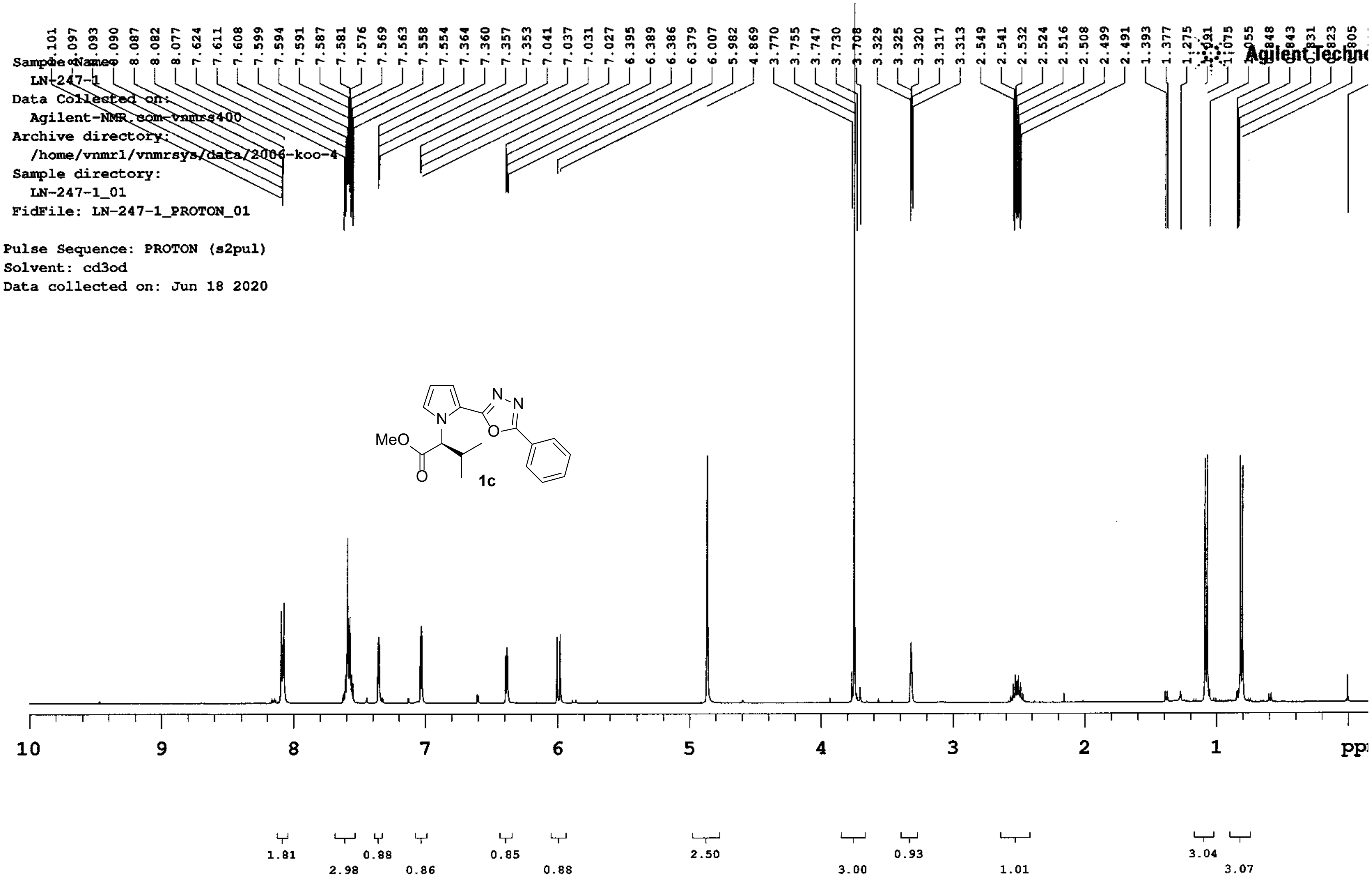
Sample Name:
LN-247-1
Data Collected on:
Agilent-NMR.com-vnmrs400
Archive directory:
/home/vnmr1/vnmrsys/data/2006-koo-4
Sample directory:
LN-247-1_01
FidFile: LN-247-1_PROTON_01
Pulse Sequence: PROTON (s2pul)
Solvent: cd3od
Data collected on: Jun 18 2020
MeO
N
O
1c
10
9
8
7
6
5
4
3
2
1
ppm
1.81
2.98
0.88
0.86
0.85
0.88
2.50
3.00
0.93
1.01
3.04
3.07

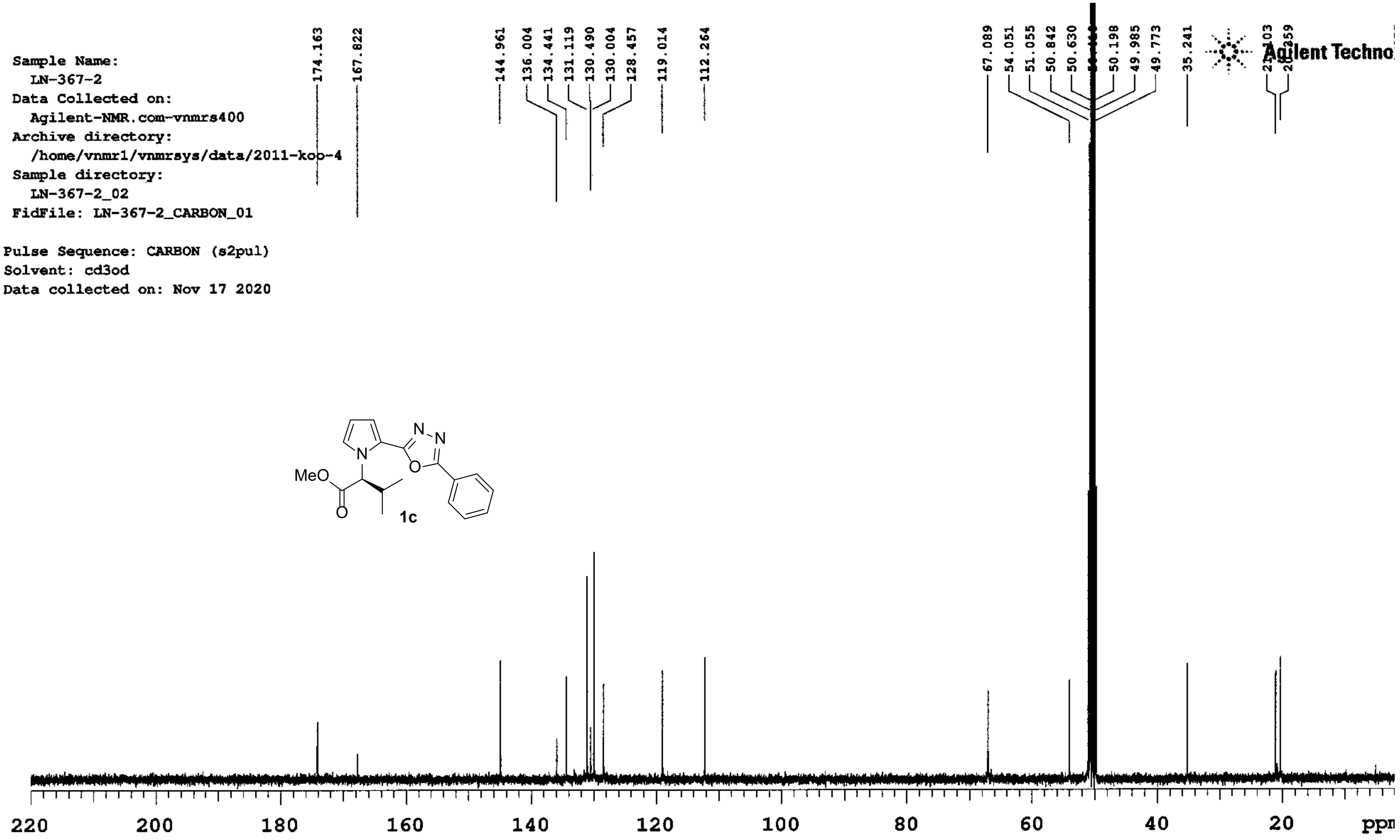

Sample Name:
LN-367-2
Data Collected on:
Agilent-NMR.com-vnmrs400
Archive directory:
/home/vnmr1/vnmrsys/data/2011-koo-4
Sample directory:
LN-367-2_02
FidFile: LN-367-2_CARBON_01
Pulse Sequence: CARBON (s2pul)
Solvent: cd3od
Data collected on: Nov 17 2020
174.163
167.822
144.961
136.004
134.441
131.119
130.490
130.004
128.457
119.014
112.264
67.089
54.051
51.055
50.842
50.630
50.198
49.985
49.773
35.241
Agilent Techno
MeO
O
N
N
N
O
1c
220
200
180
160
140
120
100
80
60
40
20
ppm

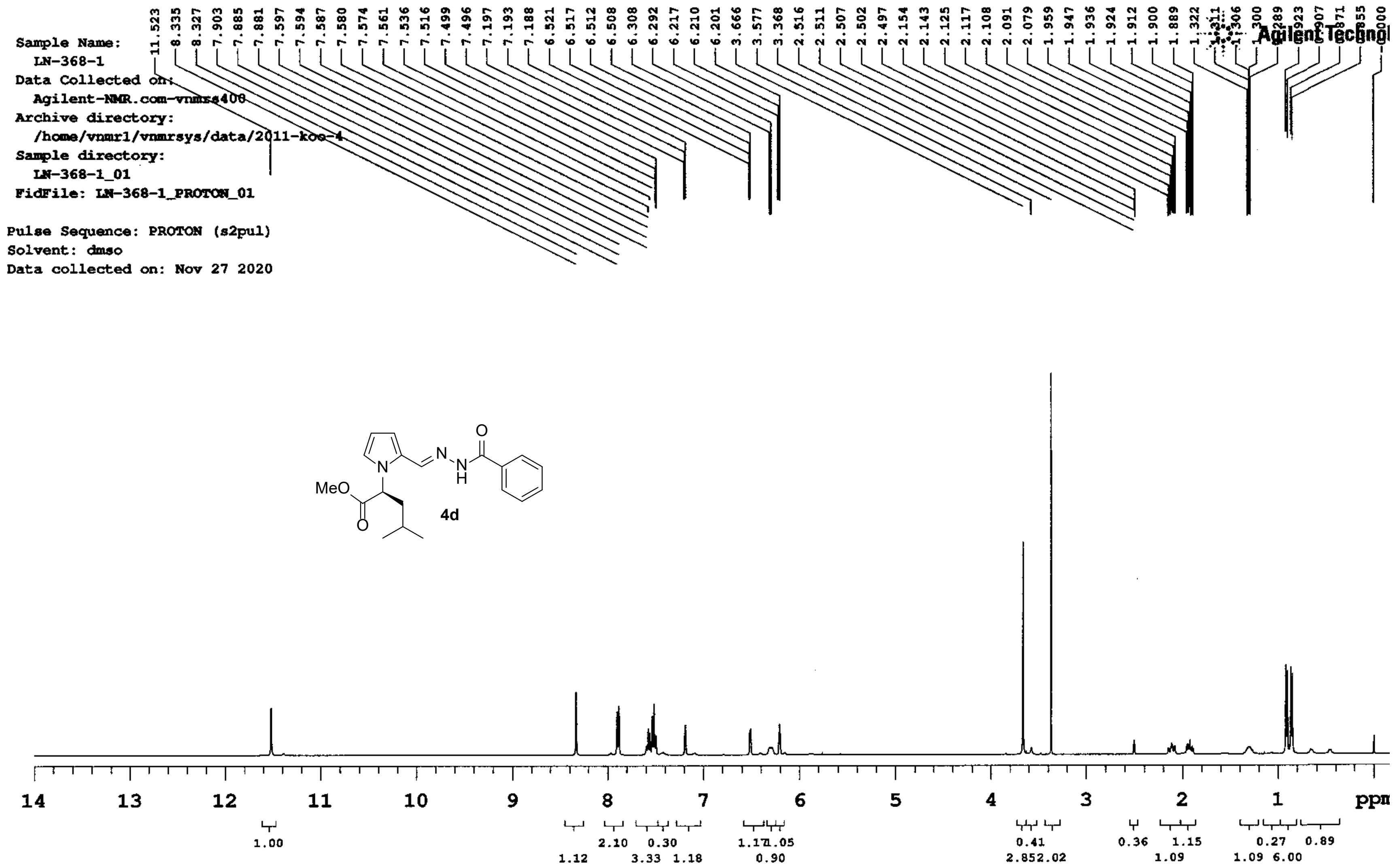
Sample Name:
LN-368-1
Data Collected on:
Agilent-NMR.com-vnmrs400
Archive directory:
/home/vnmr1/vnmrsys/data/2011-koo-4
Sample directory:
LN-368-1_01
FidFile: LN-368-1_PROTON_01
Pulse Sequence: PROTON (s2pul)
Solvent: dmso
Data collected on: Nov 27 2020
11.523 8.335 8.327 7.903 7.885 7.881 7.597 7.594 7.587 7.580 7.574 7.561 7.536 7.516 7.499 7.496 7.197 7.193 7.188 6.521 6.517 6.512 6.508 6.308 6.292 6.217 6.210 6.201 3.666 3.577 3.368 2.516 2.511 2.507 2.502 2.497 2.154 2.143 2.125 2.117 2.108 2.091 2.079 1.959 1.947 1.936 1.924 1.912 1.900 1.889 1.322 1.300 1.289 0.923 0.907 0.871 0.000
Agilent Technologies
MeO
N
N
N
H
O
O
4d
14 13 12 11 10 9 8 7 6 5 4 3 2 1 ppm
1.00 1.12 2.10 3.33 0.30 1.18 1.17 1.05 0.90 0.41 2.85 2.02 0.36 1.15 1.09 1.09 0.27 6.00 0.89

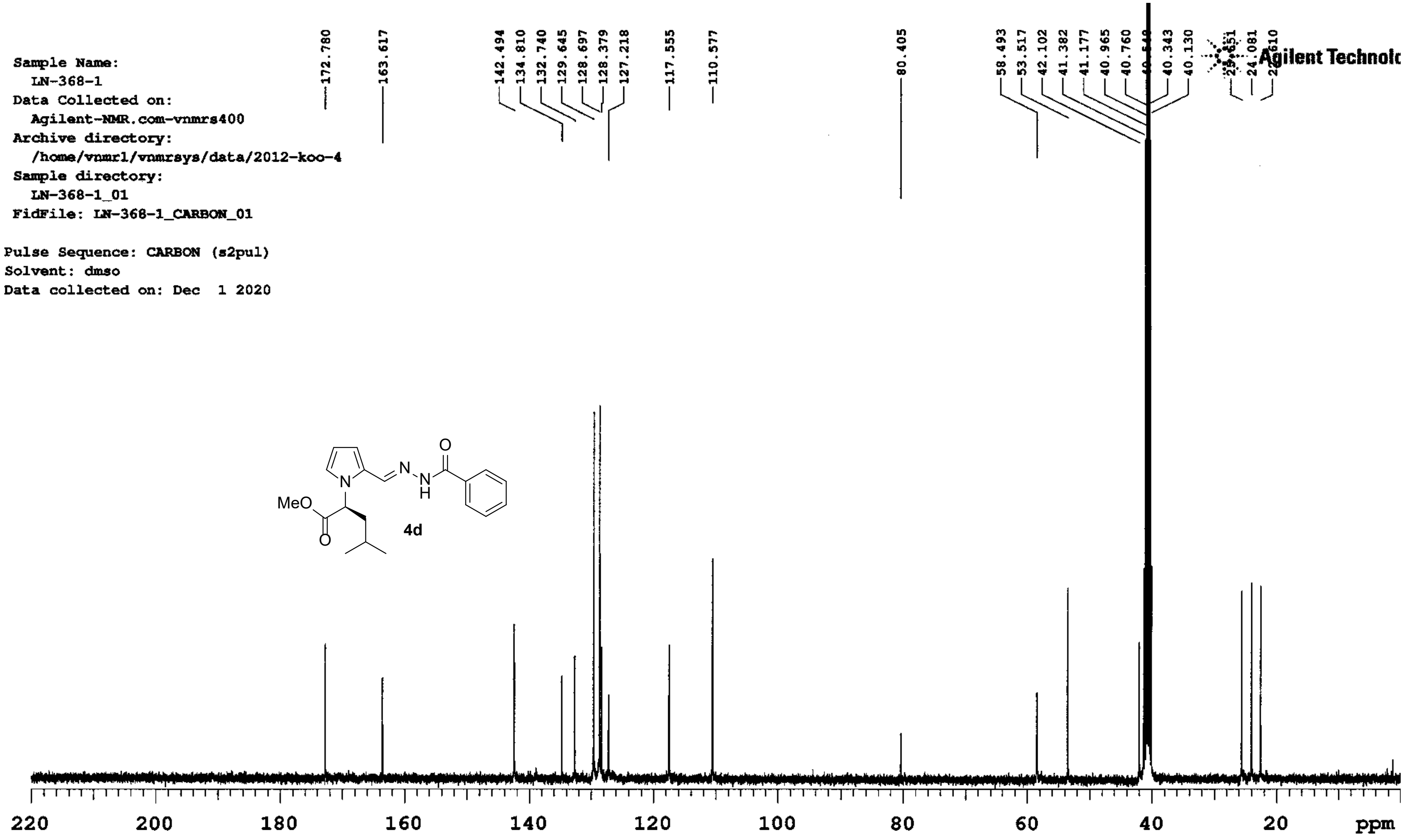
Sample Name:
LN-368-1
Data Collected on:
Agilent-NMR.com-vnmrs400
Archive directory:
/home/vnmr1/vnmrsys/data/2012-koo-4
Sample directory:
LN-368-1_01
FidFile: LN-368-1_CARBON_01
Pulse Sequence: CARBON (s2pul)
Solvent: dmso
Data collected on: Dec 1 2020
172.780
163.617
142.494
134.810
132.740
129.645
128.697
128.379
127.218
117.555
110.577
80.405
58.493
53.517
42.102
41.382
41.177
40.965
40.760
40.343
40.130
24.081
Agilent Technol
MeO
N
N
N
H
O
O
4d
220
200
180
160
140
120
100
80
60
40
20
ppm

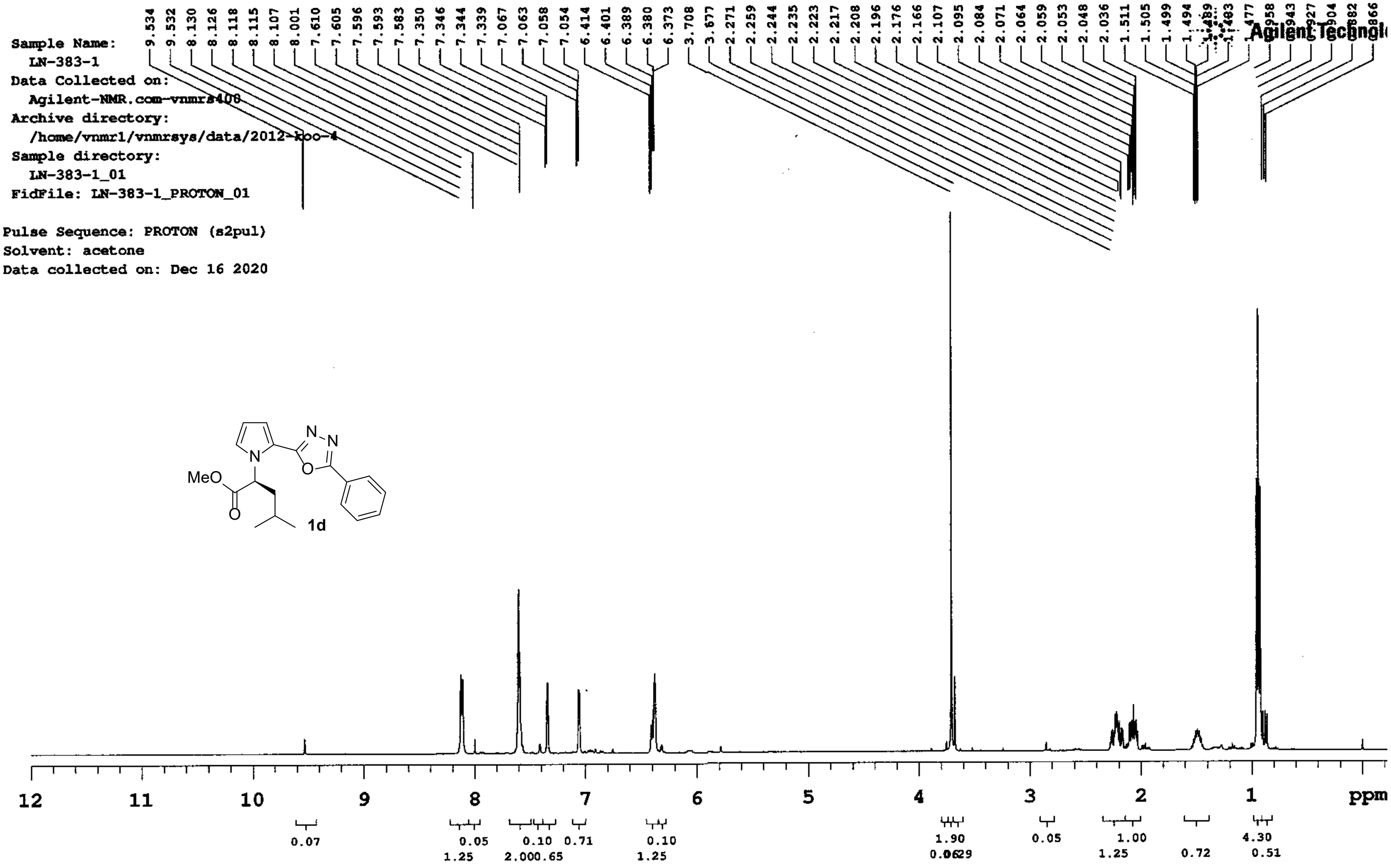
Sample Name:
LN-383-1
Data Collected on:
Agilent-NMR.com-vnmrs400
Archive directory:
/home/vnmr1/vnmrsys/data/2012-koo-4
Sample directory:
LN-383-1_01
FidFile: LN-383-1_PROTON_01
Pulse Sequence: PROTON (s2pul)
Solvent: acetone
Data collected on: Dec 16 2020
Agilent Technologies
9.534 9.532 8.130 8.126 8.118 8.115 8.107 8.001 7.610 7.605 7.596 7.593 7.583 7.350 7.346 7.344 7.339 7.067 7.063 7.058 7.054 6.414 6.401 6.389 6.380 6.373 3.708 3.677 2.271 2.259 2.244 2.235 2.223 2.217 2.208 2.196 2.176 2.166 2.107 2.095 2.084 2.071 2.064 2.059 2.053 2.048 2.036 1.511 1.505 1.499 1.494 1.477 0.958 0.943 0.927 0.904 0.882 0.866
MeO
N
N
N
O
O
1d
12 11 10 9 8 7 6 5 4 3 2 1 ppm
0.07
0.05
1.25
0.10
2.00
0.65
0.71
0.10
1.25
1.90
0.05
1.00
1.25
0.72
4.30
0.51

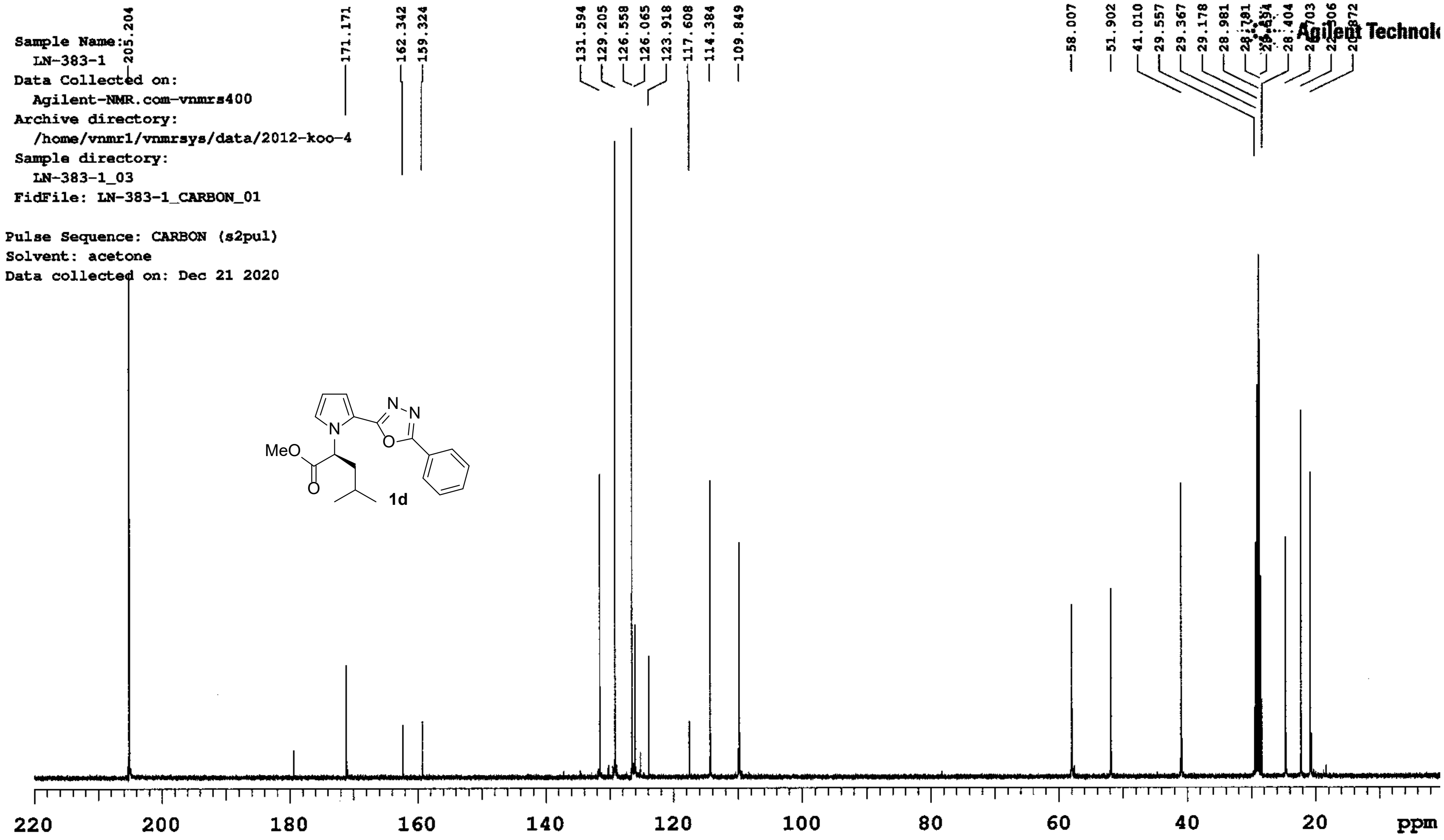
Sample Name:
LN-383-1
Data Collected on:
Agilent-NMR.com-vnmrs400
Archive directory:
/home/vnmr1/vnmrsys/data/2012-koo-4
Sample directory:
LN-383-1_03
FidFile: LN-383-1_CARBON_01
Pulse Sequence: CARBON (s2pul)
Solvent: acetone
Data collected on: Dec 21 2020
205.204
171.171
162.342
159.324
131.594
129.205
126.558
126.065
123.918
117.608
114.384
109.849
58.007
51.902
41.010
29.557
29.367
29.178
28.981
28.404
MeO
O
N
N
N
O
1d
220
200
180
160
140
120
100
80
60
40
20
ppm

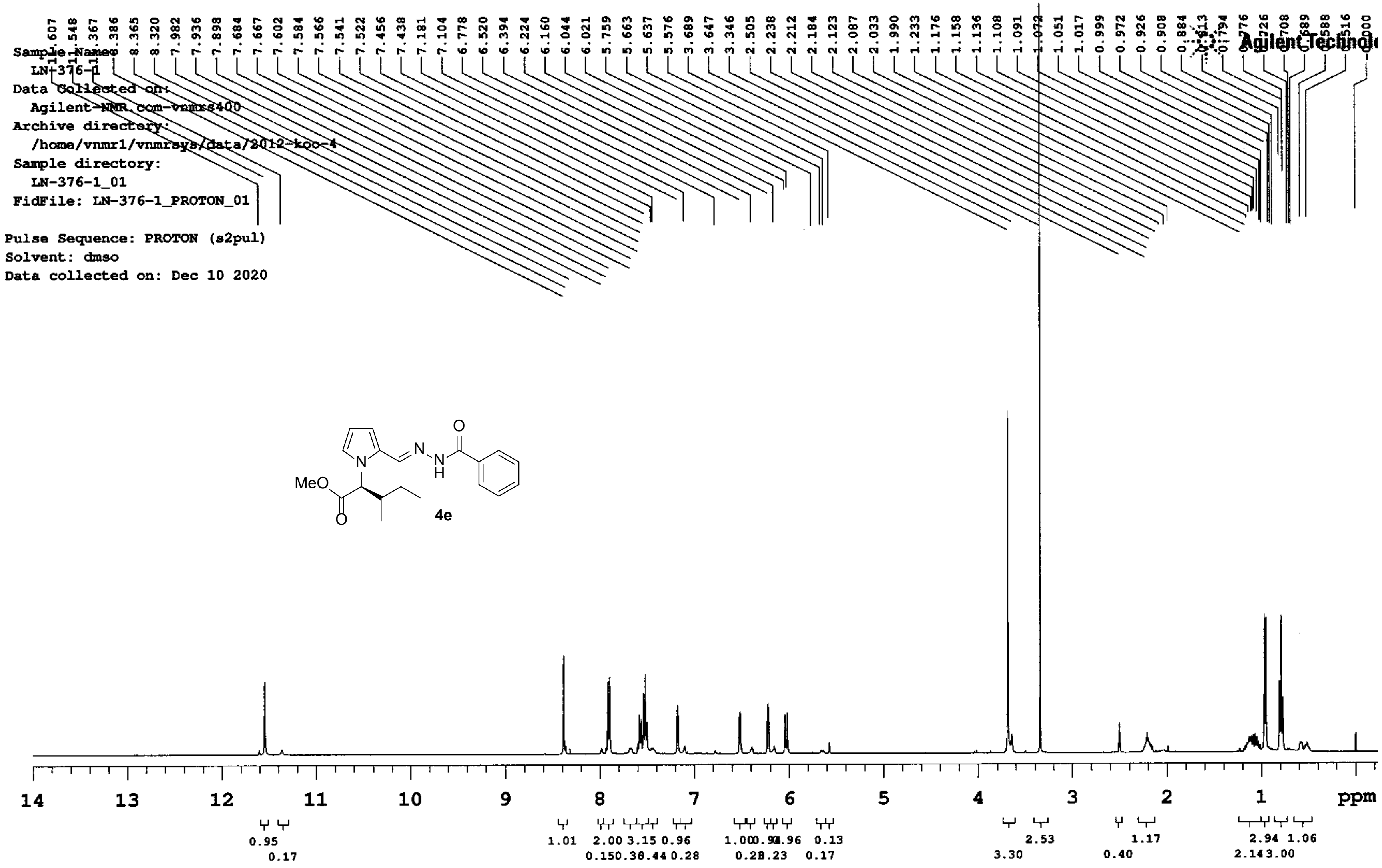
Sample Name:
LN-376-1
Data Collected on:
Agilent-NMR.com-vnmrs400
Archive directory:
/home/vnmr1/vnmrsys/data/2012-koo-4
Sample directory:
LN-376-1_01
FidFile: LN-376-1_PROTON_01
Pulse Sequence: PROTON (s2pul)
Solvent: dmso
Data collected on: Dec 10 2020
Agilent Technologies
11.607 11.548 11.367 8.386 8.365 8.320 7.982 7.936 7.898 7.684 7.667 7.602 7.584 7.566 7.541 7.522 7.456 7.438 7.181 7.104 6.778 6.520 6.394 6.224 6.160 6.044 6.021 5.759 5.663 5.637 5.576 3.689 3.647 3.346 2.505 2.238 2.212 2.184 2.123 2.087 2.033 1.990 1.233 1.176 1.158 1.136 1.108 1.091 1.072 1.051 1.017 0.999 0.972 0.926 0.908 0.884 0.794 0.776 0.726 0.708 0.689 0.588 0.516 0.000
MeO
N
O
H
4e
14 13 12 11 10 9 8 7 6 5 4 3 2 1 ppm
0.95 0.17 1.01 2.00 0.15 0.30 3.15 0.44 0.96 0.28 1.00 0.28 0.23 0.96 0.13 0.17 3.30 2.53 0.40 1.17 2.14 2.94 3.00 1.06

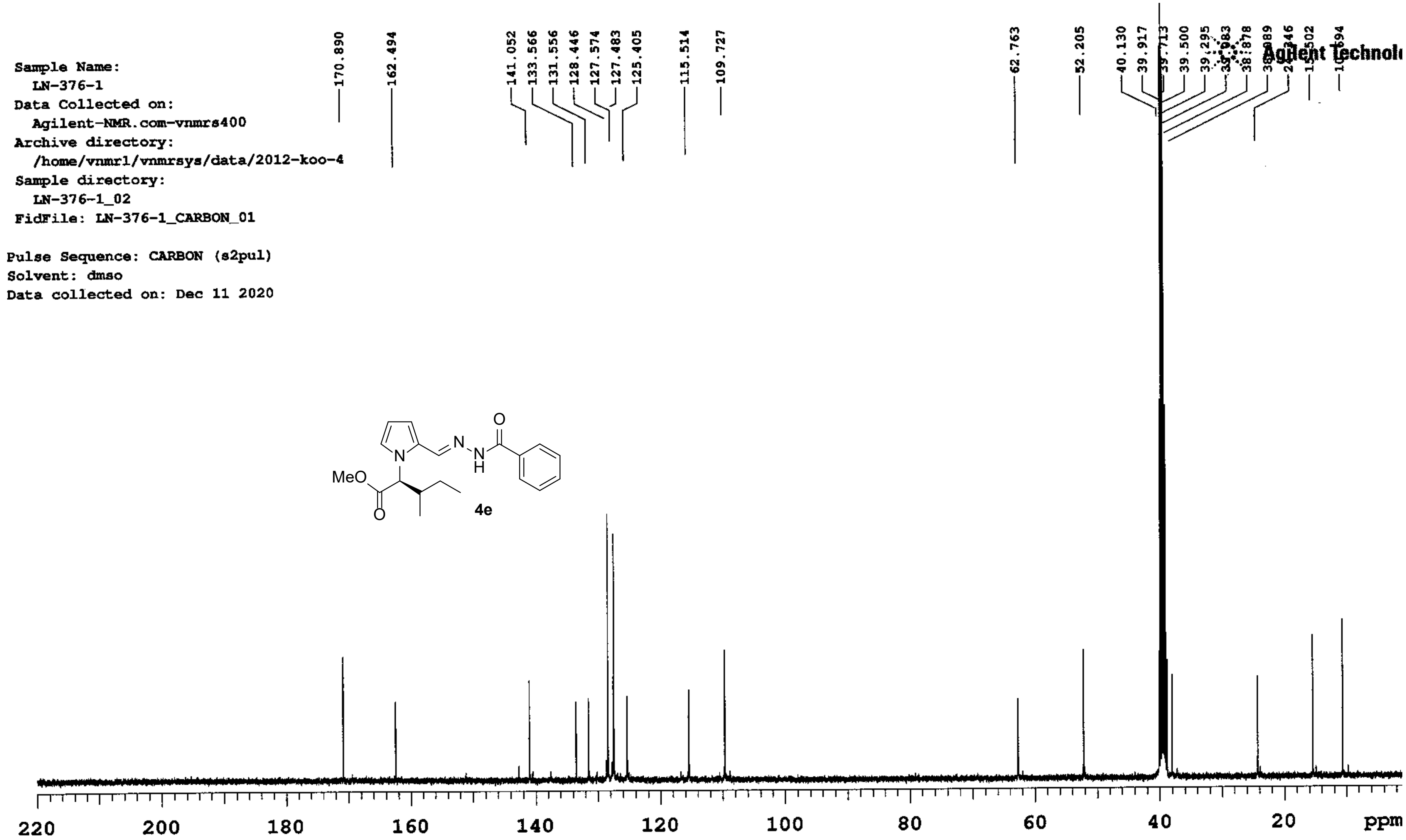
Sample Name:
LN-376-1
Data Collected on:
Agilent-NMR.com-vnmrs400
Archive directory:
/home/vnmr1/vnmrsys/data/2012-koo-4
Sample directory:
LN-376-1_02
FidFile: LN-376-1_CARBON_01
Pulse Sequence: CARBON (s2pul)
Solvent: dmso
Data collected on: Dec 11 2020
170.890
162.494
141.052
133.566
131.556
128.446
127.574
127.483
125.405
115.514
109.727
62.763
52.205
40.130
39.917
39.713
39.500
39.295
MeO
N
N
N
H
O
O
4e
220
200
180
160
140
120
100
80
60
40
20
ppm

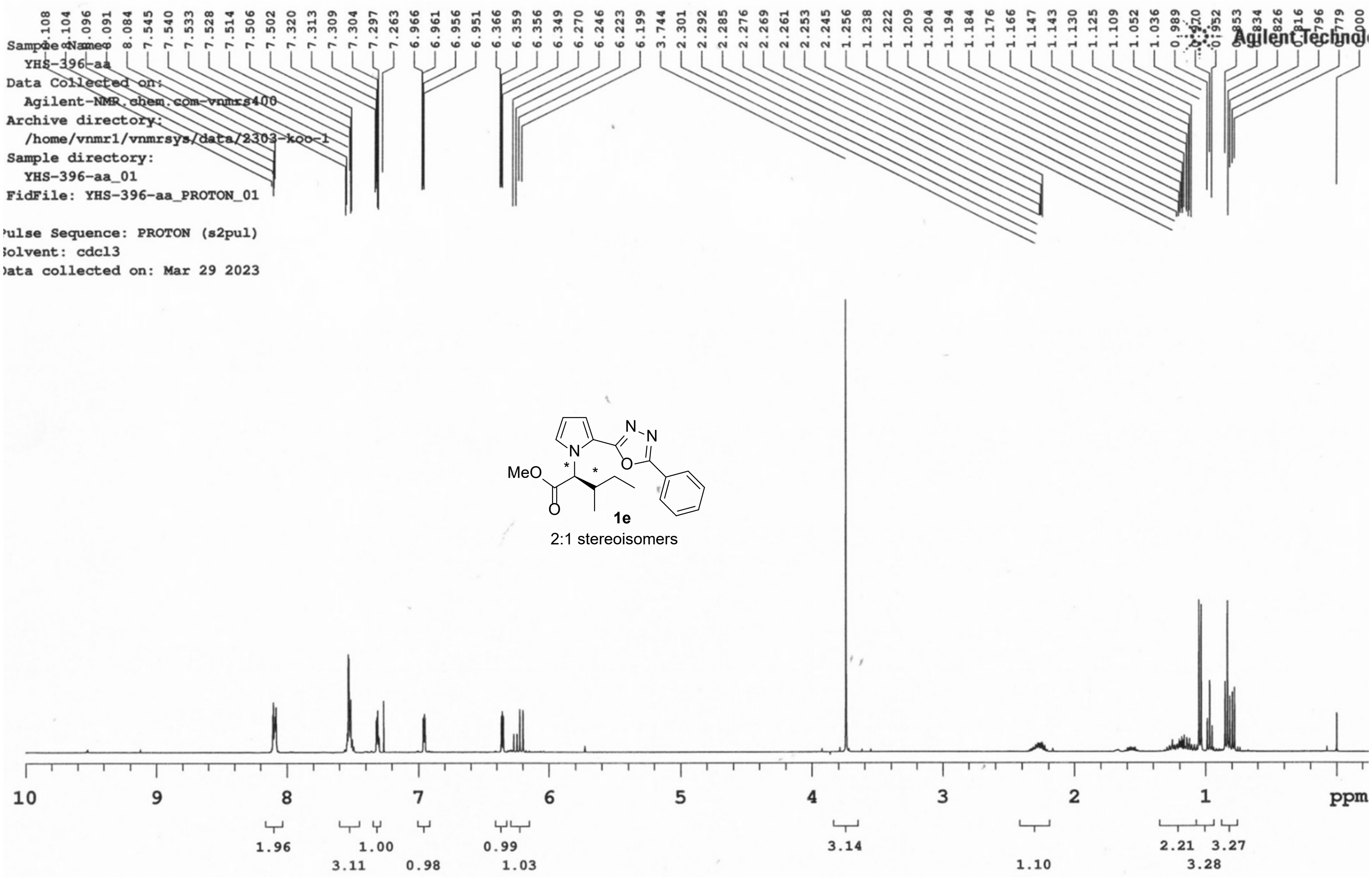

Sample Name:
YHS-396-aa
Data Collected on:
Agilent-NMR.chem.com-vnmrs400
Archive directory:
/home/vnmr1/vnmrsys/data/2303-koo-1
Sample directory:
YHS-396-aa_01
FidFile: YHS-396-aa_PROTON_01
Pulse Sequence: PROTON (s2pul)
Solvent: cdcl3
Data collected on: Mar 29 2023
MeO
N
O
1e
2:1 stereoisomers
ppm

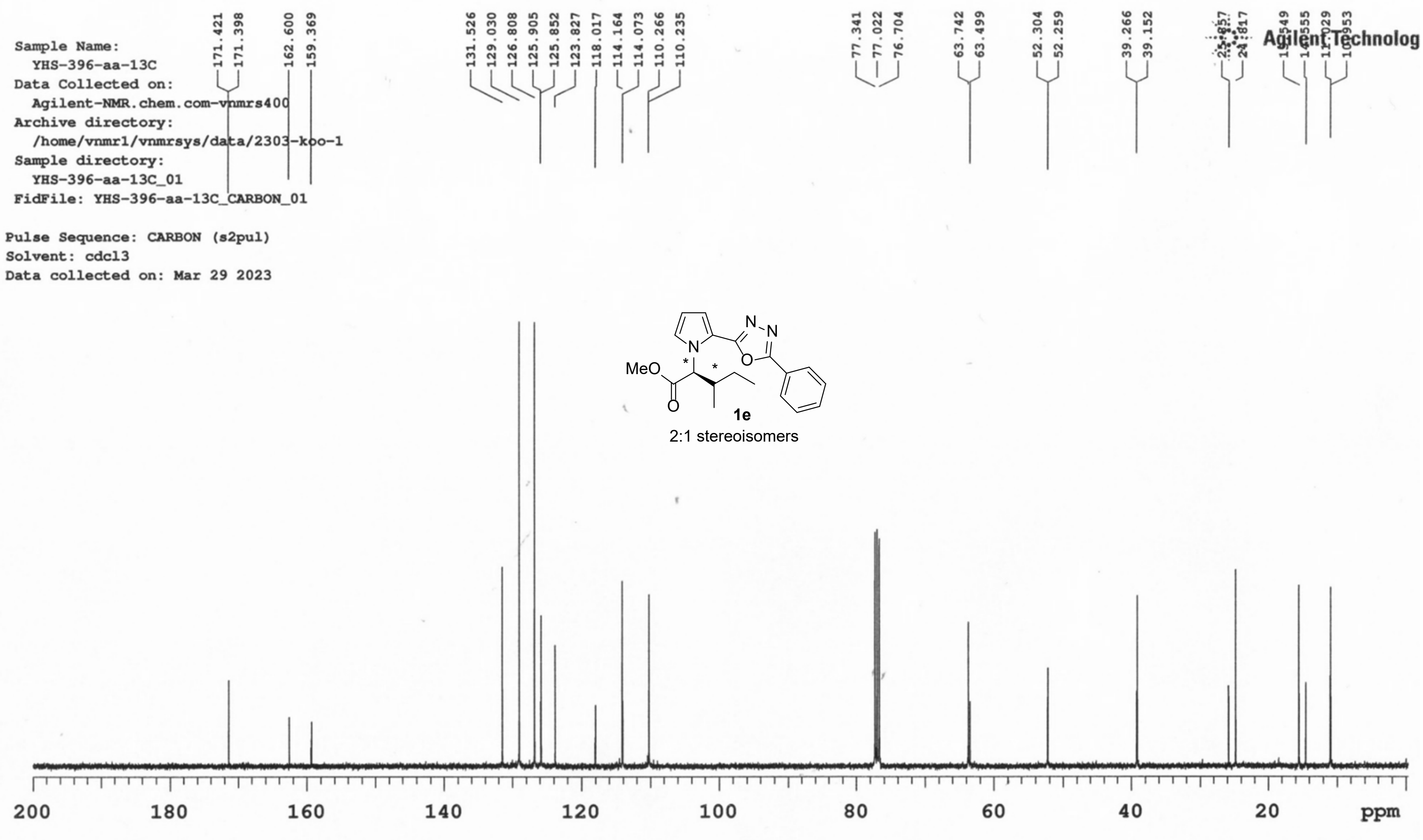
Sample Name:
YHS-396-aa-13C
Data Collected on:
Agilent-NMR.chem.com-vnmrs400
Archive directory:
/home/vnmr1/vnmrsys/data/2303-koo-1
Sample directory:
YHS-396-aa-13C_01
FidFile: YHS-396-aa-13C_CARBON_01
Pulse Sequence: CARBON (s2pul)
Solvent: cdcl3
Data collected on: Mar 29 2023
171.421
171.398
162.600
159.369
131.526
129.030
126.808
125.905
125.852
123.827
118.017
114.164
114.073
110.266
110.235
77.341
77.022
76.704
63.742
63.499
52.304
52.259
39.266
39.152
24.817
MeO
N
N
N
O
O
*
*
1e
2:1 stereoisomers
200
180
160
140
120
100
80
60
40
20
ppm

Sample Name:
LN-362-1
Data Collected on:
Agilent-NMR.com-vnmrs400
Archive directory:
/home/vnmr1/vnmrsys/data/2011-koo-4
Sample directory:
LN-362-1_01
FidFile: LN-362-1_PROTON_01

Pulse Sequence: PROTON (s2pul)
Solvent: dmso
Data collected on: Nov 11 2020

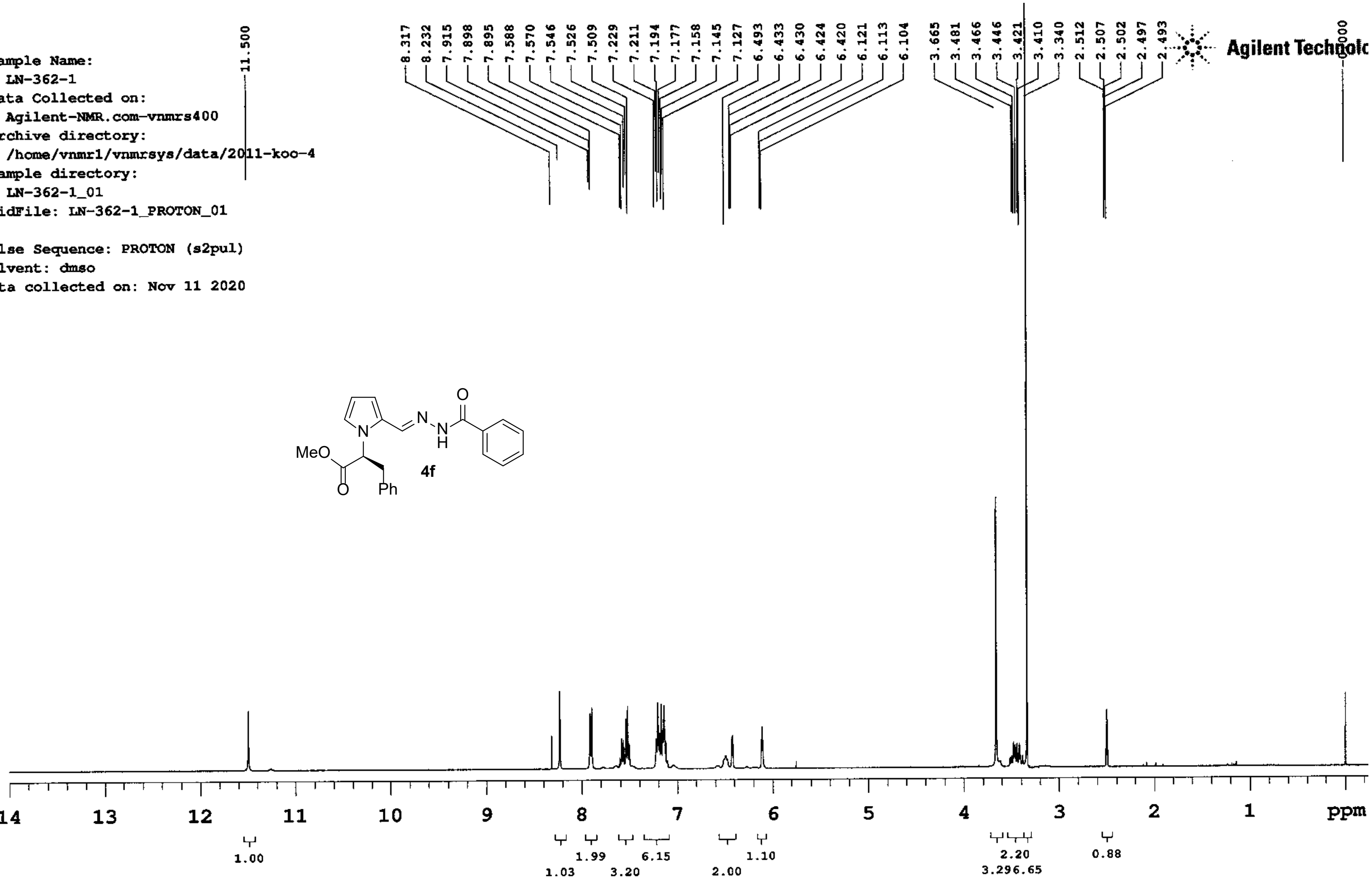

4f

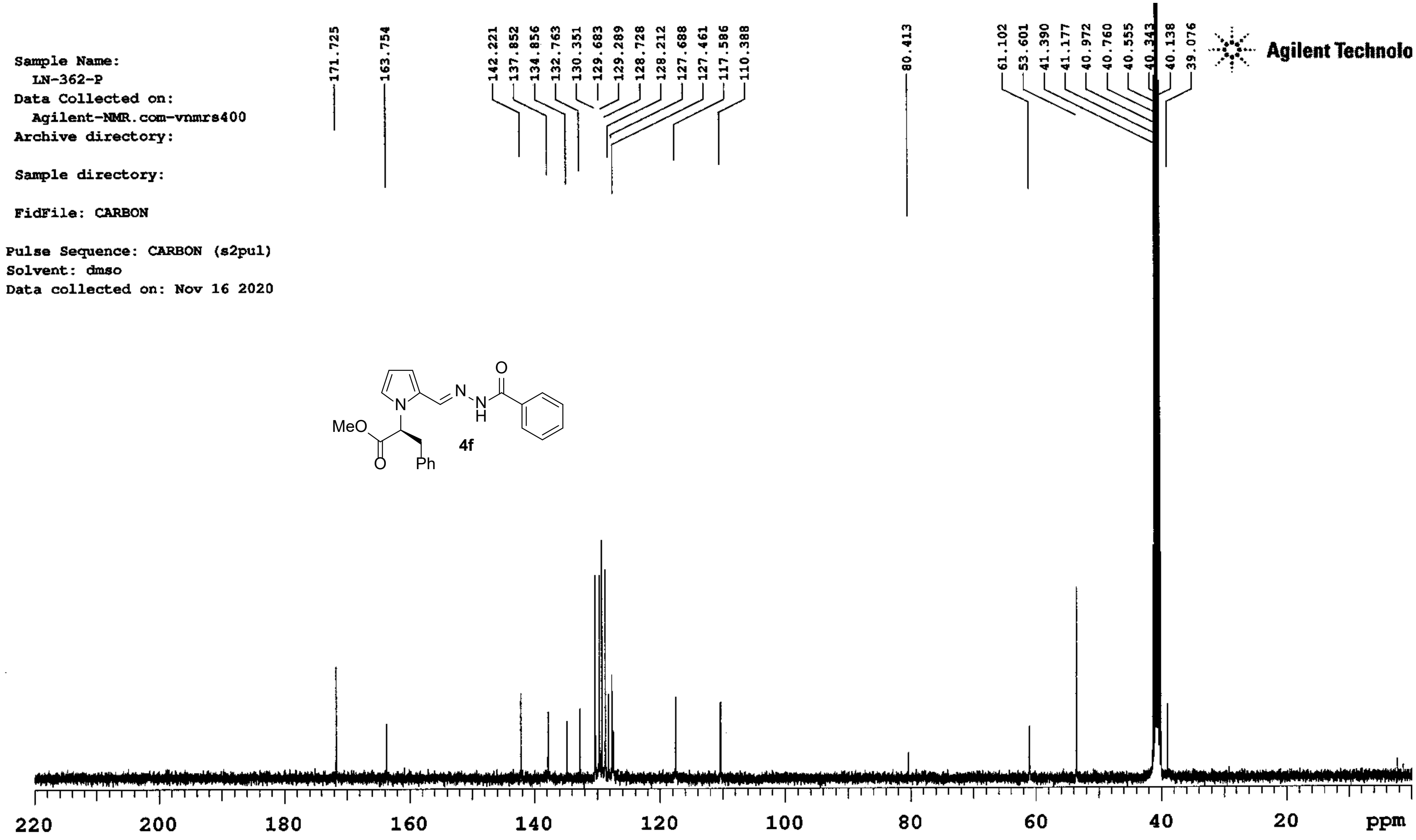
Sample Name:
LN-362-P
Data Collected on:
Agilent-NMR.com-vnmrs400
Archive directory:
Sample directory:
FidFile: CARBON
Pulse Sequence: CARBON (s2pul)
Solvent: dmso
Data collected on: Nov 16 2020
171.725
163.754
142.221
137.852
134.856
132.763
130.351
129.683
129.289
128.728
128.212
127.688
127.461
117.586
110.388
80.413
61.102
53.601
41.390
41.177
40.972
40.760
40.555
40.343
40.138
39.076
Agilent Technolo
MeO
O
N
N
N
H
O
Ph
4f
220
200
180
160
140
120
100
80
60
40
20
ppm

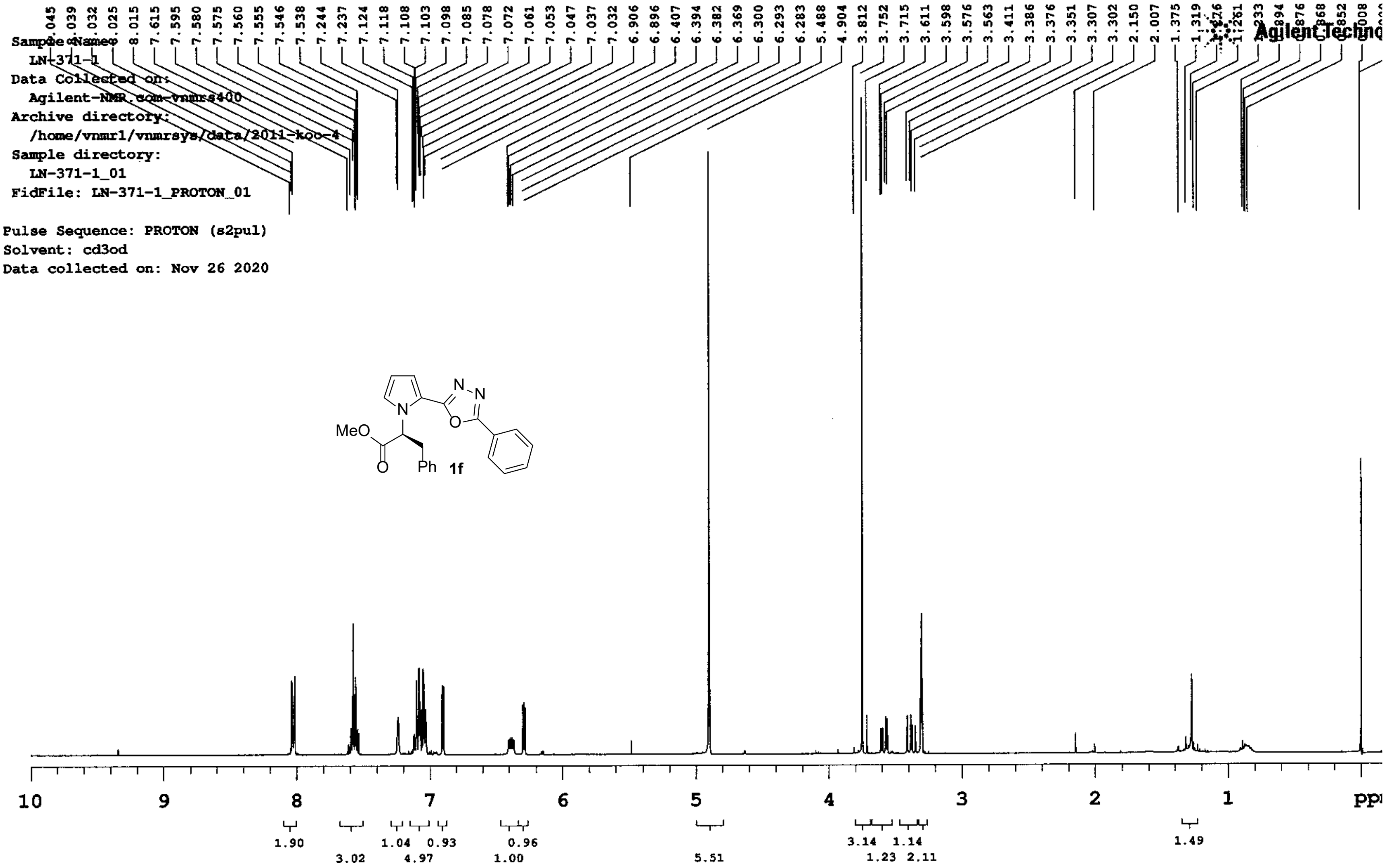

Sample Name:
LN-371-1
Data Collected on:
Agilent-NMR-vnmrs400
Archive directory:
/home/vnmr1/vnmrsys/data/2011-koo-4
Sample directory:
LN-371-1_01
FidFile: LN-371-1_PROTON_01
Pulse Sequence: PROTON (s2pul)
Solvent: cd3od
Data collected on: Nov 26 2020
Agilent Technologies
8.045 8.039 8.032 8.025 8.015 7.615 7.595 7.580 7.575 7.560 7.555 7.546 7.538 7.244 7.237 7.124 7.118 7.108 7.103 7.098 7.085 7.078 7.072 7.061 7.053 7.047 7.037 7.032 6.906 6.896 6.407 6.394 6.382 6.369 6.300 6.293 6.283 5.488 4.904 3.812 3.752 3.715 3.611 3.598 3.576 3.563 3.411 3.386 3.376 3.351 3.307 3.302 2.150 2.007 1.375 1.319 1.261 0.894 0.876 0.868 0.852 0.008
MeO
N
N
N
O
O
Ph
1f
10 9 8 7 6 5 4 3 2 1 ppm
1.90 3.02 1.04 4.97 0.93 1.00 0.96 5.51 3.14 1.23 1.14 2.11 1.49

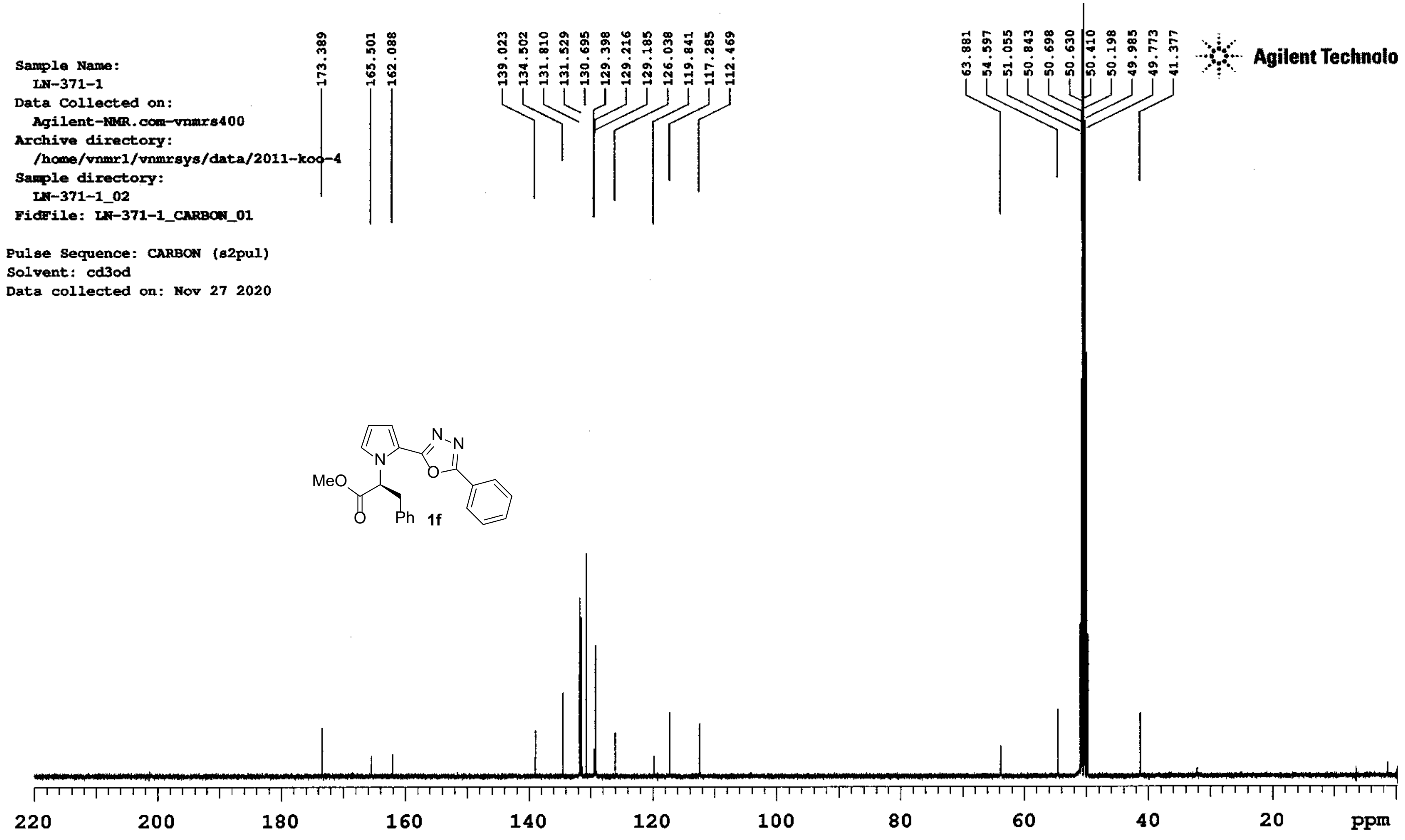
Sample Name:
LN-371-1
Data Collected on:
Agilent-NMR.com-vnmrs400
Archive directory:
/home/vnmr1/vnmrsys/data/2011-koo-4
Sample directory:
LN-371-1_02
FidFile: LN-371-1_CARBON_01
Pulse Sequence: CARBON (s2pul)
Solvent: cd3od
Data collected on: Nov 27 2020
Agilent Technolo
173.389
165.501
162.088
139.023
134.502
131.810
131.529
130.695
129.398
129.216
129.185
126.038
119.841
117.285
112.469
63.881
54.597
51.055
50.843
50.698
50.630
50.410
50.198
49.985
49.773
41.377
MeO
O
N
N
N
O
Ph
1f
220
200
180
160
140
120
100
80
60
40
20
ppm

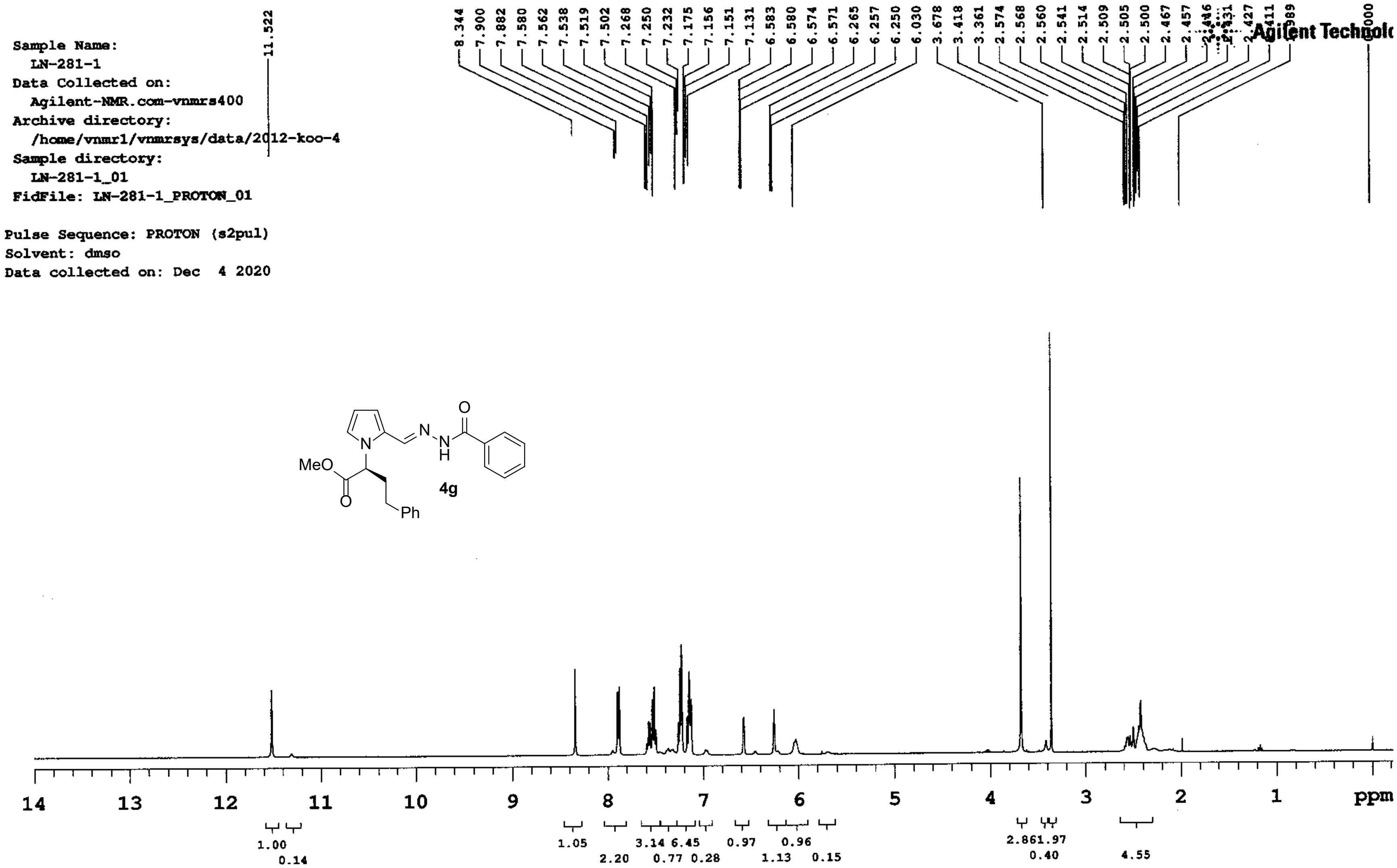

Sample Name:
LN-281-1
Data Collected on:
Agilent-NMR.com-vnmrs400
Archive directory:
/home/vnmr1/vnmrsys/data/2012-koo-4
Sample directory:
LN-281-1_01
FidFile: LN-281-1_PROTON_01
Pulse Sequence: PROTON (s2pul)
Solvent: dmso
Data collected on: Dec 4 2020
11.522
8.344
7.900
7.882
7.580
7.562
7.538
7.519
7.502
7.268
7.250
7.232
7.175
7.156
7.151
7.131
6.583
6.580
6.574
6.571
6.265
6.257
6.250
6.030
3.678
3.418
3.361
2.574
2.568
2.560
2.541
2.514
2.509
2.505
2.500
2.467
2.457
2.446
2.431
2.427
2.411
1.989
0.000
MeO
O
N
N
N
H
O
Ph
4g
14 13 12 11 10 9 8 7 6 5 4 3 2 1 ppm
1.00
0.14
1.05
2.20
3.14
6.45
0.77
0.28
0.97
1.13
0.96
0.15
2.86
1.97
0.40
4.55

Sample Name:
LN-281-1
Data Collected on:
Agilent-NMR.com-vnmrs400
Archive directory:
/home/vnmr1/vnmrsys/data/2012-koo-4
Sample directory:
LN-281-1_02
FidFile: LN-281-1_CARBON_01

Pulse Sequence: CARBON (s2pul)
Solvent: dmso
Data collected on: Dec 7 2020

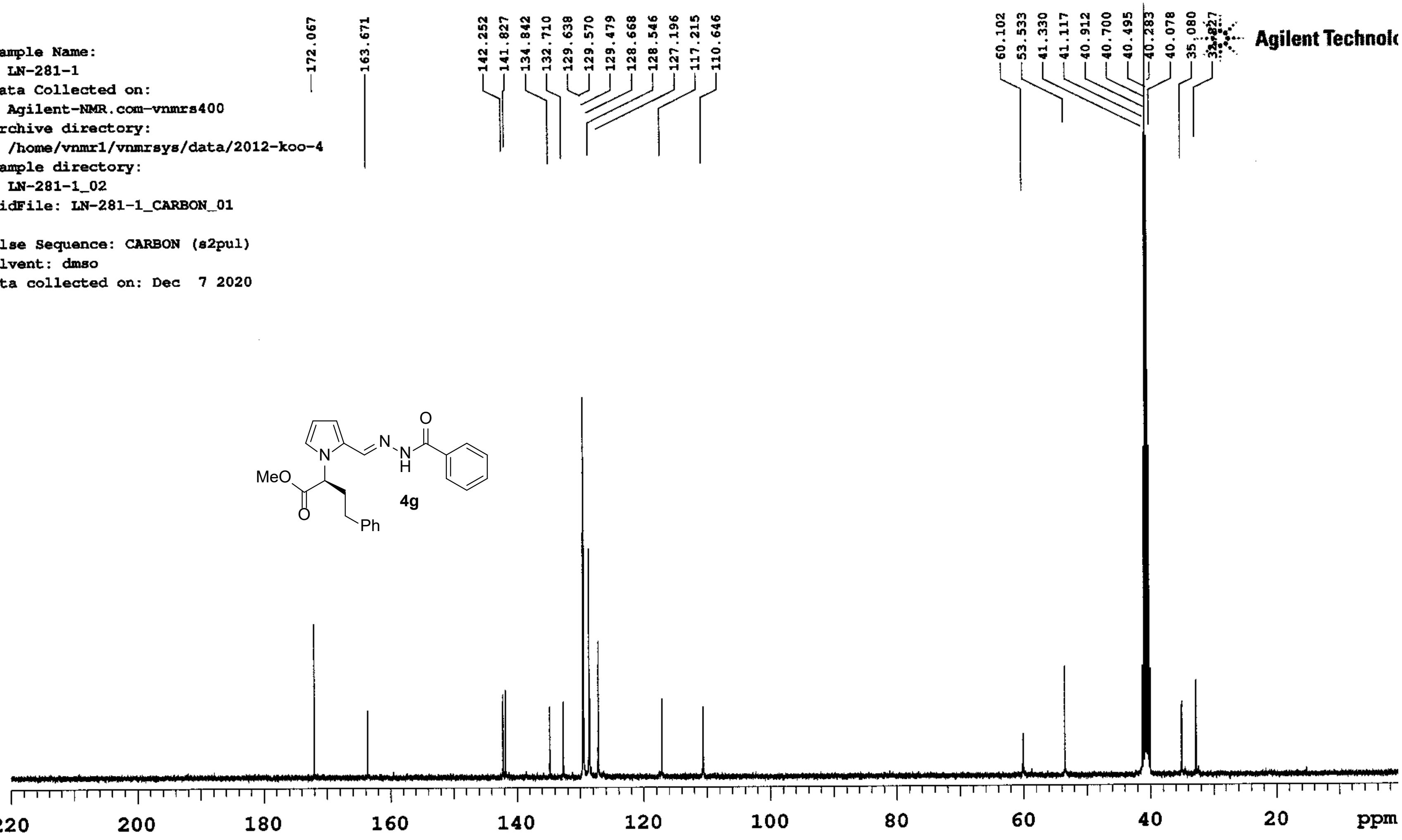

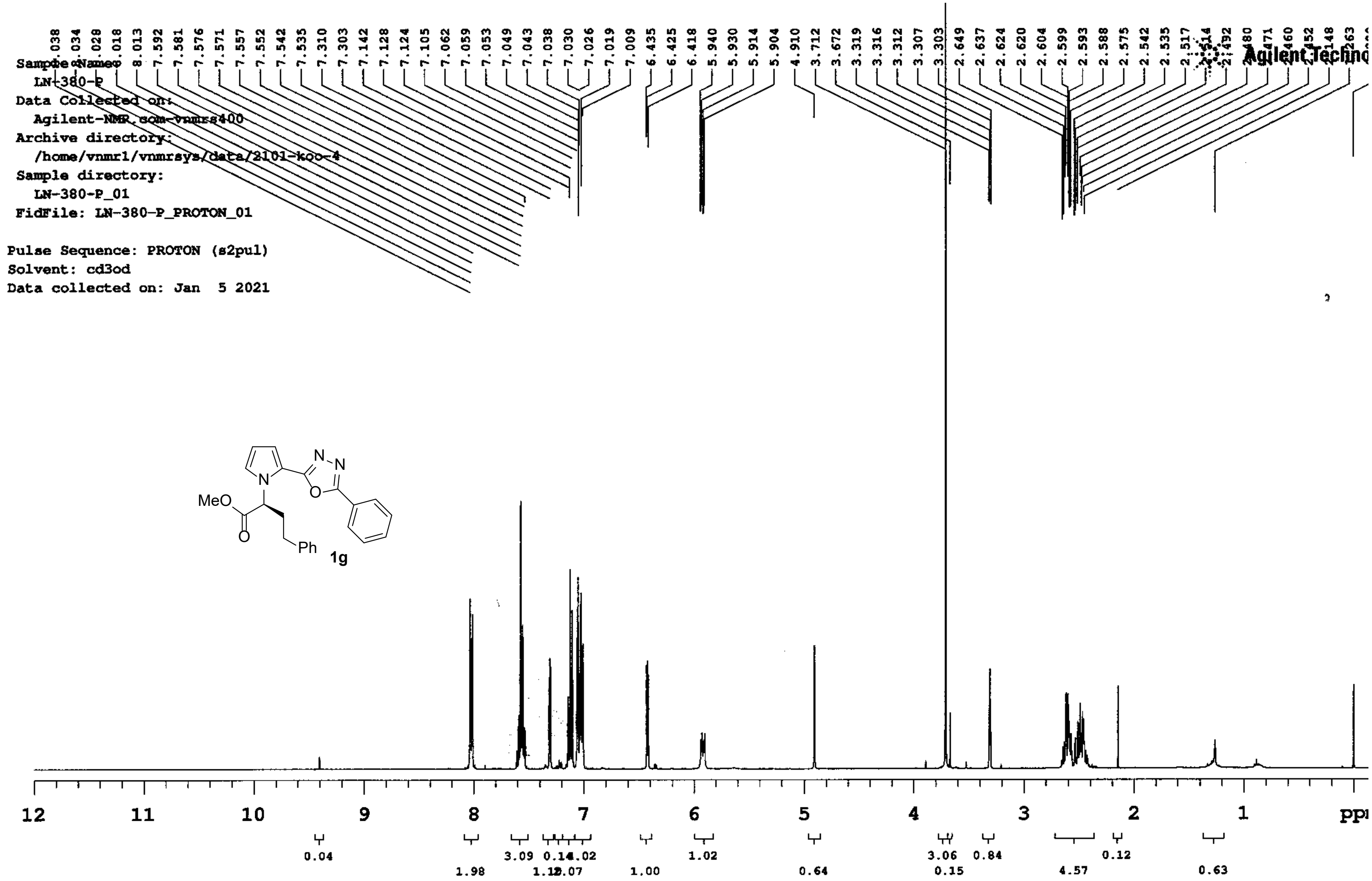

Sample Name:
LN-380-P
Data Collected on:
Agilent-NMR.com-vnmrs400
Archive directory:
/home/vnmr1/vnmrsys/data/2101-koo-4
Sample directory:
LN-380-P_01
FidFile: LN-380-P_PROTON_01
Pulse Sequence: PROTON (s2pul)
Solvent: cd3od
Data collected on: Jan 5 2021
Agilent Technologies
8.038 8.034 8.028 8.018 8.013 7.592 7.581 7.576 7.571 7.557 7.552 7.542 7.535 7.310 7.303 7.142 7.128 7.124 7.105 7.062 7.059 7.053 7.049 7.043 7.038 7.030 7.026 7.019 7.009 6.435 6.425 6.418 5.940 5.930 5.914 5.904 4.910 3.712 3.672 3.319 3.316 3.312 3.307 3.303 2.649 2.637 2.624 2.620 2.604 2.599 2.593 2.588 2.575 2.542 2.535 2.517
MeO
N
O
Ph
1g
12 11 10 9 8 7 6 5 4 3 2 1 ppm
0.04 1.98 3.09 1.00 1.02 0.64 3.06 0.15 0.84 4.57 0.12 0.63

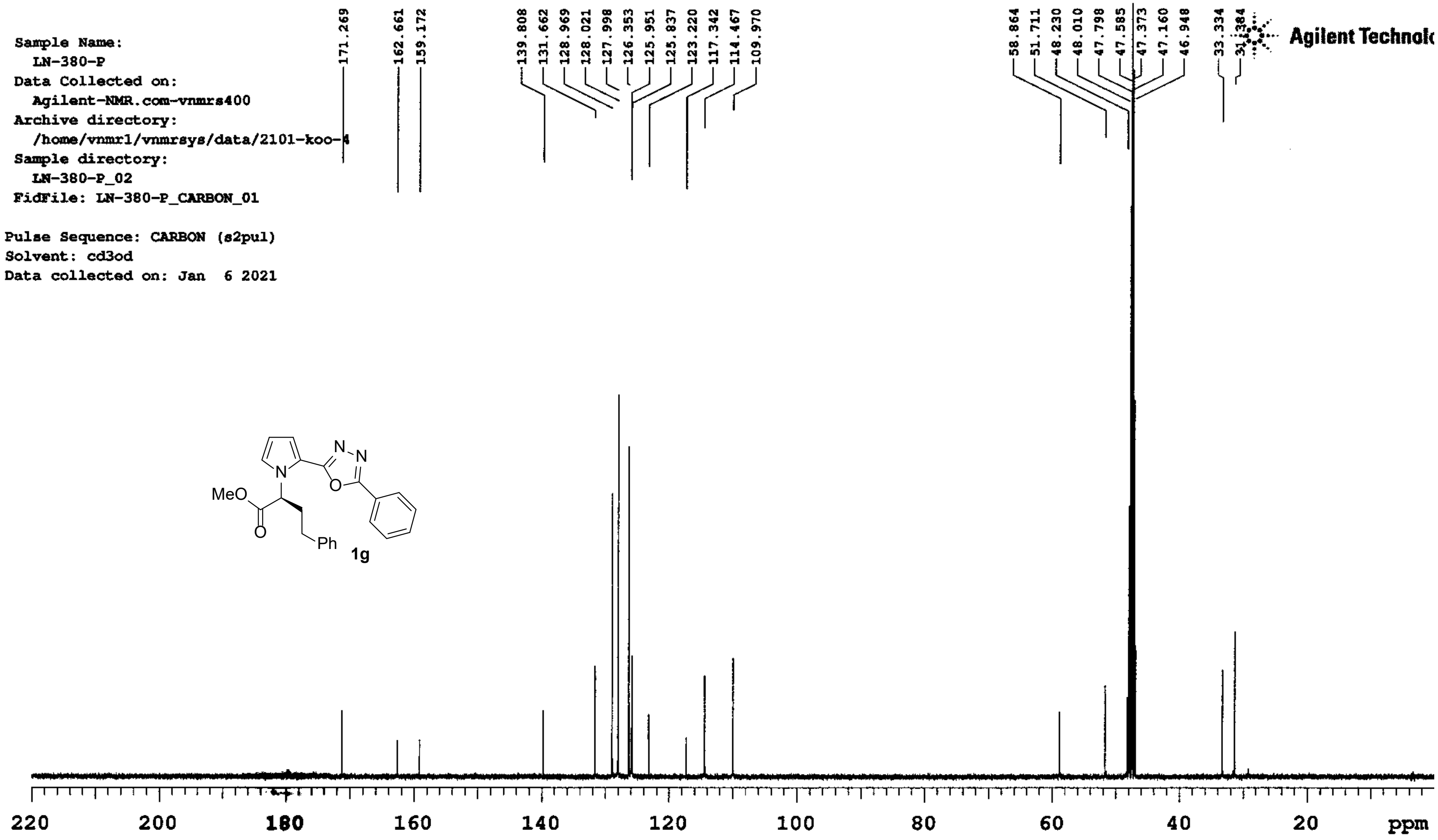
Sample Name:
LN-380-P
Data Collected on:
Agilent-NMR.com-vnmrs400
Archive directory:
/home/vnmr1/vnmrsys/data/2101-koo-4
Sample directory:
LN-380-P_02
FidFile: LN-380-P_CARBON_01
Pulse Sequence: CARBON (s2pul)
Solvent: cd3od
Data collected on: Jan 6 2021
171.269
162.661
159.172
139.808
131.662
128.969
128.021
127.998
126.353
125.951
125.837
123.220
117.342
114.467
109.970
58.864
51.711
48.230
48.010
47.798
47.585
47.373
47.160
46.948
33.334
31.384
Agilent Technolo
MeO
O
N
N
N
O
Ph
1g
220
200
180
160
140
120
100
80
60
40
20
ppm

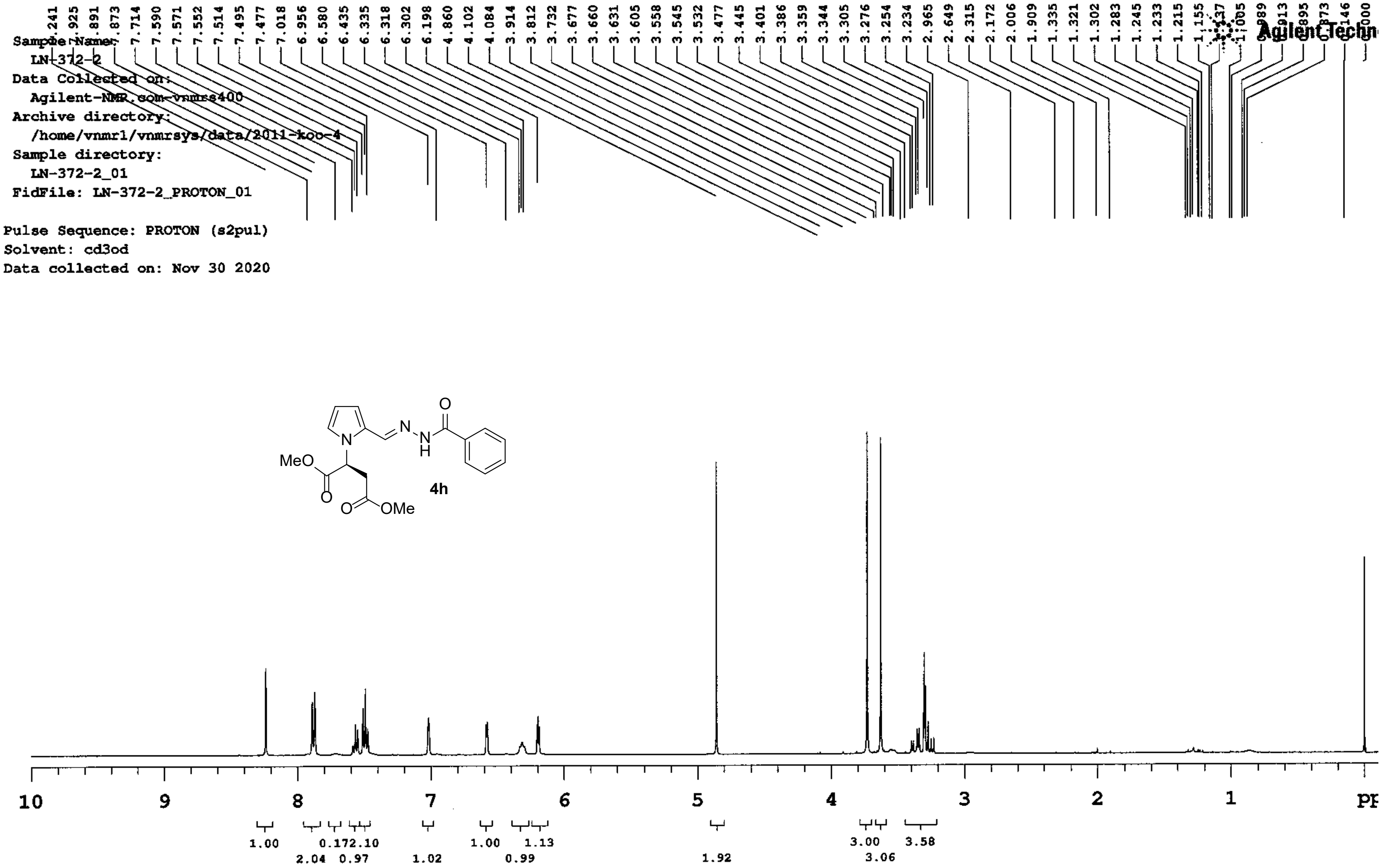
Sample Name:
LN-372-2
Data Collected on:
Agilent-NMR.com-vnmrs400
Archive directory:
/home/vnmrl/vnmrsys/data/2011-koo-4
Sample directory:
LN-372-2_01
FidFile: LN-372-2_PROTON_01
Pulse Sequence: PROTON (s2pul)
Solvent: cd3od
Data collected on: Nov 30 2020
Agilent Techn
8.241 7.925 7.891 7.873 7.714 7.590 7.571 7.552 7.514 7.495 7.477 7.018 6.956 6.580 6.435 6.335 6.318 6.302 6.198 4.860 4.102 4.084 3.914 3.812 3.732 3.677 3.660 3.631 3.605 3.558 3.545 3.532 3.477 3.445 3.401 3.386 3.359 3.344 3.305 3.276 3.254 3.234 2.965 2.649 2.315 2.172 2.006 1.909 1.335 1.321 1.302 1.283 1.245 1.233 1.215 1.155 1.005 0.000
MeO
O
N
N
N
H
O
O
OMe
4h
10 9 8 7 6 5 4 3 2 1 pp
1.00 2.04 0.17 2.10 0.97 1.02 1.00 0.99 1.13 1.92 3.00 3.06 3.58

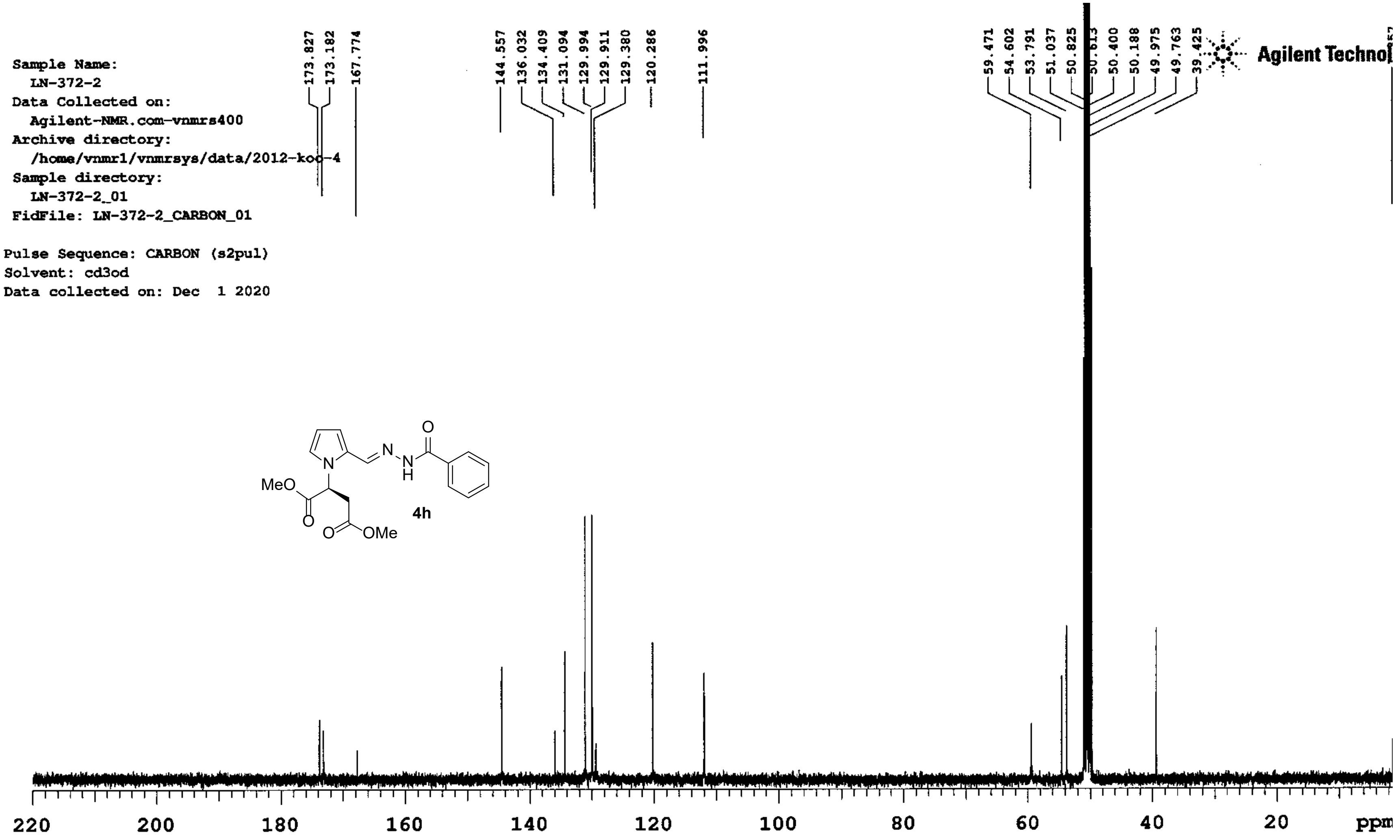
Sample Name:
LN-372-2
Data Collected on:
Agilent-NMR.com-vnmrs400
Archive directory:
/home/vnmr1/vnmrsys/data/2012-koc-4
Sample directory:
LN-372-2_01
FidFile: LN-372-2_CARBON_01
Pulse Sequence: CARBON (s2pul)
Solvent: cd3od
Data collected on: Dec 1 2020
173.827
173.182
167.774
144.557
136.032
134.409
131.094
129.994
129.911
129.380
120.286
111.996
59.471
54.602
53.791
51.037
50.825
50.613
50.400
50.188
49.975
49.763
39.425
Agilent Technol
MeO
O
N
N
H
OMe
4h
220
200
180
160
140
120
100
80
60
40
20
ppm

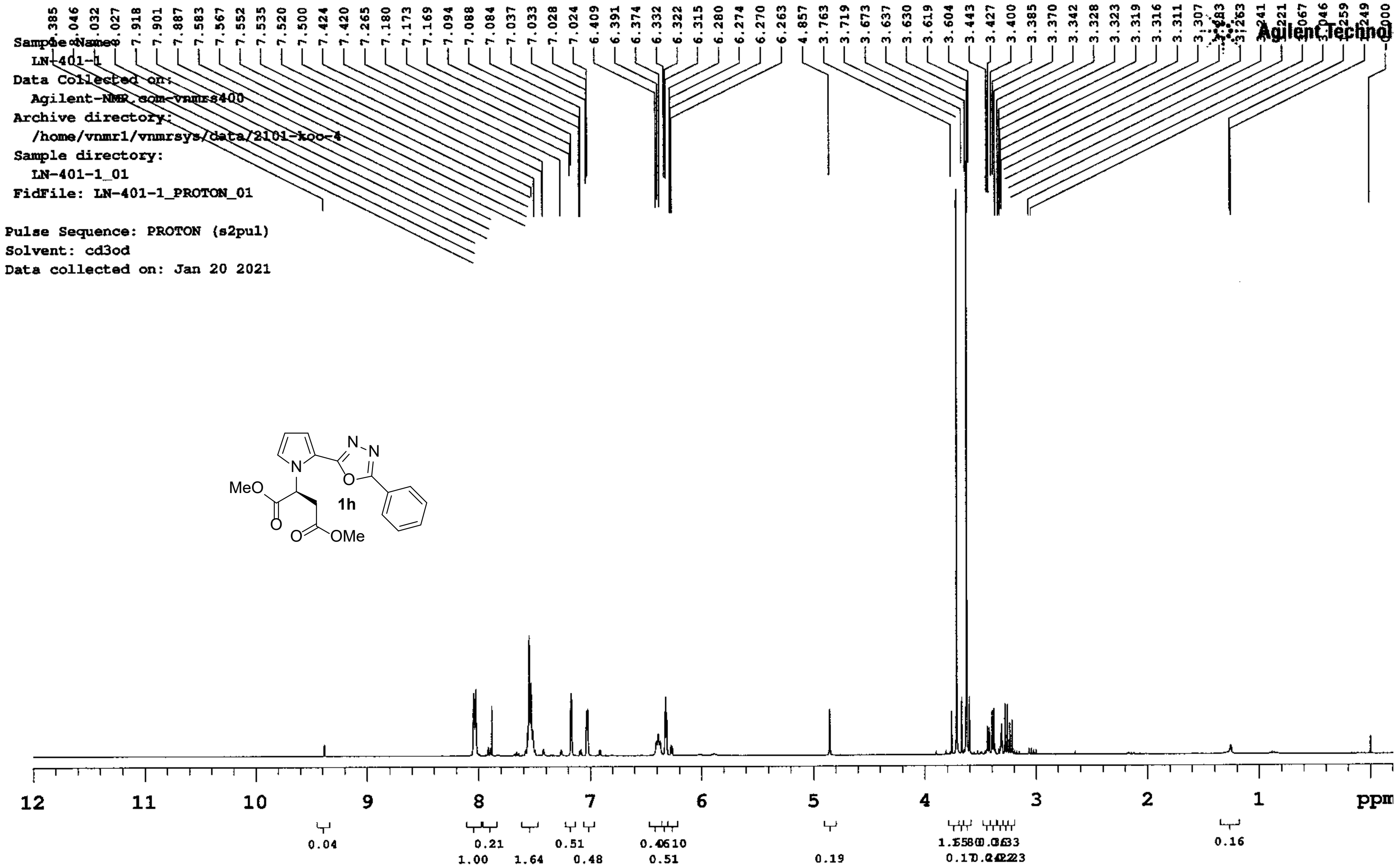
Sample Name:
LN-401-1
Data Collected on:
Agilent-NMR.com-vnmrs400
Archive directory:
/home/vnmr1/vnmrsys/data/2101-koo-4
Sample directory:
LN-401-1_01
FidFile: LN-401-1_PROTON_01
Pulse Sequence: PROTON (s2pul)
Solvent: cd3od
Data collected on: Jan 20 2021
Agilent Technol
MeO
N
N
N
O
O
O
OMe
1h
ppm

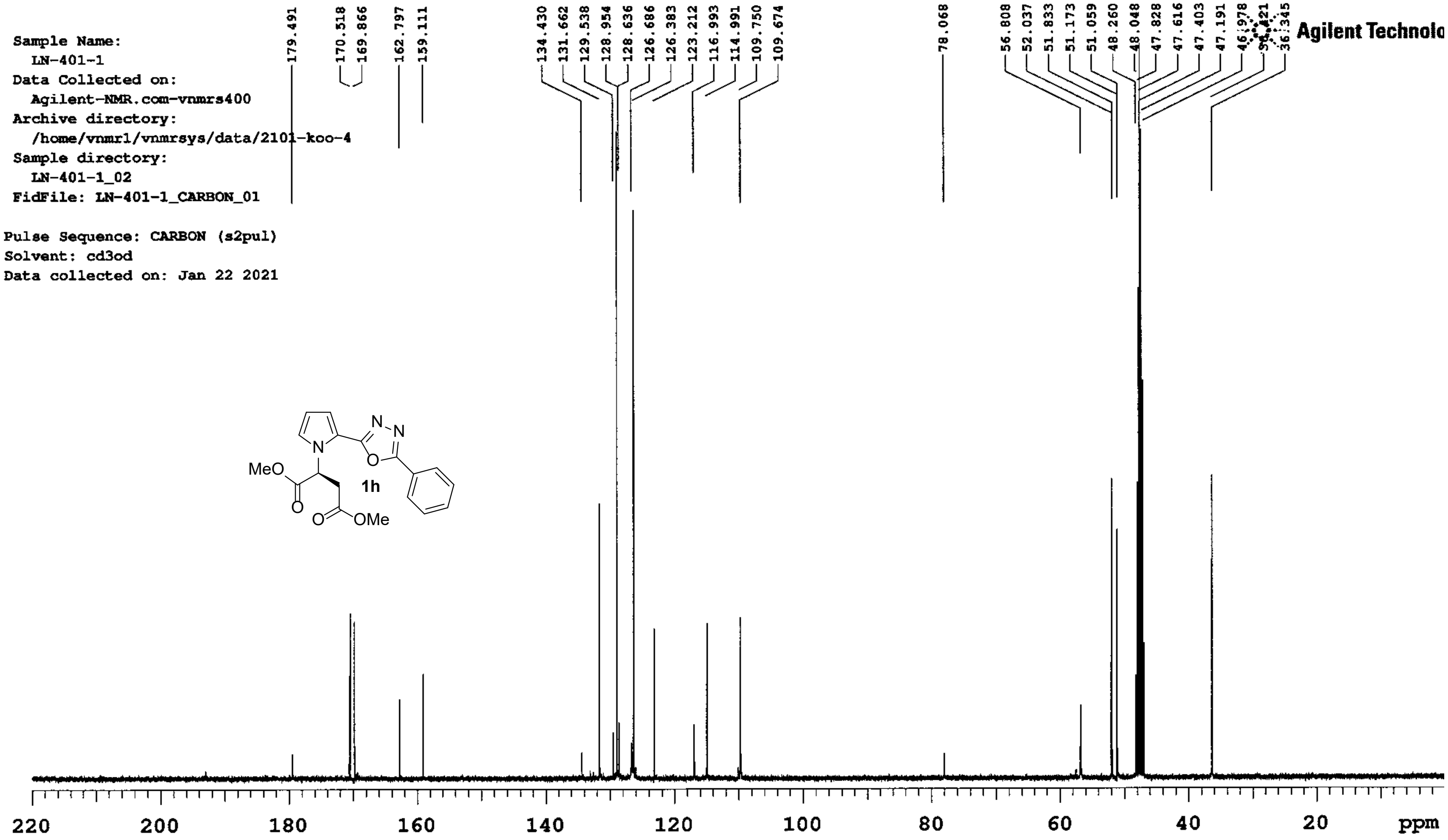

Sample Name:
LN-401-1
Data Collected on:
Agilent-NMR.com-vnmrs400
Archive directory:
/home/vnmr1/vnmrsys/data/2101-koo-4
Sample directory:
LN-401-1_02
FidFile: LN-401-1_CARBON_01
Pulse Sequence: CARBON (s2pul)
Solvent: cd3od
Data collected on: Jan 22 2021
Agilent Technolo
179.491
170.518
169.866
162.797
159.111
134.430
131.662
129.538
128.954
128.636
126.686
126.383
123.212
116.993
114.991
109.750
109.674
78.068
56.808
52.037
51.833
51.173
51.059
48.260
48.048
47.828
47.616
47.403
47.191
46.978
36.345
MeO
N
N
N
O
1h
O
O
OMe
220
200
180
160
140
120
100
80
60
40
20
ppm

Sample Name:
LN-374-1
Data Collected on:
Agilent-NMR.com-vnmrs400
Archive directory:
/home/vnmr1/vnmrsys/data/2012-koo-4
Sample directory:
LN-374-1_01
FidFile: LN-374-1_PROTON_01

Pulse Sequence: PROTON (s2pul)
Solvent: dmso
Data collected on: Dec 4 2020

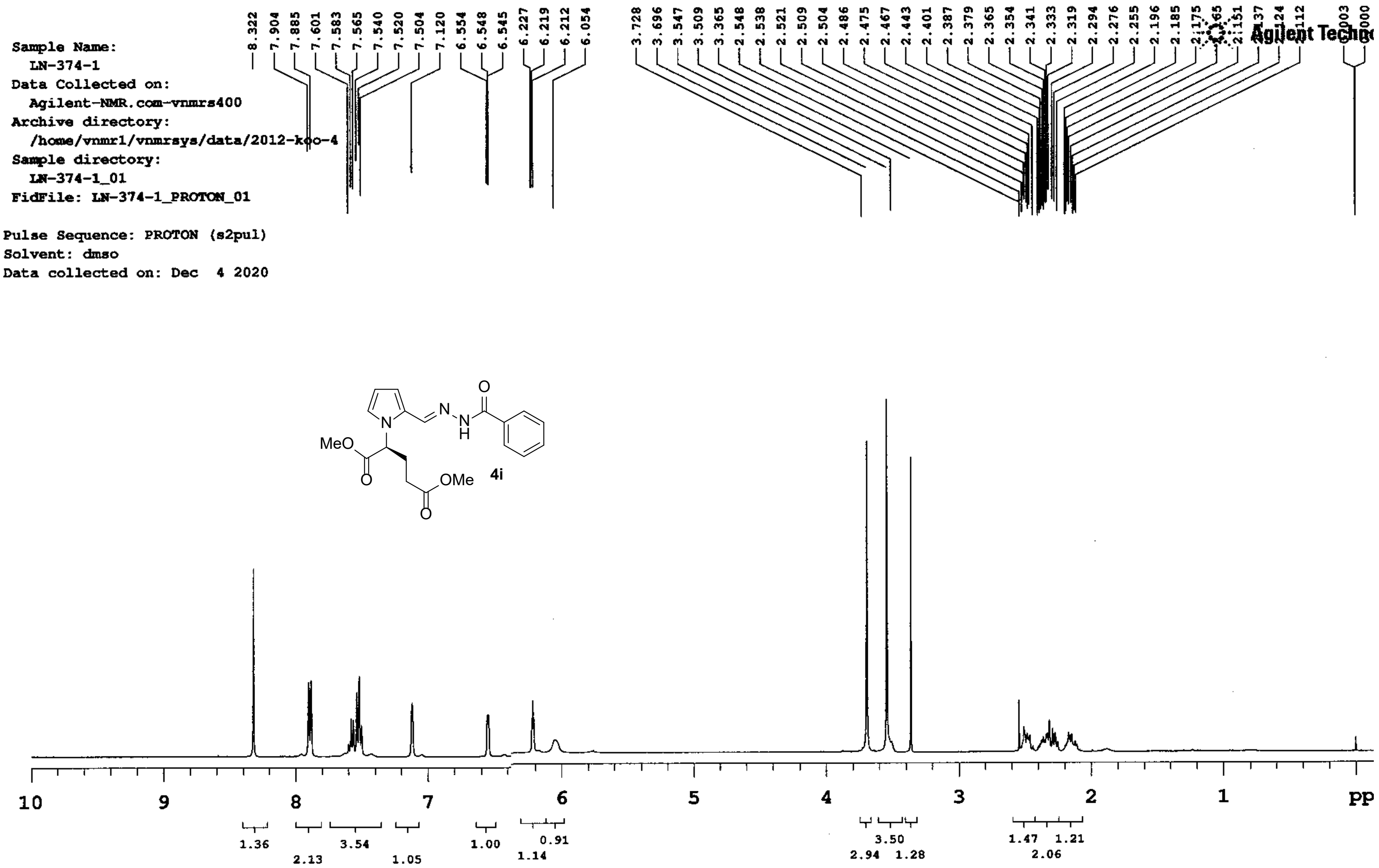

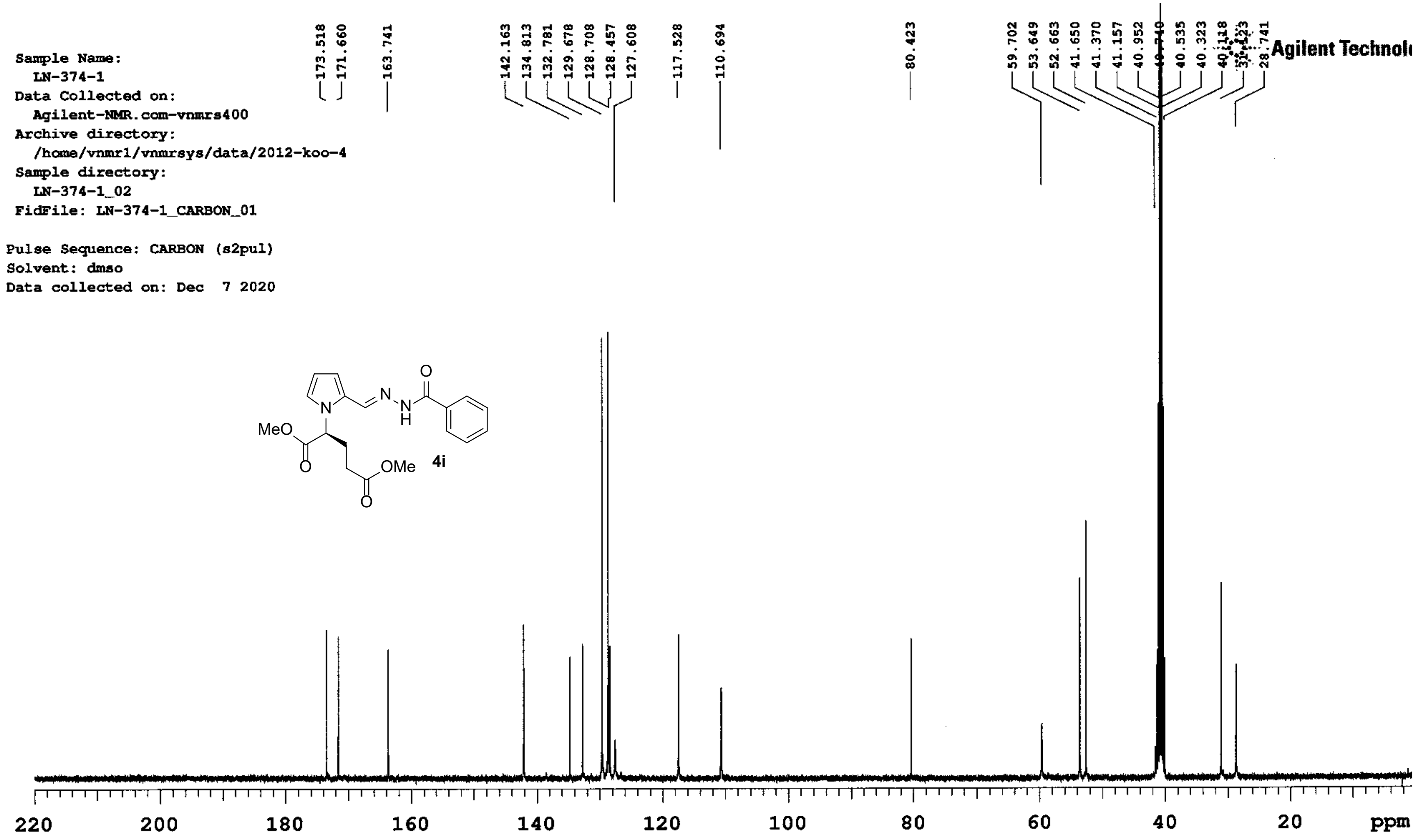
Sample Name:
LN-374-1
Data Collected on:
Agilent-NMR.com-vnmrs400
Archive directory:
/home/vnmr1/vnmrsys/data/2012-koo-4
Sample directory:
LN-374-1_02
FidFile: LN-374-1_CARBON_01
Pulse Sequence: CARBON (s2pul)
Solvent: dmso
Data collected on: Dec 7 2020
Agilent Technolo
173.518
171.660
163.741
142.163
134.813
132.781
129.678
128.708
128.457
127.608
117.528
110.694
80.423
59.702
53.649
52.663
41.650
41.370
41.157
40.952
40.535
40.323
28.741
MeO
OMe
O
N
H
4i
220
200
180
160
140
120
100
80
60
40
20
ppm

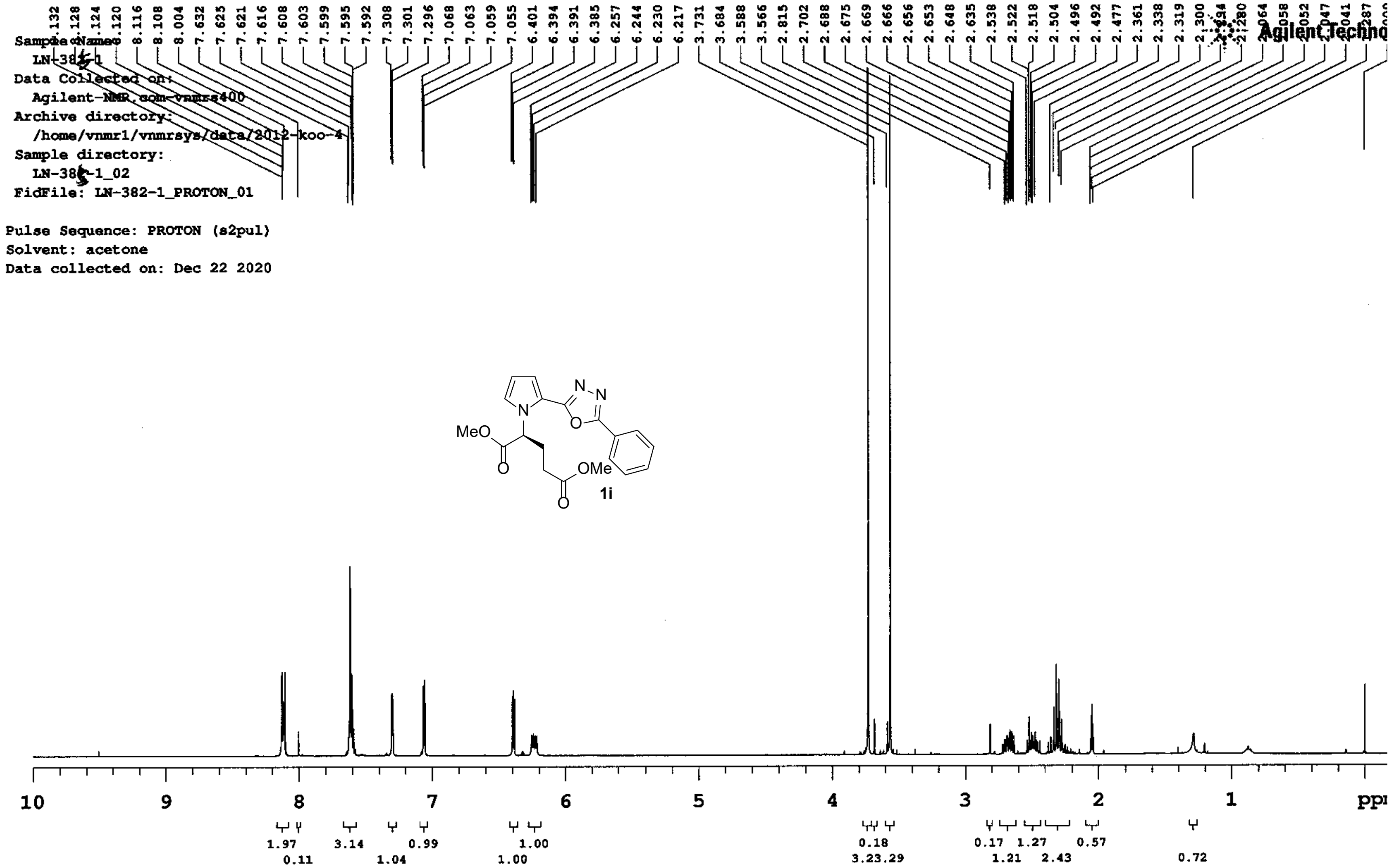
Sample Name:
LN-382-1
Data Collected on:
Agilent-NMR-vnmrs400
Archive directory:
/home/vnmr1/vnmrsys/data/2012-koo-4
Sample directory:
LN-382-1_02
FidFile: LN-382-1_PROTON_01
Pulse Sequence: PROTON (s2pul)
Solvent: acetone
Data collected on: Dec 22 2020
MeO
OMe
1i
ppm

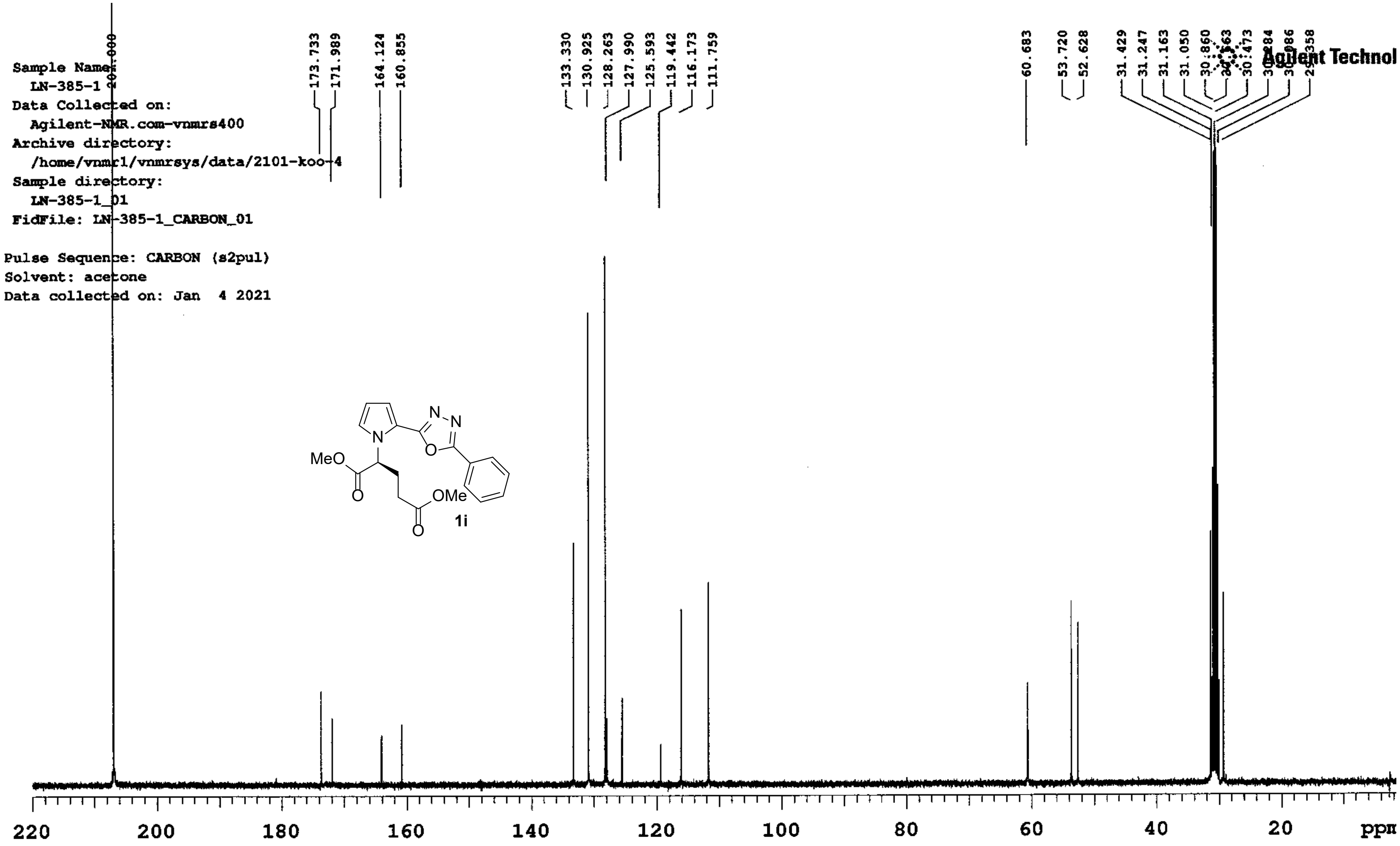
Sample Name:
LN-385-1
Data Collected on:
Agilent-NMR.com-vnmrs400
Archive directory:
/home/vnmr1/vnmrsys/data/2101-koo-4
Sample directory:
LN-385-1_01
FidFile: LN-385-1_CARBON_01
Pulse Sequence: CARBON (s2pul)
Solvent: acetone
Data collected on: Jan 4 2021
Agilent Technol
173.733
171.989
164.124
160.855
133.330
130.925
128.263
127.990
125.593
119.442
116.173
111.759
60.683
53.720
52.628
31.429
31.247
31.163
31.050
30.860
30.473
MeO
N
N–N
O
OMe
O
O
220
200
180
160
140
120
100
80
60
40
20
ppm

1i

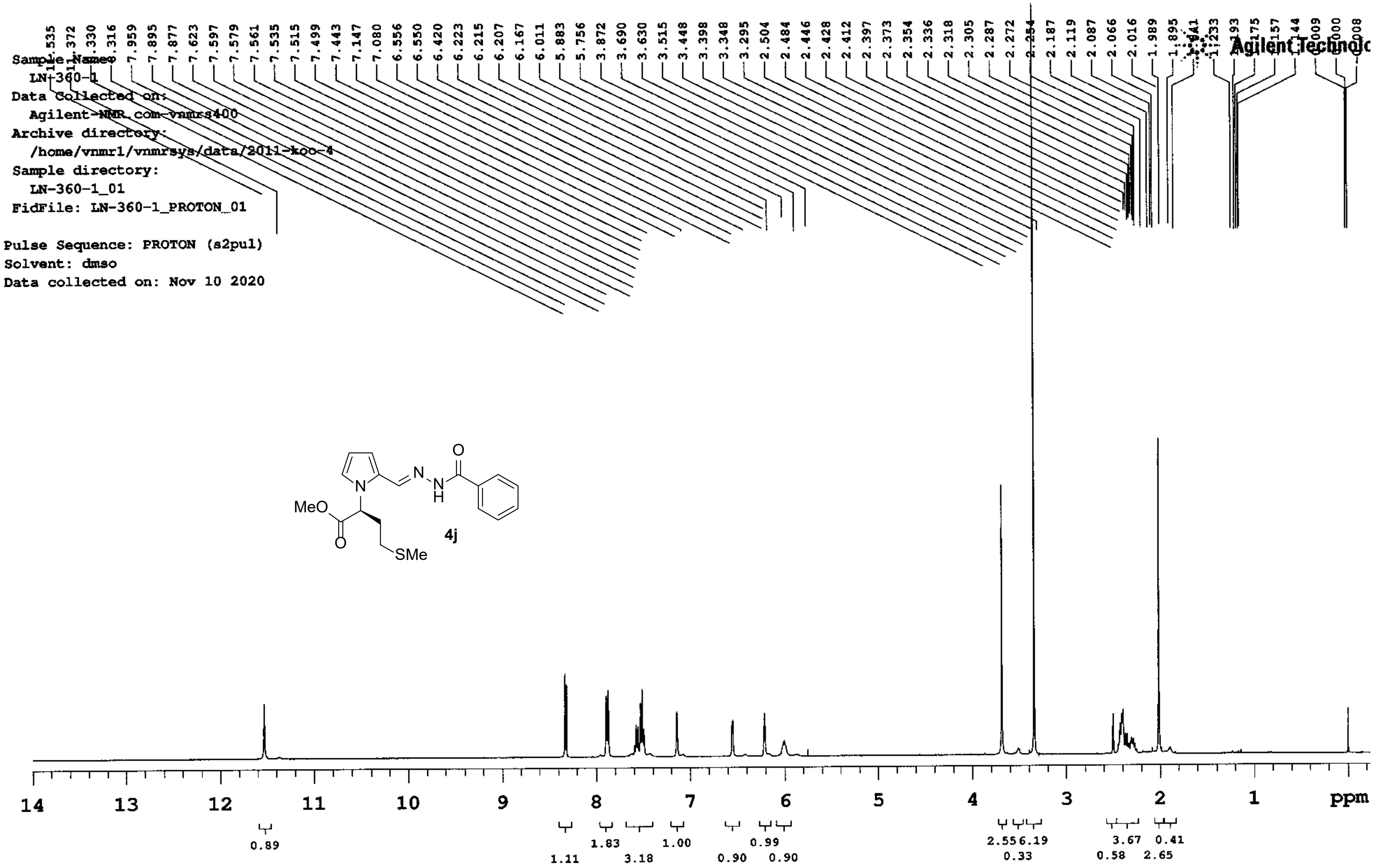
Sample Name:
LN-360-1
Data Collected on:
Agilent-NMR.com-vnmrs400
Archive directory:
/home/vnmr1/vnmrsys/data/2011-koo-4
Sample directory:
LN-360-1_01
FidFile: LN-360-1_PROTON_01
Pulse Sequence: PROTON (s2pul)
Solvent: dmso
Data collected on: Nov 10 2020
Agilent Technologies
MeO
O
N
N
N
H
O
SMe
4j
14 13 12 11 10 9 8 7 6 5 4 3 2 1 ppm
0.89
1.11 1.83 3.18 1.00 0.90 0.99 0.90
2.55 6.19 0.33
0.58 3.67 2.65 0.41

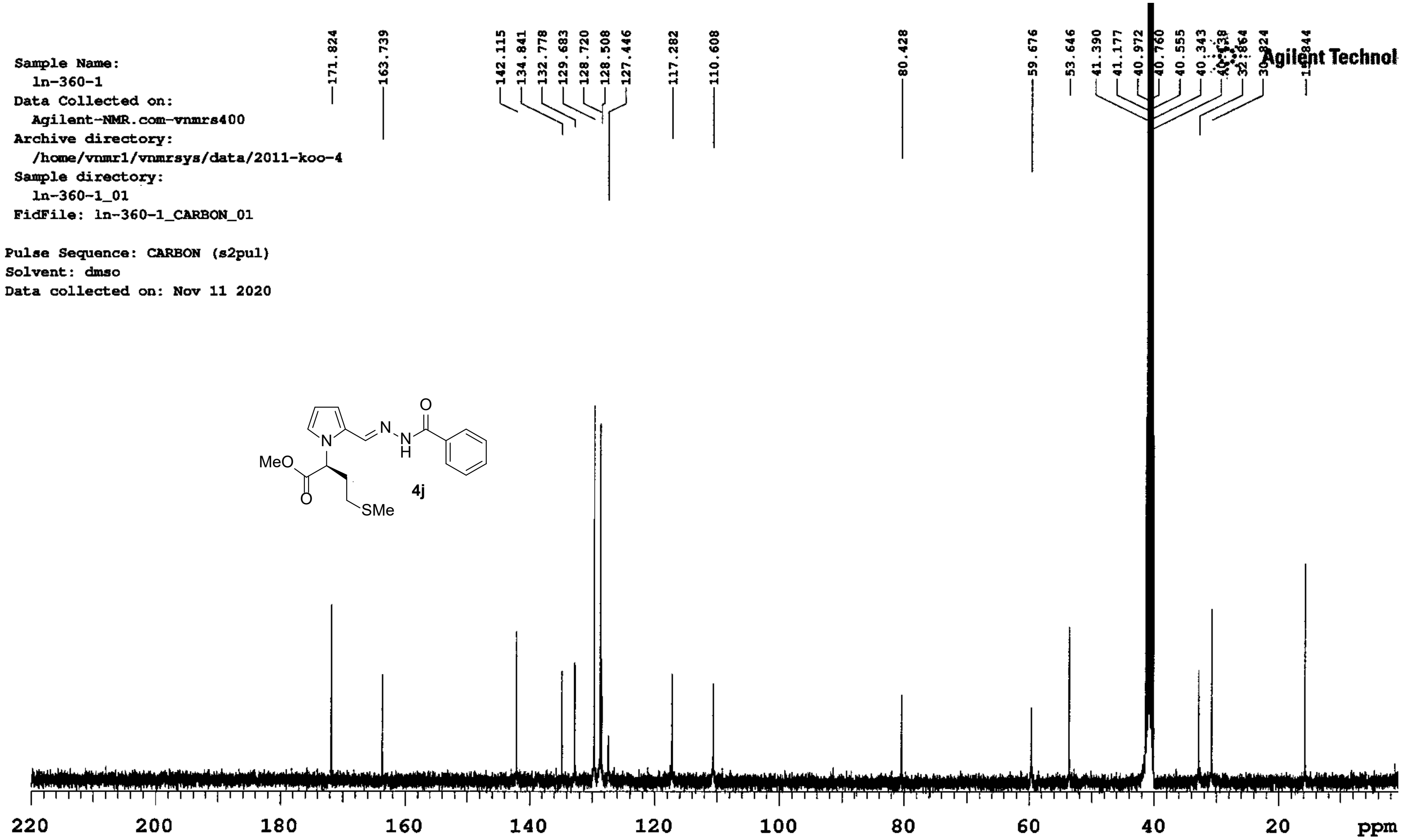

Sample Name:
ln-360-1
Data Collected on:
Agilent-NMR.com-vnmrs400
Archive directory:
/home/vnmr1/vnmrsys/data/2011-koo-4
Sample directory:
ln-360-1_01
FidFile: ln-360-1_CARBON_01
Pulse Sequence: CARBON (s2pul)
Solvent: dmso
Data collected on: Nov 11 2020
171.824
163.739
142.115
134.841
132.778
129.683
128.720
128.508
127.446
117.282
110.608
80.428
59.676
53.646
41.390
41.177
40.972
40.760
40.555
40.343
30.824
Agilent Technol
MeO
O
N
N
N
H
O
SMe
4j
220
200
180
160
140
120
100
80
60
40
20
ppm

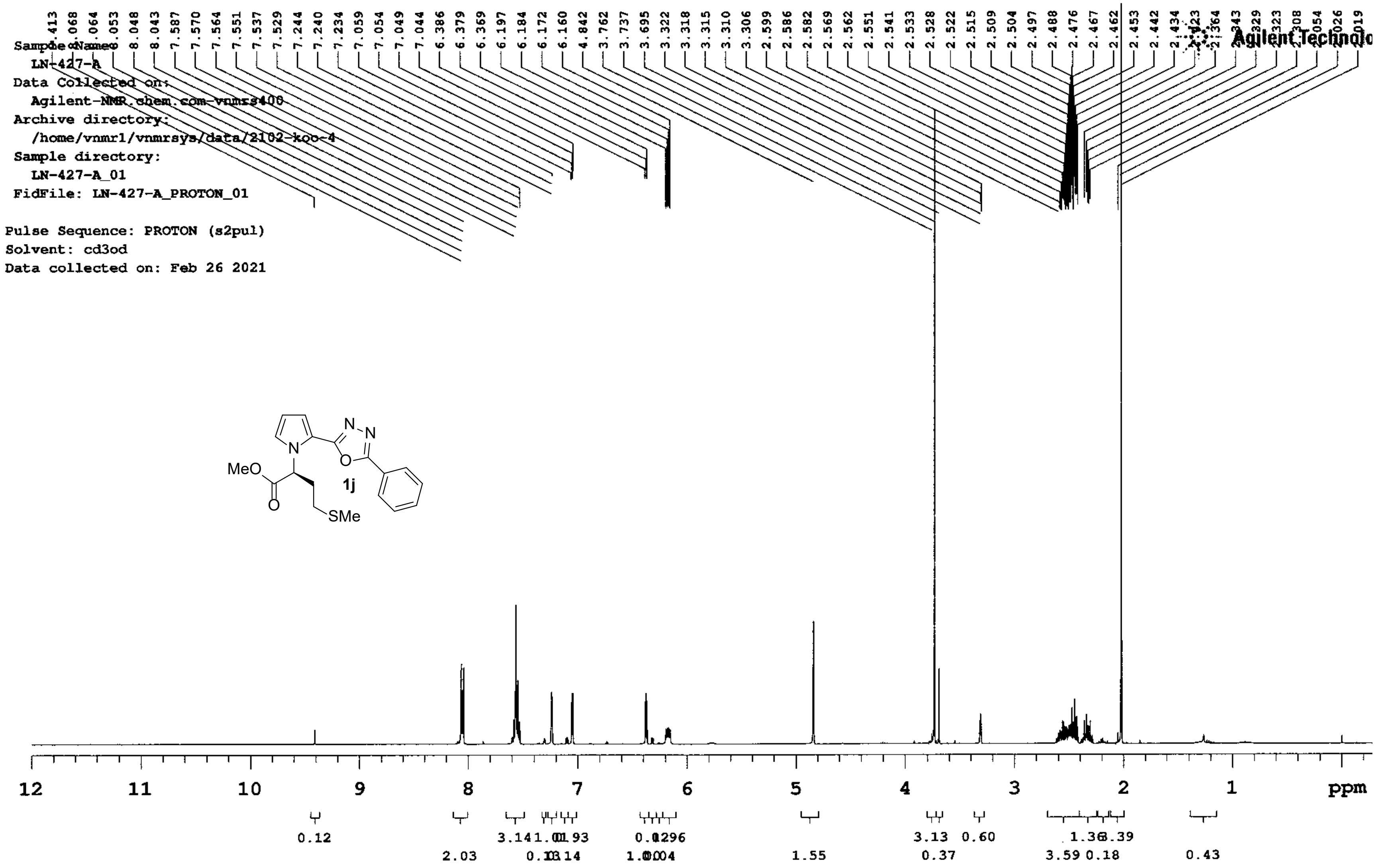
Sample Name:
LN-427-A
Data Collected on:
Agilent-NMR.chem.com-vnmrs400
Archive directory:
/home/vnmr1/vnmrsys/data/2102-koo-4
Sample directory:
LN-427-A_01
FidFile: LN-427-A_PROTON_01
Pulse Sequence: PROTON (s2pul)
Solvent: cd3od
Data collected on: Feb 26 2021
Agilent Technologies
MeO
N
N
N
O
O
SMe
1j
12 11 10 9 8 7 6 5 4 3 2 1 ppm

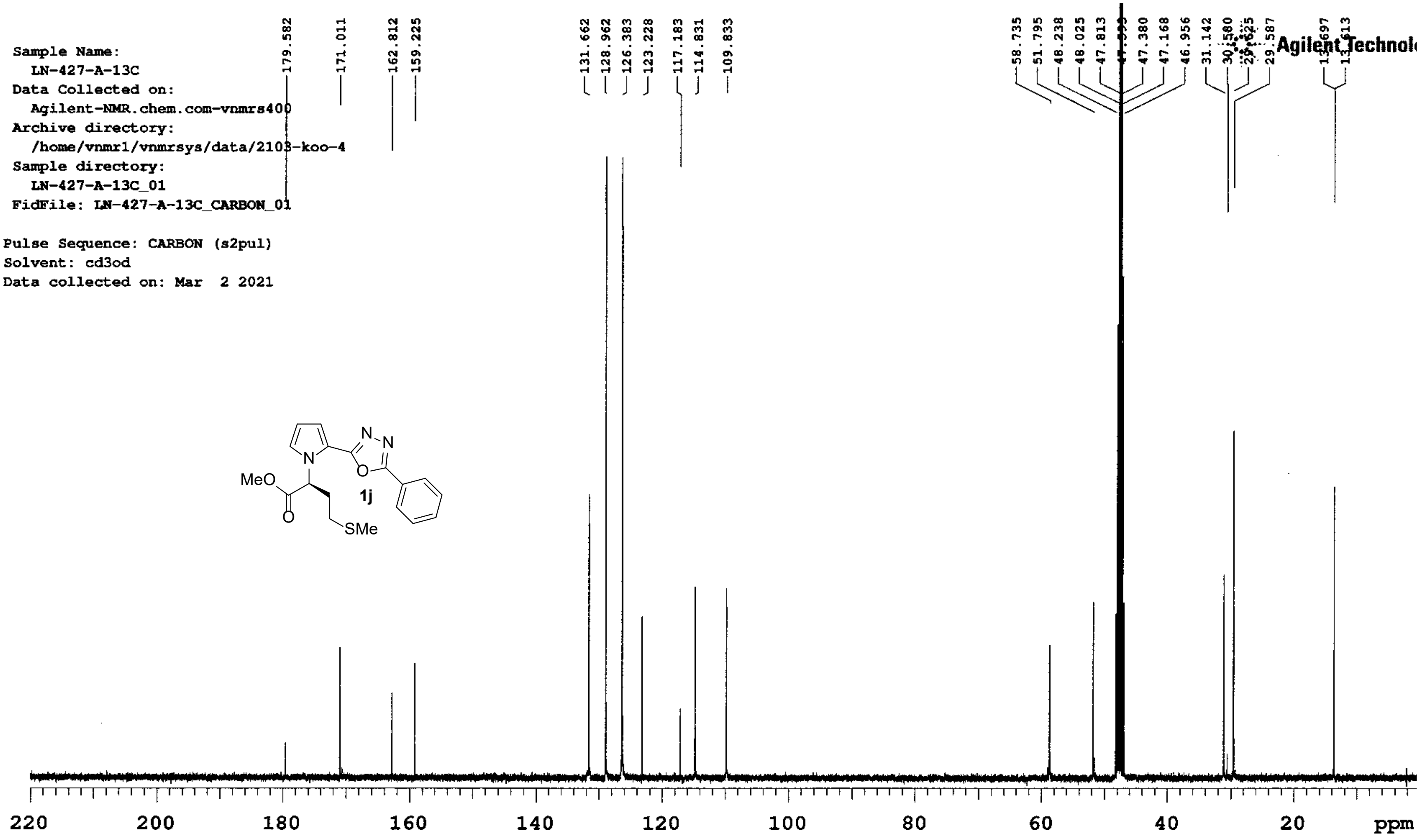
Sample Name:
LN-427-A-13C
Data Collected on:
Agilent-NMR.chem.com-vnmrs400
Archive directory:
/home/vnmr1/vnmrsys/data/2103-koo-4
Sample directory:
LN-427-A-13C_01
FidFile: LN-427-A-13C_CARBON_01
Pulse Sequence: CARBON (s2pul)
Solvent: cd3od
Data collected on: Mar 2 2021
179.582
171.011
162.812
159.225
131.662
128.962
126.383
123.228
117.183
114.831
109.833
58.735
51.795
48.238
48.025
47.813
47.380
47.168
46.956
31.142
29.587
Agilent Technol
MeO
O
N
N
N
O
SMe
1j
220
200
180
160
140
120
100
80
60
40
20
ppm

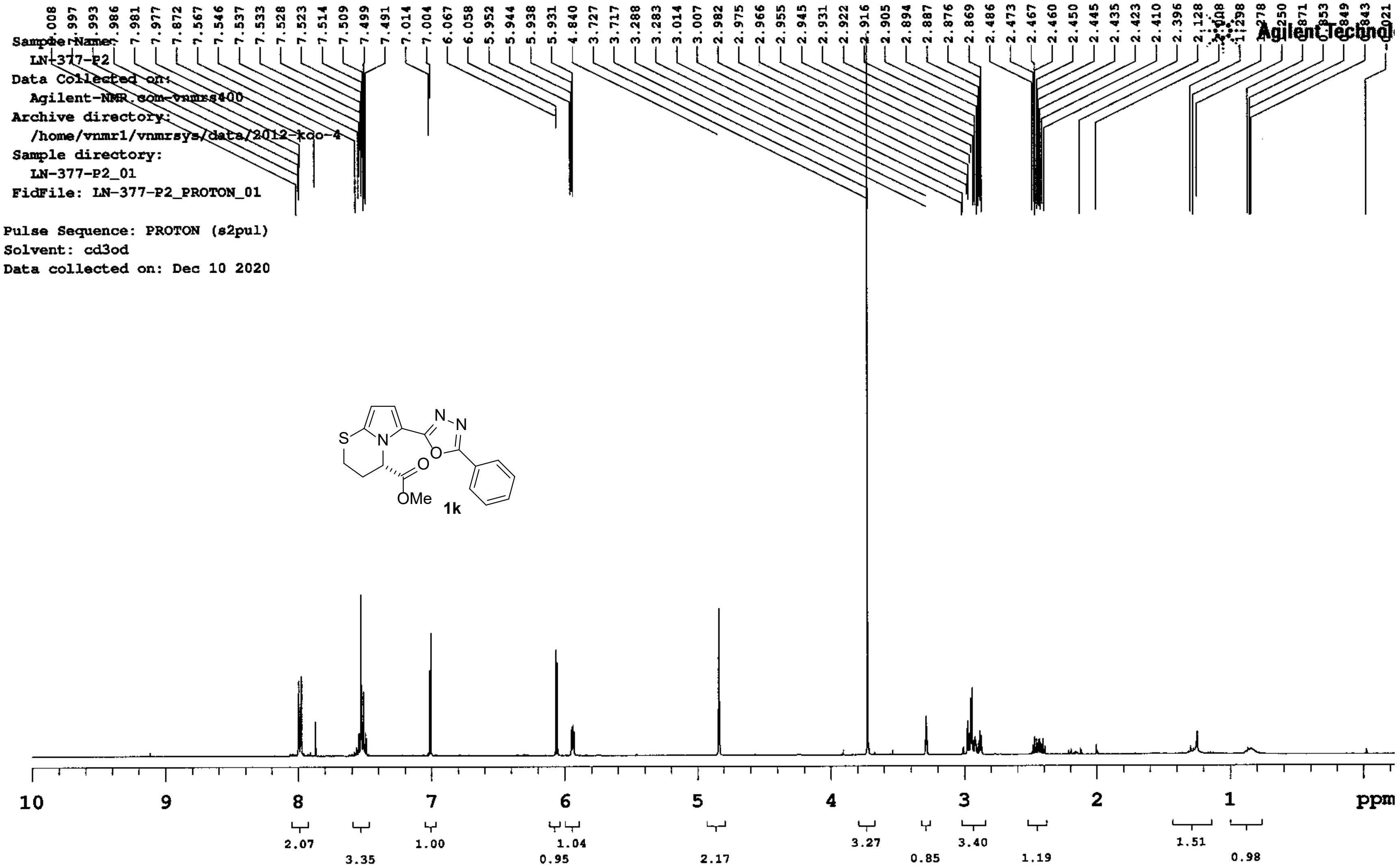
Sample Name:
LN-377-P2
Data Collected on:
Agilent-NMR.com-vnmrs400
Archive directory:
/home/vnmr1/vnmrsys/data/2012-kco-4
Sample directory:
LN-377-P2_01
FidFile: LN-377-P2_PROTON_01
Pulse Sequence: PROTON (s2pul)
Solvent: cd3od
Data collected on: Dec 10 2020
Agilent Technol
7.981 7.977 7.872 7.567 7.546 7.537 7.533 7.528 7.523 7.514 7.509 7.499 7.491 7.014 7.004 6.067 6.058 5.952 5.944 5.938 5.931 4.840 3.727 3.717 3.288 3.283 3.014 3.007 2.982 2.975 2.966 2.955 2.945 2.931 2.922 2.916 2.905 2.894 2.887 2.876 2.869 2.486 2.473 2.467 2.460 2.450 2.445 2.435 2.423 2.410 2.396 2.128
S
N
N
N
O
O
OMe
1k
10
9
8
7
6
5
4
3
2
1
ppm
2.07
3.35
1.00
0.95
1.04
2.17
3.27
0.85
3.40
1.19
1.51
0.98

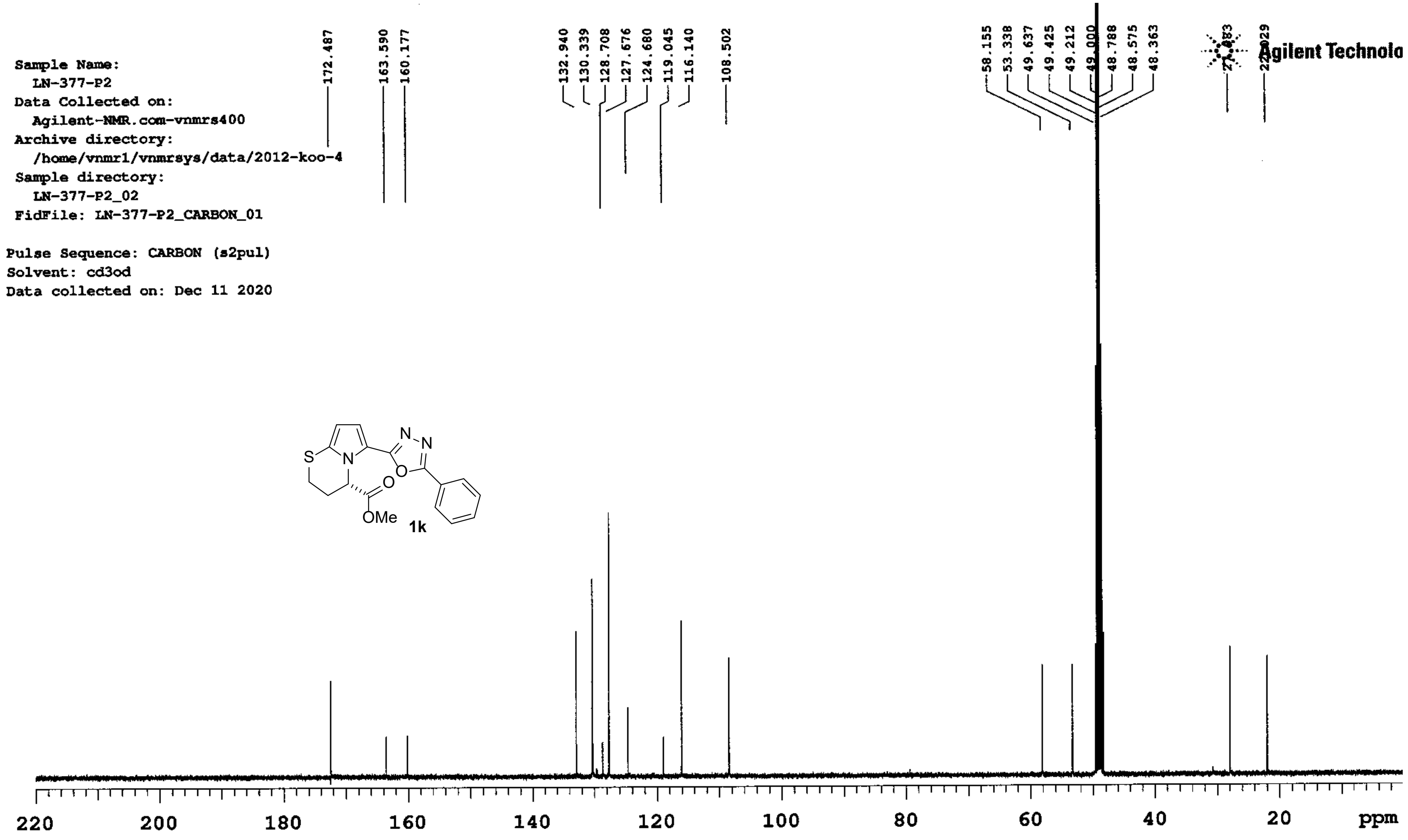
Sample Name:
LN-377-P2
Data Collected on:
Agilent-NMR.com-vnmrs400
Archive directory:
/home/vnmr1/vnmrsys/data/2012-koo-4
Sample directory:
LN-377-P2_02
FidFile: LN-377-P2_CARBON_01
Pulse Sequence: CARBON (s2pul)
Solvent: cd3od
Data collected on: Dec 11 2020
172.487
163.590
160.177
132.940
130.339
128.708
127.676
124.680
119.045
116.140
108.502
58.155
53.338
49.637
49.425
49.212
49.000
48.788
48.575
48.363
Agilent Technolo
S
N
N
N
O
O
OMe
1k
220
200
180
160
140
120
100
80
60
40
20
ppm

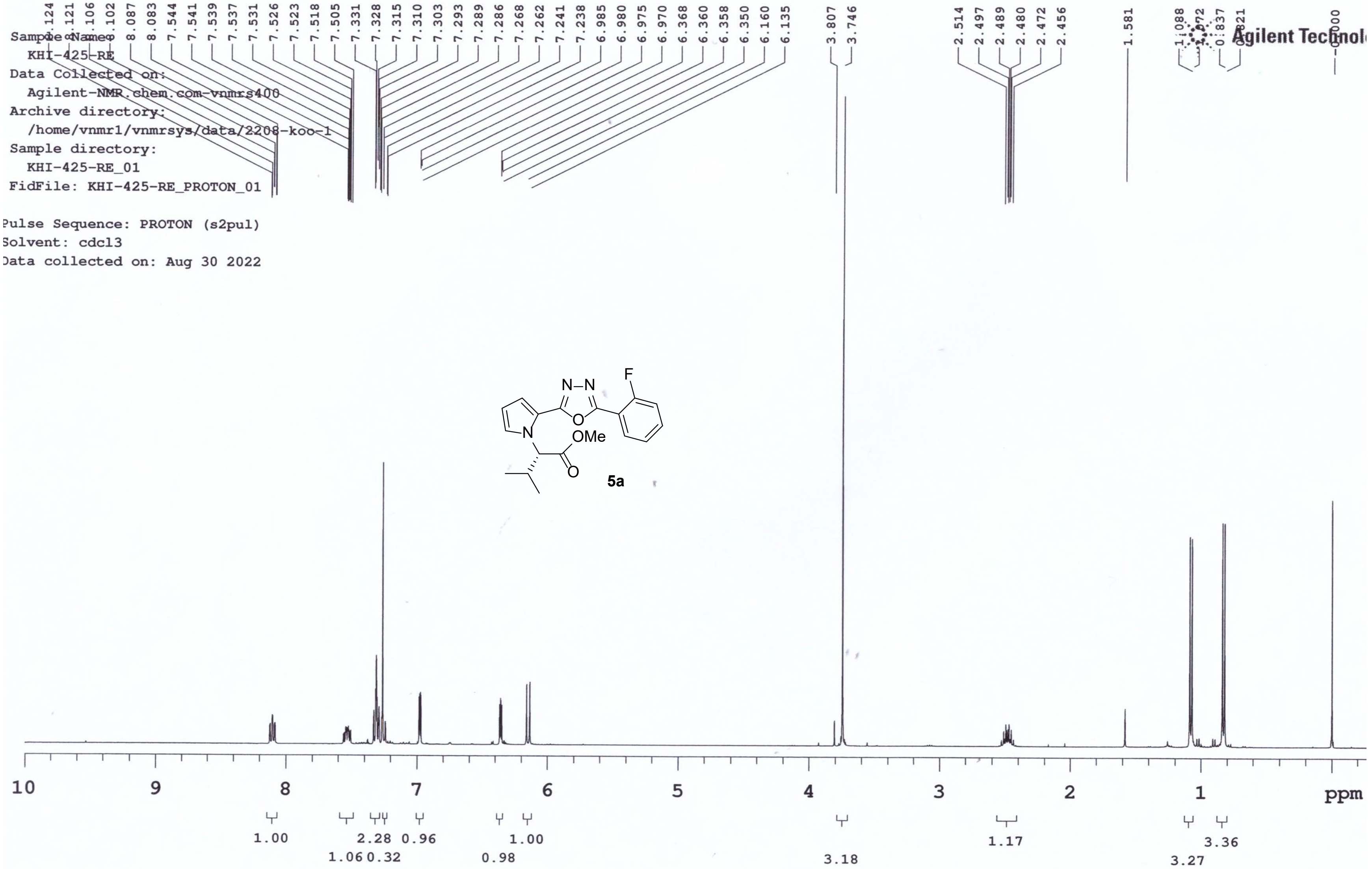
Sample Name:
KHI-425-RE
Data Collected on:
Agilent-NMR.chem.com-vnmrs400
Archive directory:
/home/vnmr1/vnmrsys/data/2208-koo-1
Sample directory:
KHI-425-RE_01
FidFile: KHI-425-RE_PROTON_01
Pulse Sequence: PROTON (s2pul)
Solvent: cdcl3
Data collected on: Aug 30 2022
Agilent Technol
8.124 8.121 8.106 8.102 8.087 8.083 7.544 7.541 7.539 7.537 7.531 7.526 7.523 7.518 7.505 7.331 7.328 7.315 7.310 7.303 7.293 7.289 7.286 7.268 7.262 7.241 7.238 6.985 6.980 6.975 6.970 6.368 6.360 6.358 6.350 6.160 6.135
3.807 3.746
2.514 2.497 2.489 2.480 2.472 2.456
1.581
1.088 0.837 0.821
N–N
F
N
O
OMe
O
5a
10 9 8 7 6 5 4 3 2 1 ppm
1.00 1.06 2.28 0.32 0.96 0.98 1.00 3.18 1.17 3.27 3.36

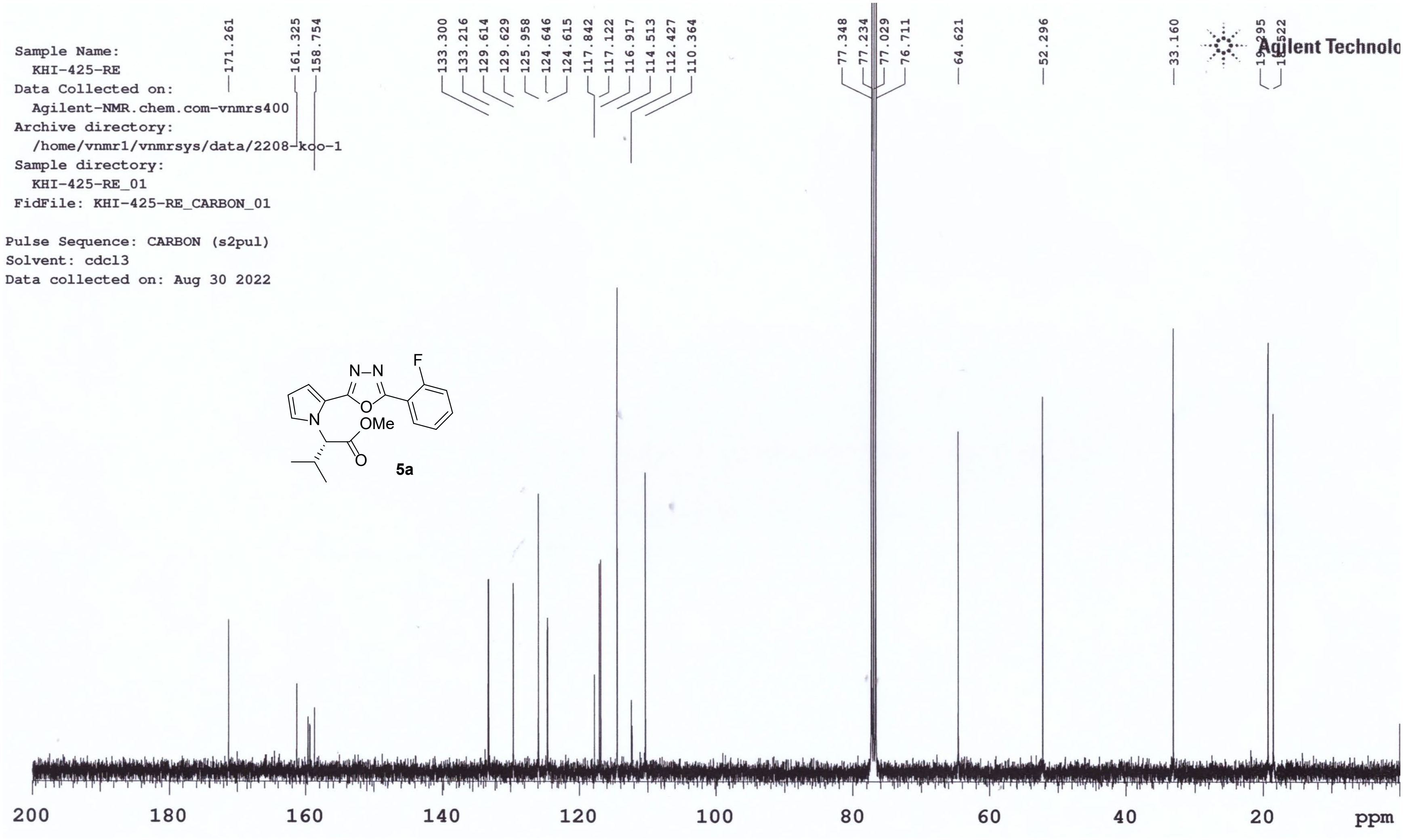
Sample Name:
KHI-425-RE
Data Collected on:
Agilent-NMR.chem.com-vnmrs400
Archive directory:
/home/vnmr1/vnmrsys/data/2208-koo-1
Sample directory:
KHI-425-RE_01
FidFile: KHI-425-RE_CARBON_01
Pulse Sequence: CARBON (s2pul)
Solvent: cdcl3
Data collected on: Aug 30 2022
171.261
161.325
158.754
133.300
133.216
129.614
129.629
125.958
124.646
124.615
117.842
117.122
116.917
114.513
112.427
110.364
77.348
77.234
77.029
76.711
64.621
52.296
33.160
19.295
18.522
Agilent Technolo
N–N
F
O
N
OMe
O
5a
200
180
160
140
120
100
80
60
40
20
ppm

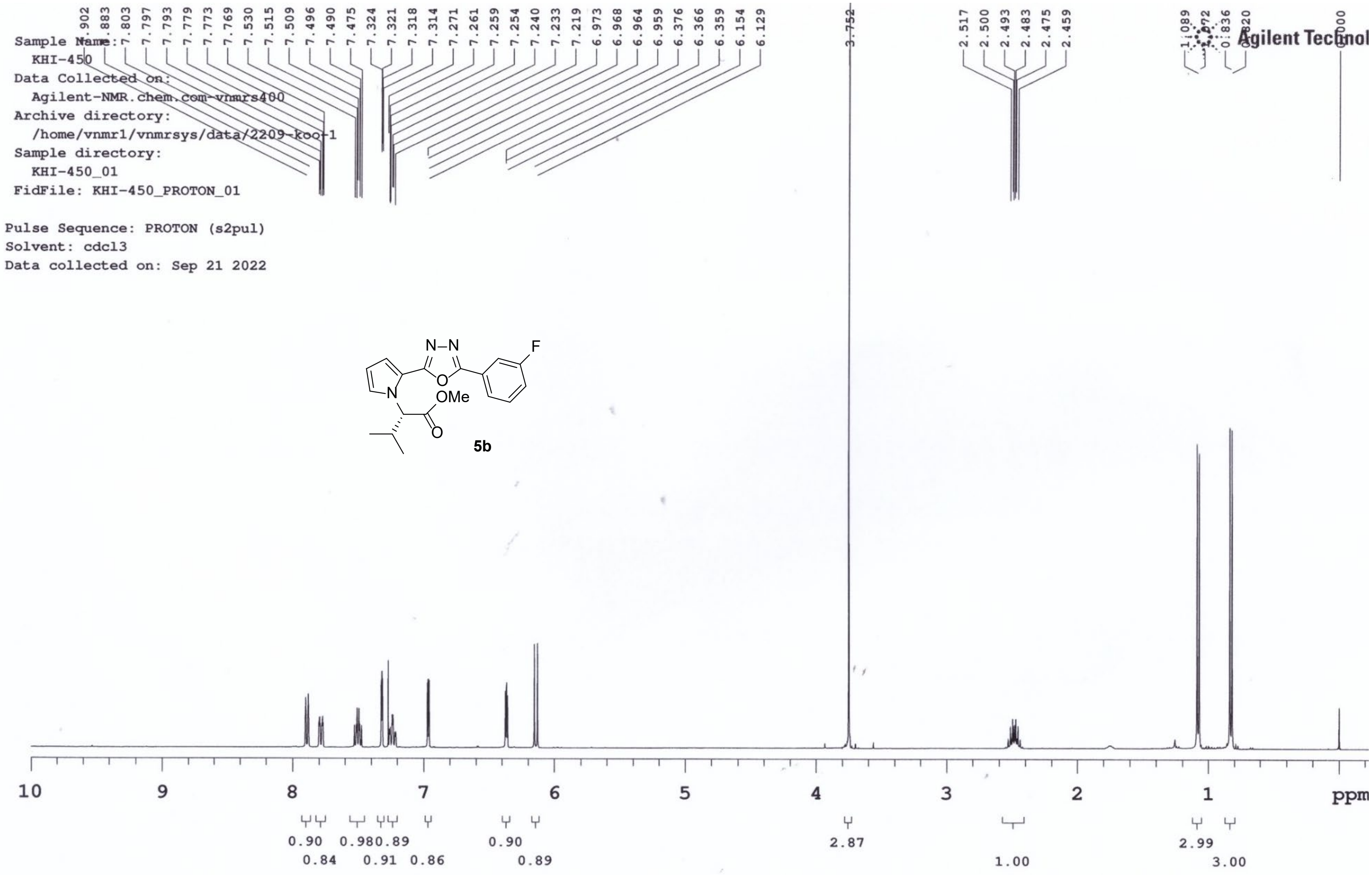
Sample Name:
KHI-450
Data Collected on:
Agilent-NMR.chem.com-vnmrs400
Archive directory:
/home/vnmr1/vnmrsys/data/2209-koo-1
Sample directory:
KHI-450_01
FidFile: KHI-450_PROTON_01
Pulse Sequence: PROTON (s2pul)
Solvent: cdcl3
Data collected on: Sep 21 2022
Agilent Technol
7.902 7.883 7.803 7.797 7.793 7.779 7.773 7.769 7.530 7.515 7.509 7.496 7.490 7.475 7.324 7.321 7.318 7.314 7.271 7.261 7.259 7.254 7.240 7.233 7.219 6.973 6.968 6.964 6.959 6.376 6.366 6.359 6.154 6.129
3.752
2.517 2.500 2.493 2.483 2.475 2.459
1.089 1.072 0.836 0.820
0.000
N–N
F
O
N
OMe
O
5b
10 9 8 7 6 5 4 3 2 1 ppm
0.90 0.84 0.98 0.91 0.89 0.86 0.90 0.89 2.87 1.00 2.99 3.00

Sample Name:
KHI-450-13C
Data Collected on:
Agilent-NMR.chem.com-vnmrs400
Archive directory:
/home/vnmr1/vnmrsys/data/2209-koo-1
Sample directory:
KHI-450-13C_01
FidFile: KHI-450-13C_CARBON_01

Pulse Sequence: CARBON (s2pul)
Solvent: cdcl3
Data collected on: Sep 22 2022

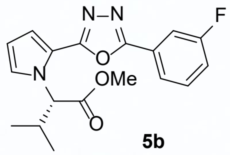

5b

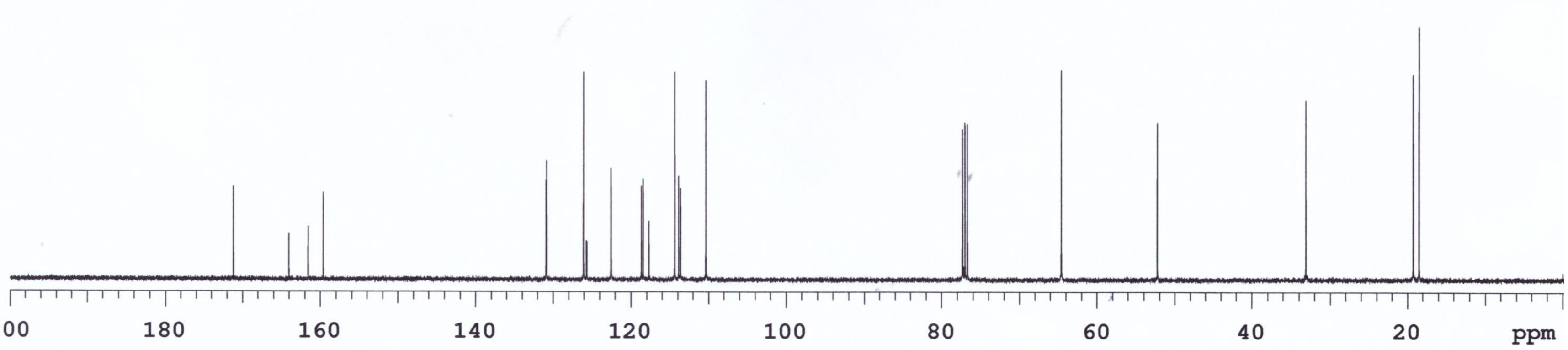

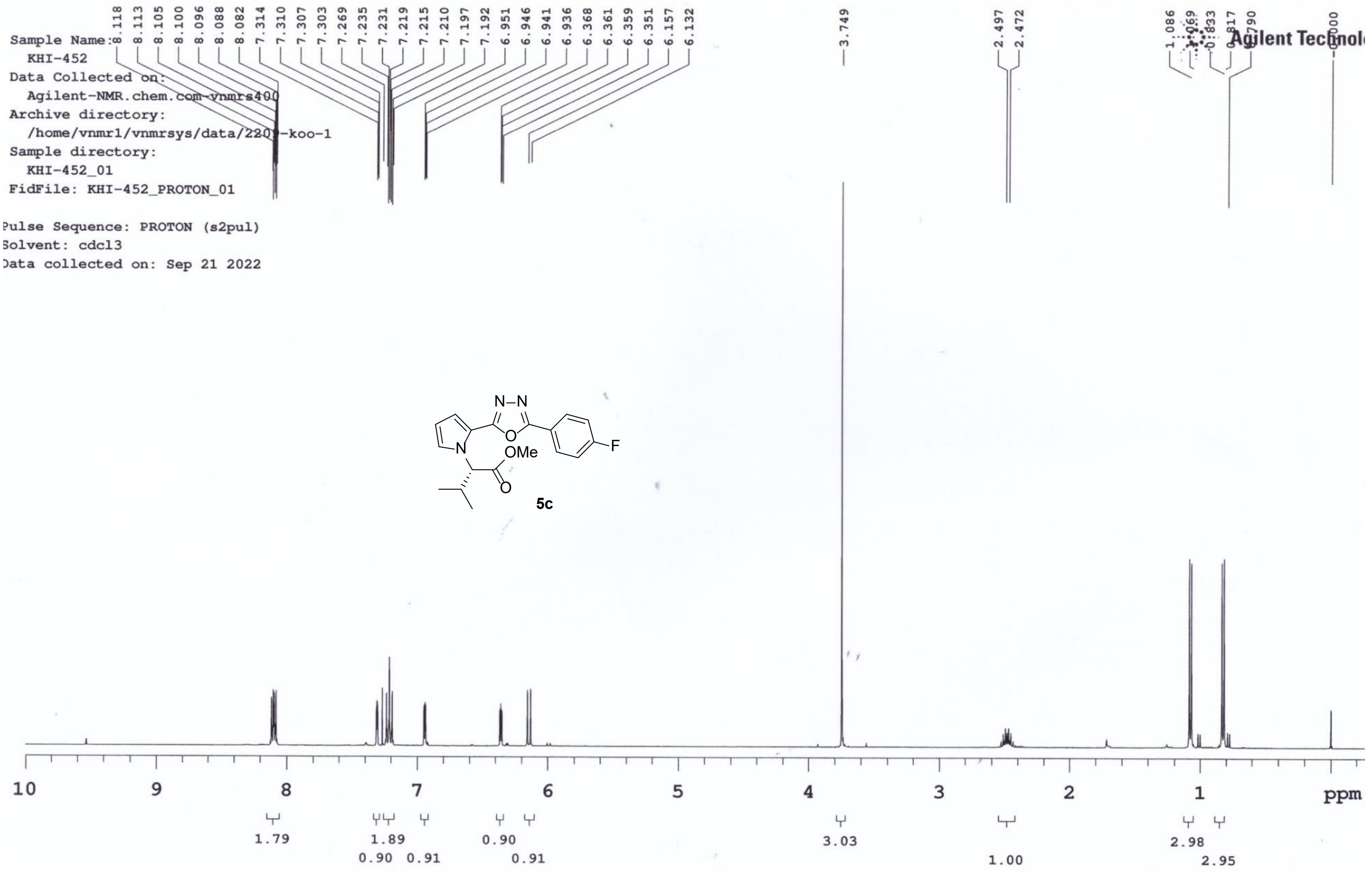
Sample Name:
KHI-452
Data Collected on:
Agilent-NMR.chem.com-vnmrs400
Archive directory:
/home/vnmr1/vnmrsys/data/2209-koo-1
Sample directory:
KHI-452_01
FidFile: KHI-452_PROTON_01
Pulse Sequence: PROTON (s2pul)
Solvent: cdcl3
Data collected on: Sep 21 2022
8.118 8.113 8.105 8.100 8.096 8.088 8.082 7.314 7.310 7.307 7.303 7.269 7.235 7.231 7.219 7.215 7.210 7.197 7.192 6.951 6.946 6.941 6.936 6.368 6.361 6.359 6.351 6.157 6.132
3.749
2.497 2.472
1.086 0.817 0.790 0.000
Agilent Technol
N–N
O
F
N
OMe
O
5c
10 9 8 7 6 5 4 3 2 1 ppm
1.79 1.89 0.90 0.91 0.90 0.91 3.03 1.00 2.98 2.95

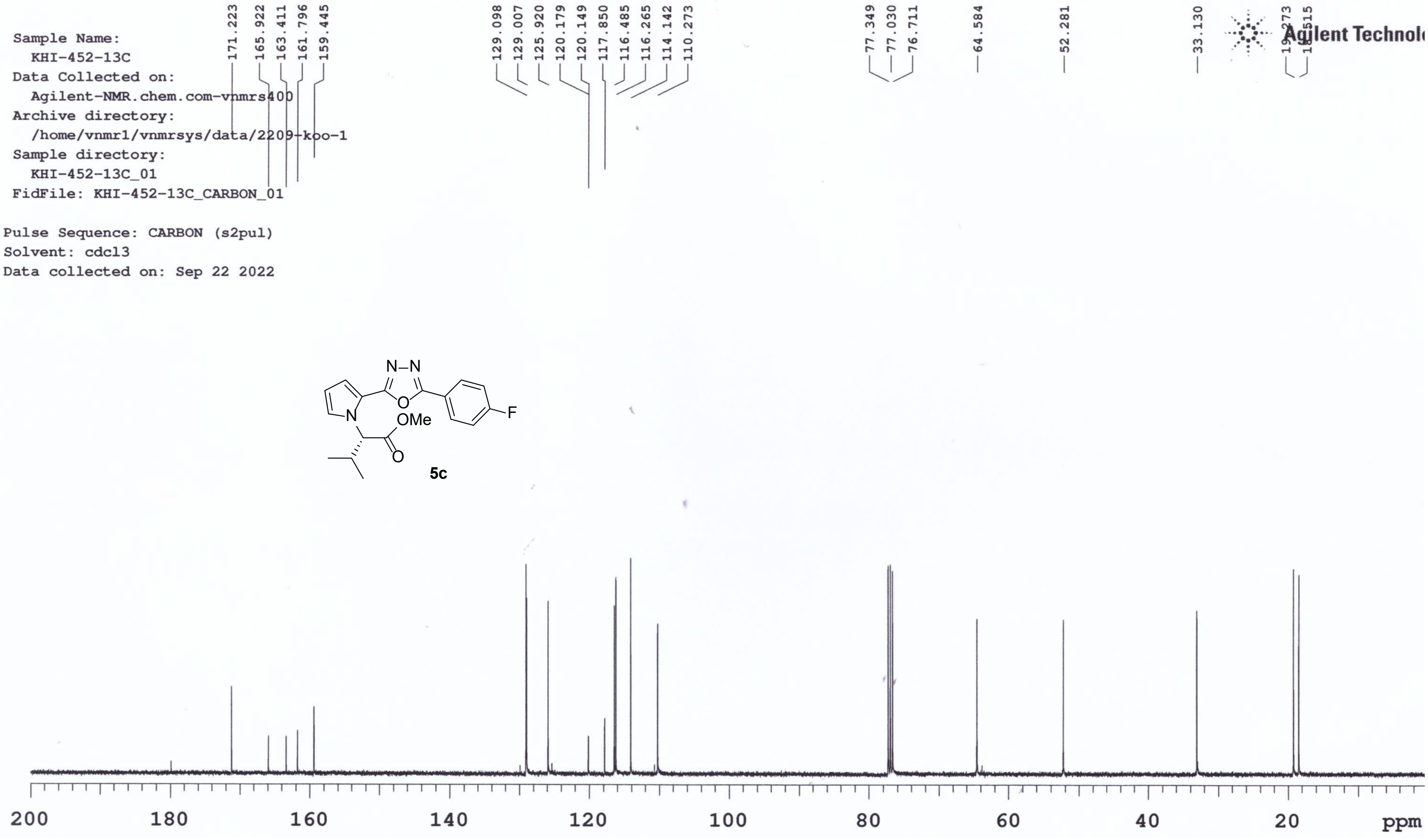
Sample Name:
KHI-452-13C
Data Collected on:
Agilent-NMR.chem.com-vnmrs400
Archive directory:
/home/vnmr1/vnmrsys/data/2209-koo-1
Sample directory:
KHI-452-13C_01
FidFile: KHI-452-13C_CARBON_01
Pulse Sequence: CARBON (s2pul)
Solvent: cdcl3
Data collected on: Sep 22 2022
171.223
165.922
163.411
161.796
159.445
129.098
129.007
125.920
120.179
120.149
117.850
116.485
116.265
114.142
110.273
77.349
77.030
76.711
64.584
52.281
33.130
OMe
O
N–N
N
F
5c
200
180
160
140
120
100
80
60
40
20
ppm

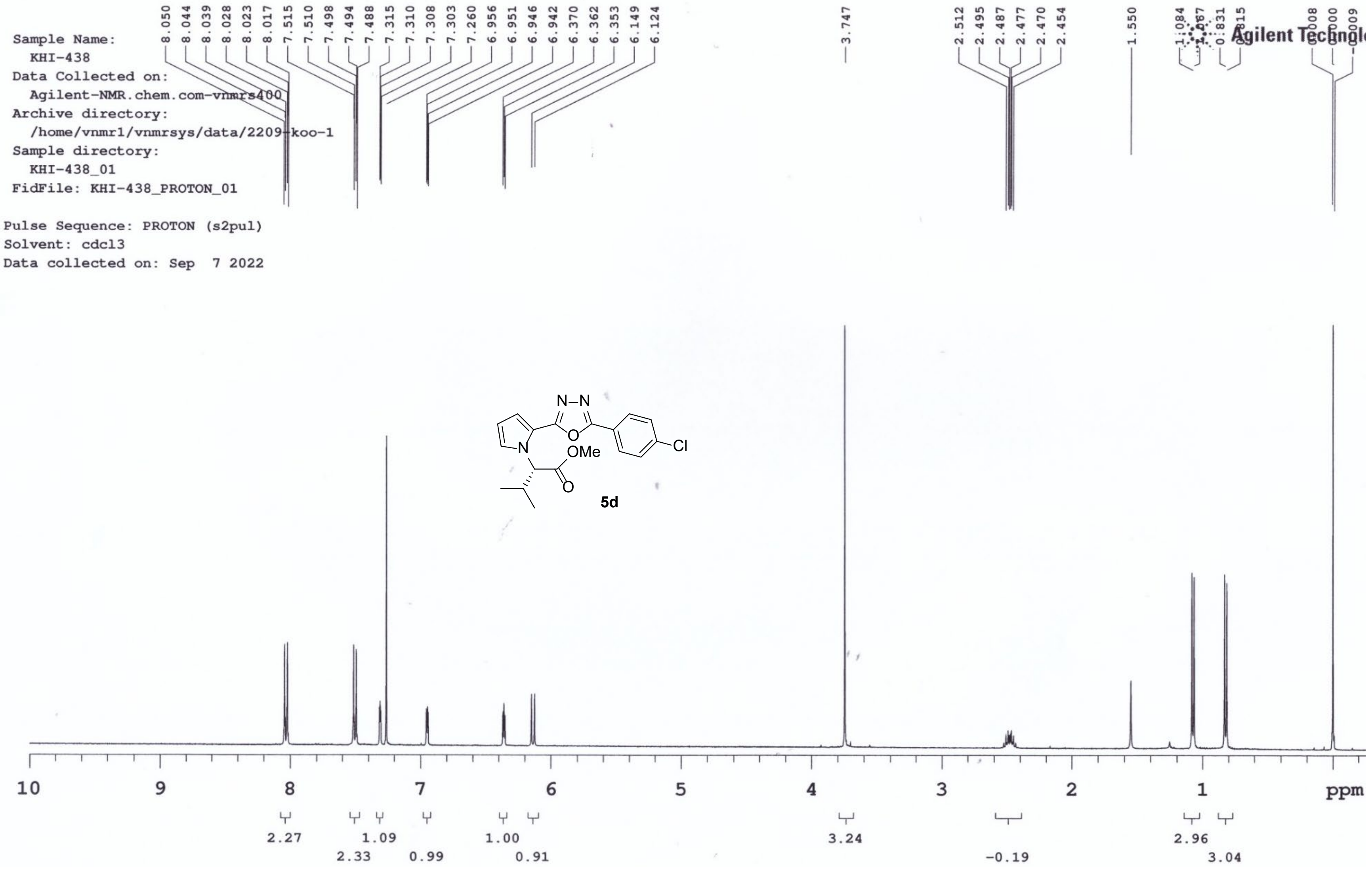
Sample Name:
KHI-438
Data Collected on:
Agilent-NMR.chem.com-vnmrs400
Archive directory:
/home/vnmr1/vnmrsys/data/2209-koo-1
Sample directory:
KHI-438_01
FidFile: KHI-438_PROTON_01
Pulse Sequence: PROTON (s2pul)
Solvent: cdcl3
Data collected on: Sep 7 2022
8.050 8.044 8.039 8.028 8.023 8.017 7.515 7.510 7.498 7.494 7.488 7.315 7.310 7.308 7.303 7.260 6.956 6.951 6.946 6.942 6.370 6.362 6.353 6.149 6.124
3.747
2.512 2.495 2.487 2.477 2.470 2.454
1.550
0.831
N–N
O
OMe
N
O
Cl
5d
10 9 8 7 6 5 4 3 2 1 ppm
2.27 2.33 1.09 0.99 1.00 0.91 3.24 -0.19 2.96 3.04

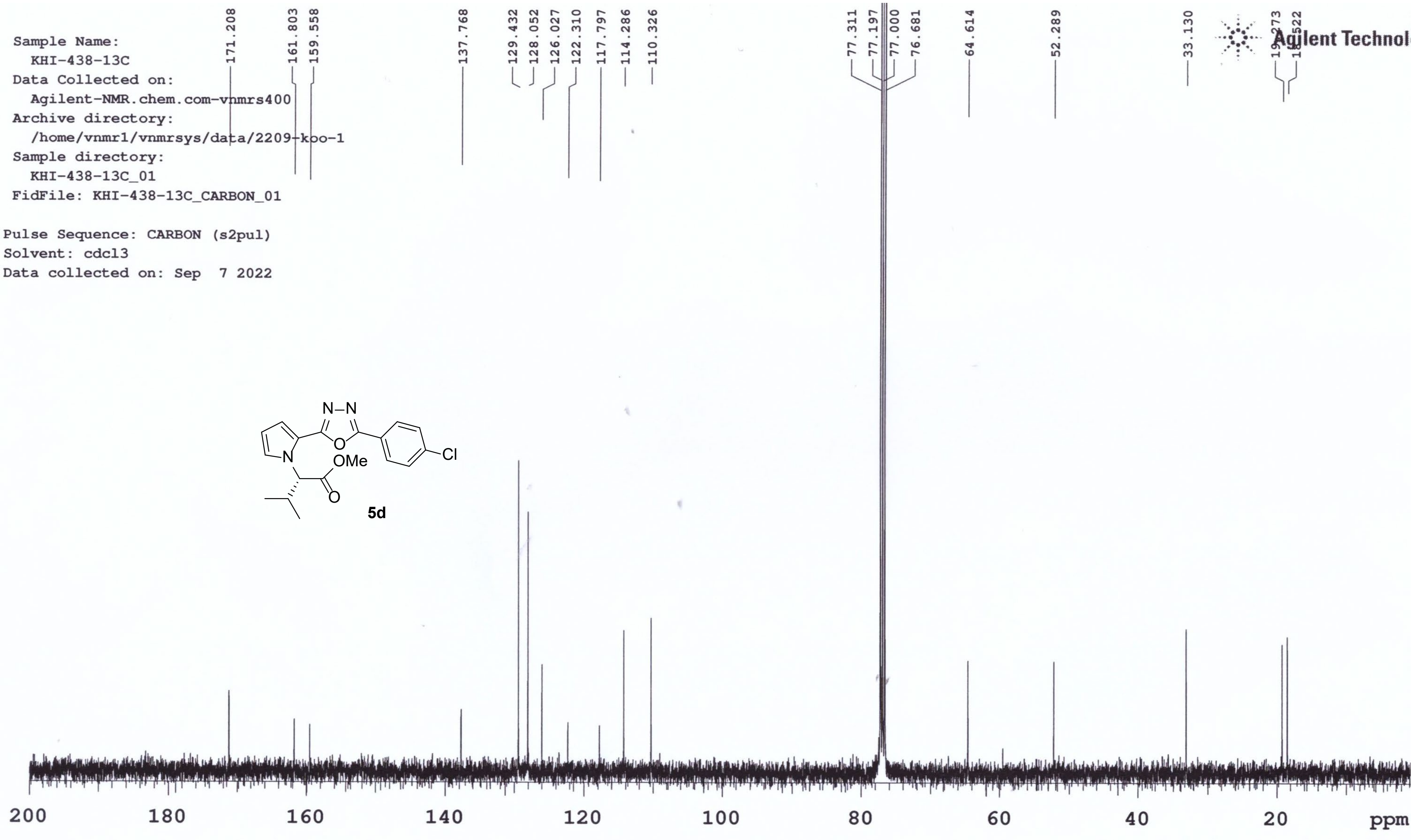
Sample Name:
KHI-438-13C
Data Collected on:
Agilent-NMR.chem.com-vnmrs400
Archive directory:
/home/vnmr1/vnmrsys/data/2209-koo-1
Sample directory:
KHI-438-13C_01
FidFile: KHI-438-13C_CARBON_01
Pulse Sequence: CARBON (s2pul)
Solvent: cdcl3
Data collected on: Sep 7 2022
171.208
161.803
159.558
137.768
129.432
128.052
126.027
122.310
117.797
114.286
110.326
77.311
77.197
77.000
76.681
64.614
52.289
33.130
19.273
18.522
Agilent Technol
N–N
O
N
OMe
O
Cl
5d
200
180
160
140
120
100
80
60
40
20
ppm

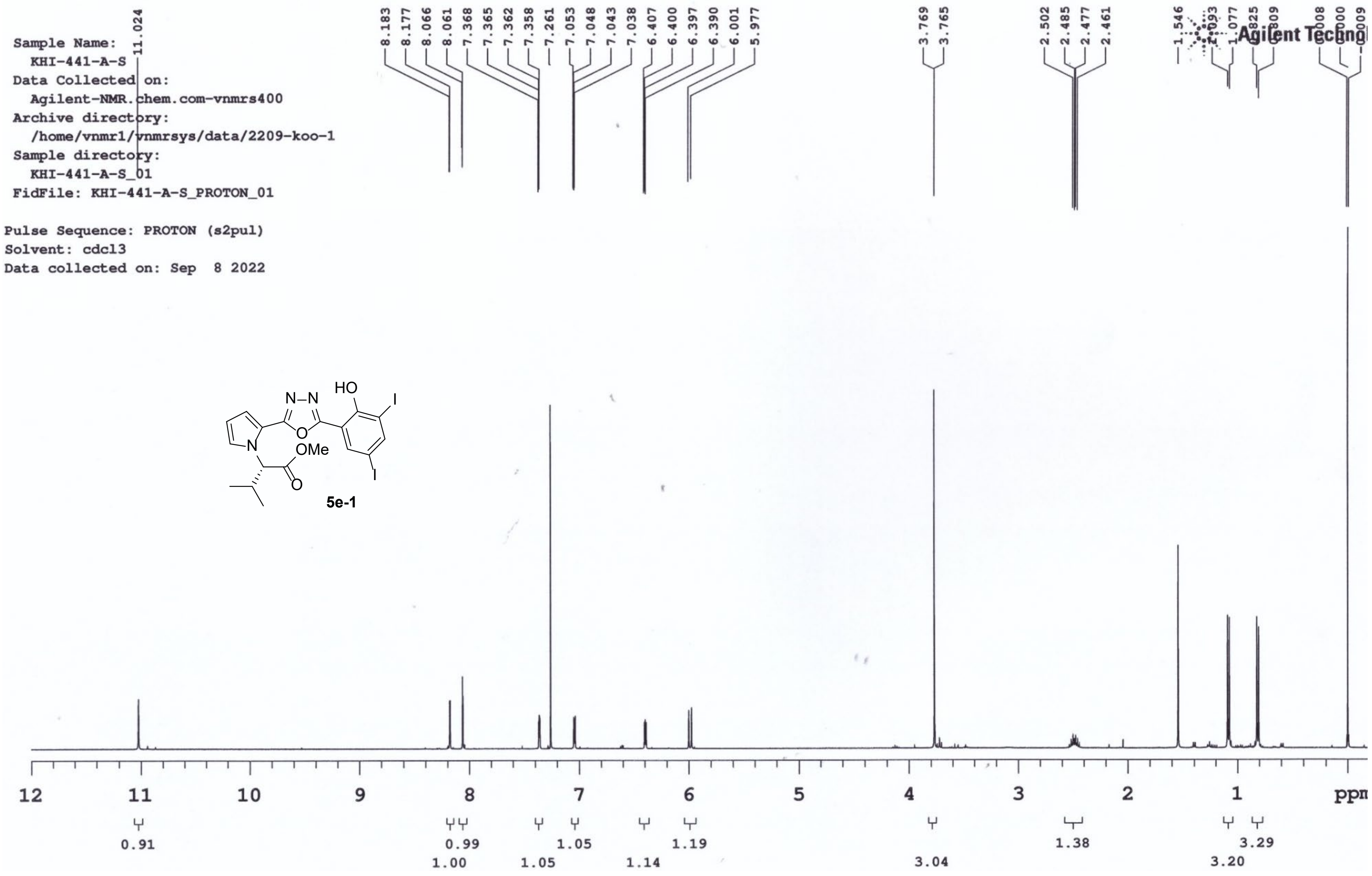

Sample Name:
KHI-441-A-S
Data Collected on:
Agilent-NMR.chem.com-vnmrs400
Archive directory:
/home/vnmr1/vnmrsys/data/2209-koo-1
Sample directory:
KHI-441-A-S_01
FidFile: KHI-441-A-S_PROTON_01
Pulse Sequence: PROTON (s2pul)
Solvent: cdcl3
Data collected on: Sep 8 2022
11.024
8.183
8.177
8.066
8.061
7.368
7.365
7.362
7.358
7.261
7.053
7.048
7.043
7.038
6.407
6.400
6.397
6.390
6.001
5.977
3.769
3.765
2.502
2.485
2.477
2.461
1.546
1.077
Agilent Techn
HO
N–N
I
O
N
OMe
O
I
5e-1
12
11
10
9
8
7
6
5
4
3
2
1
ppm
0.91
0.99
1.00
1.05
1.05
1.14
1.19
3.04
1.38
3.20
3.29

Sample Name:
KHI-441-A-S-13C
Data Collected on:
Agilent-NMR.chem.com-vnmrs400
Archive directory:
/home/vnmr1/vnmrsys/data/2209-kco-1
Sample directory:
KHI-441-A-S-13C_01
FidFile: KHI-441-A-S-13C_CARBON_01

Pulse Sequence: CARBON (s2pul)
Solvent: cdcl3
Data collected on: Sep 13 2022

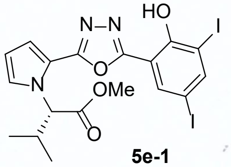

5e-1

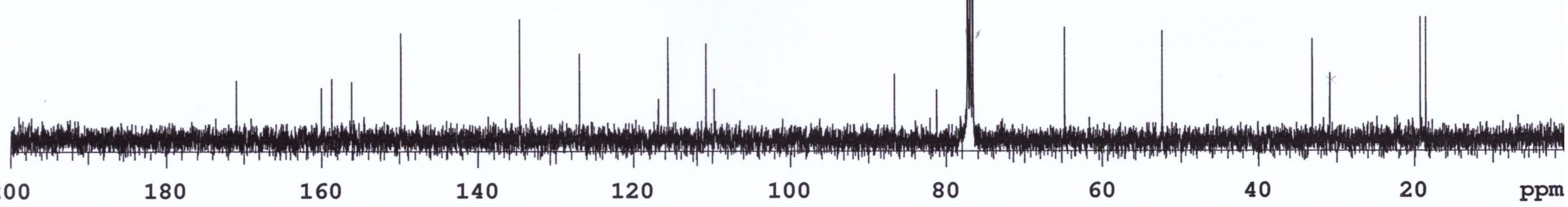

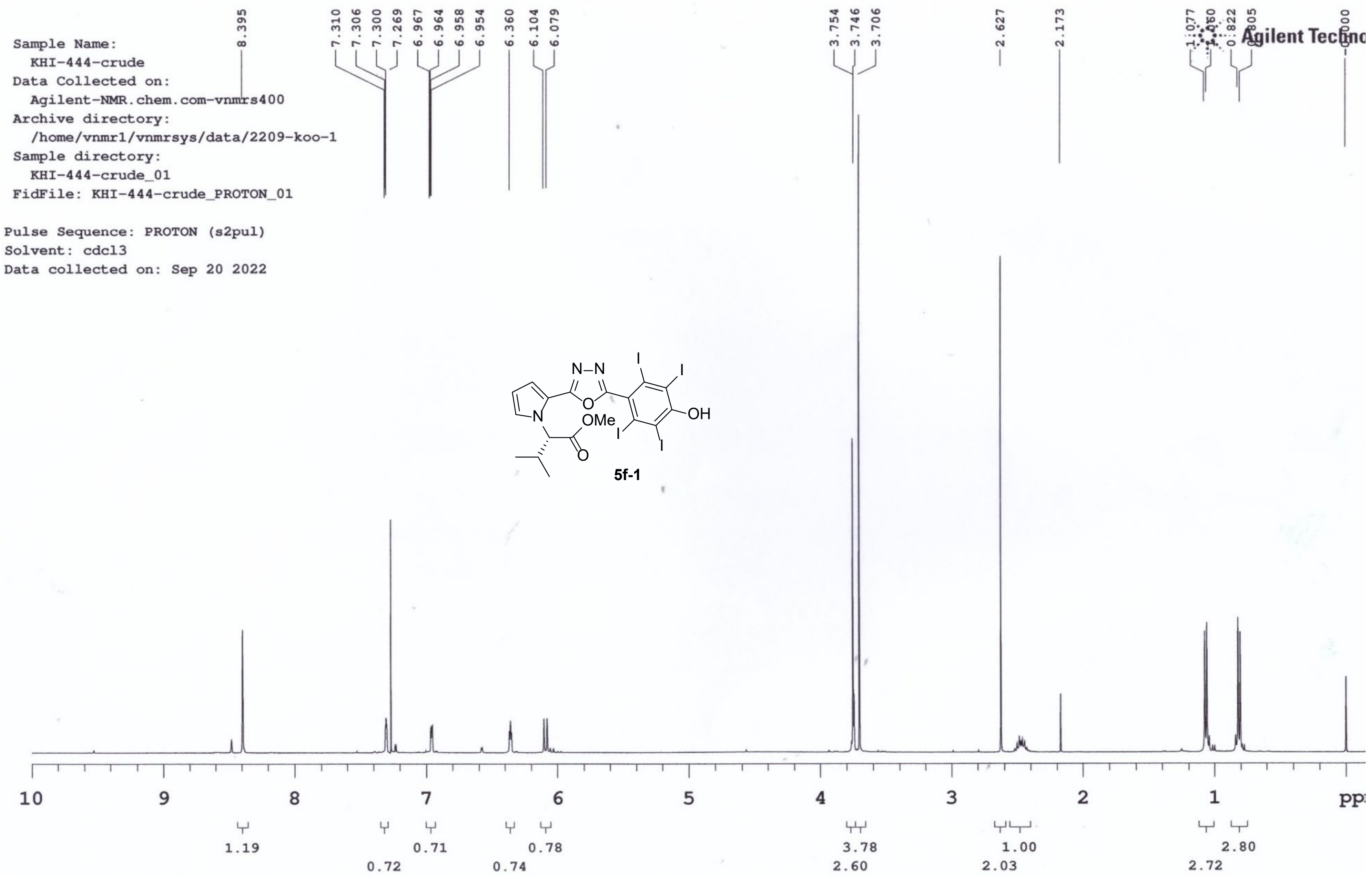
Sample Name:
KHI-444-crude
Data Collected on:
Agilent-NMR.chem.com-vnmrs400
Archive directory:
/home/vnmr1/vnmrsys/data/2209-koo-1
Sample directory:
KHI-444-crude_01
FidFile: KHI-444-crude_PROTON_01
Pulse Sequence: PROTON (s2pul)
Solvent: cdcl3
Data collected on: Sep 20 2022
8.395
7.310
7.306
7.300
7.269
6.967
6.964
6.958
6.954
6.360
6.104
6.079
3.754
3.746
3.706
2.627
2.173
0.000
Agilent Techno
N–N
O
N
OMe
O
I
I
I
I
OH
5f-1
10 9 8 7 6 5 4 3 2 1 pp
1.19
0.72
0.71
0.74
0.78
3.78
2.60
1.00
2.03
2.72
2.80

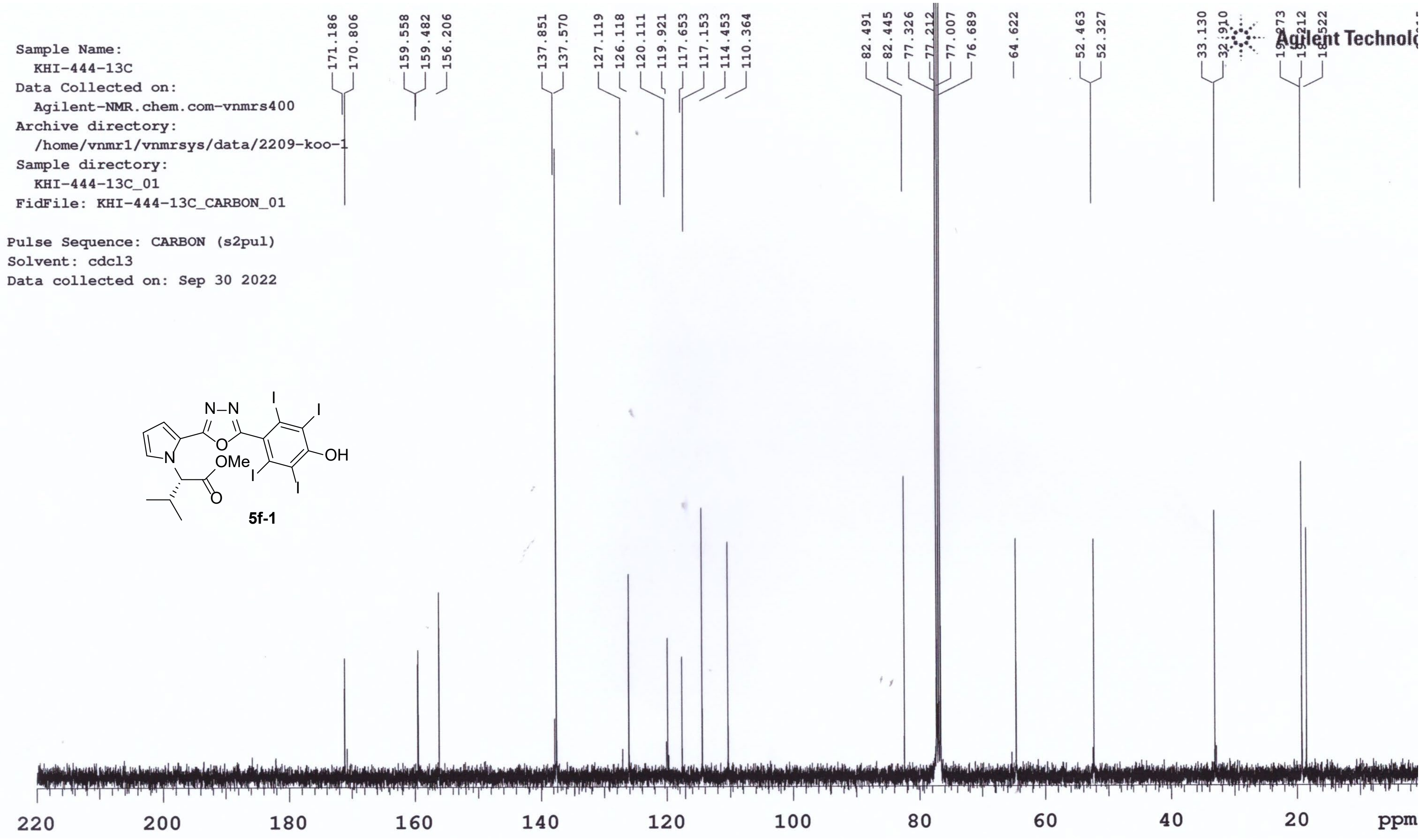
Sample Name:
KHI-444-13C
Data Collected on:
Agilent-NMR.chem.com-vnmrs400
Archive directory:
/home/vnmr1/vnmrsys/data/2209-koo-1
Sample directory:
KHI-444-13C_01
FidFile: KHI-444-13C_CARBON_01
Pulse Sequence: CARBON (s2pul)
Solvent: cdcl3
Data collected on: Sep 30 2022
171.186
170.806
159.558
159.482
156.206
137.851
137.570
127.119
126.118
120.111
119.921
117.653
117.153
114.453
110.364
82.491
82.445
77.326
77.212
77.007
76.689
64.622
52.463
52.327
33.130
5f-1
OMe
OH
220
200
180
160
140
120
100
80
60
40
20
ppm

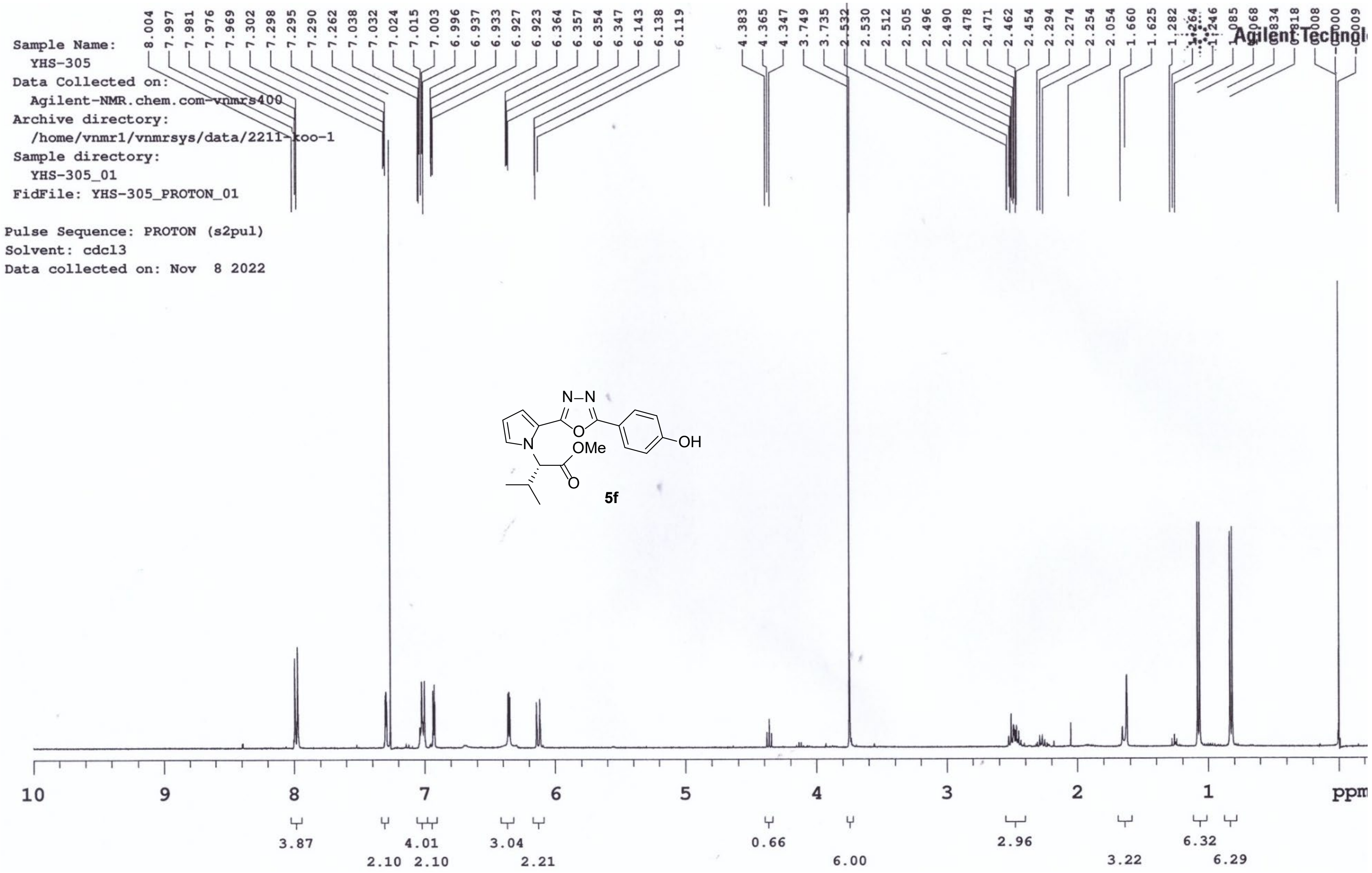
Sample Name:
YHS-305
Data Collected on:
Agilent-NMR.chem.com-vnmrs400
Archive directory:
/home/vnmr1/vnmrsys/data/2211-koo-1
Sample directory:
YHS-305_01
FidFile: YHS-305_PROTON_01
Pulse Sequence: PROTON (s2pul)
Solvent: cdcl3
Data collected on: Nov 8 2022
Agilent Technologies
8.004 7.997 7.981 7.976 7.969 7.302 7.298 7.295 7.290 7.262 7.038 7.032 7.024 7.015 7.003 6.996 6.937 6.933 6.927 6.923 6.364 6.357 6.354 6.347 6.143 6.138 6.119
4.383 4.365 4.347 3.749 3.735 2.532 2.530 2.512 2.505 2.496 2.490 2.478 2.471 2.462 2.454 2.294 2.274 2.254 2.054 1.660 1.625 1.282 1.264 1.246 1.085 1.068 0.834 0.818 0.008 0.000 -0.009
N–N
O
OMe
OH
N
O
5f
10 9 8 7 6 5 4 3 2 1 ppm
3.87 2.10 4.01 2.10 3.04 2.21 0.66 6.00 2.96 3.22 6.32 6.29

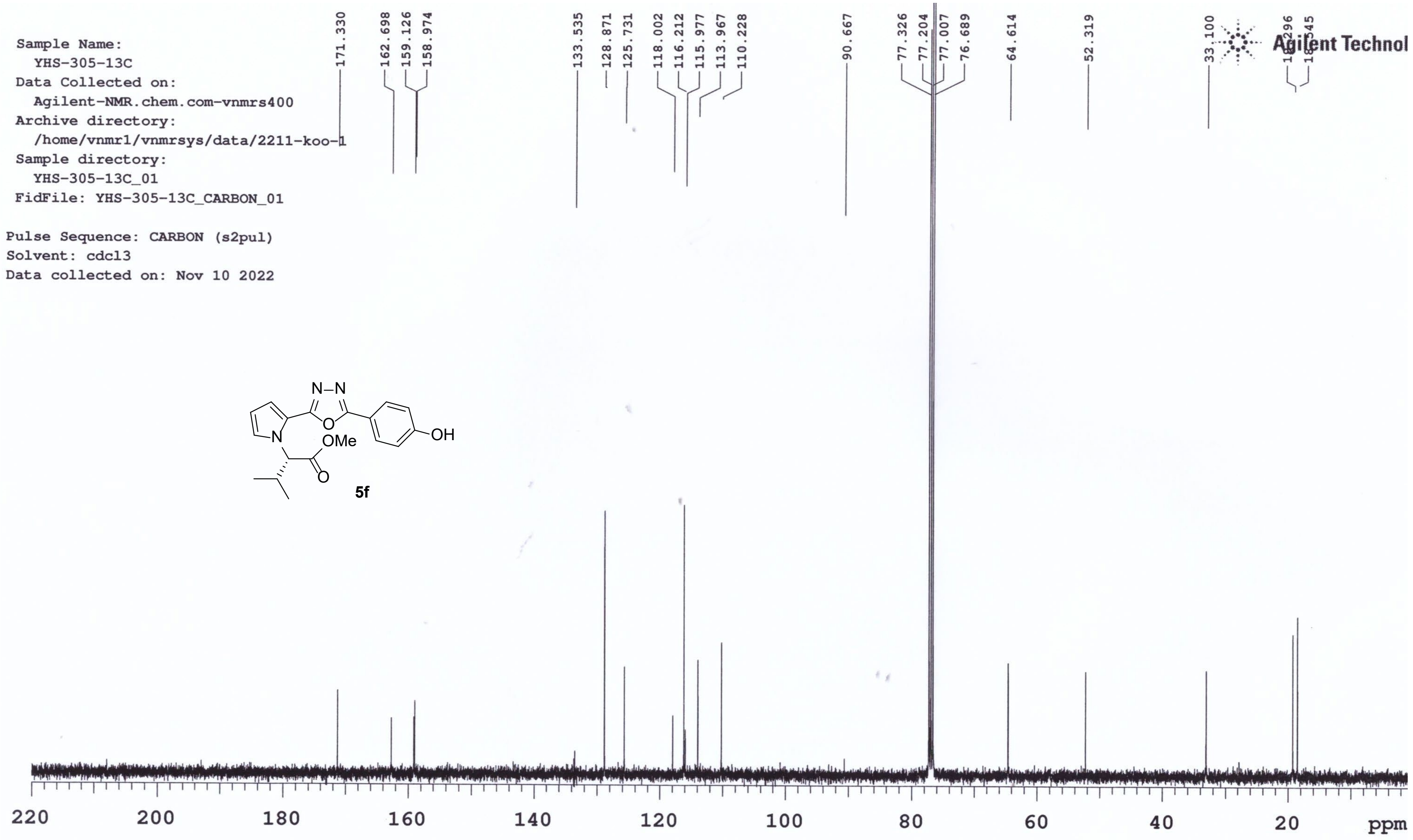

Sample Name:
YHS-305-13C
Data Collected on:
Agilent-NMR.chem.com-vnmrs400
Archive directory:
/home/vnmr1/vnmrsys/data/2211-koo-1
Sample directory:
YHS-305-13C_01
FidFile: YHS-305-13C_CARBON_01
Pulse Sequence: CARBON (s2pul)
Solvent: cdcl3
Data collected on: Nov 10 2022
171.330
162.698
159.126
158.974
133.535
128.871
125.731
118.002
116.212
115.977
113.967
110.228
90.667
77.326
77.204
77.007
76.689
64.614
52.319
33.100
Agilent Technol
N–N
O
OMe
OH
N
O
5f
220
200
180
160
140
120
100
80
60
40
20
ppm

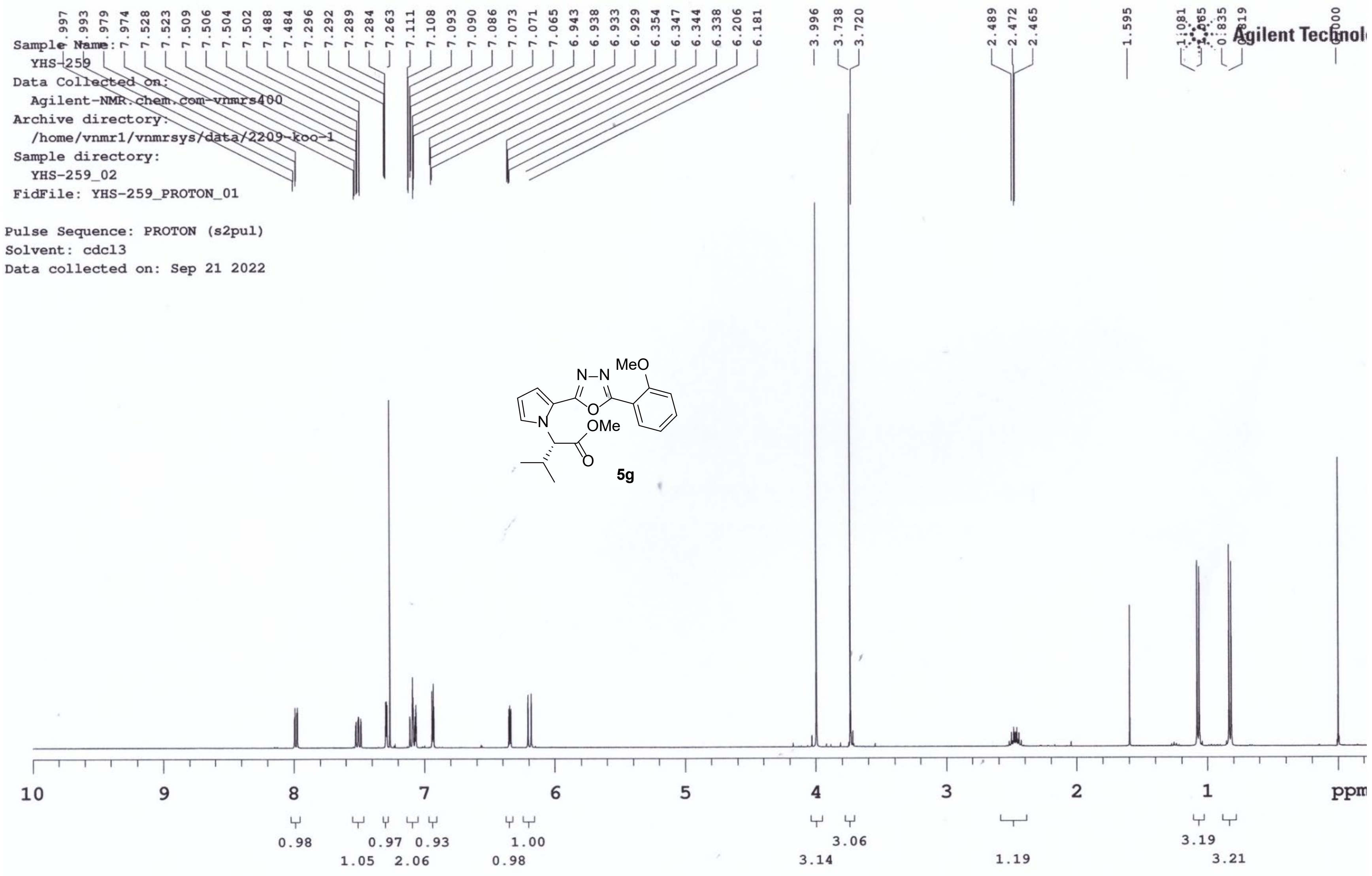
Sample Name:
YHS-259
Data Collected on:
Agilent-NMR.chem.com-vnmrs400
Archive directory:
/home/vnmr1/vnmrsys/data/2209-koo-1
Sample directory:
YHS-259_02
FidFile: YHS-259_PROTON_01
Pulse Sequence: PROTON (s2pul)
Solvent: cdcl3
Data collected on: Sep 21 2022
Agilent Technol
7.997 7.993 7.979 7.974 7.528 7.523 7.509 7.506 7.504 7.502 7.488 7.484 7.296 7.292 7.289 7.284 7.263 7.111 7.108 7.093 7.090 7.086 7.073 7.071 7.065 6.943 6.938 6.933 6.929 6.354 6.347 6.344 6.338 6.206 6.181 3.996 3.738 3.720 2.489 2.472 2.465 1.595 1.081 1.065 0.835 0.819 0.000
N–N
MeO
O
OMe
N
O
5g
10 9 8 7 6 5 4 3 2 1 ppm
0.98 1.05 0.97 2.06 0.93 0.98 1.00 3.14 3.06 1.19 3.19 3.21

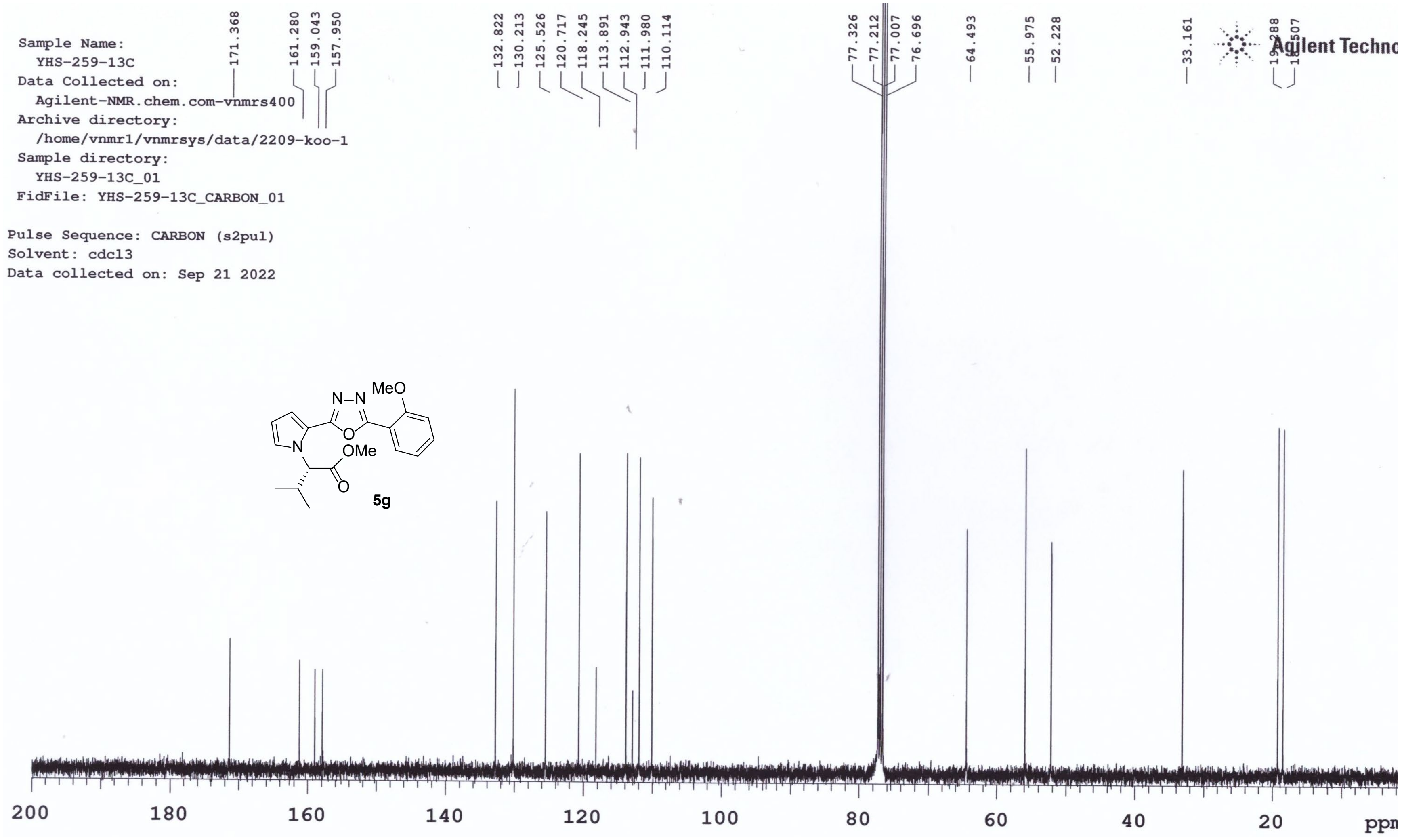
Sample Name:
YHS-259-13C
Data Collected on:
Agilent-NMR.chem.com-vnmrs400
Archive directory:
/home/vnmr1/vnmrsys/data/2209-koo-1
Sample directory:
YHS-259-13C_01
FidFile: YHS-259-13C_CARBON_01
Pulse Sequence: CARBON (s2pul)
Solvent: cdcl3
Data collected on: Sep 21 2022
171.368
161.280
159.043
157.950
132.822
130.213
125.526
120.717
118.245
113.891
112.943
111.980
110.114
77.326
77.212
77.007
76.696
64.493
55.975
52.228
33.161
19.288
18.507
Agilent Techno
N–N
MeO
N
OMe
O
O
5g
200
180
160
140
120
100
80
60
40
20
ppm

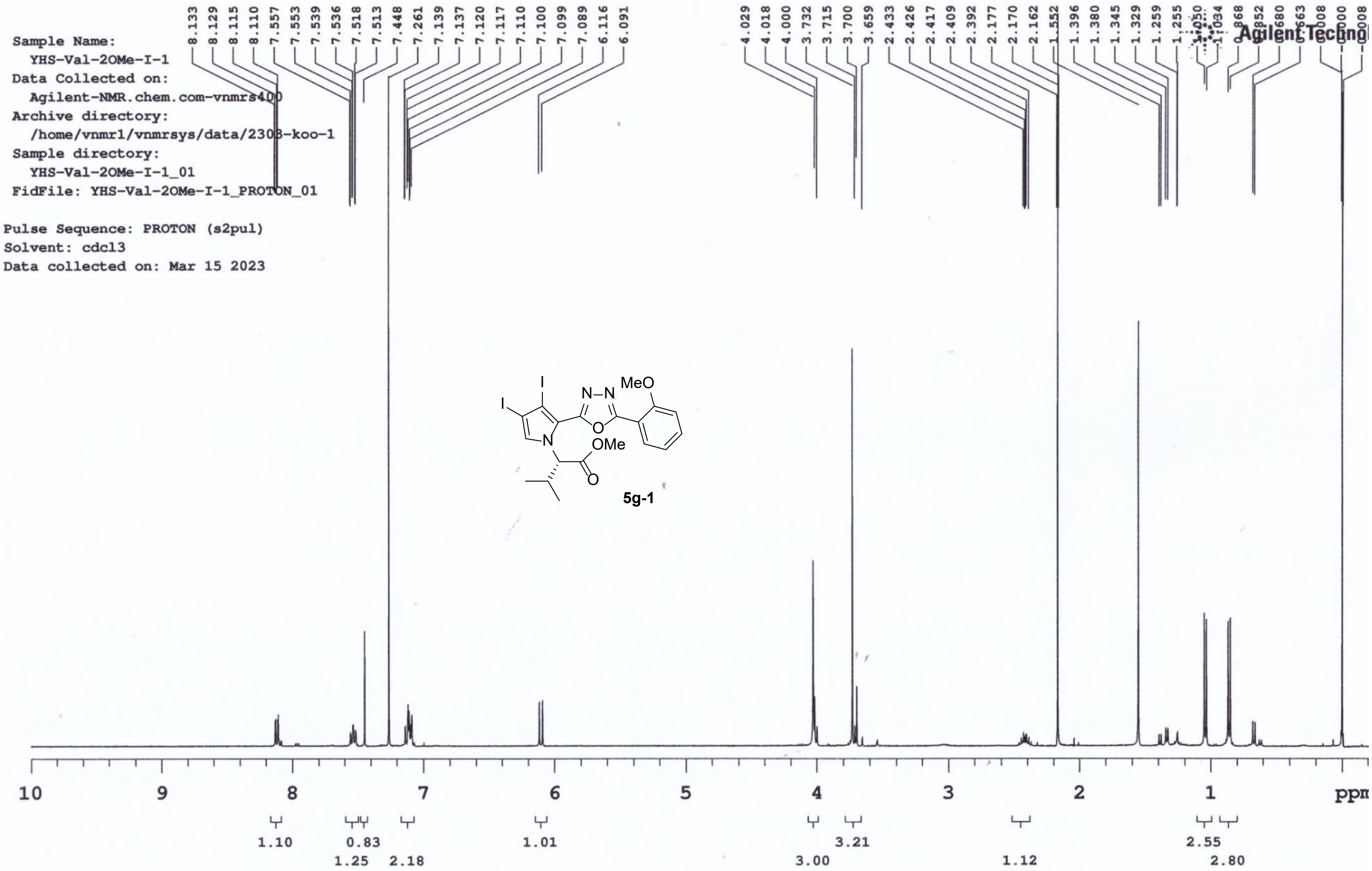
Sample Name:
YHS-Val-2OMe-I-1
Data Collected on:
Agilent-NMR.chem.com-vnmrs400
Archive directory:
/home/vnmr1/vnmrsys/data/2303-koo-1
Sample directory:
YHS-Val-2OMe-I-1_01
FidFile: YHS-Val-2OMe-I-1_PROTON_01
Pulse Sequence: PROTON (s2pul)
Solvent: cdcl3
Data collected on: Mar 15 2023
8.133 8.129 8.115 8.110 7.557 7.553 7.539 7.536 7.518 7.513 7.448 7.261 7.139 7.137 7.120 7.117 7.110 7.100 7.099 7.089 6.116 6.091
4.029 4.018 4.000 3.732 3.715 3.700 3.659 2.433 2.426 2.417 2.409 2.392 2.177 2.170 2.162 1.552 1.396 1.380 1.345 1.329 1.255
Agilent Technol
I
I
N–N
MeO
O
N
OMe
O
5g-1
10 9 8 7 6 5 4 3 2 1 ppm
1.10 1.25 0.83 2.18 1.01 3.00 3.21 1.12 2.55 2.80

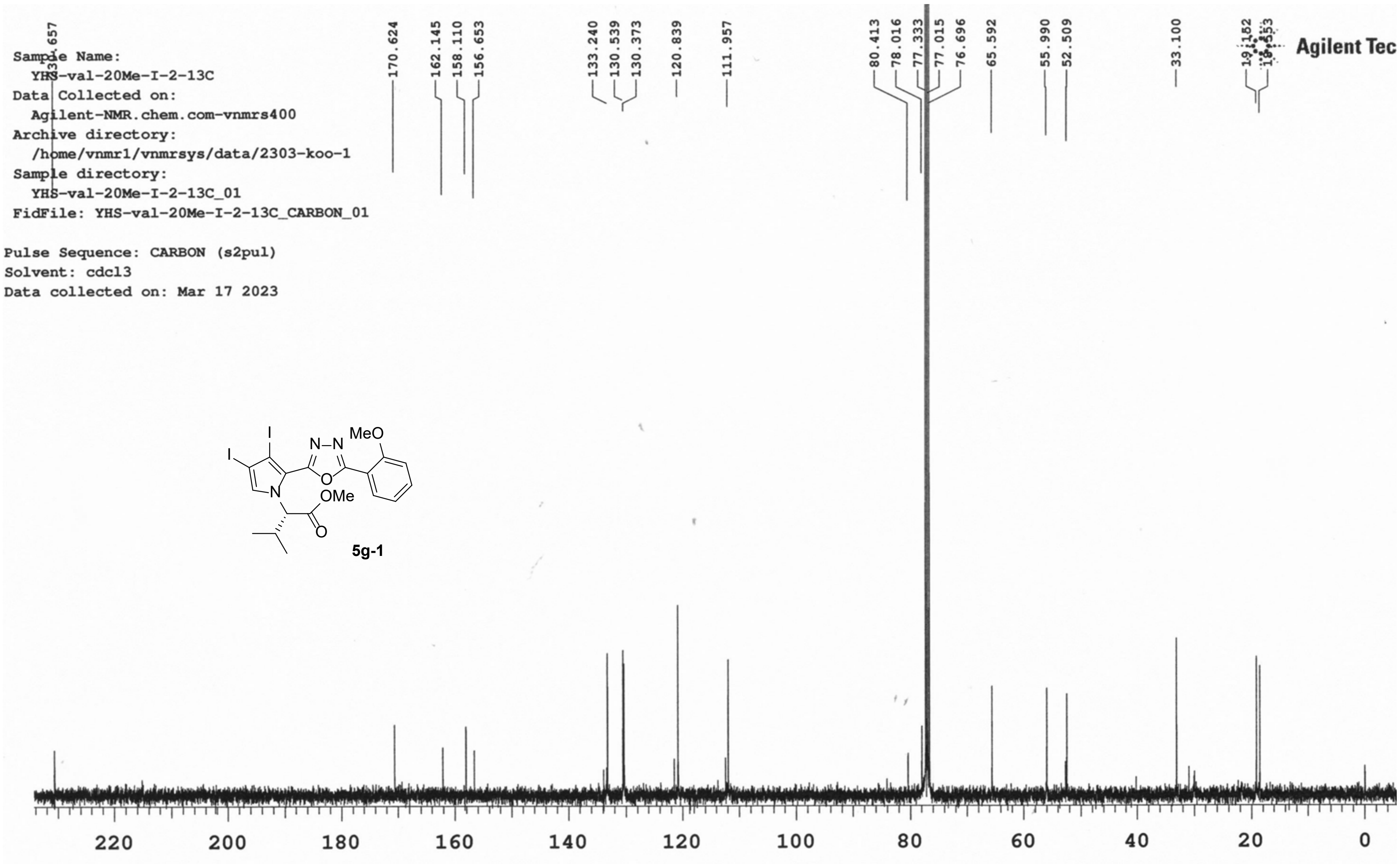
Sample Name:
YHS-val-20Me-I-2-13C
Data Collected on:
Agilent-NMR.chem.com-vnmrs400
Archive directory:
/home/vnmr1/vnmrsys/data/2303-koo-1
Sample directory:
YHS-val-20Me-I-2-13C_01
FidFile: YHS-val-20Me-I-2-13C_CARBON_01
Pulse Sequence: CARBON (s2pul)
Solvent: cdcl3
Data collected on: Mar 17 2023
Agilent Tec
170.624
162.145
158.110
156.653
133.240
130.539
130.373
120.839
111.957
80.413
78.016
77.333
77.015
76.696
65.592
55.990
52.509
33.100
MeO
N–N
O
I
I
N
OMe
O
5g-1
220
200
180
160
140
120
100
80
60
40
20
0

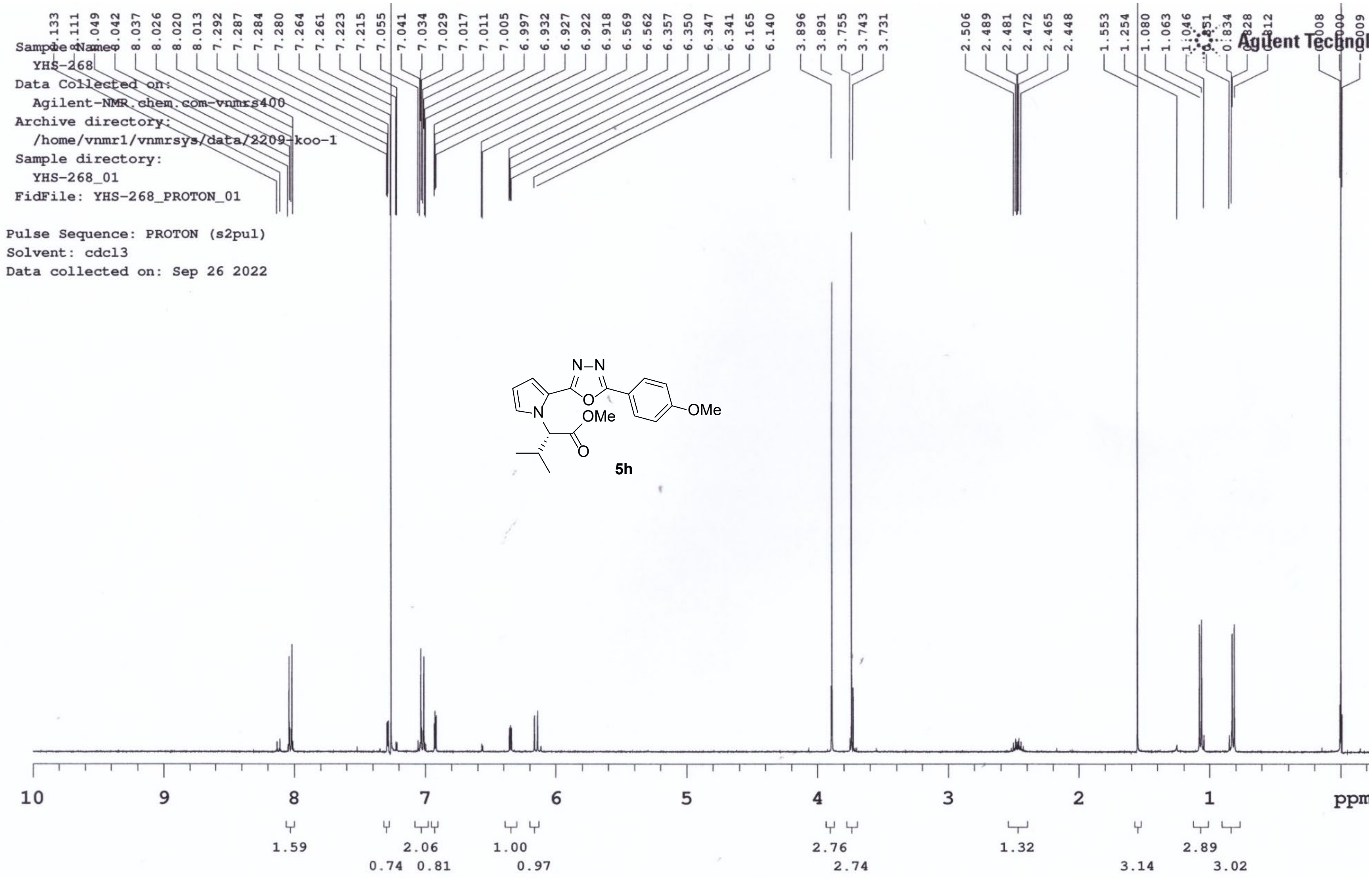
Sample Name:
YHS-268
Data Collected on:
Agilent-NMR.chem.com-vnmrs400
Archive directory:
/home/vnmr1/vnmrsys/data/2209-koo-1
Sample directory:
YHS-268_01
FidFile: YHS-268_PROTON_01
Pulse Sequence: PROTON (s2pul)
Solvent: cdcl3
Data collected on: Sep 26 2022
8.133 8.111 8.049 8.042 8.037 8.026 8.020 8.013 7.292 7.287 7.284 7.280 7.264 7.261 7.223 7.215 7.055 7.041 7.034 7.029 7.017 7.011 7.005 6.997 6.932 6.927 6.922 6.918 6.569 6.562 6.357 6.350 6.347 6.341 6.165 6.140
3.896 3.891 3.755 3.743 3.731
2.506 2.489 2.481 2.472 2.465 2.448
1.553 1.254 1.080 1.063 1.046 0.851 0.834 0.828 0.812
0.008 0.000 -0.009
Agilent Technologies
N–N
O
N
OMe
OMe
5h
10 9 8 7 6 5 4 3 2 1 ppm
1.59 0.74 2.06 0.81 1.00 0.97 2.76 2.74 1.32 3.14 2.89 3.02

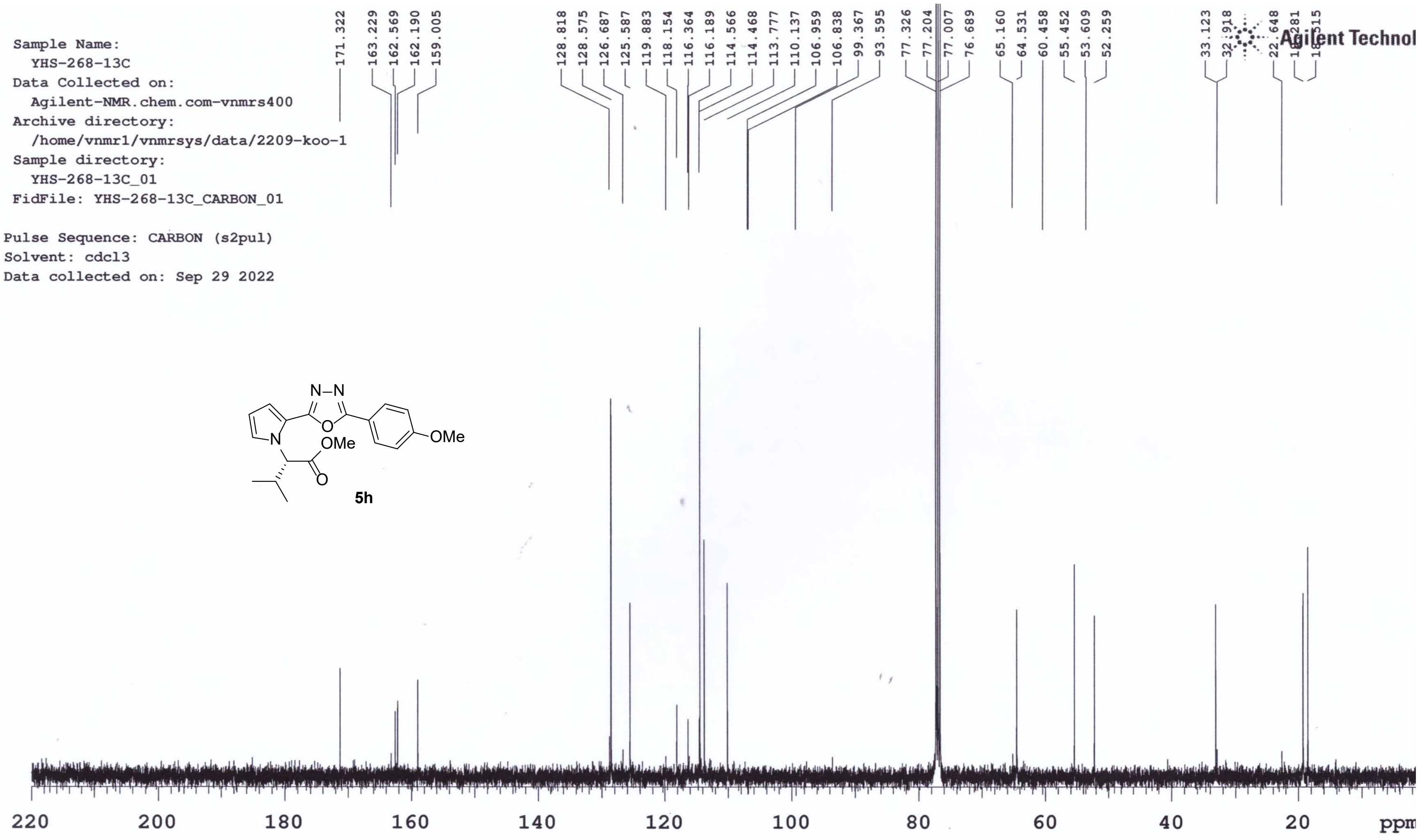

Sample Name:
YHS-268-13C
Data Collected on:
Agilent-NMR.chem.com-vnmrs400
Archive directory:
/home/vnmr1/vnmrsys/data/2209-koo-1
Sample directory:
YHS-268-13C_01
FidFile: YHS-268-13C_CARBON_01
Pulse Sequence: CARBON (s2pul)
Solvent: cdcl3
Data collected on: Sep 29 2022
171.322
163.229
162.569
162.190
159.005
128.818
128.575
126.687
125.587
119.883
118.154
116.364
116.189
114.566
114.468
113.777
110.137
106.959
106.838
99.367
93.595
77.326
77.204
77.007
76.689
65.160
64.531
60.458
55.452
53.609
52.259
33.123
22.648
Agilent Technol
N–N
O
N
OMe
OMe
O
5h
220
200
180
160
140
120
100
80
60
40
20
ppm

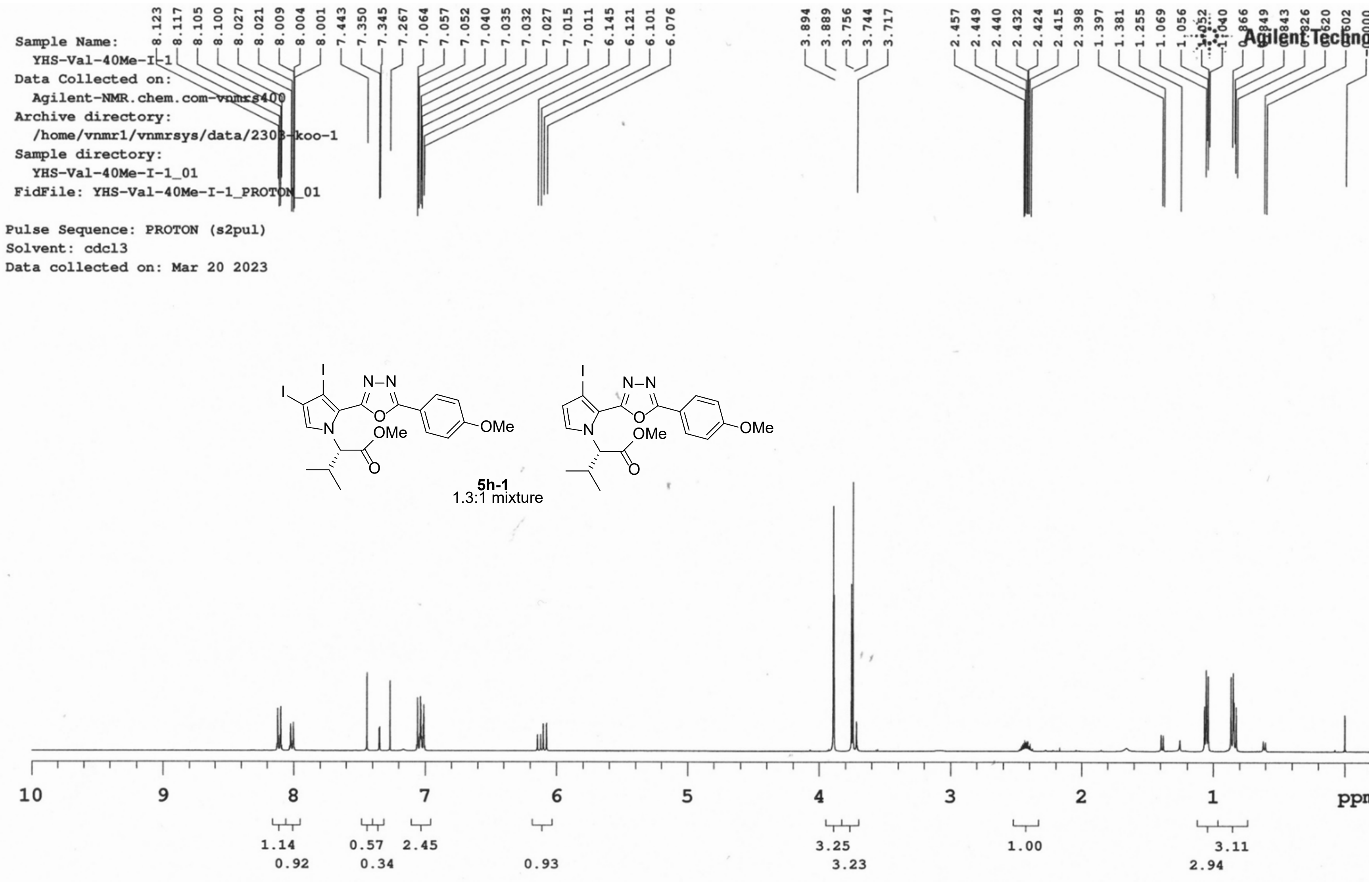

Sample Name:
YHS-Val-4OMe-I-1
Data Collected on:
Agilent-NMR.chem.com-vnmrs400
Archive directory:
/home/vnmr1/vnmrsys/data/2303-koo-1
Sample directory:
YHS-Val-4OMe-I-1_01
FidFile: YHS-Val-4OMe-I-1_PROTON_01
Pulse Sequence: PROTON (s2pul)
Solvent: cdcl3
Data collected on: Mar 20 2023
8.123 8.117 8.105 8.100 8.027 8.021 8.009 8.004 8.001 7.443 7.350 7.345 7.267 7.064 7.057 7.052 7.040 7.035 7.032 7.027 7.015 7.011 6.145 6.121 6.101 6.076
3.894 3.889 3.756 3.744 3.717
2.457 2.449 2.440 2.432 2.424 2.415 2.398 1.397 1.381 1.255 1.069 1.056
I
N–N
O
OMe
N
O
5h-1
1.3:1 mixture
10 9 8 7 6 5 4 3 2 1 ppm
1.14 0.92 0.57 0.34 2.45 0.93 3.25 3.23 1.00 3.11 2.94

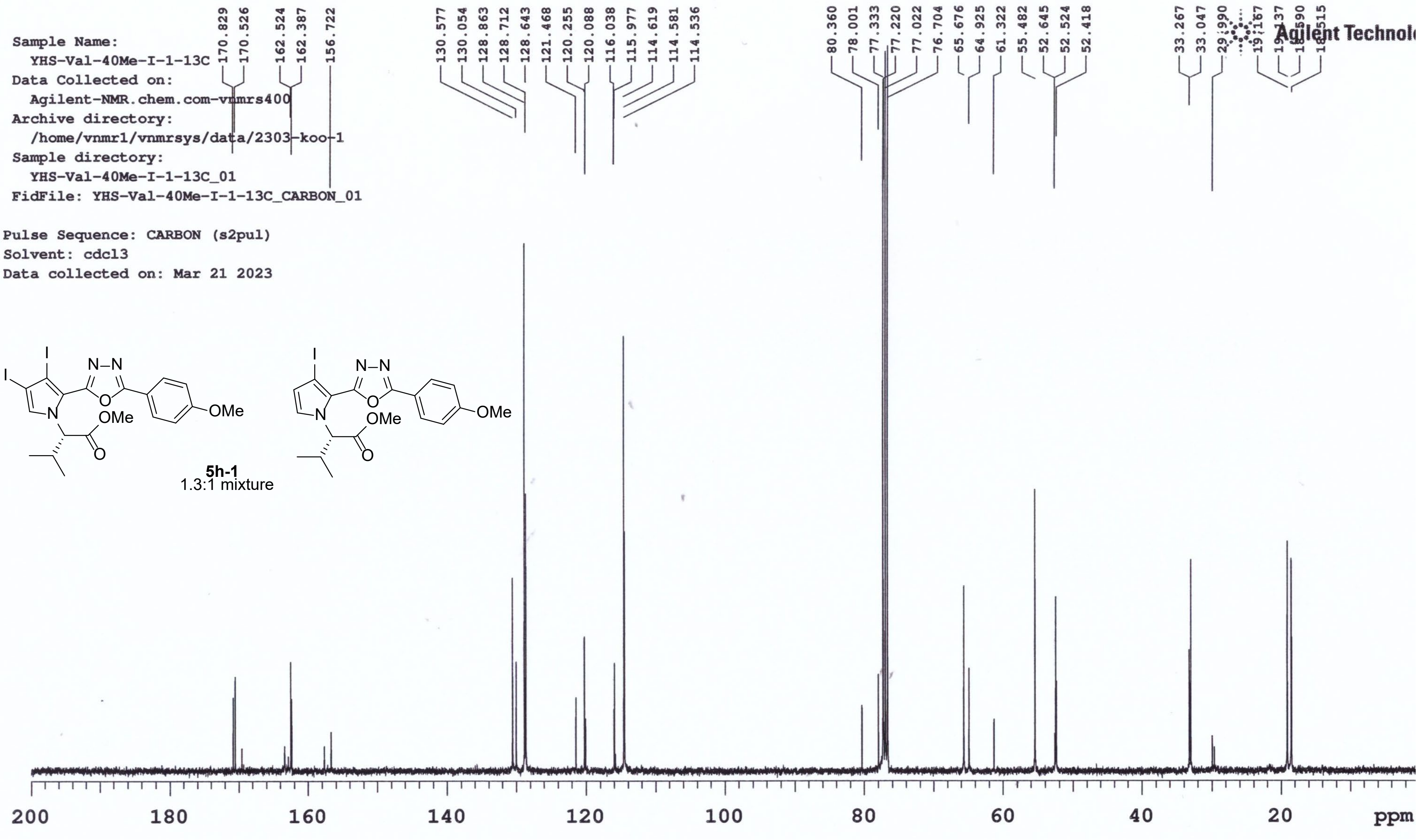
Sample Name:
YHS-Val-4OMe-I-1-13C
Data Collected on:
Agilent-NMR.chem.com-vnmrs400
Archive directory:
/home/vnmr1/vnmrsys/data/2303-koo-1
Sample directory:
YHS-Val-4OMe-I-1-13C_01
FidFile: YHS-Val-4OMe-I-1-13C_CARBON_01
Pulse Sequence: CARBON (s2pul)
Solvent: cdcl3
Data collected on: Mar 21 2023
170.829
170.526
162.524
162.387
156.722
130.577
130.054
128.863
128.712
128.643
121.468
120.255
120.088
116.038
115.977
114.619
114.581
114.536
80.360
78.001
77.333
77.220
77.022
76.704
65.676
64.925
61.322
55.482
52.645
52.524
52.418
33.267
33.047
29.990
19.167
Agilent Technol
I
N–N
O
N
OMe
O
OMe
5h-1
1.3:1 mixture
200
180
160
140
120
100
80
60
40
20
ppm

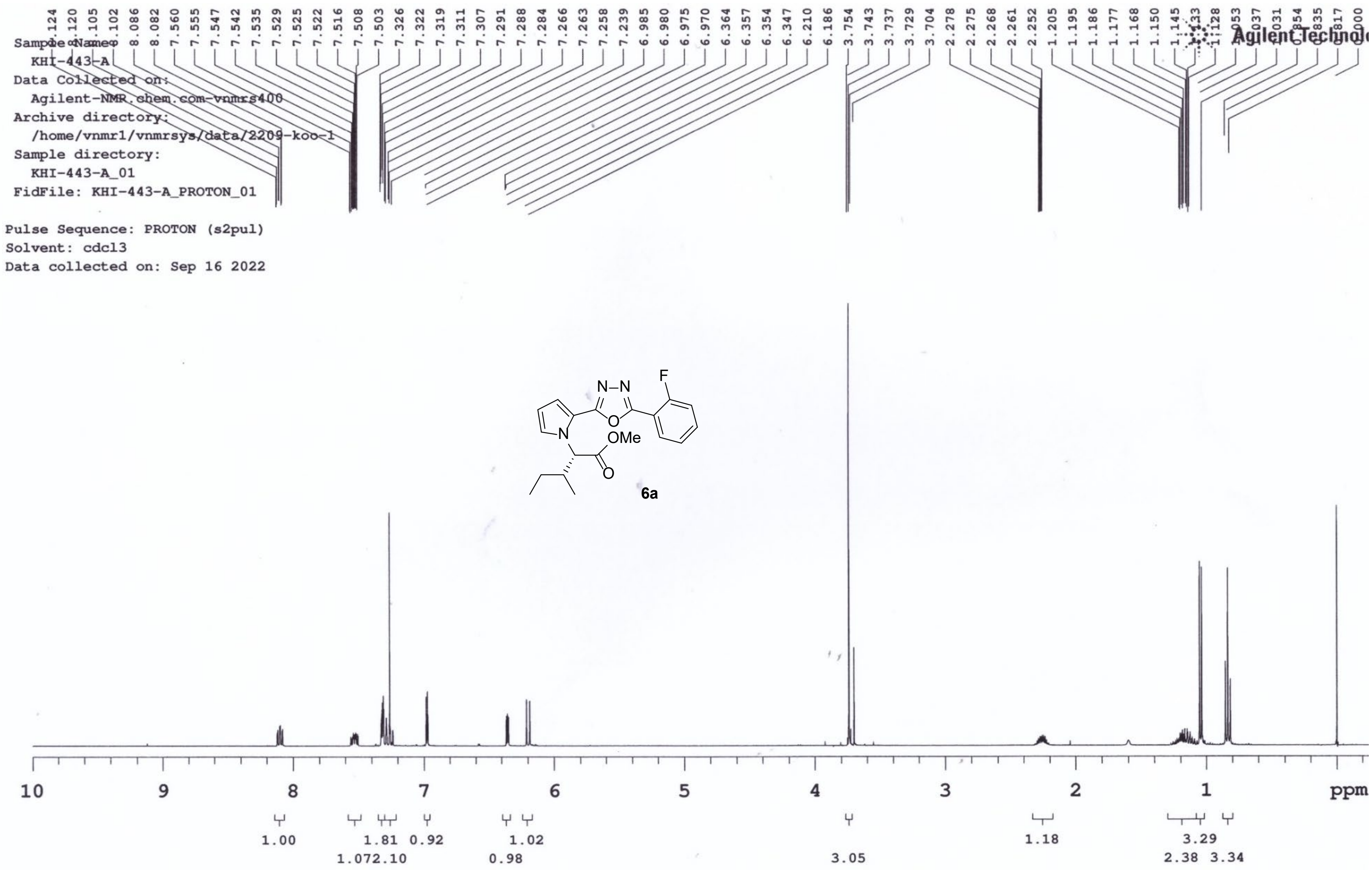
Sample Name:
KHI-443-A
Data Collected on:
Agilent-NMR.chem.com-vnmrs400
Archive directory:
/home/vnmr1/vnmrsys/data/2209-koo-1
Sample directory:
KHI-443-A_01
FidFile: KHI-443-A_PROTON_01
Pulse Sequence: PROTON (s2pul)
Solvent: cdcl3
Data collected on: Sep 16 2022
Agilent Technologies
8.124 8.120 8.105 8.102 8.086 8.082 7.560 7.555 7.547 7.542 7.535 7.529 7.525 7.522 7.516 7.508 7.503 7.326 7.322 7.319 7.311 7.307 7.291 7.288 7.284 7.266 7.263 7.258 7.239 6.985 6.980 6.975 6.970 6.364 6.357 6.354 6.347 6.210 6.186 3.754 3.743 3.737 3.729 3.704 2.278 2.275 2.268 2.261 2.252 1.205 1.195 1.186 1.177 1.168 1.150 1.145 1.133 1.128 1.053 1.037 1.031 0.854 0.835 0.817 0.000
N–N
F
O
N
OMe
O
6a
10 9 8 7 6 5 4 3 2 1 ppm
1.00
1.07
1.81
2.10
0.92
1.02
0.98
3.05
1.18
3.29
2.38
3.34

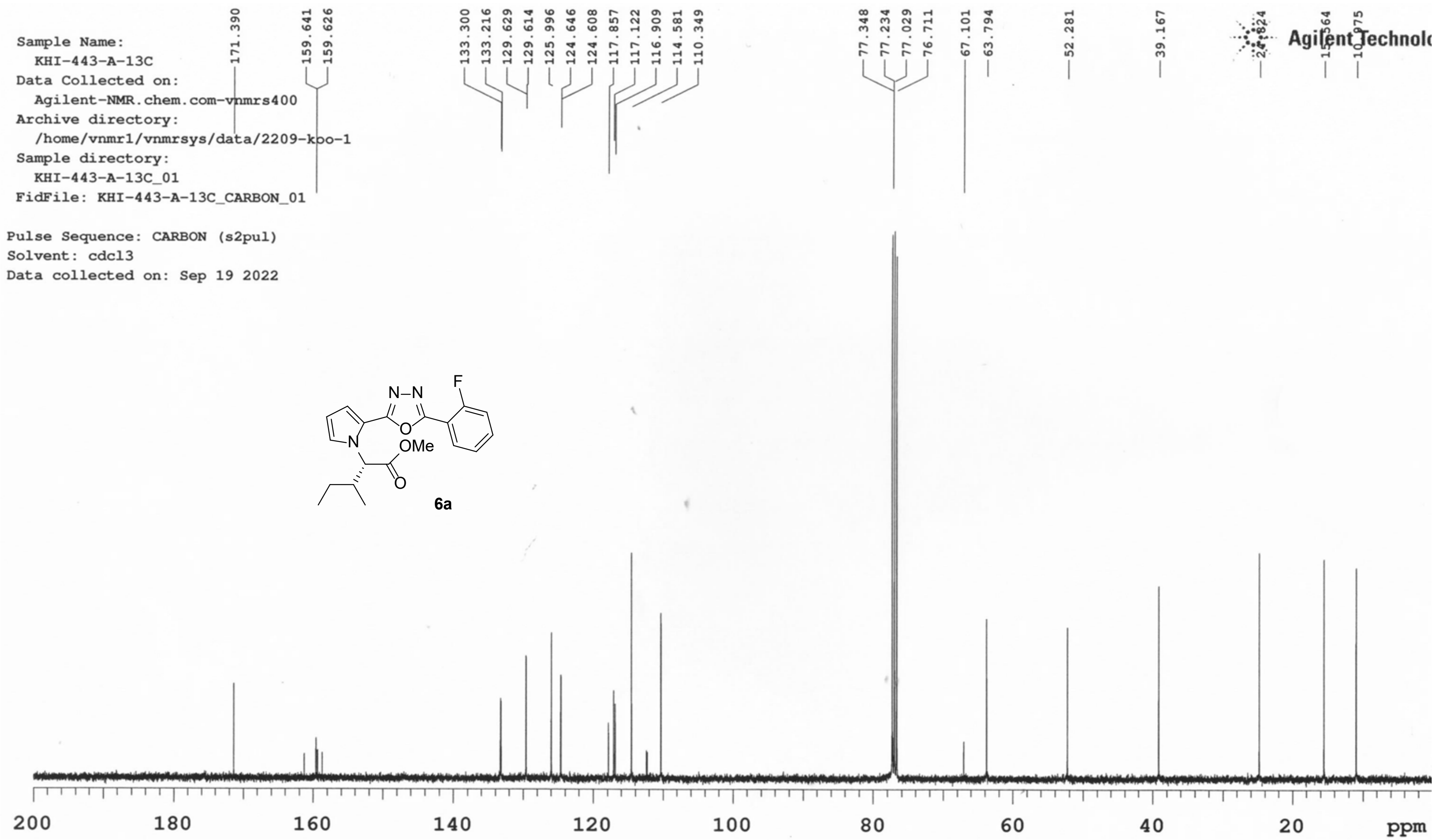
Sample Name:
KHI-443-A-13C
Data Collected on:
Agilent-NMR.chem.com-vnmrs400
Archive directory:
/home/vnmr1/vnmrsys/data/2209-koo-1
Sample directory:
KHI-443-A-13C_01
FidFile: KHI-443-A-13C_CARBON_01
Pulse Sequence: CARBON (s2pul)
Solvent: cdcl3
Data collected on: Sep 19 2022
171.390
159.641
159.626
133.300
133.216
129.629
129.614
125.996
124.646
124.608
117.857
117.122
116.909
114.581
110.349
77.348
77.234
77.029
76.711
67.101
63.794
52.281
39.167
Agilent Technolo
F
N–N
O
N
OMe
O
6a
200
180
160
140
120
100
80
60
40
20
ppm

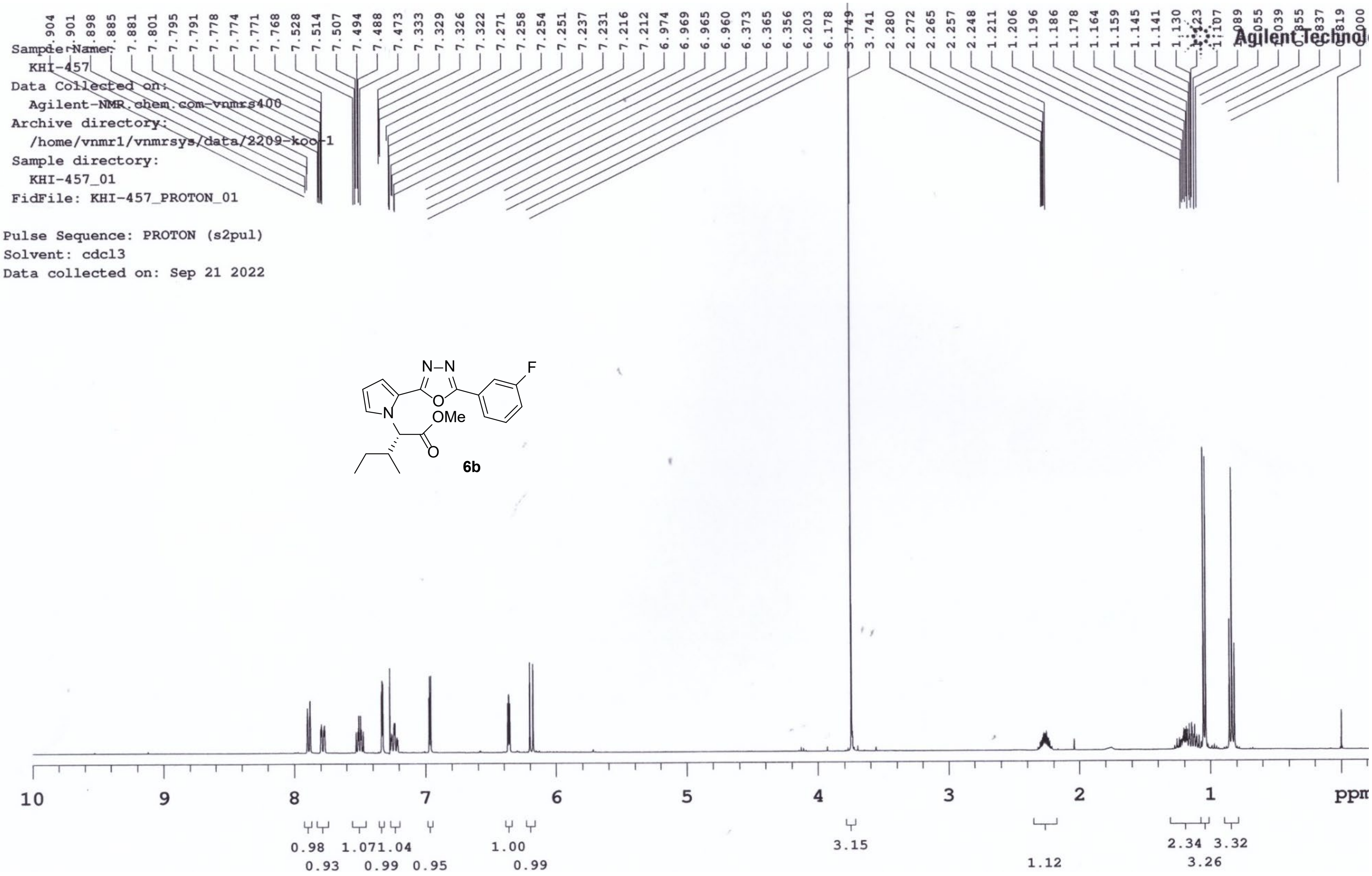

Sample Name:
KHI-457
Data Collected on:
Agilent-NMR.chem.com-vnmrs400
Archive directory:
/home/vnmr1/vnmrsys/data/2209-koo-1
Sample directory:
KHI-457_01
FidFile: KHI-457_PROTON_01
Pulse Sequence: PROTON (s2pul)
Solvent: cdcl3
Data collected on: Sep 21 2022
Agilent Technol
N–N
F
O
N
OMe
O
6b
ppm

Sample Name:
KHI-457-13C
Data Collected on:
Agilent-NMR.chem.com-vnmrs400
Archive directory:
/home/vnmr1/vnmrsys/data/2209-koo-1
Sample directory:
KHI-457-13C_01
FidFile: KHI-457-13C_CARBON_01

Pulse Sequence: CARBON (s2pul)
Solvent: cdcl3
Data collected on: Sep 22 2022

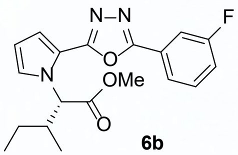

6b

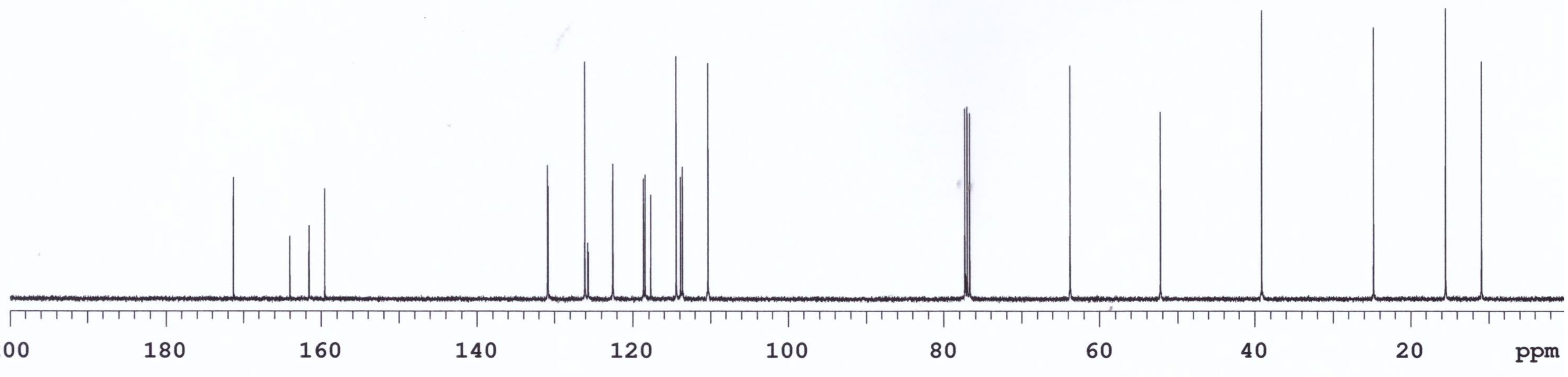

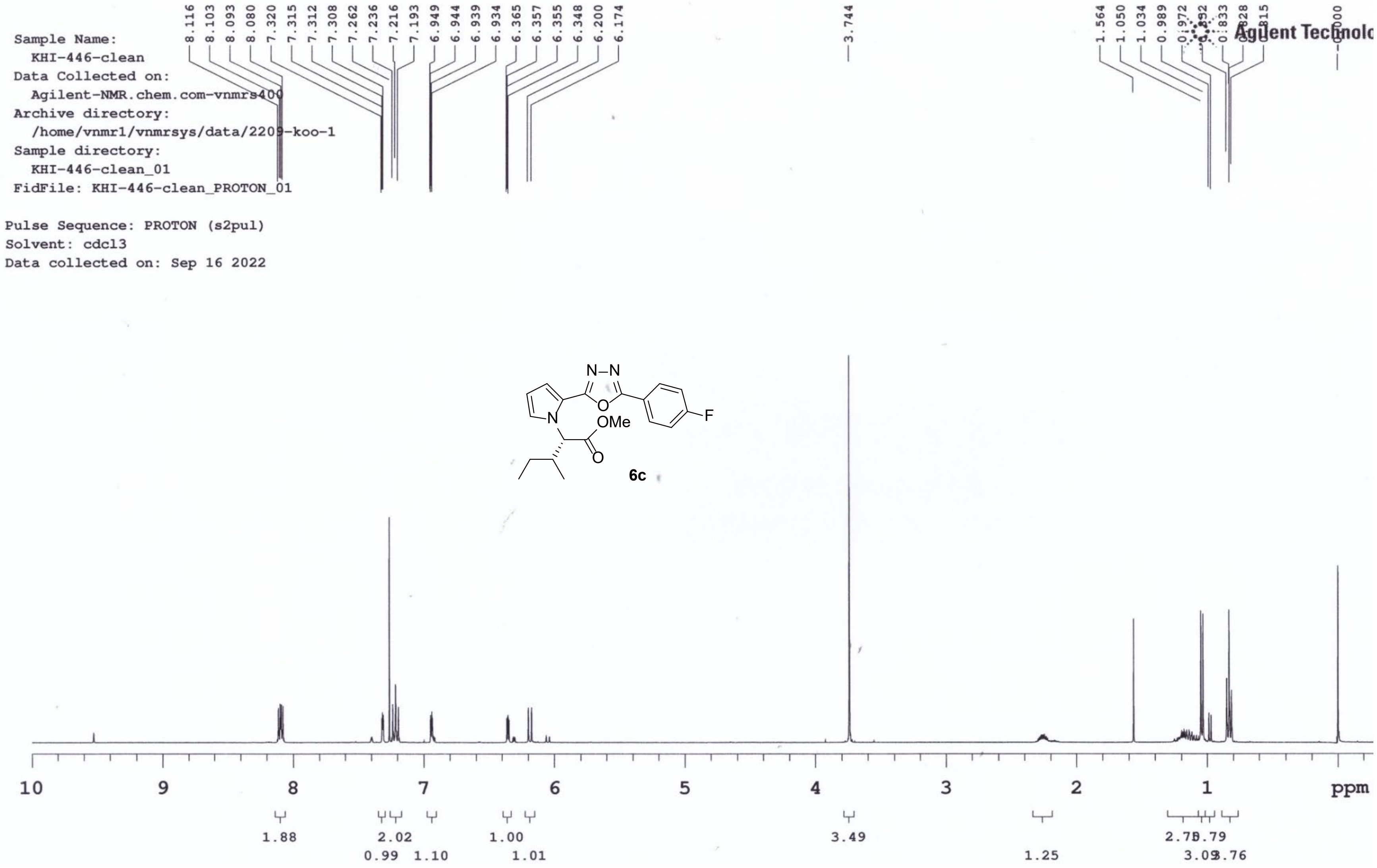
Sample Name:
KHI-446-clean
Data Collected on:
Agilent-NMR.chem.com-vnmrs400
Archive directory:
/home/vnmr1/vnmrsys/data/2209-koo-1
Sample directory:
KHI-446-clean_01
FidFile: KHI-446-clean_PROTON_01
Pulse Sequence: PROTON (s2pul)
Solvent: cdcl3
Data collected on: Sep 16 2022
8.116
8.103
8.093
8.080
7.320
7.315
7.312
7.308
7.262
7.236
7.216
7.193
6.949
6.944
6.939
6.934
6.365
6.357
6.355
6.348
6.200
6.174
3.744
1.564
1.050
1.034
0.989
0.833
0.000
N–N
O
N
OMe
O
F
6c
10
9
8
7
6
5
4
3
2
1
ppm
1.88
2.02
0.99
1.10
1.00
1.01
3.49
1.25

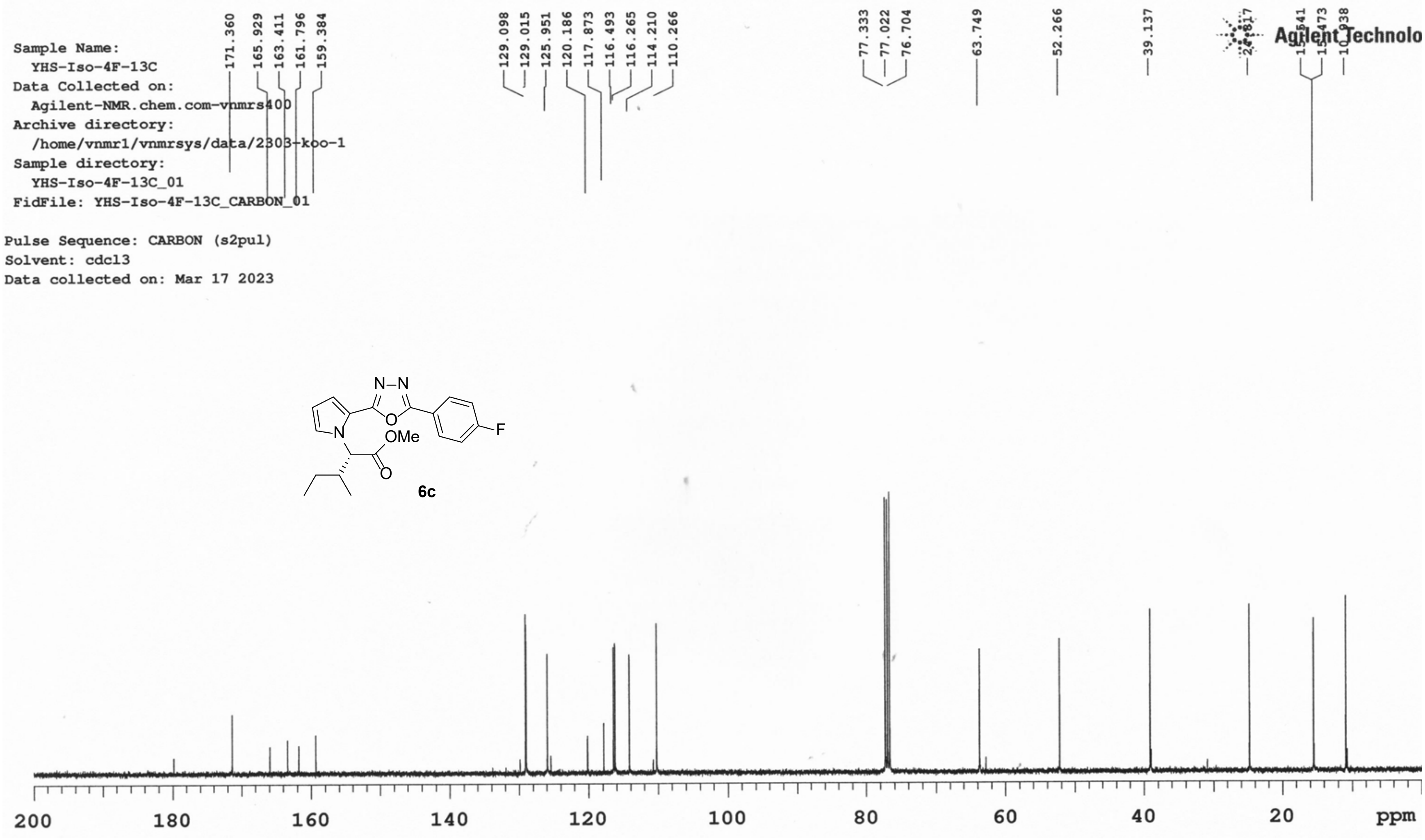
Sample Name:
YHS-Iso-4F-13C
Data Collected on:
Agilent-NMR.chem.com-vnmrs400
Archive directory:
/home/vnmr1/vnmrsys/data/2303-koo-1
Sample directory:
YHS-Iso-4F-13C_01
FidFile: YHS-Iso-4F-13C_CARBON_01
Pulse Sequence: CARBON (s2pul)
Solvent: cdcl3
Data collected on: Mar 17 2023
171.360
165.929
163.411
161.796
159.384
129.098
129.015
125.951
120.186
117.873
116.493
116.265
114.210
110.266
77.333
77.022
76.704
63.749
52.266
39.137
Agilent Technolo
N–N
O
N
OMe
O
F
6c
200
180
160
140
120
100
80
60
40
20
ppm

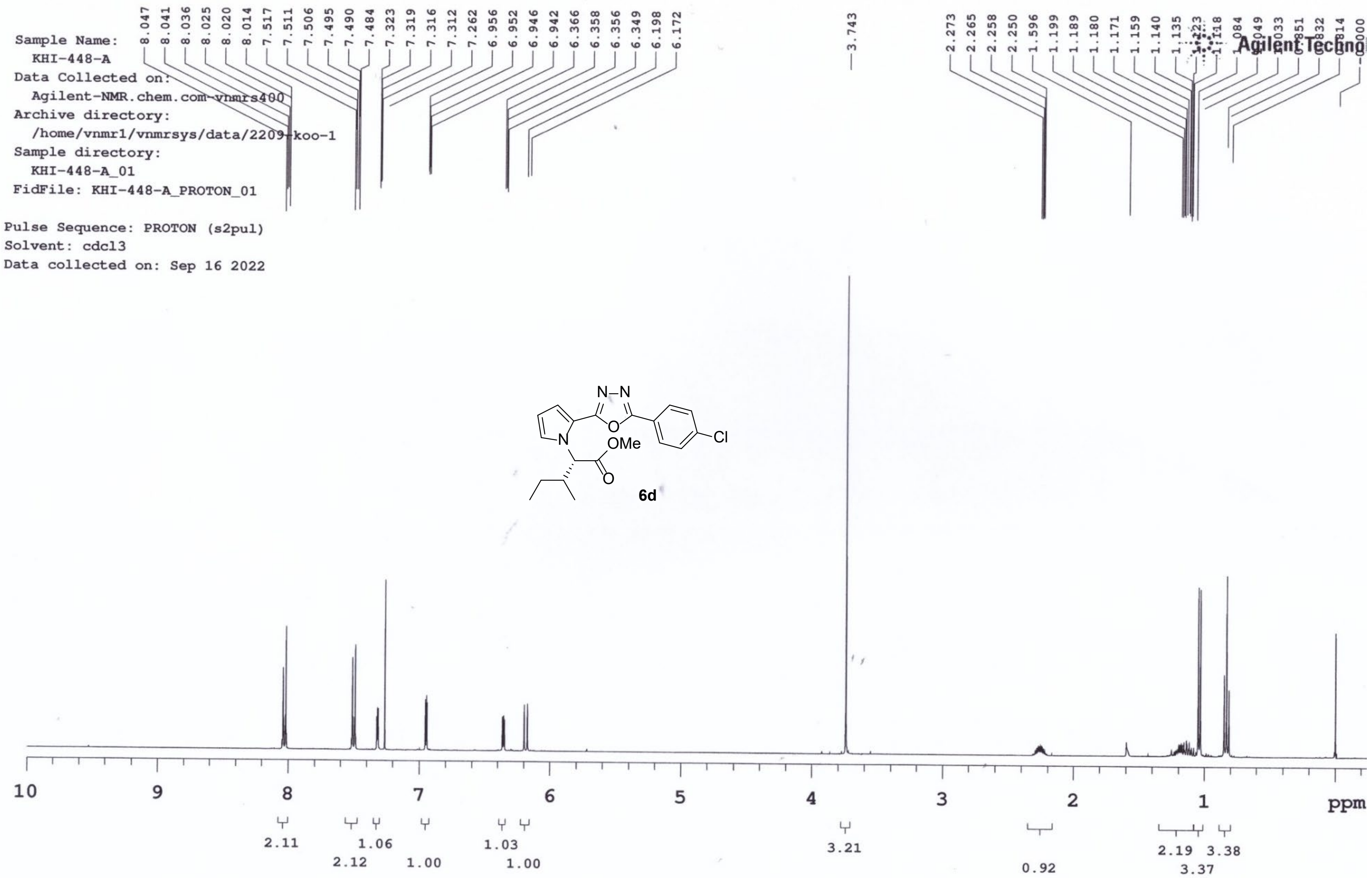
Sample Name:
KHI-448-A
Data Collected on:
Agilent-NMR.chem.com-vnmrs400
Archive directory:
/home/vnmr1/vnmrsys/data/2209-koo-1
Sample directory:
KHI-448-A_01
FidFile: KHI-448-A_PROTON_01
Pulse Sequence: PROTON (s2pul)
Solvent: cdcl3
Data collected on: Sep 16 2022
8.047 8.041 8.036 8.025 8.020 8.014 7.517 7.511 7.506 7.495 7.490 7.484 7.323 7.319 7.316 7.312 7.262 6.956 6.952 6.946 6.942 6.366 6.358 6.356 6.349 6.198 6.172
3.743
2.273 2.265 2.258 2.250 1.596 1.199 1.189 1.180 1.171 1.159 1.140 1.135 1.118 1.084 1.049 1.033 0.851 0.832 0.814 0.000
Agilent Techno
N–N
O
N
OMe
O
Cl
6d
10 9 8 7 6 5 4 3 2 1 ppm
2.11 2.12 1.06 1.00 1.03 1.00 3.21 0.92 2.19 3.37 3.38

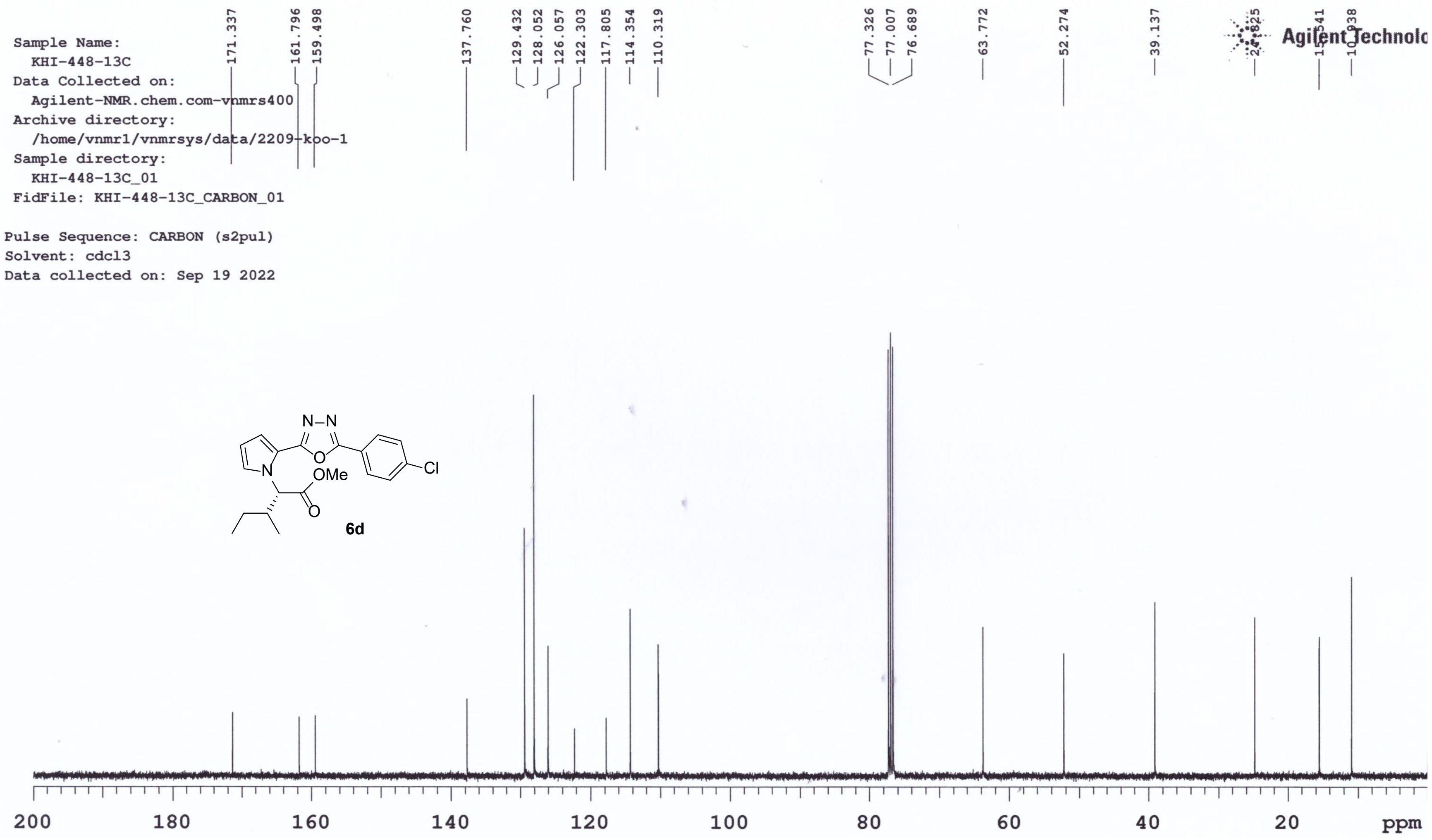

Sample Name:
KHI-448-13C
Data Collected on:
Agilent-NMR.chem.com-vnmrs400
Archive directory:
/home/vnmr1/vnmrsys/data/2209-koo-1
Sample directory:
KHI-448-13C_01
FidFile: KHI-448-13C_CARBON_01
Pulse Sequence: CARBON (s2pul)
Solvent: cdcl3
Data collected on: Sep 19 2022
171.337
161.796
159.498
137.760
129.432
128.052
126.057
122.303
117.805
114.354
110.319
77.326
77.007
76.689
63.772
52.274
39.137
N–N
O
N
OMe
O
Cl
6d
200
180
160
140
120
100
80
60
40
20
ppm

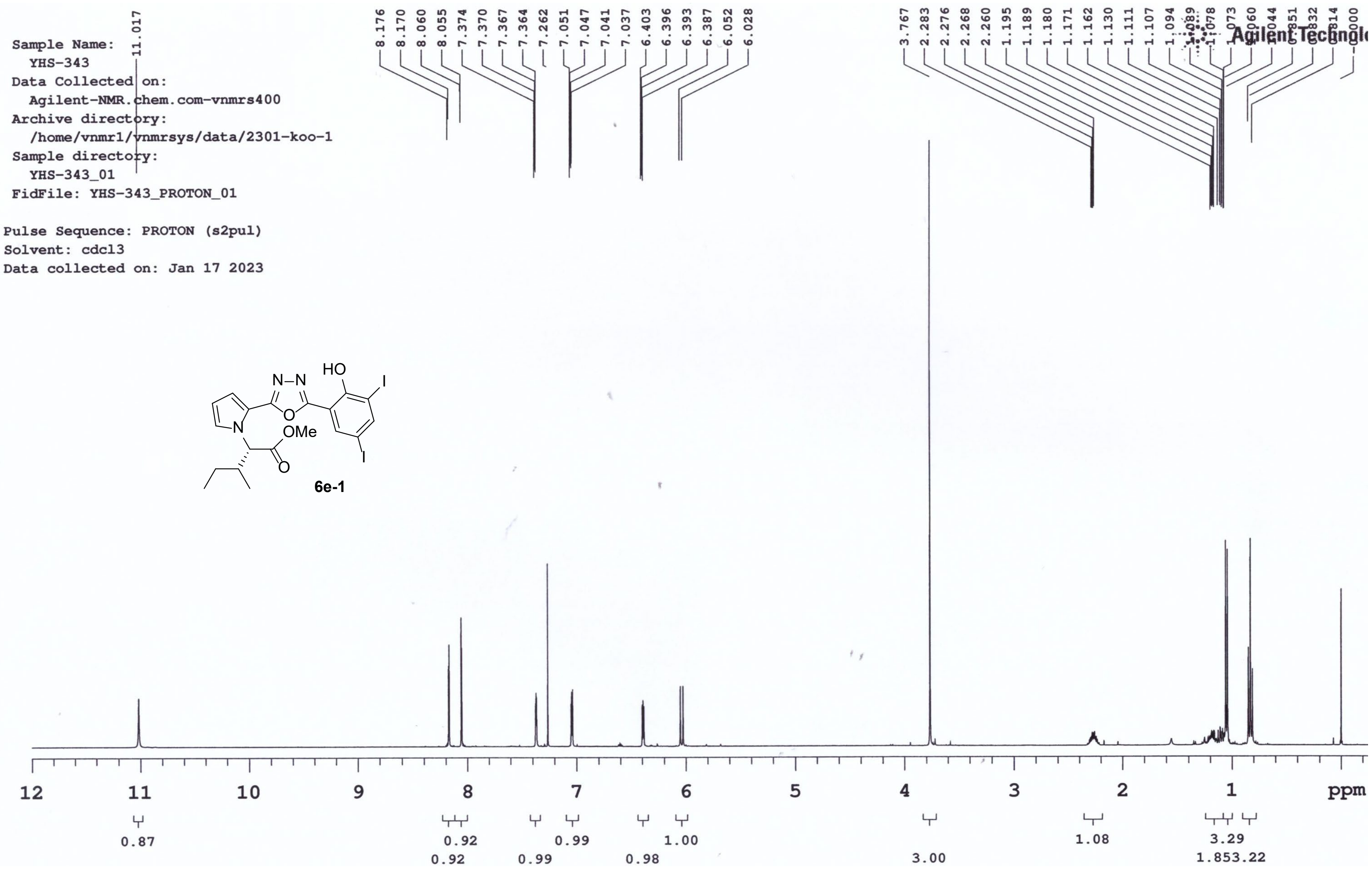

Sample Name:
YHS-343
Data Collected on:
Agilent-NMR.chem.com-vnmrs400
Archive directory:
/home/vnmr1/vnmrsys/data/2301-koo-1
Sample directory:
YHS-343_01
FidFile: YHS-343_PROTON_01
Pulse Sequence: PROTON (s2pul)
Solvent: cdcl3
Data collected on: Jan 17 2023
11.017
8.176
8.170
8.060
8.055
7.374
7.370
7.367
7.364
7.262
7.051
7.047
7.041
7.037
6.403
6.396
6.393
6.387
6.052
6.028
3.767
2.283
2.276
2.268
2.260
1.195
1.189
1.180
1.171
1.162
1.130
1.111
1.107
1.094
1.089
1.078
073
060
044
851
832
814
0.000
Agilent Technolo
HO
N–N
O
N
OMe
O
I
I
6e-1
12 11 10 9 8 7 6 5 4 3 2 1 ppm
0.87
0.92
0.92
0.99
0.99
0.98
1.00
3.00
1.08
3.29
1.853.22

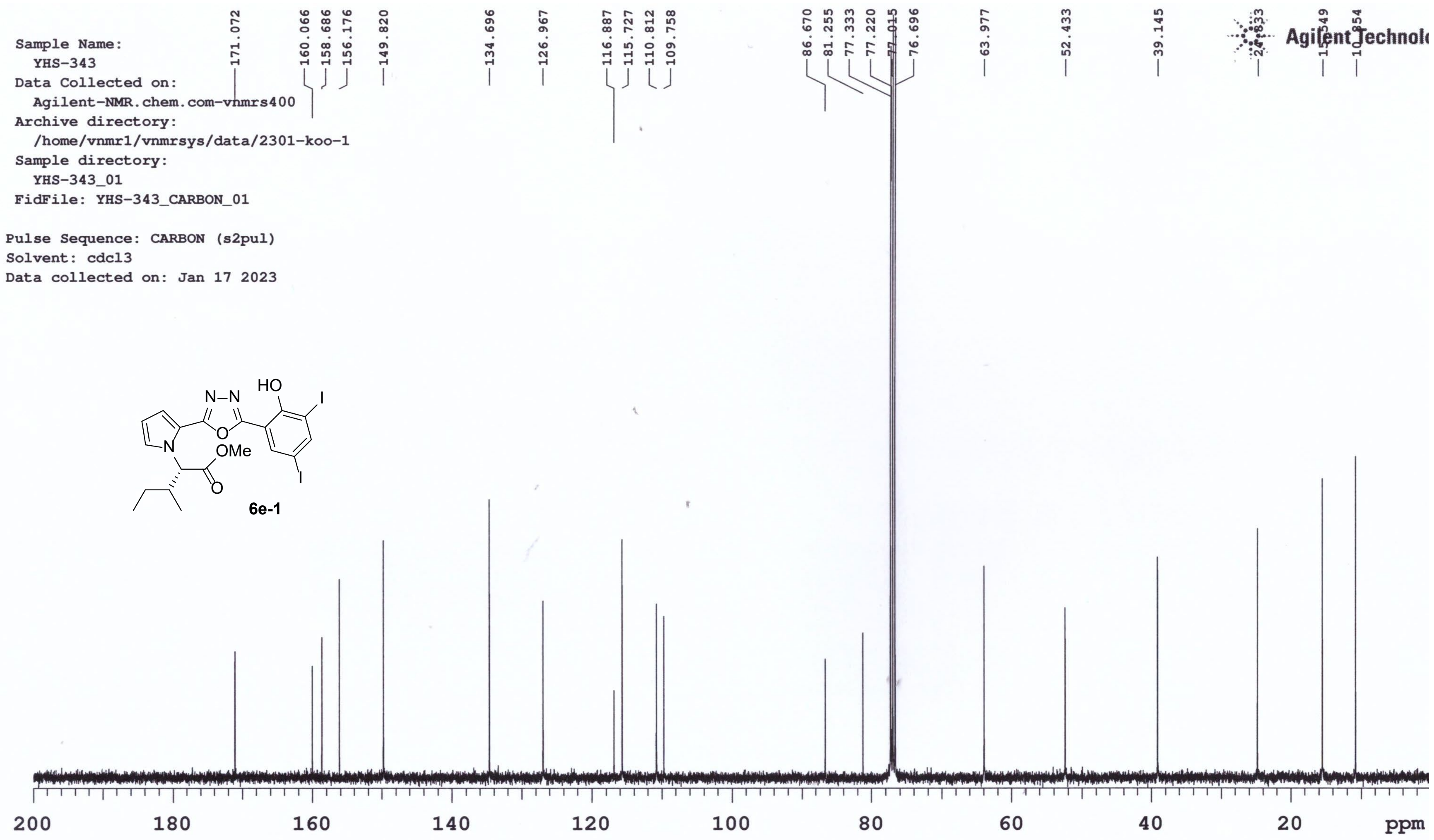
Sample Name:
YHS-343
Data Collected on:
Agilent-NMR.chem.com-vnmrs400
Archive directory:
/home/vnmr1/vnmrsys/data/2301-koo-1
Sample directory:
YHS-343_01
FidFile: YHS-343_CARBON_01
Pulse Sequence: CARBON (s2pul)
Solvent: cdcl3
Data collected on: Jan 17 2023
171.072
160.066
158.686
156.176
149.820
134.696
126.967
116.887
115.727
110.812
109.758
86.670
81.255
77.333
77.220
77.015
76.696
63.977
52.433
39.145
HO
I
N–N
O
N
OMe
O
I
6e-1
200
180
160
140
120
100
80
60
40
20
ppm

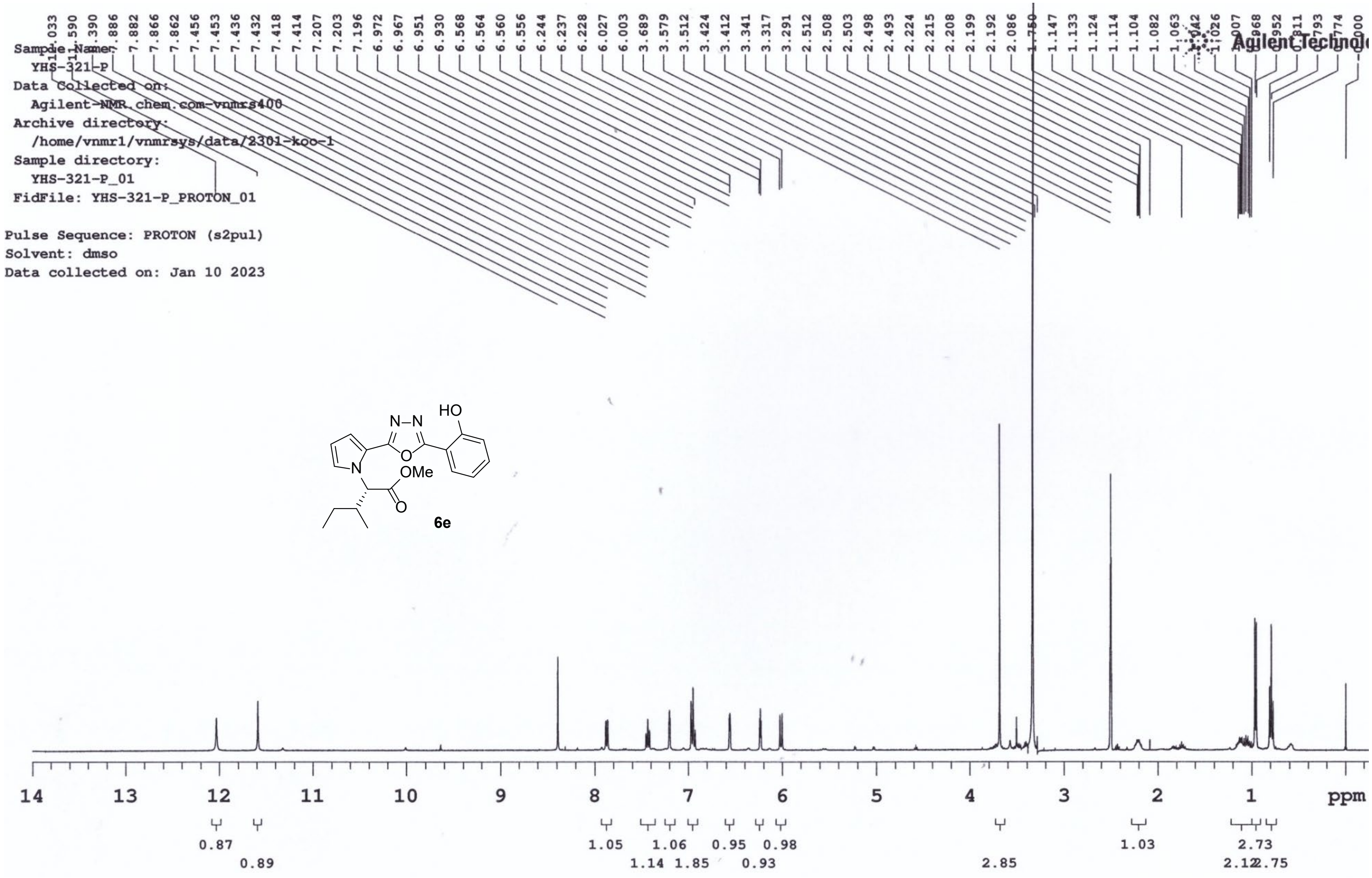
Sample Name:
YHS-321-P
Data Collected on:
Agilent-NMR.chem.com-vnmrs400
Archive directory:
/home/vnmr1/vnmrsys/data/2301-koo-1
Sample directory:
YHS-321-P_01
FidFile: YHS-321-P_PROTON_01
Pulse Sequence: PROTON (s2pul)
Solvent: dmso
Data collected on: Jan 10 2023
HO
N–N
O
N
OMe
O
6e
14 13 12 11 10 9 8 7 6 5 4 3 2 1 ppm
0.87 0.89 1.05 1.14 1.06 1.85 0.95 0.93 0.98 2.85 1.03 2.12 2.73 2.75

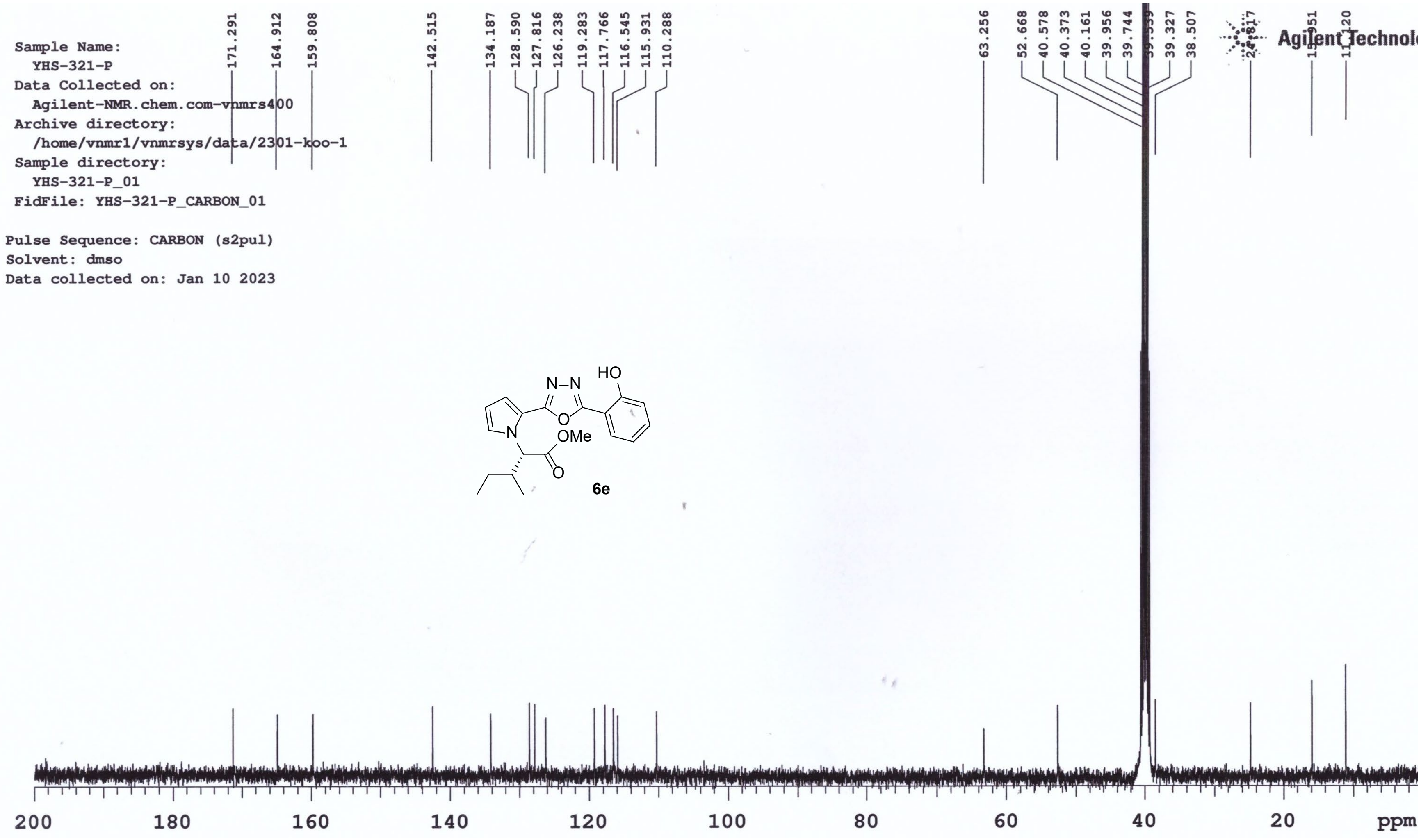

Sample Name:
YHS-321-P
Data Collected on:
Agilent-NMR.chem.com-vnmrs400
Archive directory:
/home/vnmr1/vnmrsys/data/2301-koo-1
Sample directory:
YHS-321-P_01
FidFile: YHS-321-P_CARBON_01
Pulse Sequence: CARBON (s2pul)
Solvent: dmso
Data collected on: Jan 10 2023
171.291
164.912
159.808
142.515
134.187
128.590
127.816
126.238
119.283
117.766
116.545
115.931
110.288
63.256
52.668
40.578
40.373
40.161
39.956
39.744
39.327
38.507
Agilent Technol
HO
N–N
O
N
OMe
O
6e
200
180
160
140
120
100
80
60
40
20
ppm

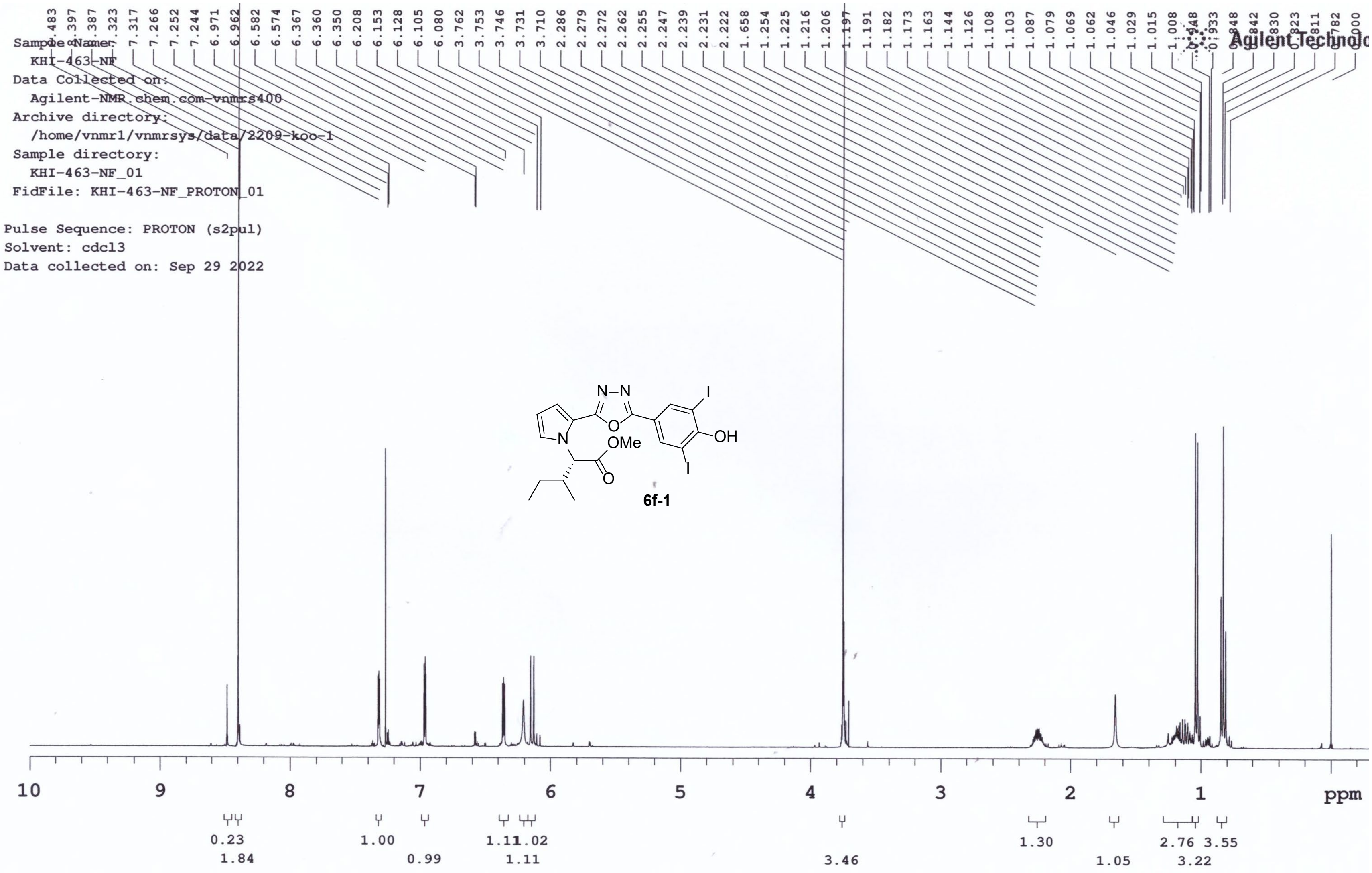
Sample Name:
KHI-463-NF
Data Collected on:
Agilent-NMR.chem.com-vnmrs400
Archive directory:
/home/vnmr1/vnmrsys/data/2209-koo-1
Sample directory:
KHI-463-NF_01
FidFile: KHI-463-NF_PROTON_01
Pulse Sequence: PROTON (s2pul)
Solvent: cdcl3
Data collected on: Sep 29 2022
Agilent Technolo
N–N
O
N
OMe
O
I
OH
I
6f-1
10
9
8
7
6
5
4
3
2
1
ppm
0.23
1.84
1.00
0.99
1.11
1.02
1.11
3.46
1.30
1.05
2.76
3.22
3.55

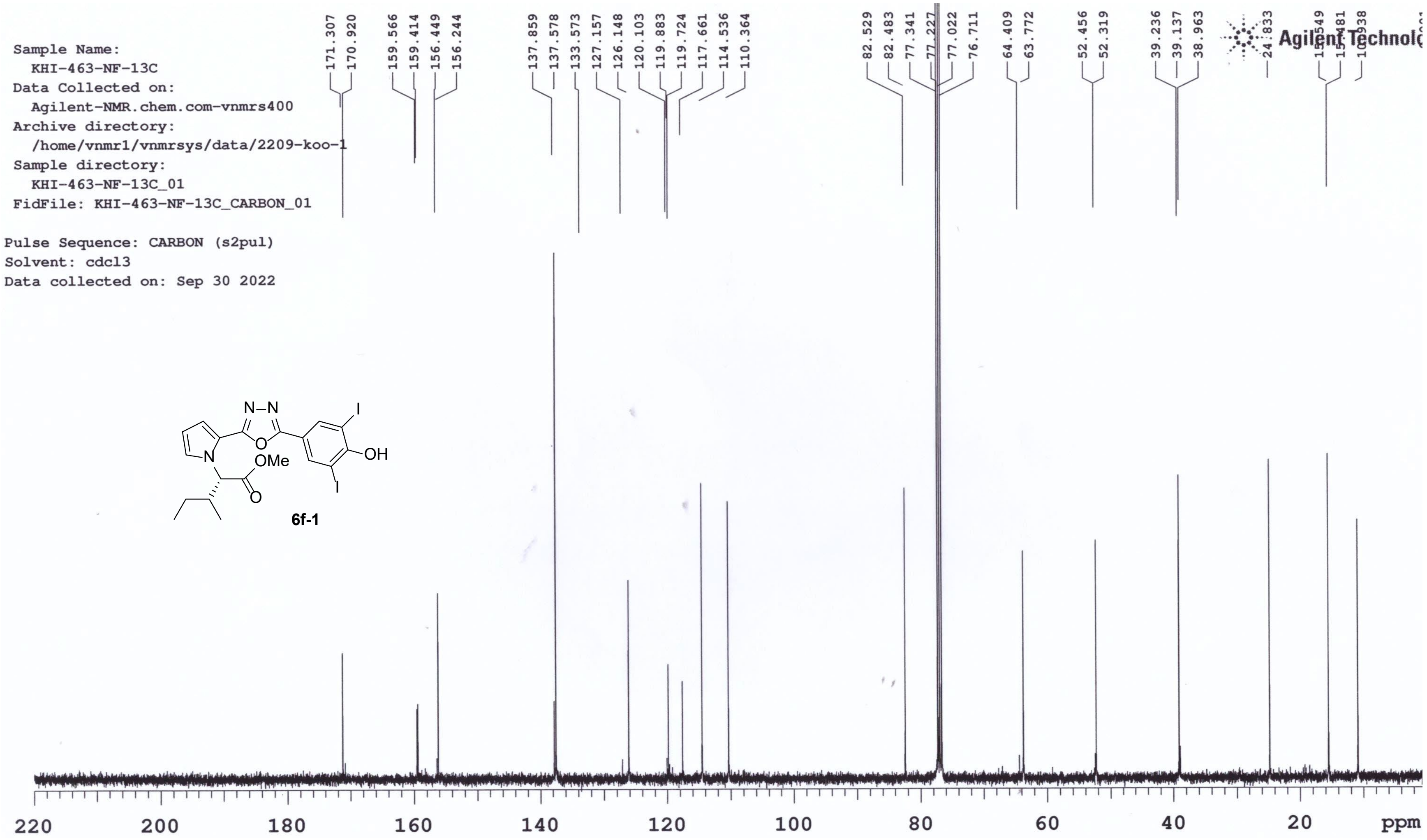
Sample Name:
KHI-463-NF-13C
Data Collected on:
Agilent-NMR.chem.com-vnmrs400
Archive directory:
/home/vnmr1/vnmrsys/data/2209-koo-1
Sample directory:
KHI-463-NF-13C_01
FidFile: KHI-463-NF-13C_CARBON_01
Pulse Sequence: CARBON (s2pul)
Solvent: cdcl3
Data collected on: Sep 30 2022
171.307
170.920
159.566
159.414
156.449
156.244
137.859
137.578
133.573
127.157
126.148
120.103
119.883
119.724
117.661
114.536
110.364
82.529
82.483
77.341
77.227
77.022
76.711
64.409
63.772
52.456
52.319
39.236
39.137
38.963
24.833
10.938
Agilent Technologies
N–N
I
OH
OMe
O
N
6f-1
220
200
180
160
140
120
100
80
60
40
20
ppm

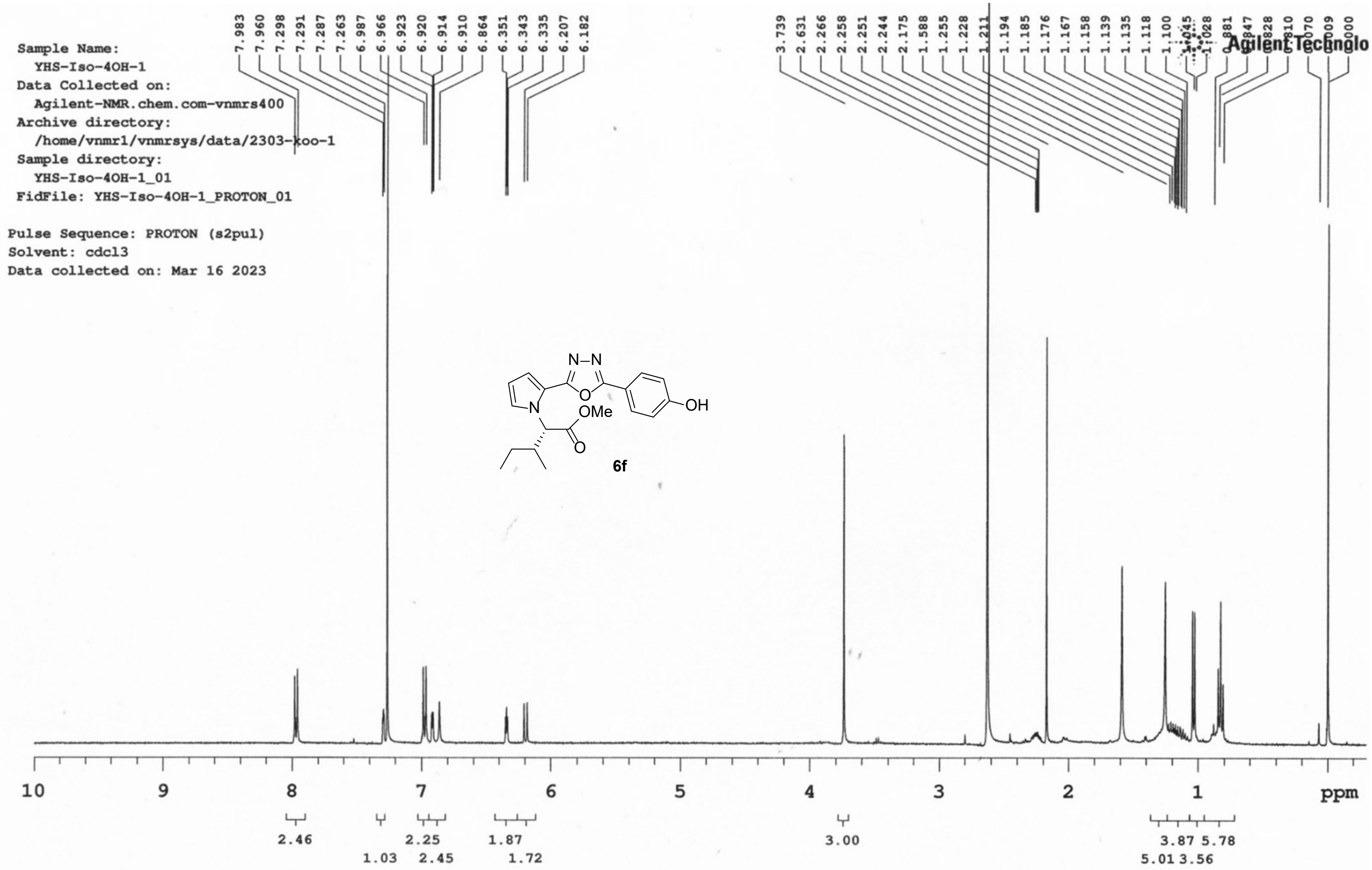
Sample Name:
YHS-Iso-4OH-1
Data Collected on:
Agilent-NMR.chem.com-vnmrs400
Archive directory:
/home/vnmr1/vnmrsys/data/2303-koo-1
Sample directory:
YHS-Iso-4OH-1_01
FidFile: YHS-Iso-4OH-1_PROTON_01
Pulse Sequence: PROTON (s2pul)
Solvent: cdcl3
Data collected on: Mar 16 2023
7.983 7.960 7.298 7.291 7.287 7.263 6.987 6.966 6.923 6.920 6.914 6.910 6.864 6.351 6.343 6.335 6.207 6.182
3.739 2.631 2.266 2.258 2.251 2.244 2.175 1.588 1.255 1.228 1.211 1.194 1.185 1.176 1.167 1.158 1.139 1.135 1.118 1.100
N–N
O
OMe
OH
N
O
6f
10 9 8 7 6 5 4 3 2 1 ppm
2.46 1.03 2.25 2.45 1.87 1.72 3.00 5.01 3.87 3.56 5.78

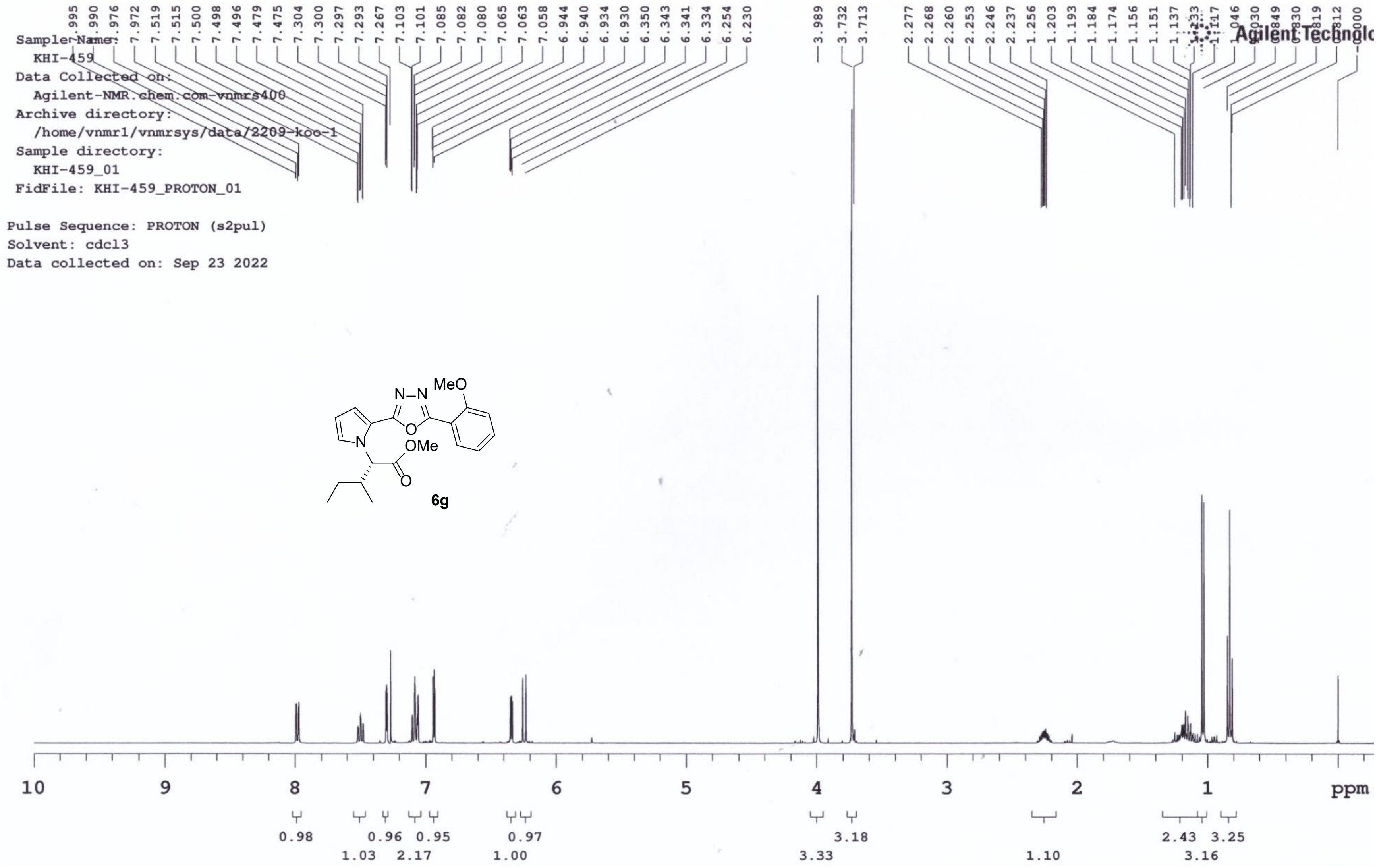
Sample Name:
KHI-459
Data Collected on:
Agilent-NMR.chem.com-vnmrs400
Archive directory:
/home/vnmr1/vnmrsys/data/2209-koo-1
Sample directory:
KHI-459_01
FidFile: KHI-459_PROTON_01
Pulse Sequence: PROTON (s2pul)
Solvent: cdcl3
Data collected on: Sep 23 2022
Agilent Technolo
7.990 7.976 7.972 7.519 7.515 7.500 7.498 7.496 7.479 7.475 7.304 7.300 7.297 7.293 7.267 7.103 7.101 7.085 7.082 7.080 7.065 7.063 7.058 6.944 6.940 6.934 6.930 6.350 6.343 6.341 6.334 6.254 6.230
3.989 3.732 3.713
2.277 2.268 2.260 2.253 2.246 2.237 1.256 1.203 1.193 1.184 1.174 1.156 1.151 1.137 1.117 1.046 1.030 0.849 0.830 0.819 0.812 0.000
MeO
N–N
O
N
OMe
O
6g
10 9 8 7 6 5 4 3 2 1 ppm
0.98 1.03 0.96 2.17 0.95 1.00 0.97 3.33 3.18 1.10 2.43 3.16 3.25

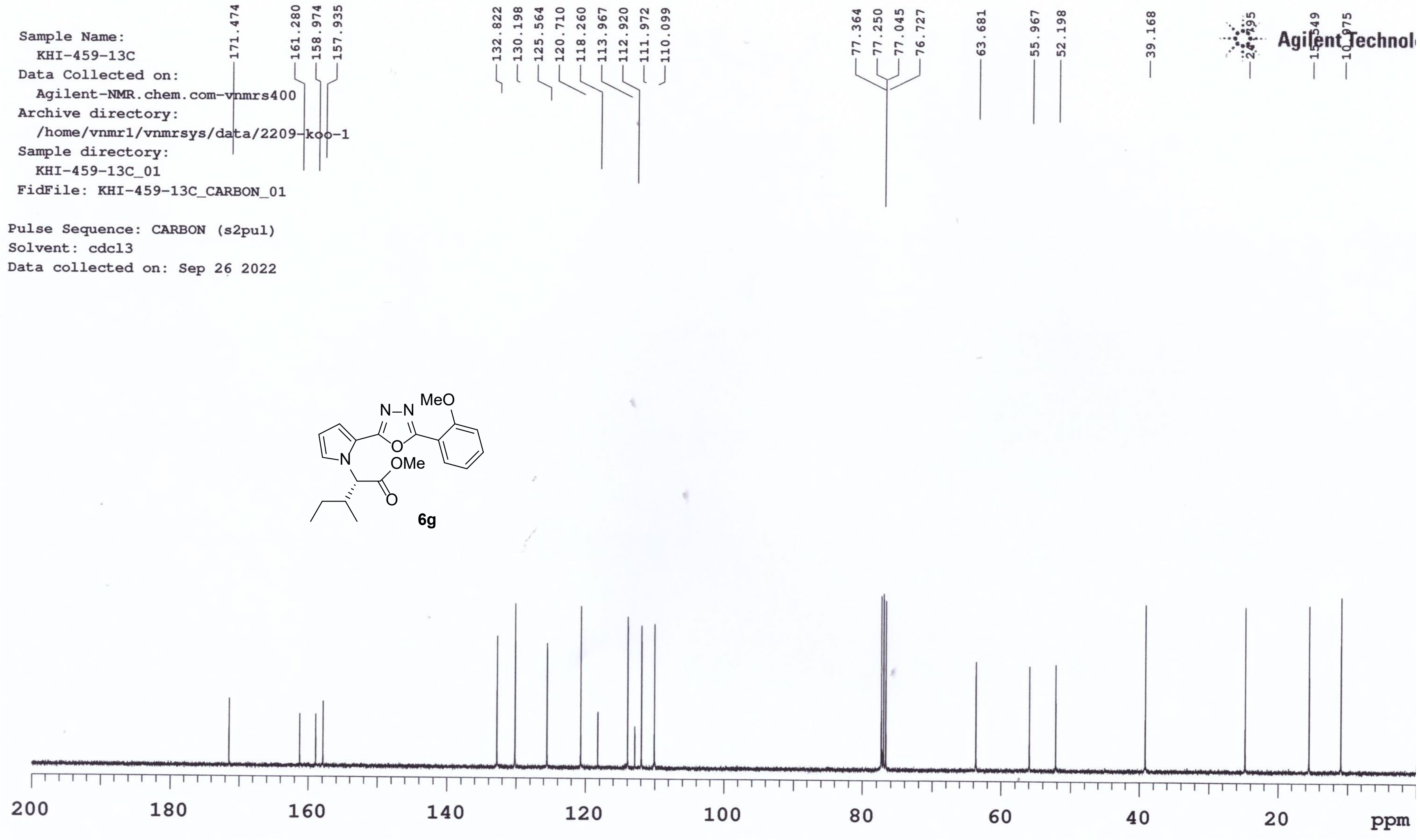
Sample Name:
KHI-459-13C
Data Collected on:
Agilent-NMR.chem.com-vnmrs400
Archive directory:
/home/vnmr1/vnmrsys/data/2209-koo-1
Sample directory:
KHI-459-13C_01
FidFile: KHI-459-13C_CARBON_01
Pulse Sequence: CARBON (s2pul)
Solvent: cdcl3
Data collected on: Sep 26 2022
171.474
161.280
158.974
157.935
132.822
130.198
125.564
120.710
118.260
113.967
112.920
111.972
110.099
77.364
77.250
77.045
76.727
63.681
55.967
52.198
39.168
N–N
MeO
OMe
N
O
6g
200
180
160
140
120
100
80
60
40
20
ppm

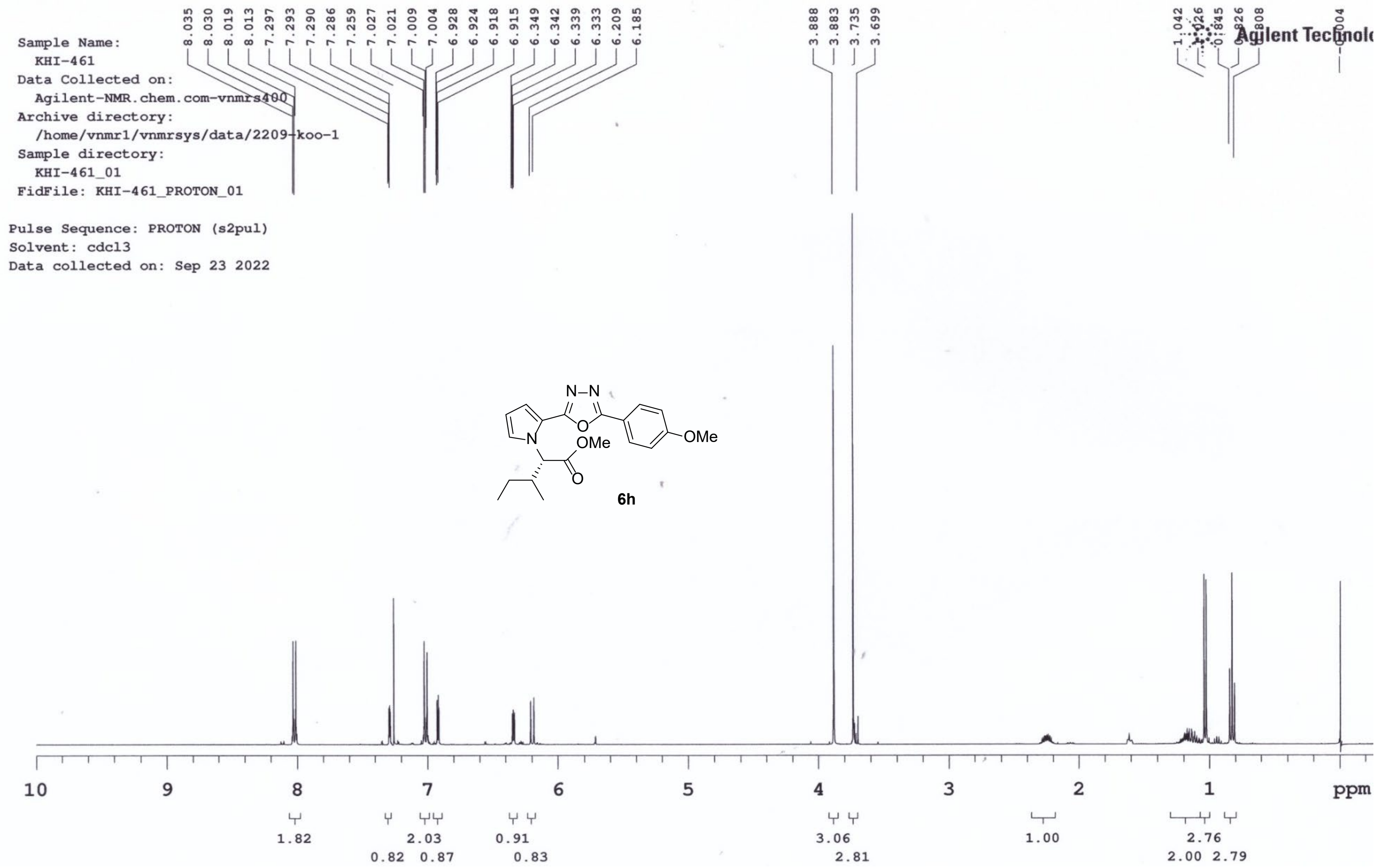
Sample Name:
KHI-461
Data Collected on:
Agilent-NMR.chem.com-vnmrs400
Archive directory:
/home/vnmr1/vnmrsys/data/2209-koo-1
Sample directory:
KHI-461_01
FidFile: KHI-461_PROTON_01
Pulse Sequence: PROTON (s2pul)
Solvent: cdcl3
Data collected on: Sep 23 2022
8.035 8.030 8.019 8.013 7.297 7.293 7.290 7.286 7.259 7.027 7.021 7.009 7.004 6.928 6.924 6.918 6.915 6.349 6.342 6.339 6.333 6.209 6.185
3.888 3.883 3.735 3.699
1.042 0.845 0.826
Agilent Technol
N–N
O
N
OMe
OMe
O
6h
10 9 8 7 6 5 4 3 2 1 ppm
1.82 0.82 2.03 0.87 0.91 0.83 3.06 2.81 1.00 2.76 2.00 2.79

Sample Name:
KHI-461-13C
Data Collected on:
Agilent-NMR.chem.com-vnmrs400
Archive directory:
/home/vnmr1/vnmrsys/data/2209-koo-1
Sample directory:
KHI-461-13C_01
FidFile: KHI-461-13C_CARBON_01

Pulse Sequence: CARBON (s2pul)
Solvent: cdcl3
Data collected on: Sep 26 2022

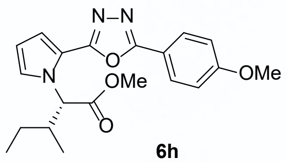

6h

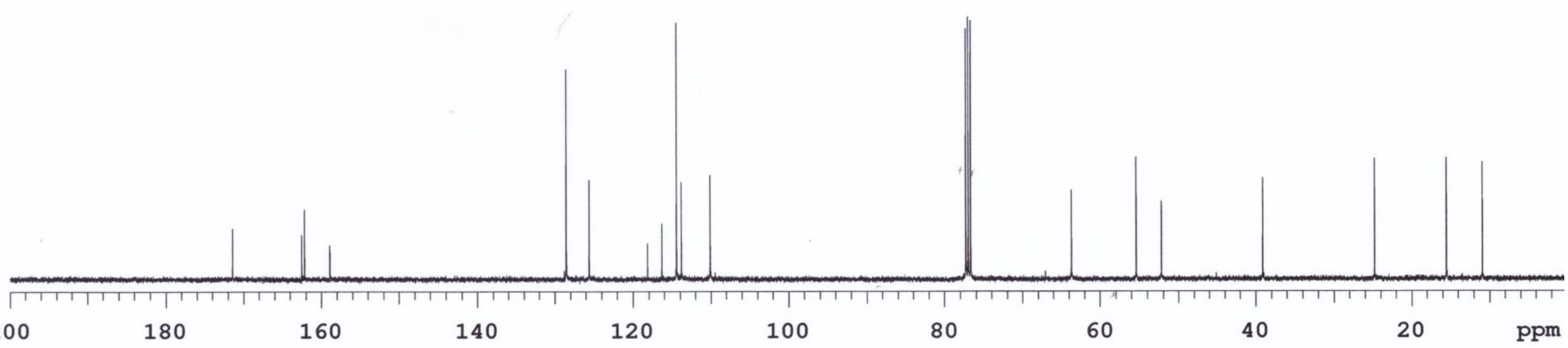

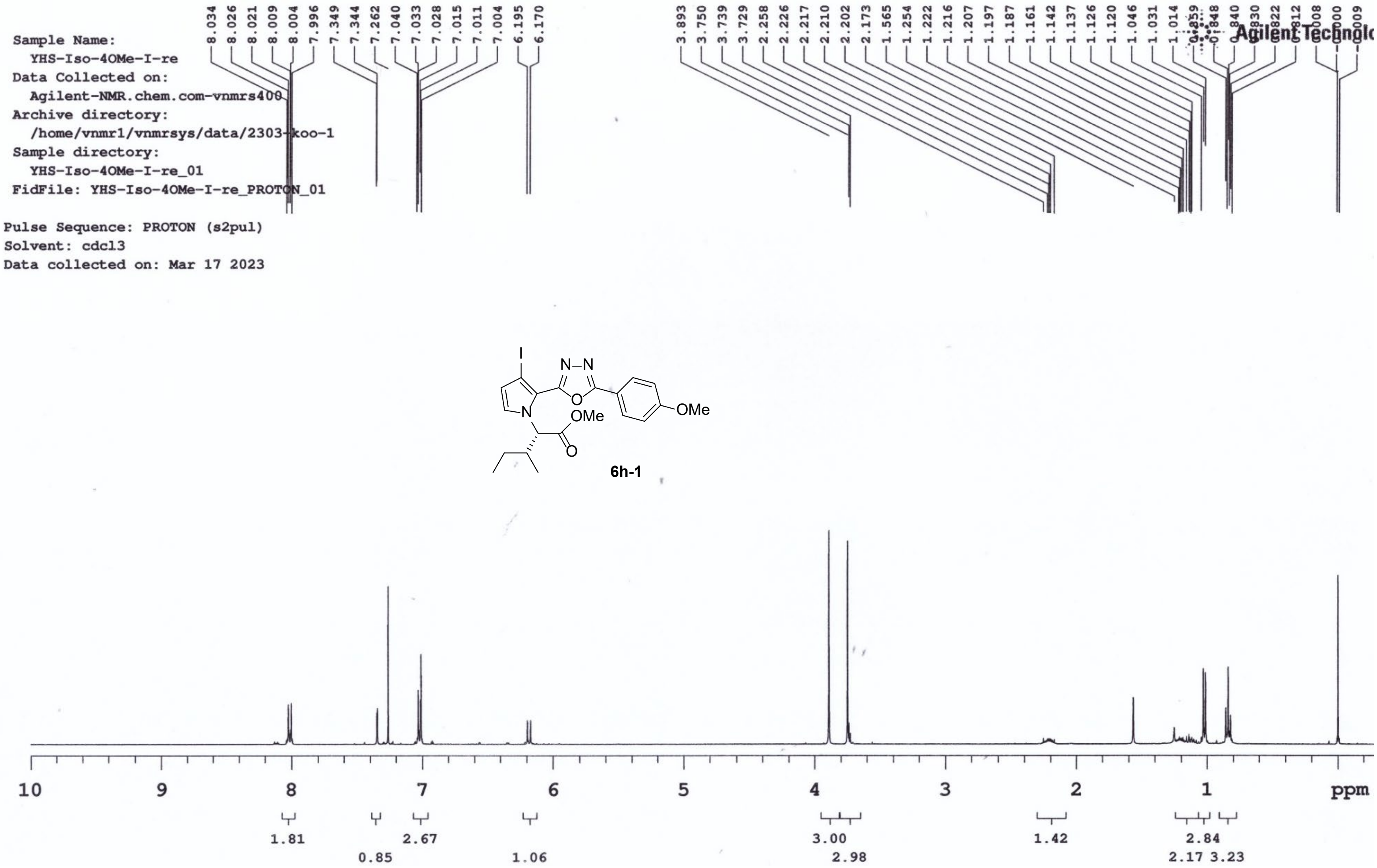
Sample Name:
YHS-Iso-4OMe-I-re
Data Collected on:
Agilent-NMR.chem.com-vnmrs400
Archive directory:
/home/vnmr1/vnmrsys/data/2303-koo-1
Sample directory:
YHS-Iso-4OMe-I-re_01
FidFile: YHS-Iso-4OMe-I-re_PROTON_01
Pulse Sequence: PROTON (s2pul)
Solvent: cdcl3
Data collected on: Mar 17 2023
8.034 8.026 8.021 8.009 8.004 7.996 7.349 7.344 7.262 7.040 7.033 7.028 7.015 7.011 7.004 6.195 6.170
3.893 3.750 3.739 3.729 2.258 2.226 2.217 2.210 2.202 2.173 1.565 1.254 1.222 1.216 1.207 1.197 1.187 1.161 1.142 1.137 1.126 1.120 1.046 1.031 1.014 0.848 0.840 0.830 0.822 0.812 0.000 -0.009
10 9 8 7 6 5 4 3 2 1 ppm
1.81 0.85 2.67 1.06 3.00 2.98 1.42 2.84 2.17 3.23
I
N–N
O
N
OMe
O
OMe

6h-1

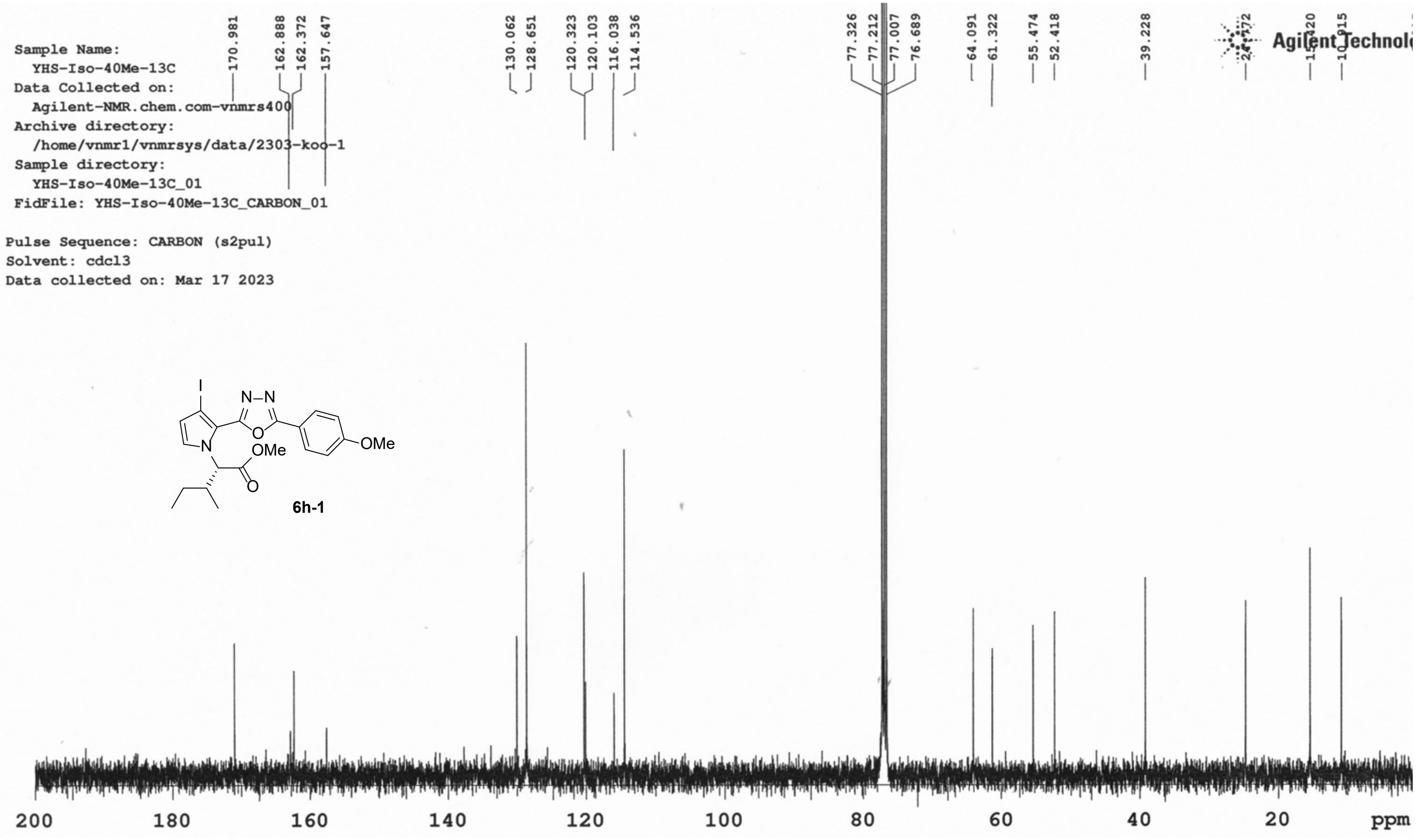
Sample Name:
YHS-Iso-40Me-13C
Data Collected on:
Agilent-NMR.chem.com-vnmrs400
Archive directory:
/home/vnmr1/vnmrsys/data/2303-koo-1
Sample directory:
YHS-Iso-40Me-13C_01
FidFile: YHS-Iso-40Me-13C_CARBON_01
Pulse Sequence: CARBON (s2pul)
Solvent: cdcl3
Data collected on: Mar 17 2023
170.981
162.888
162.372
157.647
130.062
128.651
120.323
120.103
116.038
114.536
77.326
77.212
77.007
76.689
64.091
61.322
55.474
52.418
39.228
Agilent Technol
I
N–N
O
OMe
N
OMe
O
6h-1
200
180
160
140
120
100
80
60
40
20
ppm

Table S1. Determination of MIC for **1** for *E. coli* and *S. aureus* by OD_{600} (20 h). Each value was obtained as an average of at least triple measurements.

OD_{600}	Average / Standard Deviation	100% DMSO	2048 μg/mL	1024 μg/mL	512 μg/mL	256 μg/mL	128 μg/mL	64 μg/mL	32 μg/mL	16 μg/mL	8 μg/mL	4 μg/mL	Triple Distilled Water
**1a**	*E. coli*	0.3478	0.289233	0.8507	0.151167	0.3022	0.426367	0.331567	0.323967	0.309567	0.307567	0.279	0.654767
		0.030547	0.850022	0.714032	0.472498	0.163467	0.045185	0.03466	0.024543	0.017898	0.025528	0.007448	0.032528
	S. aureus	0.2505	-0.0939	0.27025	0.1779	0.2686	0.3424	0.2688	0.2575	0.2414	0.23315	0.226	0.5288
		0.000424	0.03182	0.215597	0.02291	0.011172	0.033517	0.019375	0.006788	0.018668	0.026941	0.004667	0.028567
**1b**	*E. coli*	0.33185	-0.5113	-0.47107	-0.24387	0.2371	0.323167	0.311667	0.3073	0.2944	0.298667	0.2851	0.644167
		0.03783	0.087665	0.099417	0.075585	0.057446	0.055494	0.053523	0.034963	0.031323	0.043386	0.046392	0.021711
	S. aureus	0.21535	-0.665	-0.7692	-0.58615	0.104	0.19055	0.18395	0.1696	0.17765	0.17695	0.18175	0.4575
		0.03684	0.032244	0.024324	0.008839	0.000141	0.010536	0.003748	0.006223	0.000778	0.003323	0.004455	0.005233
**1c**	*E. coli*	0.33135	-0.4664	-0.62733	-0.51347	-0.1628	0.101767	0.231333	0.288167	0.319667	0.3146	0.296833	0.646467
		0.035567	0.050321	0.02021	0.090596	0.036104	0.043366	0.043653	0.03173	0.036844	0.054097	0.050751	0.026931
	S. aureus	0.22985	-0.4684	-0.6106	-0.68205	-0.3628	-0.06505	0.10315	0.17225	0.19655	0.19245	0.18055	0.47835
		0.028214	0.069438	0.212698	0.2856	0.078065	0.003465	0.005445	0.006293	0.015061	0.012799	0.005162	0.008415
**1d**	*E. coli*	0.3297	-0.35697	-0.18807	0.0547	0.136567	0.245367	0.282333	0.313767	0.3293	0.3124	0.296633	0.655067
		0.035214	0.055124	0.079664	0.062815	0.025095	0.046661	0.036691	0.027534	0.029802	0.042749	0.051993	0.027386
	S. aureus	0.2168	-0.5331	-0.48685	-0.21885	-0.03435	0.0706	0.1423	0.1725	0.1717	0.1677	0.17205	0.4882
		0.032951	0.21793	0.358008	0.238224	0.061589	0.011879	0	0.000849	0.003111	0.007071	7.07E-05	0.037194
**1e**	*E. coli*	0.32355	-0.24903	-0.19493	-0.04953	0.048233	0.1481	0.2334	0.269967	0.305833	0.3231	0.313467	0.638467
		0.036982	0.031268	0.04301	0.06383	0.030179	0.031921	0.047883	0.031503	0.026131	0.039861	0.055979	0.029372
	S. aureus	0.21815	-0.2259	-0.16365	-0.13155	-0.08995	-0.0194	0.0853	0.14685	0.17255	0.1912	0.1758	0.47435
		0.015627	0.067458	0.081529	0.12042	0.114905	0.023759	0.009192	0.006718	0.010394	0.000283	0.003818	0.017183
**1f**	*E. coli*	0.33405	-0.2903	-0.256	0.178533	0.5189	0.447633	0.346633	0.299733	0.3095	0.323633	0.298933	0.646933
		0.052962	0.09575	0.036979	0.083553	0.039168	0.026536	0.041134	0.026318	0.031795	0.055569	0.049194	0.028221
	S. aureus	0.2372	-0.32965	-0.3046	0.035	0.21185	0.24615	0.2057	0.16555	0.17325	0.19085	0.1656	0.4664
		0.02588	0.135411	0.188939	0.238295	0.158321	0.056639	0.000566	0.010253	0.004172	0.002192	0.005233	0.050063
**1g**	*E. coli*	0.3458	-0.32957	-0.2146	0.271	0.461733	0.415333	0.347033	0.316833	0.3149	0.316267	0.298967	0.652733
		0.055296	0.084745	0.024778	0.038061	0.010671	0.036195	0.037208	0.024892	0.034566	0.046847	0.053796	0.024363

	S. aureus	0.23595	-0.3185	-0.25655	0.00045	0.15445	0.21035	0.18	0.17185	0.1809	0.2007	0.18405	0.45085
		0.022415	0.145381	0.155776	0.223516	0.119572	0.071771	0.020648	0.001626	0.000283	0.010748	0.020435	0.060033
**1h**	*E. coli*	0.3743	-0.14843	0.286733	0.337267	0.358567	0.368367	0.3572	0.351733	0.356733	0.323433	0.2907	0.659467
		0.091924	0.144926	0.07582	0.027097	0.04007	0.042204	0.04702	0.027555	0.064329	0.039783	0.073212	0.006788
	S. aureus	0.26115	-0.16995	0.0988	0.1624	0.2799	0.24735	0.2239	0.2181	0.21875	0.21805	0.20855	0.5153
		0.026234	0.050417	0.082307	0.055296	0.014425	0.015344	0.016971	0.037477	0.023547	0.005303	0.003465	0.086833
**1i**	*E. coli*	0.29705	-0.07643	0.302533	0.2503	0.287533	0.285167	0.284033	0.2734	0.28	0.277267	0.280767	0.601667
		0.045043	0.134622	0.062365	0.014714	0.004665	0.008344	0.01954	0.009896	0.01815	0.020188	0.026755	0.007808
	S. aureus	0.25425	-0.08225	0.13875	0.22455	0.28055	0.24695	0.2318	0.2303	0.2213	0.23295	0.2204	0.55235
		0.047023	0.236386	0.169776	0.032032	0.029486	0.008415	0.002263	0.015556	0.017253	0.019304	0.006364	0.057629
**1j**	*E. coli*	0.29785	0.102133	0.321467	0.2305	0.225867	0.257767	0.286467	0.282867	0.2803	0.284767	0.2814	0.626967
		0.04278	0.052547	0.018309	0.020705	0.009965	0.006839	0.020582	0.016959	0.018865	0.019558	0.039122	0.019843
	S. aureus	0.2189	-0.0115	0.12025	0.1411	0.1658	0.2065	0.2115	0.2006	0.20105	0.1877	0.19245	0.5254
		0.023476	0.08457	0.098641	0.014142	0.038467	0.00099	0.001273	0.008627	0.001202	0.002828	0.008839	0.009617
**1k**	*E. coli*	0.3603	-1.5086	-1.35513	-0.6903	-0.04887	0.292933	0.324833	0.314667	0.340767	0.308833	0.303367	0.665167
		0.102248	0.072676	0.043698	0.248257	0.061502	0.104457	0.030421	0.019211	0.042656	0.033313	0.026279	0.028111
	S. aureus	0.2184	-1.61145	-1.4803	-0.8555	-0.1416	0.1075	0.19625	0.1868	0.19645	0.1896	0.20035	0.5429
		0.004243	0.167655	0.087116	0.24876	0.128835	0.042709	0.012092	0.014142	0.003889	0.007212	0.002616	0.000707

Table S2. Determination of MIC for **5** and **6** for *E. coli*, *S. aureus,* and *A. baumannii* by OD_{600} (20 h). Each value was obtained as an average of at least triple measurements (vancomycin and erythromycin as positive controls).

OD_{600}	Average / Standard Deviation	5% DMSO	1024 μg/mL	512 μg/mL	256 μg/mL	128 μg/mL	64 μg/mL	32 μg/mL	16 μg/mL	8 μg/mL	4 μg/mL	2 μg/mL	Triple Distilled Water
**5a** *o*-F	*E. coli*	0.5663	-0.0935	-0.00173	0.181667	0.269833	0.5345	0.525867	0.559767	0.496167	0.6068	0.317	0.420467
		0.004779	0.031166	0.056804	0.043941	0.06155	0.022098	0.025765	0.020482	0.156749	0.137563	0.400098	0.236646
	S. aureus	0.4818	0.069367	-0.02667	0.052733	0.117067	0.301067	0.2923	0.3547	0.380767	0.4321	0.339567	0.289467
		0.039473	0.004285	0.056203	0.072894	0.031306	0.018322	0.035823	0.050483	0.046983	0.384518	0.138847	0.327076
	A. baumannii	0.2678	-0.19767	-0.19657	-0.00073	-0.03173	0.253667	0.1872	0.217867	0.226933	0.053	0.22915	-0.09783
		0.028071	0.193864	0.167014	0.115973	0.022621	0.143279	0.01693	0.022806	0.031214	0.073103	0.394707	0.070474
**5b** *m*-F	*E. coli*	0.585533	0.131	0.198133	0.317167	0.394467	0.4791	0.5444	0.571467	0.6097	0.4236	0.631833	0.5571
		0.032805	0.119208	0.189769	0.039728	0.018809	0.010886	0.009372	0.021289	0.029182	0.188151	0.027663	0.098856
	S. aureus	0.487633	0.102767	0.1662	0.246933	0.279233	0.279633	0.329767	0.332633	0.375433	0.3622	0.415733	0.359433
		0.029329	0.001986	0.085839	0.023332	0.024953	0.029252	0.016905	0.018159	0.01323	0.038219	0.09099	0.406902
	A. baumannii	0.134133	-0.01857	0.0047	0.113567	0.198433	0.227733	0.217367	0.2452	0.238567	0.211633	0.202567	0.481167
		0.169371	0.028894	0.066938	0.030551	0.008556	0.017352	0.028045	0.018368	0.031305	0.005845	0.07391	0.173203
**5c** *p*-F	*E. coli*	0.548433	0.151733	0.236633	0.3586	0.429	0.503267	0.540967	0.561633	0.561433	0.152067	0.567367	0.496467
		0.021914	0.026979	0.069258	0.069205	0.020637	0.018661	0.014262	0.025335	0.031601	0.409621	0.036247	0.141964
	S. aureus	0.441133	0.058333	0.1148	0.169867	0.254333	0.2757	0.334067	0.372167	0.380333	0.1813	0.289833	0.602933
		0.010098	0.03269	0.010253	0.029358	0.006962	0.011774	0.023027	0.046016	0.041734	0.271455	0.234179	0.076836
	A. baumannii	0.2341	-0.05587	-0.02777	0.0701	0.115767	0.179367	0.240467	0.260667	0.279733	0.0995	0.121733	0.623067
		0.024456	0.021574	0.044891	0.026683	0.025335	0.041527	0.024751	0.113443	0.11848	0.099321	0.127833	0.285097

OD$_{600}$	Average / Standard Deviation	5% DMSO	1024 µg/mL	512 µg/mL	256 µg/mL	128 µg/mL	64 µg/mL	32 µg/mL	16 µg/mL	8 µg/mL	4 µg/mL	2 µg/mL	Triple Distilled Water
**5d** *p*-Cl	*E. coli*	0.579033	-0.14127	0.0054	0.3527	0.474933	0.5682	0.589033	0.598533	0.596467	0.5875	0.594733	0.648133
		0.053497	0.04099	0.118053	0.035666	0.055678	0.021054	0.007596	0.026585	0.008654	0.013396	0.04294	0.37161
	S. aureus	0.447033	-0.2376	-0.08727	-0.0187	0.254167	0.300367	0.380367	0.336767	0.3453	0.3313	0.080633	0.525
		0.004936	0.032193	0.043104	0.007732	0.051822	0.026651	0.021373	0.014327	0.013305	0.065115	0.413322	0.202245
	A. baumannii	0.246833	-0.16697	-0.12503	0.153567	0.236833	0.242933	0.371167	0.223533	0.2197	0.229133	0.174033	0.527267
		0.026606	0.031151	0.05423	0.071986	0.082996	0.026258	0.014022	0.012529	0.01149	0.015652	0.059859	0.164986
**5e-1** *o*-OH	*E. coli*	0.584033	-0.00397	0.1366	0.406833	0.4453	0.535633	0.6021	0.555633	0.353433	0.4878	0.3224	0.337533
		0.036057	0.049138	0.015221	0.03456	0.025638	0.028504	0.02205	0.014318	0.368088	0.128463	0.126218	0.216063
	S. aureus	0.4603	-0.12153	-0.33813	-0.1466	-0.10913	-0.0546	-0.03017	-0.0187	-0.00907	0.031433	0.3362	0.4777
		0.028008	0.010365	0.117167	0.047326	0.011721	0.011049	0.003121	0.00423	0.094579	0.432203	0.039103	0.190024
	A. baumannii	0.257133	-0.1009	-0.18663	-0.11497	0.078667	0.021633	0.196133	0.193967	0.121967	0.0963	0.234567	0.141767
		0.019135	0.020148	0.108839	0.076689	0.068777	0.085558	0.1074	0.040734	0.128972	0.337207	0.156701	0.163562
**5f-1** *p*-OH	*E. coli*	0.610767	-0.04497	0.304967	0.444	0.347633	0.4712	0.469867	0.571967	0.465867	0.376667	0.2635	0.494967
		0.032207	0.025902	0.055612	0.05995	0.121436	0.02763	0.145089	0.037334	0.227569	0.336678	0.384056	0.265685
	S. aureus	0.476367	-0.11507	-0.22337	-0.14757	-0.13423	0.039433	-0.0043	-0.28307	-0.22003	-0.34043	0.289467	0.242267
		0.011828	0.126333	0.225514	0.130583	0.10695	0.159668	0.028178	0.258498	0.206889	0.435493	0.126238	0.289827
	A. baumannii	0.207467	-0.09443	-0.25543	-0.13213	0.002233	-0.12997	-0.02577	0.001133	-0.01047	-0.25243	-0.00337	0.140033
		0.03379	0.115879	0.186161	0.053673	0.036527	0.30727	0.150633	0.393035	0.266581	0.443574	0.270916	0.268719

OD600	Average / Standard Deviation	5% DMSO	1024 µg/mL	512 µg/mL	256 µg/mL	128 µg/mL	64 µg/mL	32 µg/mL	16 µg/mL	8 µg/mL	4 µg/mL	2 µg/mL	Triple Distilled Water
**5f** *o*-OH	*E. coli*	0.5628	0.1427	0.2282	0.269467	0.3946	0.5437	0.607867	0.6051	0.617233	0.584533	0.576067	0.6816
		0.00374	0.018266	0.074549	0.041967	0.047099	0.029865	0.032888	0.039824	0.040086	0.033624	0.010333	0.029025
	S. aureus	0.4341	-0.1521	-0.1373	-0.14833	0.027633	0.136333	0.195033	0.0272	0.268333	0.239333	0.389833	0.5614
		0.010235	0.069882	0.085083	0.078398	0.059014	0.028494	0.027081	0.240389	0.075575	0.130233	0.021768	0.051241
	A. baumannii	0.203633	-0.0818	-0.09567	-0.1738	-0.0636	0.163067	0.207967	0.164	0.1454	0.102533	0.0891	0.6636
		0.018188	0.079953	0.086918	0.085729	0.056571	0.022547	0.048014	0.132417	0.143797	0.123057	0.250629	0.173474
**5g** *o*-OMe	*E. coli*	0.5669	0.1844	0.274633	0.378867	0.4271	0.511633	0.564233	0.582333	0.580233	0.5568	0.540567	0.1807
		0.019918	0.017485	0.0352	0.002577	0.019901	0.018367	0.008656	0.00369	0.015509	0.014681	0.027802	0.38267
	S. aureus	0.480267	0.038367	0.057167	0.147533	0.1874	0.2207	0.2516	0.2973	0.324133	0.257033	0.276667	0.207167
		0.009955	0.01766	0.044252	0.036703	0.03826	0.042948	0.018	0.02287	0.030935	0.192673	0.131945	0.386512
	A. baumannii	0.211433	-0.0343	-0.00167	0.051733	0.106567	0.1529	0.211833	0.220733	-0.2874	0.114633	0.1702	0.668733
		0.020501	0.021118	0.033471	0.018607	0.028527	0.02335	0.0195	0.020325	0.448755	0.12236	0.082064	0.043359
**5g-1** *o*-OMe	*E. coli*	0.5811	0.089967	-0.06553	0.514867	0.461267	0.558367	0.622933	0.644933	0.574267	0.599167	0.5497	0.3087
		0.011607	0.305614	0.172808	0.329993	0.112704	0.037812	0.012552	0.019053	0.119493	0.060493	0.07266	0.504621
	S. aureus	0.151767	-0.4264	-0.19373	0.1073	0.214567	0.1032	0.052933	0.218667	0.2879	0.271567	0.035433	0.574567
		0.228957	0.252254	0.209641	0.063035	0.0491	0.245718	0.380542	0.175763	0.041644	0.068112	0.419599	0.032973
	A. baumannii	0.2284	-0.28307	-0.2196	-0.00743	0.119033	0.201433	0.294033	0.3238	0.0616	0.1952	0.1544	0.1093
		0.038088	0.172324	0.130771	0.124592	0.093969	0.094973	0.042036	0.523788	0.309035	0.174984	0.114535	0.335283

OD$_{600}$	Average / Standard Deviation	5% DMSO	1024 µg/mL	512 µg/mL	256 µg/mL	128 µg/mL	64 µg/mL	32 µg/mL	16 µg/mL	8 µg/mL	4 µg/mL	2 µg/mL	Triple Distilled Water
**5h** *p*-OMe	*E. coli*	0.6065	0.2759	0.2894	0.388967	0.425267	0.463333	0.539967	0.5749	0.589633	0.599633	0.297133	0.665533
		0.043278	0.033042	0.089637	0.024927	0.008003	0.019803	0.014063	0.013524	0.028575	0.029255	0.480936	0.012708
	S. aureus	0.232567	0.0714	0.074533	0.105967	0.1629	0.1887	0.230767	0.265933	0.285	0.349367	0.323133	0.4444
		0.202334	0.058215	0.095632	0.044127	0.023381	0.012562	0.008674	0.005498	0.014389	0.015651	0.078875	0.134025
	A. baumannii	0.231867	0.022633	-0.00797	0.059267	0.1346	0.1992	0.2569	0.2641	0.258533	0.260933	0.225033	0.376767
		0.034406	0.05872	0.021548	0.056912	0.01599	0.014936	0.022995	0.017201	0.011692	0.003873	0.041971	0.430816
**5h-1** *p*-OMe	*E. coli*	0.582233	-0.6511	-0.31983	0.321533	0.2436	0.3794	0.587367	0.596533	0.592	0.481	0.515933	0.680767
		0.015767	0.548606	0.41569	0.23707	0.390896	0.21503	0.04358	0.057936	0.012968	0.20526	0.089811	0.020469
	S. aureus	0.4426	-0.74527	-0.58953	0.0805	0.188533	0.2749	0.1625	0.1872	0.295733	0.3309	0.2201	0.5721
		0.019235	0.529512	0.517887	0.090981	0.099146	0.078272	0.125207	0.078118	0.062152	0.048683	0.322145	0.041743
	A. baumannii	0.135633	-0.24467	-0.56443	-0.21503	-0.01423	0.130133	0.122467	0.336233	0.181967	0.0584	0.290167	0.782967
		0.069146	0.244337	0.015529	0.30294	0.300765	0.090814	0.253604	0.152639	0.130578	0.338963	0.015167	0.018312
**6a** *o*-F	*E. coli*	0.563267	0.013467	0.055867	0.2771	0.405467	0.493067	0.5474	0.602033	0.590133	0.495167	0.497233	0.5904
		0.014833	0.059975	0.039829	0.071188	0.061659	0.028513	0.02019	0.023157	0.011302	0.082901	0.070453	0.060836
	S. aureus	0.3412	-0.1639	-0.07717	0.063533	0.145033	0.205	0.268267	0.316	0.322533	0.3593	0.402567	0.487833
		0.235327	0.034927	0.061558	0.06494	0.026627	0.037385	0.030399	0.042011	0.048313	0.047546	0.035848	0.218122
	A. baumannii	0.227233	-0.07443	-0.0908	-0.01367	0.101867	0.183	0.173467	0.266	0.228667	0.2411	-0.09203	0.661067
		0.050281	0.046175	0.024187	0.012133	0.023599	0.073893	0.041175	0.037129	0.016805	0.027742	0.061919	0.010594

OD_{600}	Average / Standard Deviation	5% DMSO	1024 µg/mL	512 µg/mL	256 µg/mL	128 µg/mL	64 µg/mL	32 µg/mL	16 µg/mL	8 µg/mL	4 µg/mL	2 µg/mL	Triple Distilled Water
**6b** *m*-F	***E. coli***	0.580167	0.068733	0.103333	0.319667	0.410067	0.5444	0.560233	0.5988	0.5935	0.6024	0.4003	0.6172
		0.016053	0.049793	0.042423	0.014895	0.04715	0.01473	0.00889	0.021391	0.0218	0.004951	0.338978	0.089482
	S. aureus	0.3399	-0.17217	-0.02323	0.0732	0.153733	0.243367	0.2494	0.2927	0.2784	0.322467	0.309233	0.415967
		0.09231	0.066646	0.076326	0.050608	0.018056	0.0171	0.012946	0.011887	0.013586	0.049838	0.122567	0.12531
	A. baumannii	0.052033	-0.18307	-0.0763	0.012967	0.141433	0.348033	0.2747	0.349567	0.2578	0.272667	0.0699	0.645967
		0.133533	0.044449	0.057484	0.058517	0.037086	0.021735	0.05032	0.04814	0.01255	0.015233	0.276596	0.021989
**6c** *p*-F	***E. coli***	0.533967	0.144567	0.2717	0.413133	0.4972	0.564533	0.6045	0.614467	0.6126	0.590133	0.553333	0.647433
		0.059126	0.077264	0.030135	0.022098	0.016179	0.005781	0.041701	0.034766	0.029313	0.008946	0.030374	0.017528
	S. aureus	0.389267	0.082067	0.088433	0.180133	0.227167	0.276667	0.3111	0.328633	0.3426	0.3163	0.0376	0.683933
		0.196708	0.010492	0.038193	0.03482	0.03857	0.038653	0.025268	0.039157	0.035958	0.130464	0.314667	0.034948
	A. baumannii	0.212767	-0.05533	-0.04417	0.101167	0.129267	0.236533	0.2822	0.240533	0.267933	0.2421	0.242267	0.303567
		0.03289	0.034027	0.009846	0.018472	0.011427	0.035897	0.056385	0.04485	0.022347	0.037164	0.008719	0.2164
**6d** *p*-Cl	***E. coli***	0.5225	-0.05783	0.047933	0.3123	0.4468	0.538733	0.575433	0.599233	0.600867	0.6024	0.6279	0.669533
		0.073983	0.035173	0.034061	0.047372	0.049501	0.009252	0.008523	0.013413	0.01839	0.003559	0.02374	0.004352
	S. aureus	0.523767	-0.14937	-0.0628	0.1049	0.206933	0.279	0.311467	0.340467	0.353933	0.373833	0.3242	0.3406
		0.017647	0.053102	0.040789	0.029518	0.016909	0.018285	0.0052	0.019755	0.022413	0.042236	0.140104	0.337874
	A. baumannii	0.128867	-0.23597	-0.0958	0.087667	0.1727	0.251267	0.273167	0.2813	0.280467	0.285567	0.281733	0.652833
		0.254936	0.020526	0.034295	0.026702	0.027623	0.041322	0.026016	0.006437	0.016511	0.014398	0.023003	0.024892

OD$_{600}$	Average / Standard Deviation	5% DMSO	1024 μg/mL	512 μg/mL	256 μg/mL	128 μg/mL	64 μg/mL	32 μg/mL	16 μg/mL	8 μg/mL	4 μg/mL	2 μg/mL	Triple Distilled Water
**6e-1** *o*-OH	*E. coli*	0.482667	-0.03527	0.174567	0.5121	0.4774	0.488033	0.553267	0.66	0.6265	0.5765	0.589333	0.579333
		0.007044	0.026736	0.0824	0.088496	0.08139	0.10833	0.1059	0.032215	0.041559	0.058123	0.047768	0.106689
	S. aureus	0.409867	-0.06263	-0.1663	-0.32883	-0.1252	-0.05663	-0.06527	-0.02003	-0.7117	0.0345	-0.032	0.200967
		0.115135	0.031599	0.168687	0.25205	0.143971	0.016998	0.057151	0.007566	0.052551	0.13493	0.277024	0.388297
	A. baumannii	0.2263	-0.09757	0.0136	0.113933	-0.03577	-0.07657	0.0375	0.218233	-0.6665	0.2268	0.202433	0.403367
		0.014309	0.009657	0.079975	0.030953	0.228161	0.260033	0.162924	0.042339	0.023822	0.024996	0.063747	0.245056
**6e** *o*-OH	*E. coli*	0.550533	-0.71263	-0.6685	-0.2245	0.193367	0.423567	0.621433	0.663833	0.641033	0.620533	0.476133	0.6734
		0.079447	0.043027	0.087305	0.077585	0.05575	0.053036	0.025778	0.068848	0.029336	0.014524	0.092484	0.006509
	S. aureus	0.432267	-1.35967	-1.18427	-0.76853	-0.43957	0.055967	0.2072	0.211067	0.265833	0.3209	0.3583	0.538633
		0.031379	0.018284	0.139737	0.0292	0.139406	0.049015	0.014823	0.011113	0.013699	0.054685	0.042579	0.055584
	A. baumannii	0.193033	-1.30287	-1.08353	-0.5868	-0.2114	-0.0019	0.1532	0.221233	0.2962	0.323867	0.297067	0.824367
		0.04258	0.074684	0.035826	0.146309	0.056868	0.087508	0.125707	0.069535	0.03509	0.046317	0.036712	0.017131
**6f-1** *p*-OH	*E. coli*	0.555333	0.038167	0.222533	0.456233	0.281633	0.546267	0.433933	0.5596	0.6226	0.4277	0.637433	0.692567
		0.024134	0.031092	0.25179	0.030192	0.196759	0.033515	0.196451	0.088235	0.020352	0.241129	0.008542	0.009547
	S. aureus	0.441433	-0.1143	-0.21467	-0.28177	-0.2246	-0.29967	-0.40083	-0.2534	-0.3732	-0.2752	-0.4245	0.065033
		0.043428	0.026457	0.133408	0.164958	0.34139	0.451706	0.395107	0.274966	0.24689	0.395395	0.311968	0.521248
	A. baumannii	0.210167	-0.08037	-0.06343	0.022367	-0.08597	0.095833	-0.33263	-0.40203	-0.16833	0.2602	0.1809	0.6579
		0.061093	0.038218	0.030068	0.03716	0.17361	0.079832	0.246976	0.12486	0.456569	0.017804	0.09659	0.068942

OD600	Average / Standard Deviation	5% DMSO	1024 µg/mL	512 µg/mL	256 µg/mL	128 µg/mL	64 µg/mL	32 µg/mL	16 µg/mL	8 µg/mL	4 µg/mL	2 µg/mL	Triple Distilled Water
**6f** *p*-OH	*E. coli*	0.580333	-0.55103	0.123533	0.5112	0.630767	0.516333	0.6104	0.622433	0.382067	0.644033	0.635333	0.6159
		0.005499	0.050954	0.051951	0.034334	0.032991	0.16293	0.022578	0.017579	0.42362	0.027766	0.054606	0.184714
	S. aureus	0.467367	-0.81597	-0.25553	0.083433	0.0425	0.0302	0.0004	-0.00203	-0.37443	0.2321	0.2364	0.5281
		0.024451	0.082553	0.071841	0.050228	0.042171	0.014697	0.00466	0.000416	0.50661	0.019489	0.157819	0.08503
	A. baumannii	0.2466	-0.77193	-0.2757	0.1809	0.2156	0.156367	0.004967	0.2751	0.1911	0.0836	0.066467	0.814933
		0.006974	0.079612	0.144879	0.037529	0.033036	0.054103	0.345068	0.229765	0.051765	0.139431	0.193652	0.098204
**6g** *o*-OMe	*E. coli*	0.514467	0.1212	0.256367	0.381667	0.5117	0.583033	0.617733	0.619233	0.625867	0.6043	0.509067	0.643467
		0.029871	0.041836	0.050119	0.025005	0.041626	0.031352	0.017664	0.013107	0.008832	0.00318	0.156222	0.062531
	S. aureus	0.351333	0.000967	0.086433	0.153367	0.221033	0.2471	0.282867	0.2767	0.274767	0.1091	0.4166	0.478733
		0.054321	0.08218	0.029258	0.037778	0.046923	0.04224	0.044203	0.016877	0.08942	0.238055	0.012454	0.201722
	A. baumannii	0.215233	-0.0594	-0.01203	0.060067	0.136333	0.193233	0.241033	0.240833	0.244267	0.2418	0.253933	0.3972
		0.007883	0.013868	0.01411	0.027256	0.025108	0.024043	0.012936	0.016836	0.027722	0.030545	0.010719	0.36455
**6h** *p*-OMe	*E. coli*	0.5625	0.141167	0.276133	0.368633	0.467233	0.541333	0.5983	0.623833	0.6327	0.394467	0.4689	0.509133
		0.036105	0.05724	0.00245	0.045647	0.033821	0.011793	0.01515	0.004051	0.003122	0.338243	0.282118	0.137559
	S. aureus	0.4173	-0.04433	0.016167	0.099667	0.1279	0.194733	0.22	0.2507	0.268333	0.317967	0.377733	0.564767
		0.153597	0.024286	0.031005	0.041051	0.038384	0.028184	0.010567	0.007572	0.017802	0.024059	0.029382	0.022851
	A. baumannii	0.194567	-0.06657	-0.05227	0.065067	0.161333	0.2098	0.2658	0.2876	0.302133	0.298467	0.279	0.753033
		0.083458	0.021736	0.041927	0.051276	0.036087	0.035985	0.035222	0.011166	0.030312	0.006313	0.02645	0.021247

OD_{600}	Average / Standard Deviation	5% DMSO	1024 µg/mL	512 µg/mL	256 µg/mL	128 µg/mL	64 µg/mL	32 µg/mL	16 µg/mL	8 µg/mL	4 µg/mL	2 µg/mL	Triple Distilled Water
6h-1 *p*-OMe	*E. coli*	0.602033	0.0413	0.178267	0.402767	0.562833	0.622233	0.625767	0.613133	0.386733	0.594333	0.629533	0.677833
		0.0141	0.043315	0.058042	0.055404	0.055585	0.018501	0.01123	0.059871	0.40273	0.070062	0.010652	0.008664
	S. aureus	0.474433	0.0119	0.0536	0.186533	0.256567	0.283067	0.275167	0.2778	0.2863	0.318	0.003033	0.516733
		0.039822	0.040041	0.0414	0.03982	0.024243	0.01216	0.005953	0.017562	0.021674	0.027731	0.405709	0.106848
	A. baumannii	0.270233	-0.01317	-0.00273	0.151333	0.228867	0.280867	0.2116	0.013733	0.2785	0.22545	0.0679	0.875633
		0.046391	0.089113	0.101433	0.119789	0.10617	0.025088	0.131107	0.401343	0.107985	0.21022	0.15906	0.059901

OD_{600}	Average / Standard Deviation	5% DMSO	1024 µg/mL	512 µg/mL	256 µg/mL	128 µg/mL	64 µg/mL	32 µg/mL	16 µg/mL	8 µg/mL	4 µg/mL	2 µg/mL	Triple Distilled Water
Vancomycin	*E. coli*	0.8013	0.4901	0.708433	0.752867	0.746967	0.742067	0.7827	0.772167	0.781967	0.855167	0.871733	0.605967
		0.039069	0.030212	0.022352	0.008937	0.021712	0.021561	0.048403	0.057	0.023133	0.091893	0.10263	0.00325
	S. aureus	0.461433	0.0395	0.0391	0.039233	0.039333	0.0396	0.039667	0.041167	0.041367	0.042967	0.278133	0.487167
		0.01291	0.000854	0.000819	0.000643	0.000379	0.000436	0.000643	0.001193	0.001922	0.003197	0.135305	0.025012
	A. baumannii	0.6197	0.141267	0.232567	0.4701	0.574167	0.59	0.609667	0.599567	0.594367	0.600067	0.585467	0.643733
		0.010897	0.027308	0.039094	0.042326	0.075942	0.048362	0.048319	0.054458	0.053718	0.054685	0.013527	0.011796
Erythromycin	*E. coli*	0.778333	0.098533	0.315367	0.4438	0.543867	0.666633	0.700233	0.797567	0.790167	0.743067	0.782833	0.607333
		0.024451	0.086119	0.025522	0.025811	0.028514	0.011809	0.03331	0.047561	0.077885	0.043578	0.01501	0.018113
	S. aureus	0.4564	0.038133	0.037767	0.038367	0.0383	0.038633	0.039733	0.040433	0.042333	0.0501	0.147433	0.466767
		0.034787	0.000839	0.000451	0.00085	0.000721	0.000874	0.001815	0.00168	0.002894	0.006022	0.049306	0.012881
	A. baumannii	0.616867	0.038133	0.0384	0.038333	0.0973	0.148733	0.184367	0.226433	0.3081	0.407467	0.4603	0.6226
		0.024035	0.00095	0.000781	0.000929	0.006762	0.009729	0.007223	0.015602	0.007879	0.018736	0.010046	0.005151

High resolution mass spectra (ESI)

1a: calcd for $C_{15}H_{13}N_3O_3$+Na, 306.0849, found 306.0851.

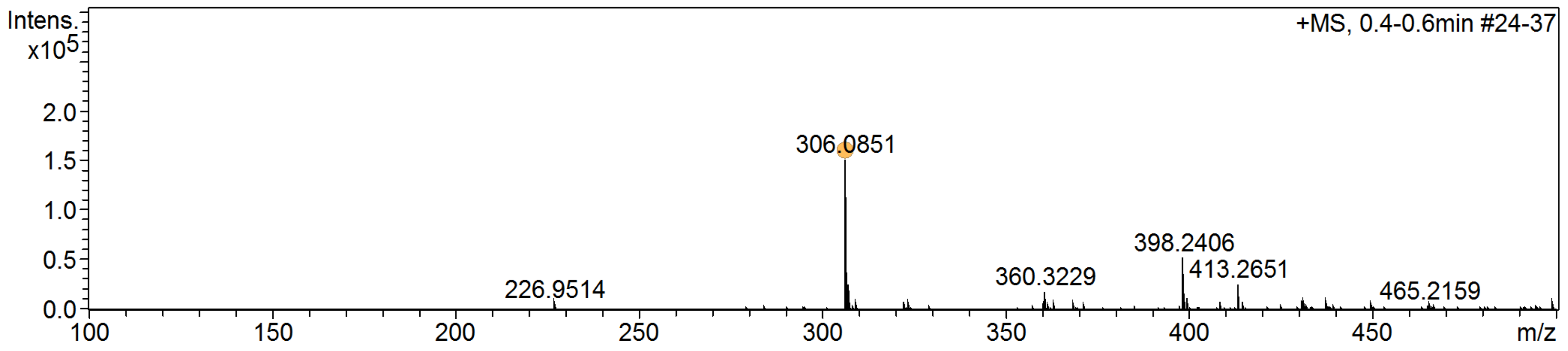

1b: calcd for $C_{16}H_{15}N_3O_3$+Na, 320.1006, found 320.1007.

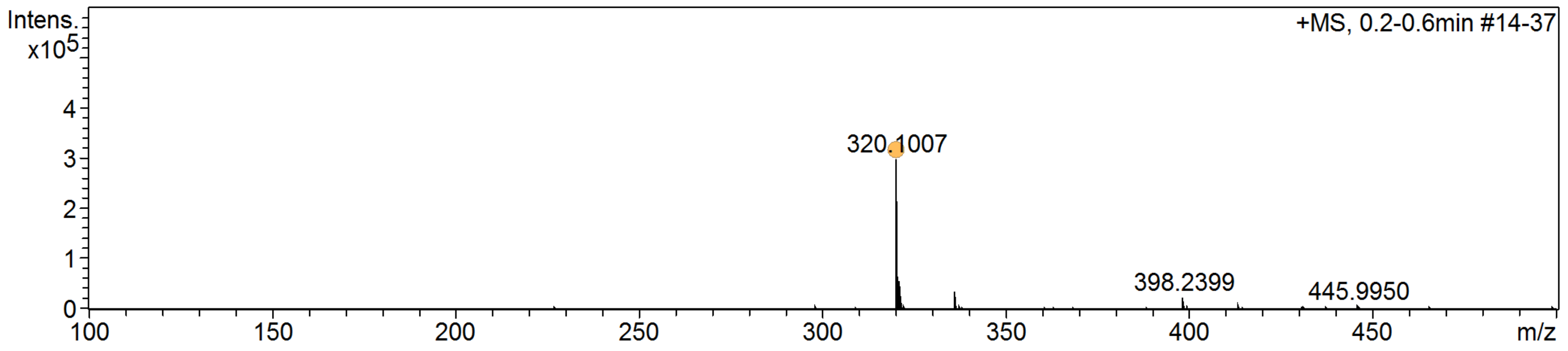

1c: calcd for $C_{18}H_{19}N_3O_3$+Na, 348.1319, found 348.1319.

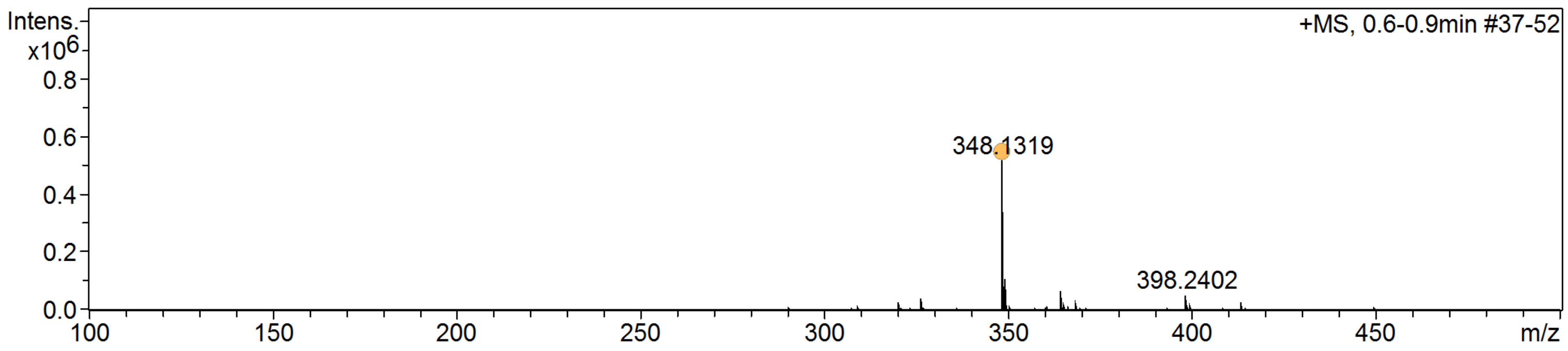

1d: calcd for $C_{19}H_{21}N_3O_3$+Na, 362.1475, found 362.1474.

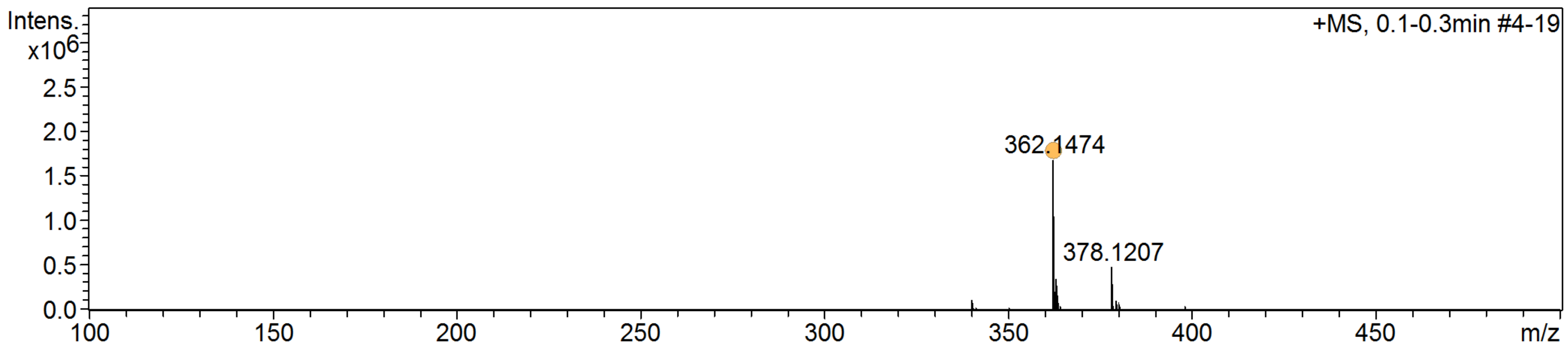

1e: calcd for $C_{19}H_{21}N_3O_3$+Na, 362.1475, found 362.1475.

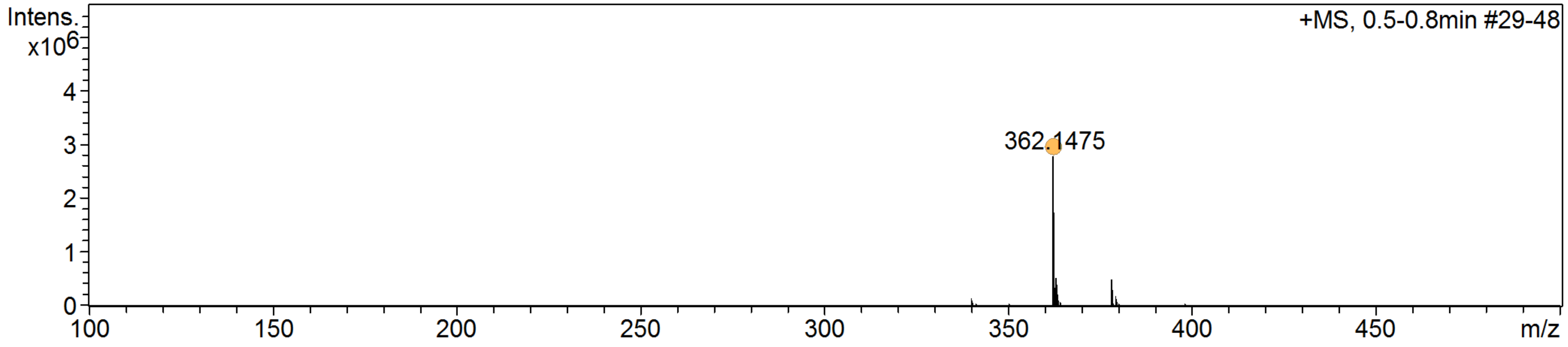

1f: calcd for $C_{22}H_{19}N_3O_3$+Na 396.1319, found 396.1321.

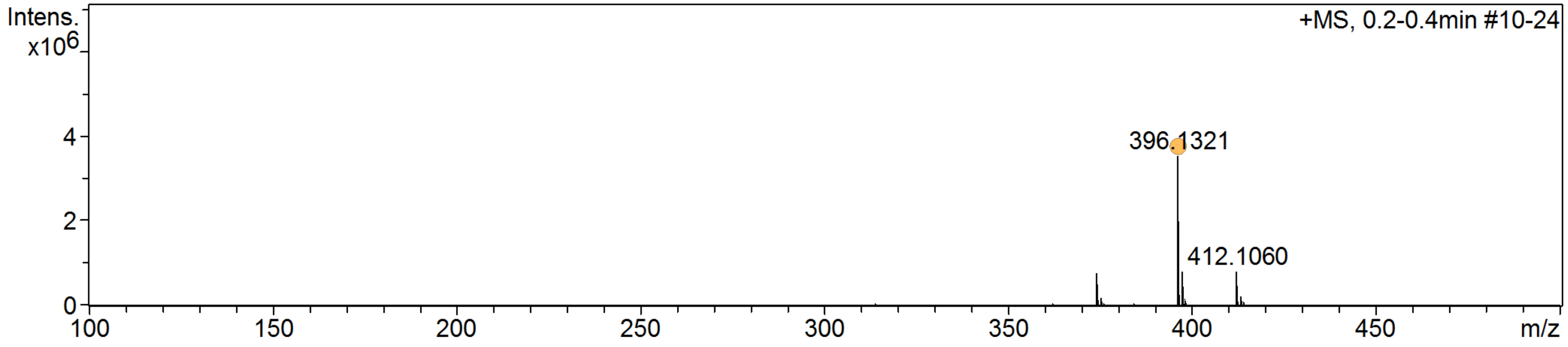

1g: calcd for $C_{23}H_{21}N_3O_3$+Na, 410.1475, found 410.1477.

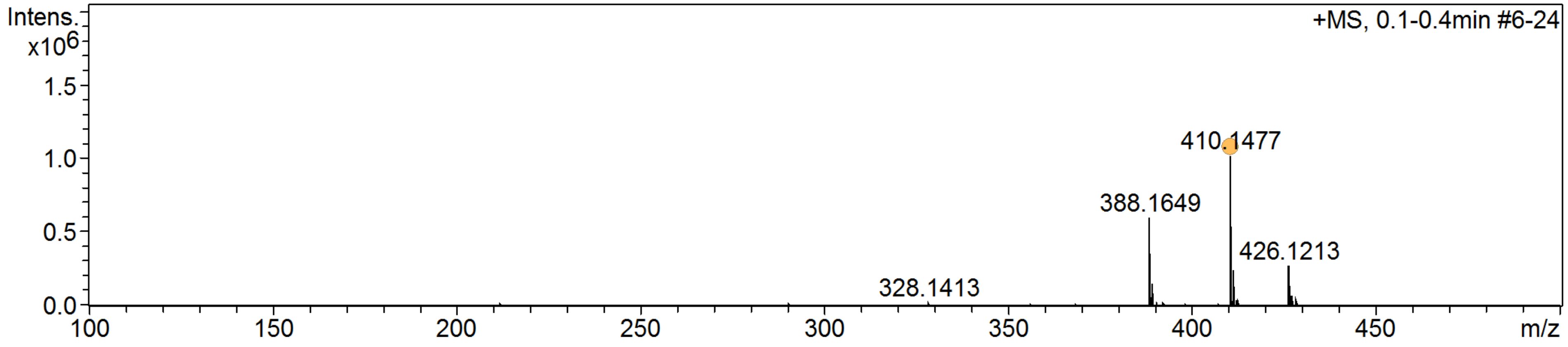

1h: calcd for $C_{18}H_{17}N_3O_5$+Na, 378.1060, found 378.1062.

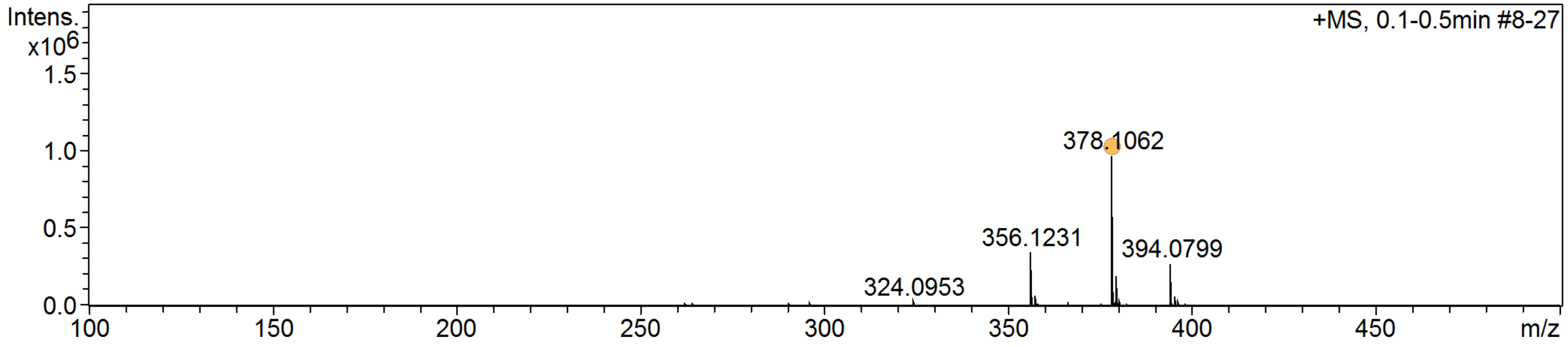

1i: calcd for $C_{19}H_{19}N_3O_5$+Na, 392.1217, found 392.1220.

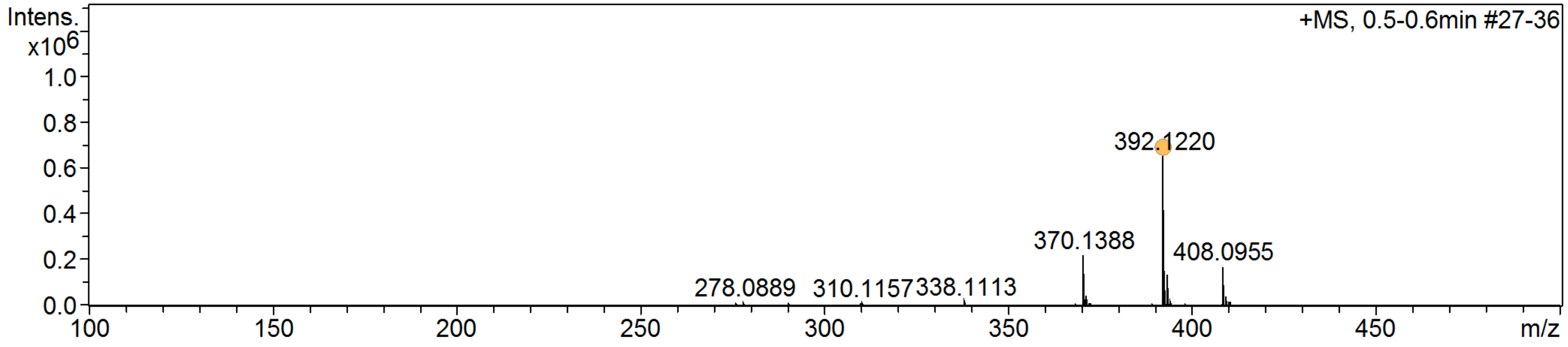

1j: calcd for $C_{18}H_{19}N_3O_3S$+Na, 380.1039, found 380.1040.

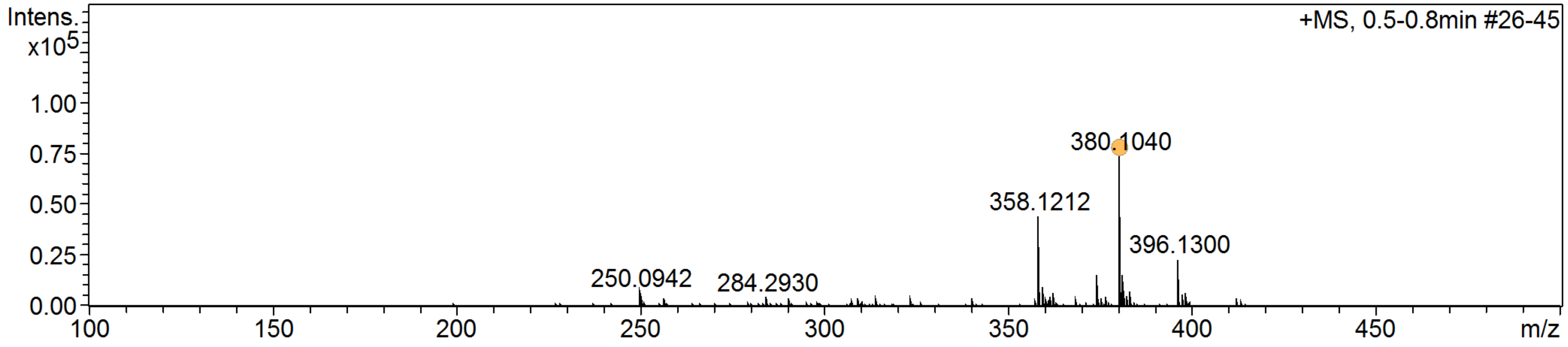

1k: calcd for $C_{17}H_{15}N_3O_3S$+Na, 364.0726, found 364.0729.

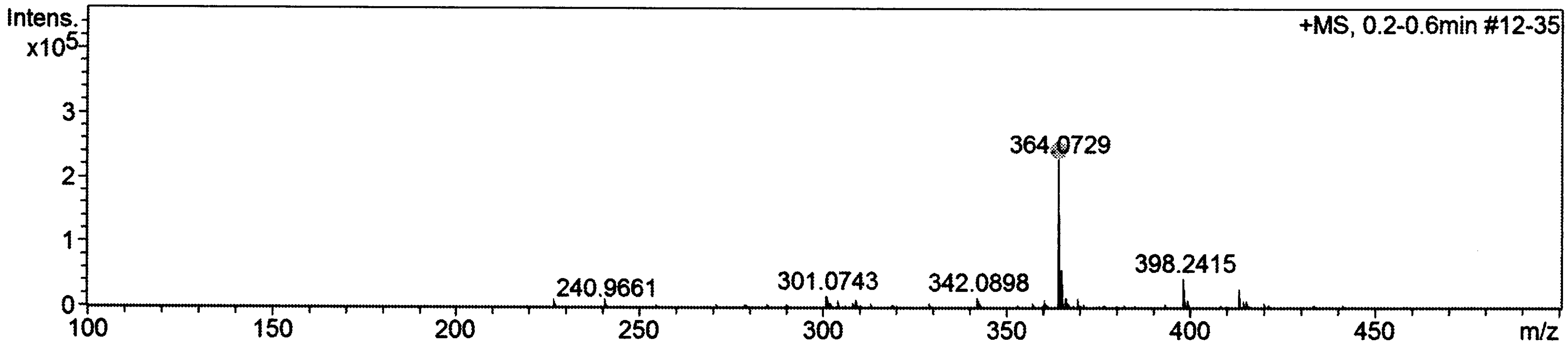

5a: calcd for $C_{18}H_{18}FN_3O_3$+Na 366.1224, found 366.1225.

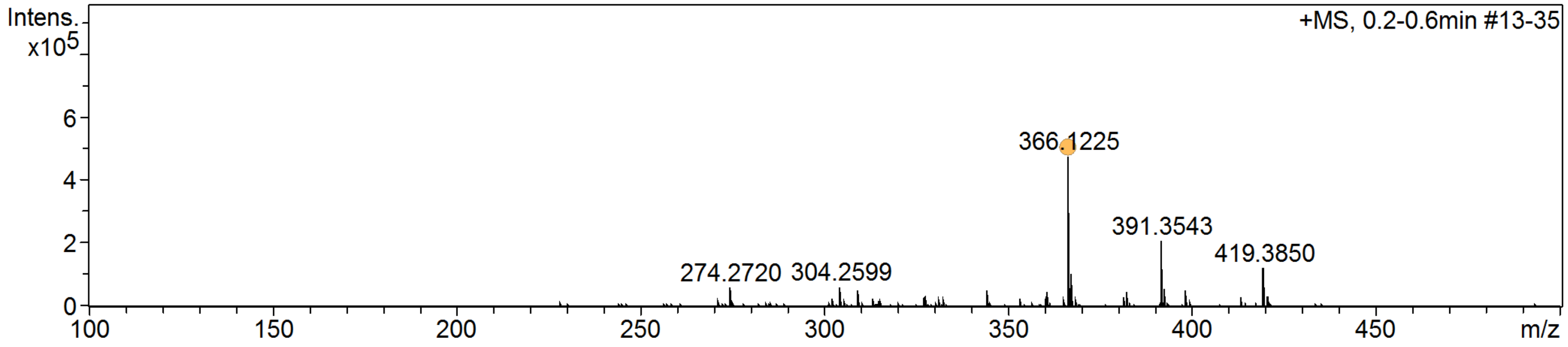

5b: calcd for $C_{18}H_{18}FN_3O_3$+Na 366.1224, found 366.1226.

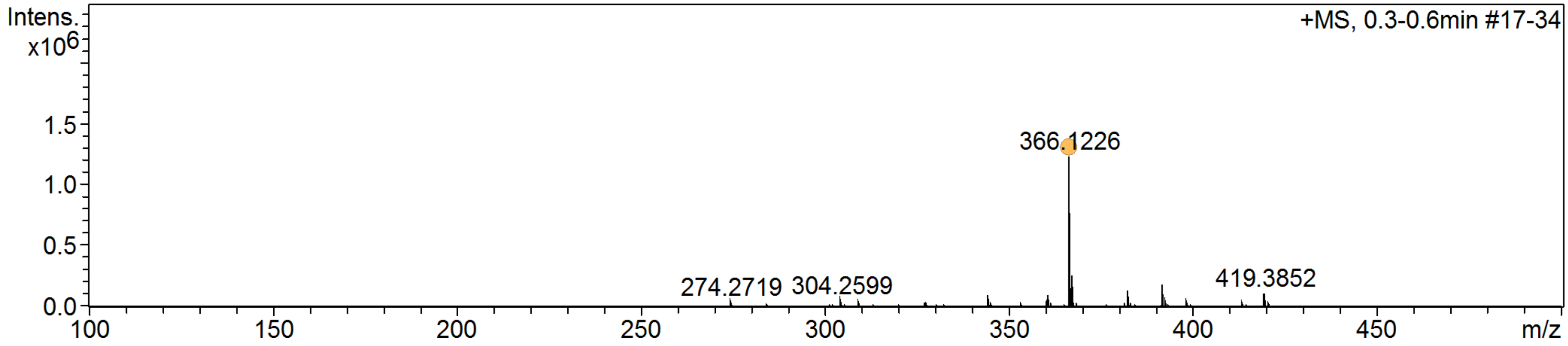

5c: calcd for $C_{18}H_{18}FN_3O_3$+Na 366.1224, found 366.1228.

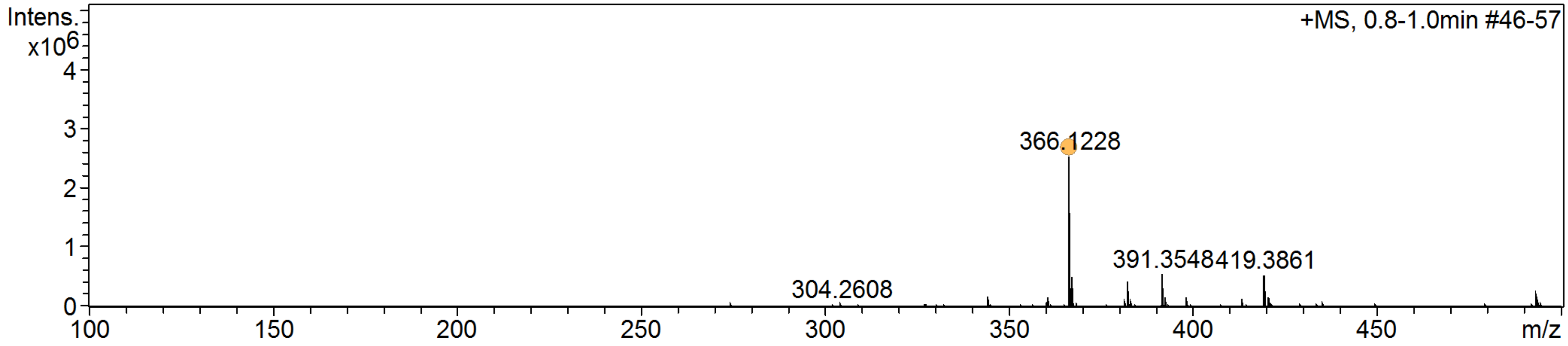

5d: calcd for $C_{18}H_{18}ClN_3O_3$+Na 382.0929, found 382.0933.

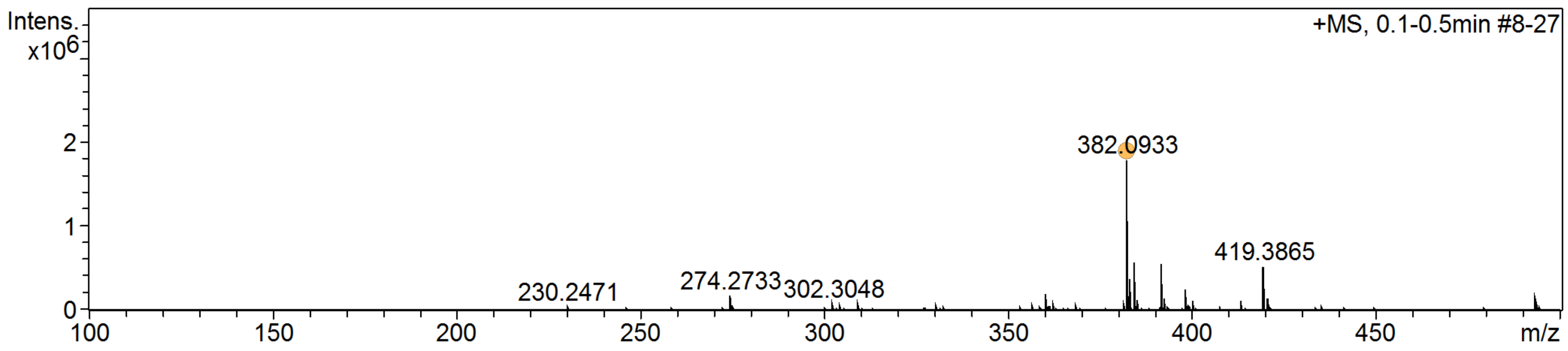

5e-1: calcd for $C_{18}H_{17}I_2N_3O_4$+Na 615.9201, found 615.9203.

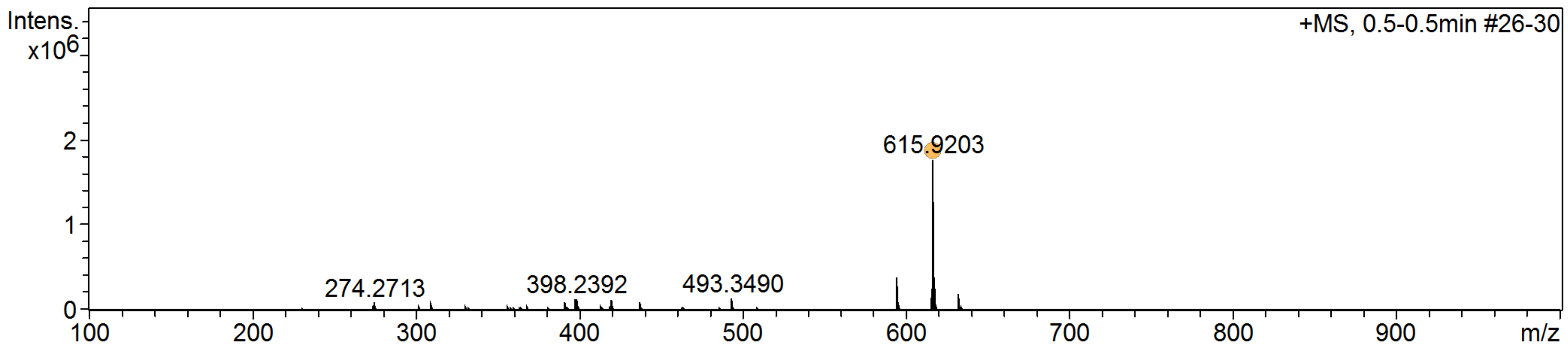

5f-1: calcd for [$C_{18}H_{15}I_4N_3O_4$ –I_2+H_2]+Na 615.9201, found 615.9213.

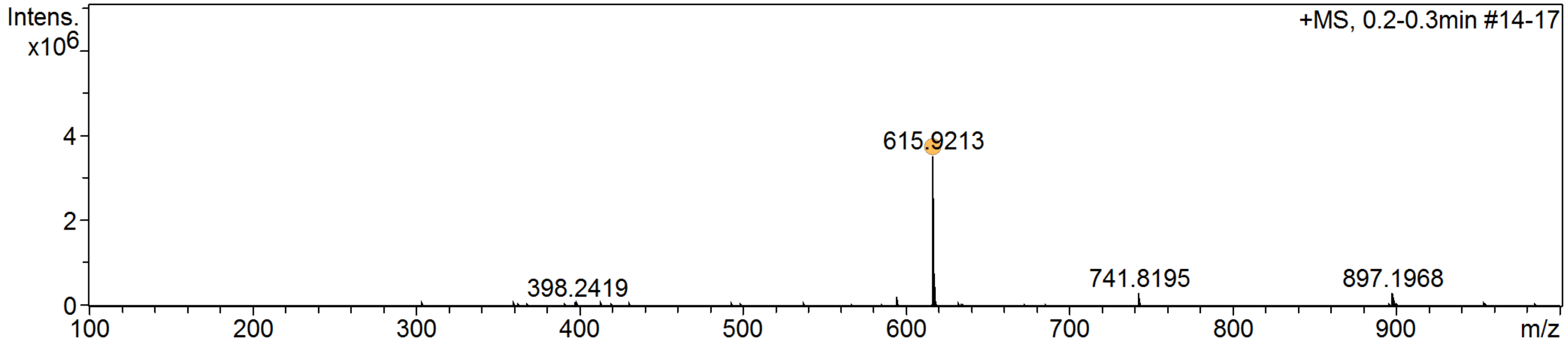

5f: calcd for $C_{18}H_{19}N_3O_4$+Na 364.1268, found 365.1271.

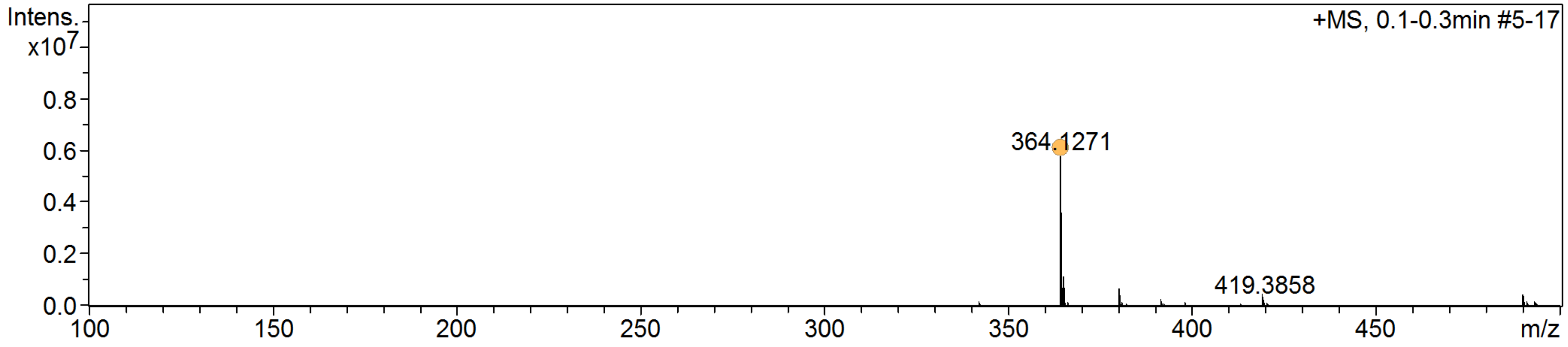

5g: calcd for $C_{19}H_{21}N_3O_4$+Na 378.1424, found 378.1428.

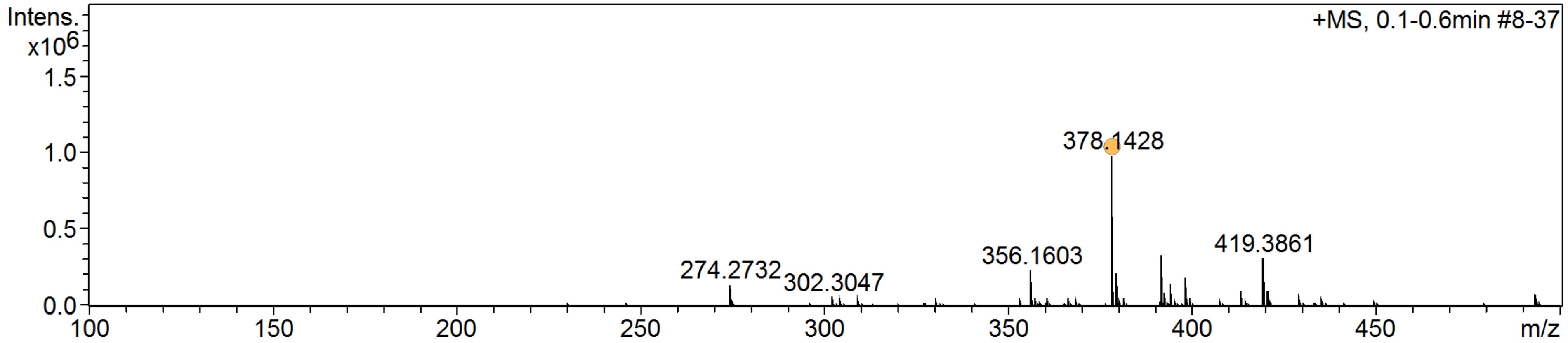

5g-1: calcd for $C_{19}H_{19}I_2N_3O_4$+Na 629.9357, found 629.9363.

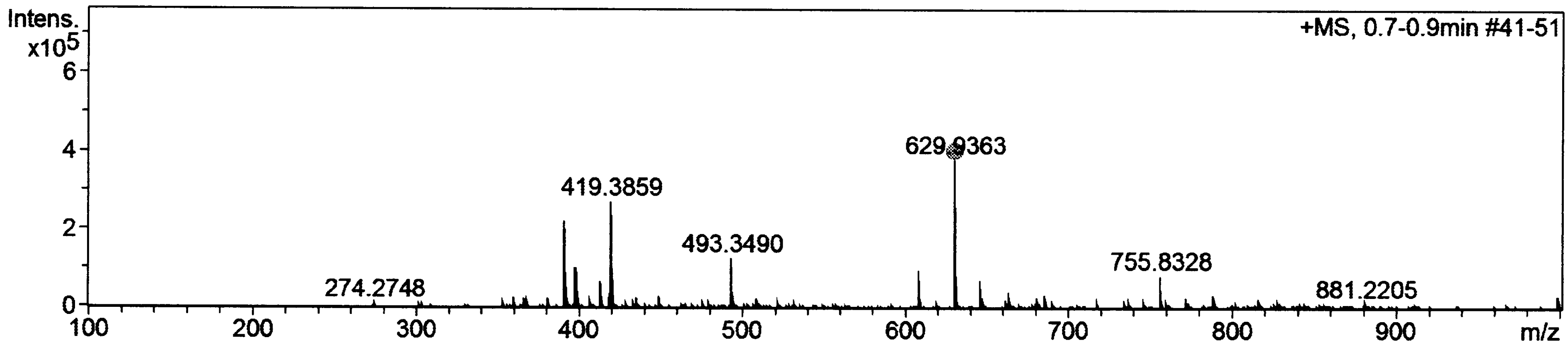

5h: calcd for $C_{19}H_{21}N_3O_4$+Na 378.1424, found 378.1426.

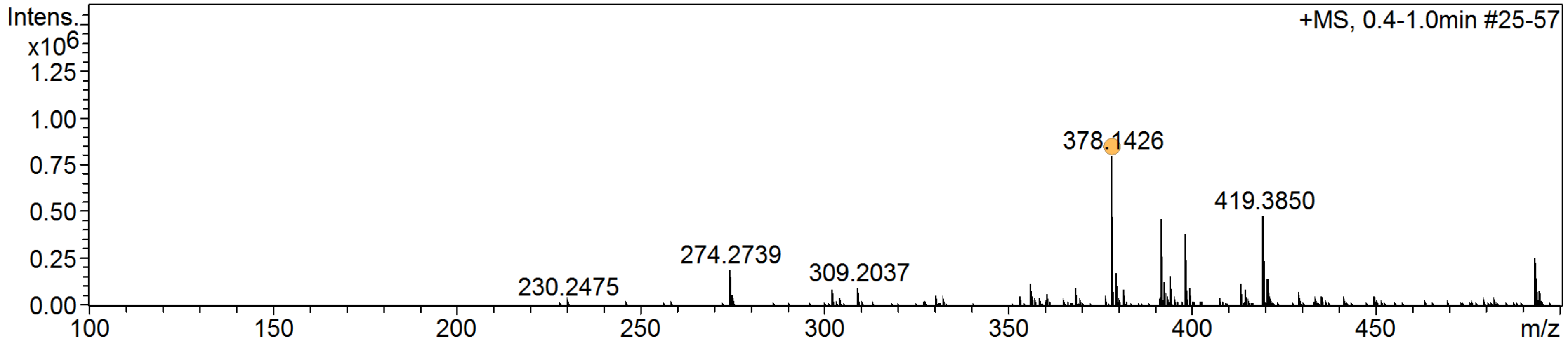

5h-1: calcd for $C_{19}H_{20}IN_3O_4$+Na 504.0396, found 504.0387; calcd for $C_{19}H_{19}I_2N_3O_4$+Na 629.9357, found 629.9358.

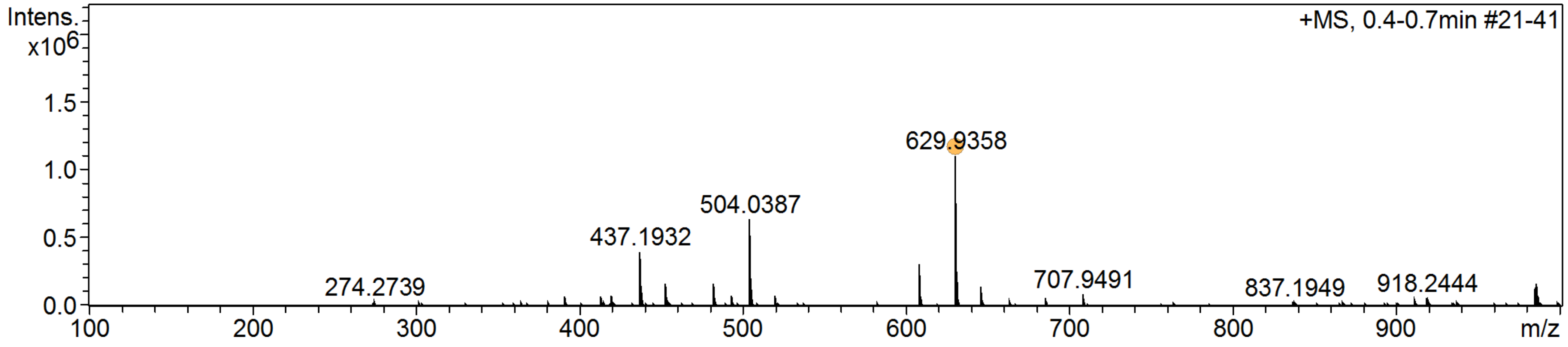

6a: calcd for $C_{19}H_{20}FN_3O_3$+Na 380.1381, found 380.1386.

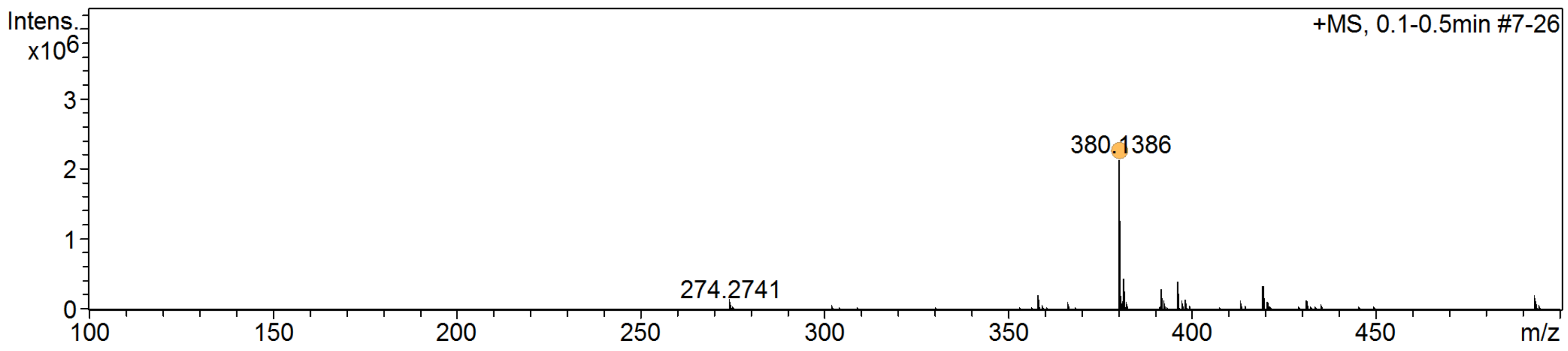

6b: calcd for $C_{19}H_{20}FN_3O_3$+Na 380.1381, found 380.1384.

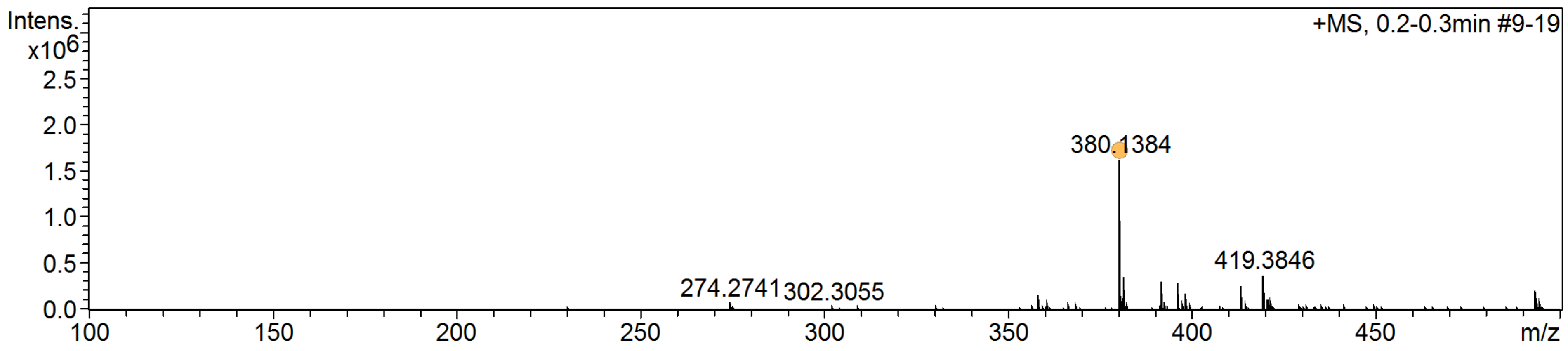

6c: calcd for $C_{19}H_{20}FN_3O_3$+Na 380.1381, found 380.1387.

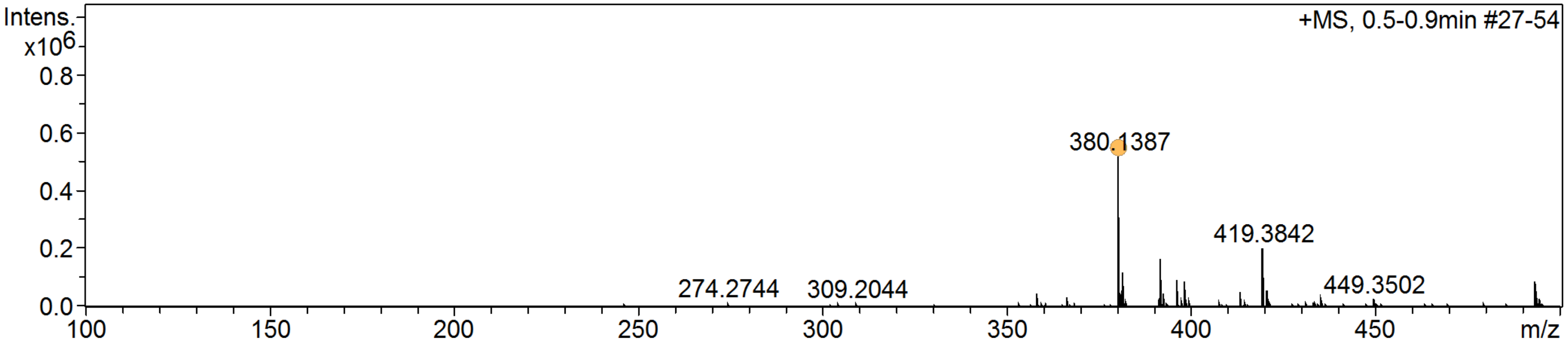

6d: calcd for $C_{19}H_{20}ClN_3O_3$+Na 396.1085, found 396.1089.

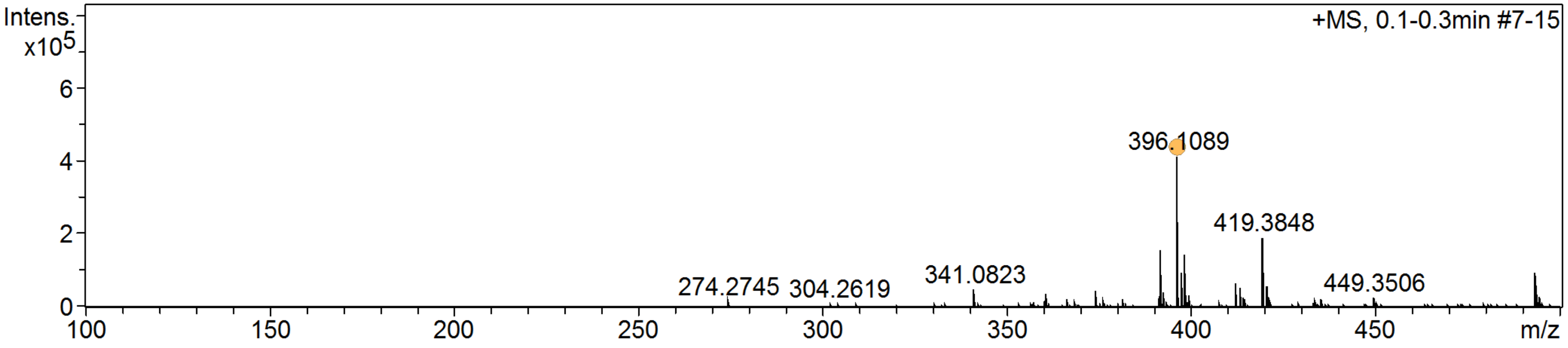

6e-1: calcd for $C_{19}H_{19}I_2N_3O_4$+Na 629.9357, found 629.9358.

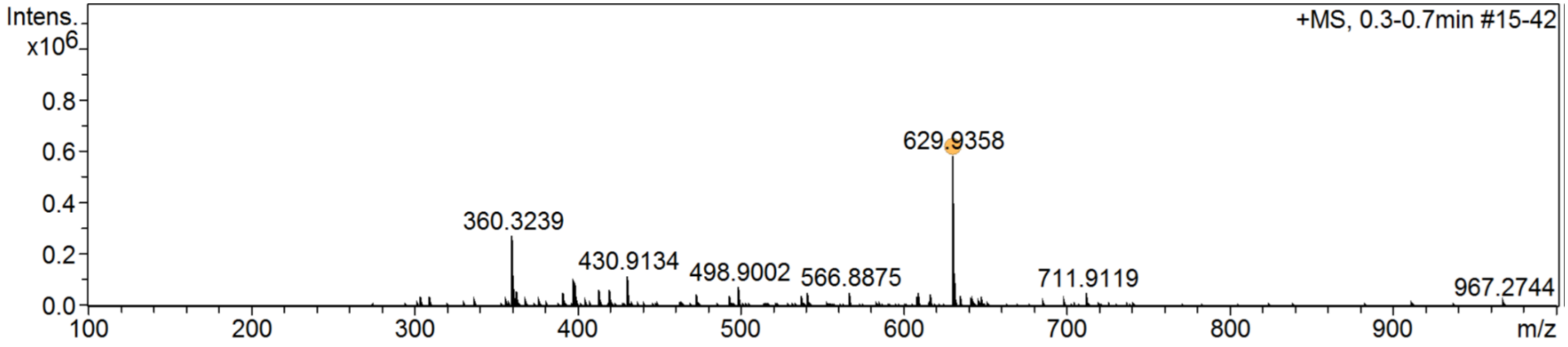

6e: calcd for $C_{19}H_{23}N_3O_4$+Na 380.1581, found 380.1586.

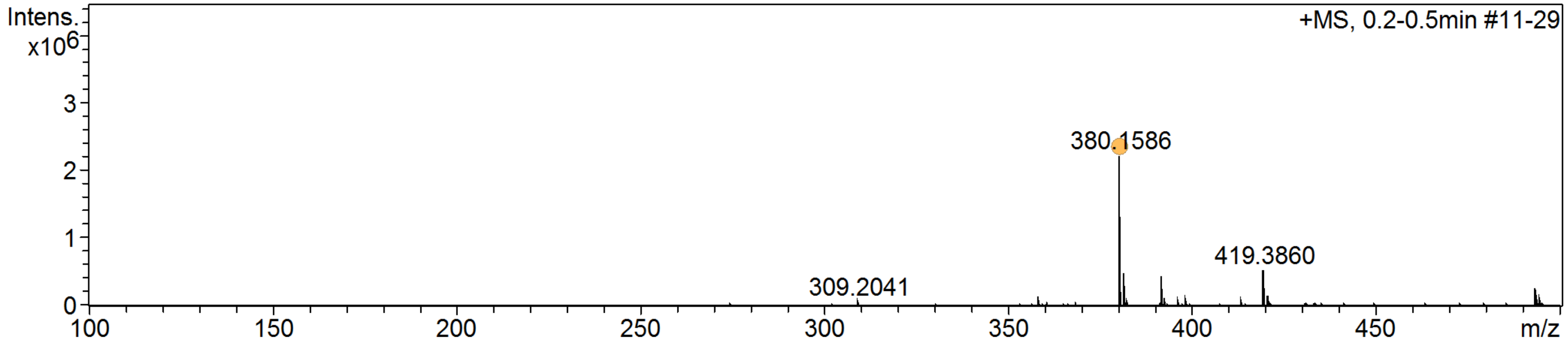

6f-1: calcd for $C_{19}H_{19}I_2N_3O_4$+Na 629.9357, found 629.9360.

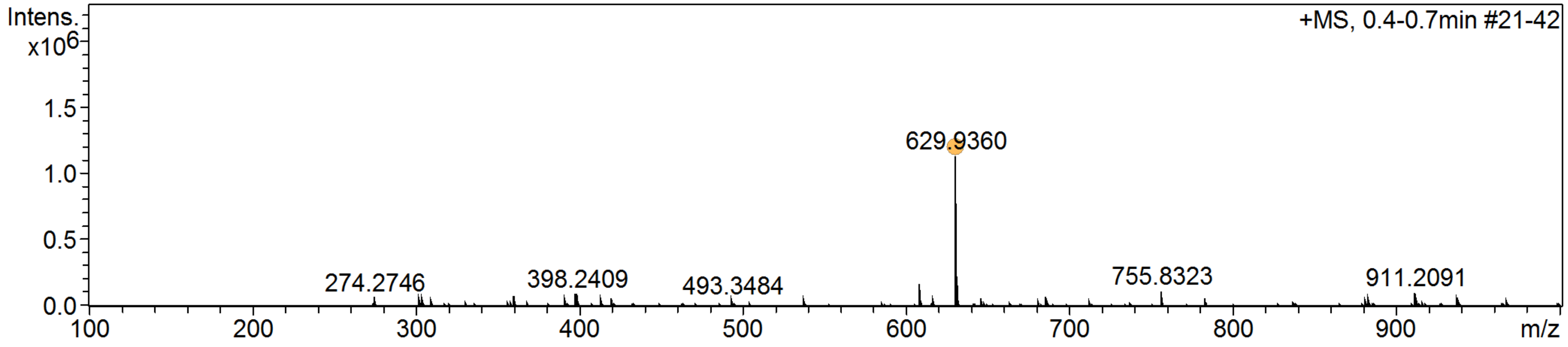

6f: calcd for $C_{19}H_{21}N_3O_4$+Na 378.1424, found 378.1427.

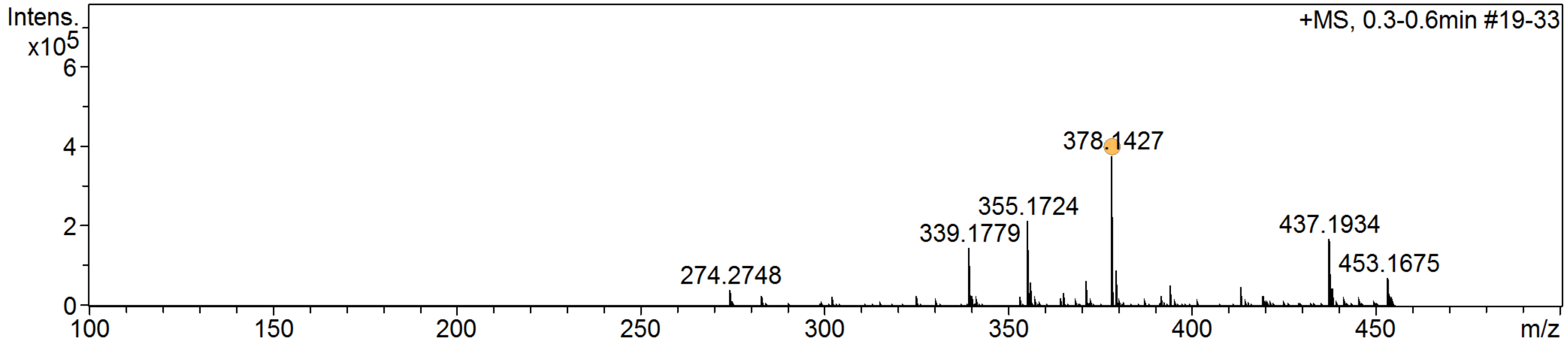

6g: calcd for $C_{20}H_{23}N_3O_4$+Na 392.1581, found 392.1585.

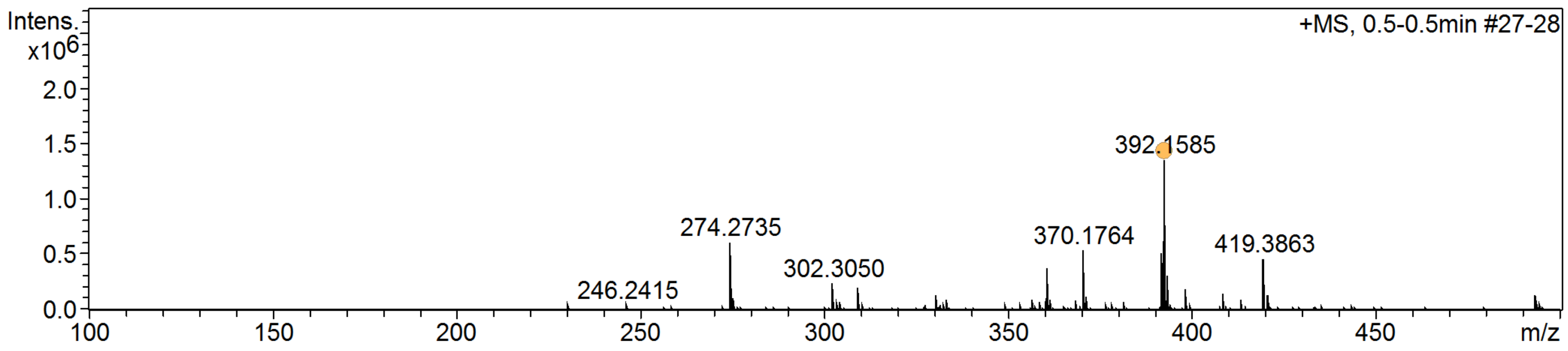

6h: calcd for $C_{20}H_{23}N_3O_4$+Na 392.1581, found 392.1584.

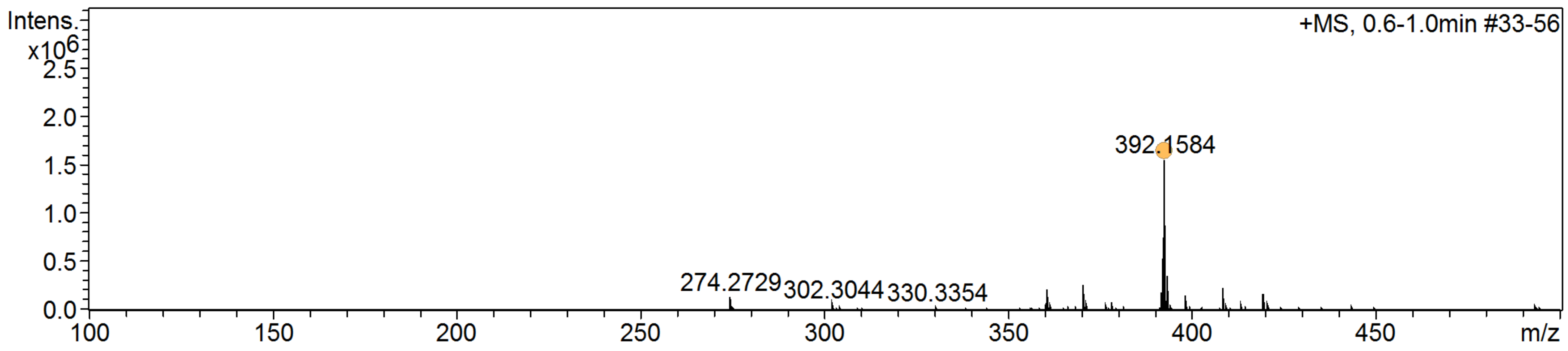

6h-1: calcd for $C_{20}H_{22}IN_3O_4$+Na 518.0547, found 518.0548.

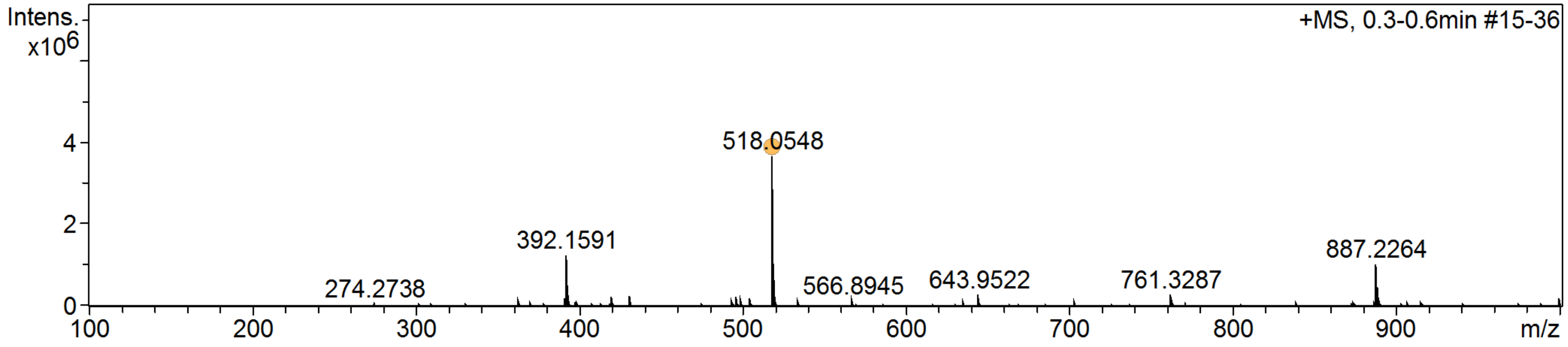

Supplement: Supplementary file 1 [file molecules-28-03638-s001.zip › molecules-2346728-supplementary.pdf]
